# Repeated Tree Inventories of Pine Forests in South Florida's Big Cypress National Preserve

**DOI:** 10.1002/ece3.70437

**Published:** 2024-10-23

**Authors:** Kenneth J. Feeley, Holly A. Belles, James R. Snyder

**Affiliations:** ^1^ Biology Department University of Miami Coral Gables Florida USA; ^2^ Douglass Texas USA; ^3^ Naples Florida USA

**Keywords:** cypress forest, everglades, *Myrica cerifera*, permanent plot, *Persea borbonia*, pine forest, *Pinus elliottii*, *Sabal palmetto*, *Taxodium ascendens*

## Abstract

The natural forest ecosystems of South Florida, USA, support a high biodiversity of plant and animal species and provide valuable ecosystem services. However, these ecosystems remain poorly represented in global studies, primarily due to a paucity of standardized data. Here, we present previously unpublished data from 332 censuses of 54 permanent 1‐ha tree inventory plots in the Racoon Point area of Big Cypress National Preserve, Florida, USA, including a total of nearly 100,000 measurements (diameter or height) of > 17,000 individual living trees and palms (with additional measurements of nearly 6000 dead pine snags) collected sporadically over a 19‐year period (1993–2012). These data, which were originally collected as part of a project to investigate tree responses to different experimental burning regimes, provide unique insight into the diversity, composition, structure, and dynamics of South Florida's unique and endangered pine forest ecosystems. Data files include the species identity, size (dbh = diameter at breast height), and location of all trees ≥ 5 cm dbh in 54 individual tree plots. Additional data are provided about heights of palm trees, and the location and burn history of each plot. These data are freely available for noncommercial scientific use under a Creative Commons license; users are encouraged to cite this paper when using the data.

## Graphical abstract

We present data from 332 censuses of 54 permanent 1‐ha tree inventory plots (Panel A) in the Racoon Point area of Big Cypress National Preserve, Florida, USA, including a total of nearly 100,000 diameter and height measurements of > 17,000 individual living trees (Panel B), with additional measurements of nearly 6000 dead pine snags,collected over a 19‐year period (1993–2012).

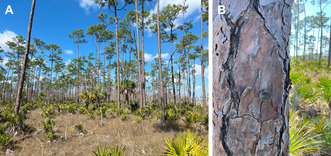

## 1 Introduction

Big Cypress National Preserve (BCNP) protects nearly 300,000 ha of pine forests, hardwood hammock forests, cypress swamps, swamp prairies, and mangroves/estuaries in southwest Florida, USA (Gunderson and Loope 1982). Combined, these natural habitats support more than 800 native plant species (> 140 plant families), including nearly 100 rare and endangered species (Black and Black 1980; Muss, Austin, and Snyder 2003). The distribution of habitats and species remains poorly studied but is commonly associated with differences in elevation, and flooding and fire regimes (Duever 2005; Snyder 1991).

The forest habitats of BCNP are dominated by Slash Pine (*Pinus elliottii* var. *densa*) and Cypress trees (Bald Cypress [*Taxodium distichum*] and Pond or Dwarf Cypress [*T. ascendens*; formerly *T. distichum* var. *imbricarium*]). Other notable tree species include Pond Apple (*Annona glabra*), Pop Ash (*Fraxinus caroliniana*), Red Maple (*Acer rubrum*), and Cabbage Palm (*Sabal palmetto*) (Black and Black 1980; Muss, Austin, and Snyder 2003). Prior to the establishment of the preserve, many of the cypress and pine forests of BCNP were logged (primarily for construction and shipbuilding). Today, the forests of BCNP are protected from direct exploitation, but they remain exposed to large‐scale environmental pressures such as climate change and alterations in regional hydrological and fire patterns.

As part of a long‐term study to document the ecological effects of possible fire management strategies on South Florida's pinelands, the National Park Service (NPS) and United States Geological Survey (USGS) established a network of 54 permanent 1‐ha tree inventory plots in the Raccoon Point area of BCNP (Snyder and Belles 2000). The main objective of the plot censuses was to provide detailed data on vegetation response to different burning regimes which could be used to refine prescribed burning programs on Department of Interior lands in South Florida.

## 2 Methods

**FIGURE 1:**
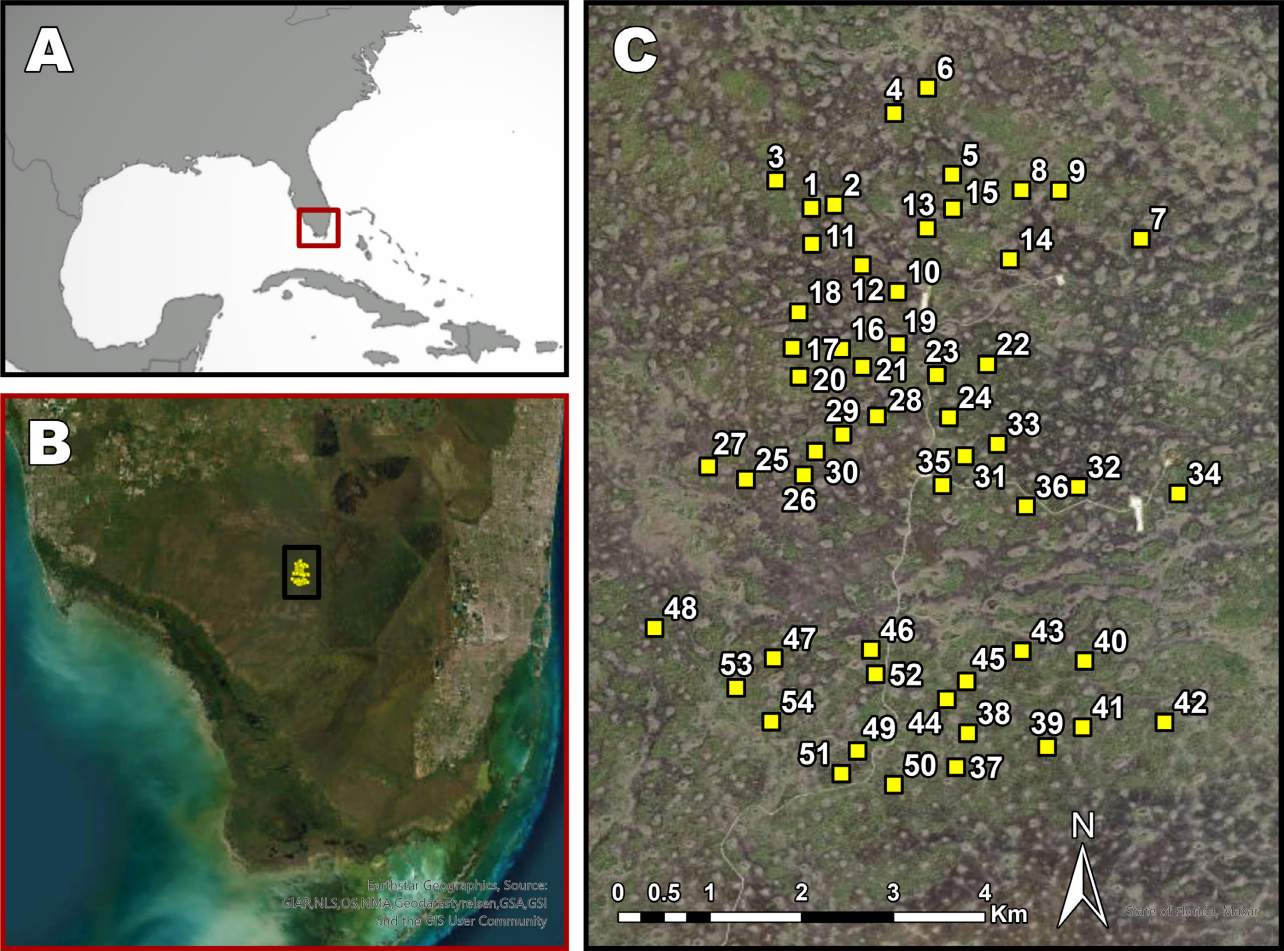
Maps showing the locations of the 54 one‐ha tree inventory plots in the Racoon Point area of Big Cypress National Preserve. Big Cypress National Preserve is located in (A) South Florida, USA. The Raccoon Point area is located in (B) the central portion of the Preserve. In Panel (C) the green areas are habitats dominated by pine forests, darker areas are flooded cypress prairies, and the round formations are flooded cypress domes. A map of the burn units is available in Snyder & Belles (2000). Maps were created by R. Fortier.

The Raccoon Point area in eastern Big Cypress National Preserve (Figure 1) is the largest remaining area of mature south Florida slash pine (*P. elliottii var. densa*) forest, having escaped the logging activities of 1900–1960 (Patterson and Robertson 1981). The pinelands consist of a mosaic of small, slightly elevated and dryer “islands” isolated among wetland swamp areas of dwarf cypress prairies and cypress domes. The isolation and inaccessibility of the Raccoon Point pine islands provided *de facto* protection against logging. However, the federal government did not acquire the mineral rights in Big Cypress when the preserve was created in 1974, and in 1977 Exxon Company, USA, constructed an all‐weather private access road running north from U.S. Highway 41 to multiple active oil well pads in the Raccoon Point oil field in BCNP.

**FIGURE 2:**
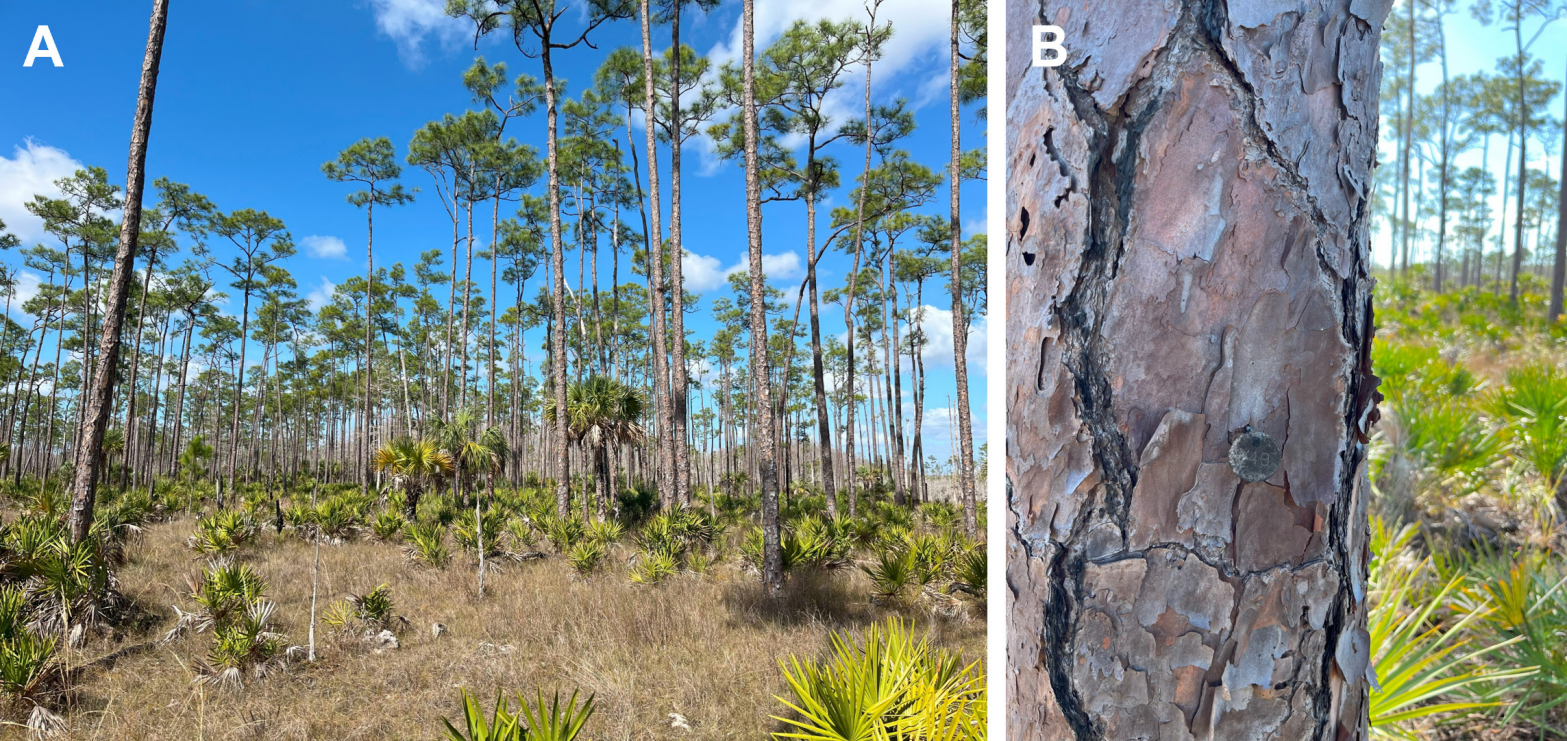
Photographs of (A) the pine forest habitat in the Racoon Point area of BCNP and (B) a tagged *Pinus elliottii* tree (Photograph by K. J. Feeley).

The study area surrounds the Raccoon Point oil field. It is divided into 18 experimental burn units. Each burn unit includes at least 50 ha of pine forest. Within each unit, three permanent 1‐ha tree plots were established (Figures 1 and 2) and repeatedly censused (Snyder and Belles 2000) starting in the early‐ to mid‐1990s.

**FIGURE 3:**
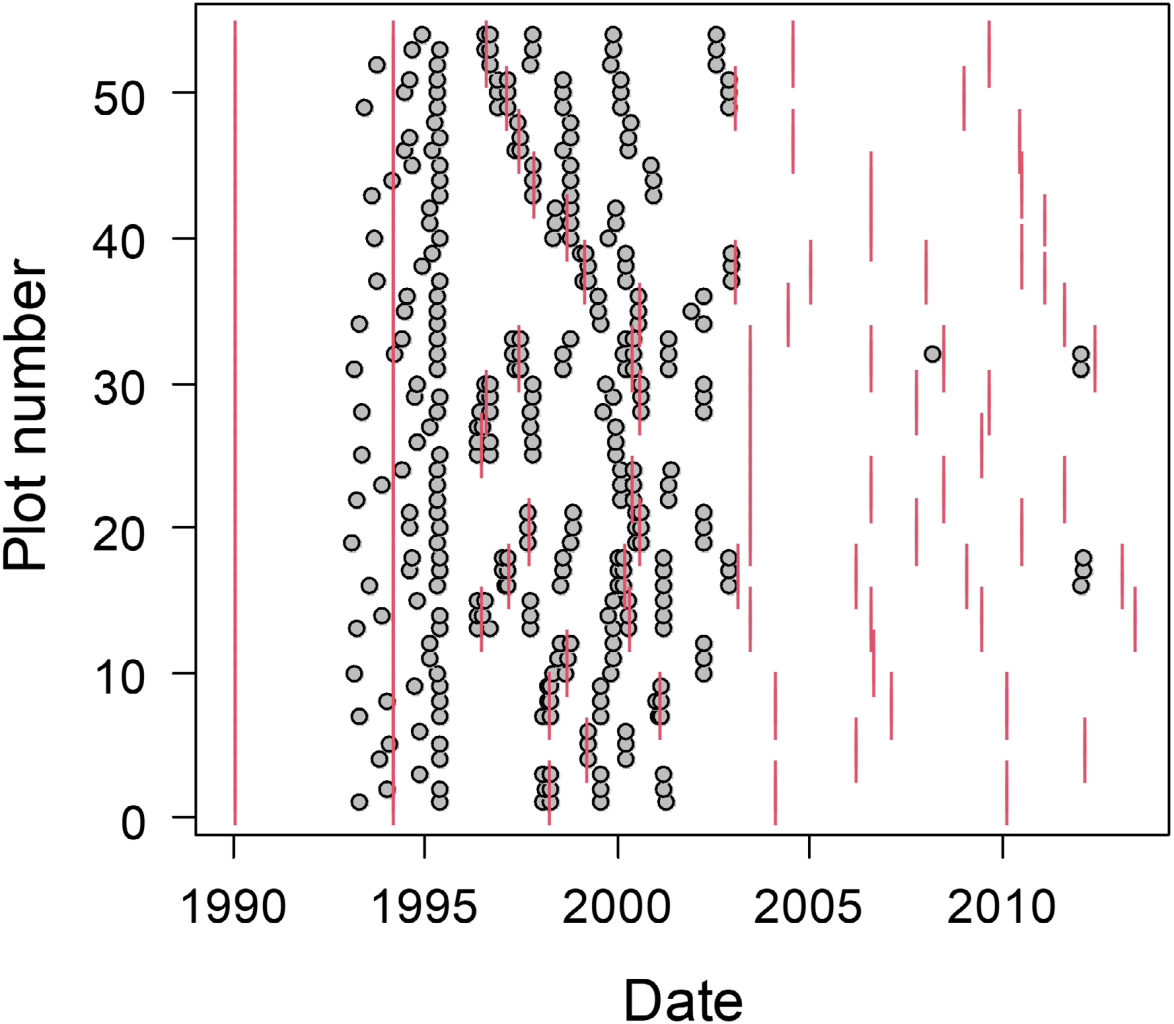
The census dates (gray circles) and burn dates (vertical red lines) for the 54 one‐ha tree inventory plots in the Racoon Point area of BCNP.

The experimental burn treatments consisted of burning at three seasons (spring, or early wet season [May to June], when the largest human‐caused or lightning‐caused wildfires occur; summer, or mid‐wet season [July to August], when there are frequent, but generally smaller, lightning‐ignited fires; and winter, or mid dry season [December 15 to February 15], when conditions are frequently favorable for prescribed burning) and two burn frequencies (every 3 years and every 6 years) for a total of six treatment combinations. Each treatment was replicated three times, with one replicate originally scheduled to burn per year for 3 years. Actual burn treatments did not always follow the prescribed schedule due to abnormally wet conditions, state‐wide burning bans brought on by drought conditions, or the occurrence of natural unplanned fires. All pinelands in the Raccoon Point study area were burned twice by the National Park Service as initializing burns before the start of this study (January 3 to February 9, 1990, and February 27 to April 4, 1994). Figure 3 shows the actual burn dates of each plot between 1996 and 2014, and a table of burn dates is included in the data files.

The permanent 1‐ha tree plots were all established between 1993 and 1995 (Snyder and Belles 2000; Figure 3). Within each burn unit, the tree plot locations were chosen by overlaying a grid on enlarged aerial photographs and randomly choosing points that fell within areas that appeared to be pineland. If it was subsequently determined during visits to the field that the area was not large enough to contain the 1‐ha plot, or if more than 10% of the area was dominated by cypress trees, another point was chosen. The final geographic coordinates of all 54 tree plots are included in the data files (coordinates indicate approximate plot centers).

Plots were oriented in a north–south direction so that the permanent markers would be easier to locate in the future. To set up each 1.0 ha tree plot, an east–west 100 m line was first established by driving 60 or 90 cm pieces of 1.3 cm diameter steel reinforcement bar (i.e., rebar) into the ground at 0, 25, 75, and 100 m. Two north–south 100 m lines were then established from the 25 and 75 m rebars using a double right‐angle prism to place 2 additional rebar stakes 50 m apart. The two remaining plot corners were put into place last. A total of 10 rebar stakes were placed in each plot. In most cases, a rotary hammer demolition drill was needed to secure the rebar in the very hard Tamiami limestone that is close to the surface throughout BCNP. Round aluminum tags with unit and plot numbers were wired to the four corner stakes of each tree plot. Each tree plot was then divided into quarters to make tree mapping more efficient. The quarters were identified by compass orientation: NW, NE, SW, and SE. Each quarter was further subdivided into halves. A 50 m tape was laid out along the center line that separated these two halves. A right‐angle prism was used to locate the position of each tree along the center line and a second tape was used to measure the distance from the center line to the tree. The field distances were subsequently adjusted to a single plot‐level XY cartesian coordinate system (Snyder and Belles 2000).

Within each plot, all tree stems with a diameter at breast height (dbh) ≥ 5.0 cm and palms with a height to the apical bud ≥ 1.4 m were tagged with a round, 3.2 cm diameter, pre‐numbered aluminum tag using a 5.4 cm aluminum nail. The tags were placed on live trees at breast height (approximately 1.4 m) and the diameter was measured just above the nail. Tags were placed in the most secure location on palm trees (i.e., a smooth area of the stem without remnant leaf bases). In addition to live trees, dead pine snags were also tagged and measured (Snyder and Belles 2000).

Data collected on individuals included tag number, XY cartesian coordinates within the plot, dbh, and species identity. For palms, the height to the apical bud was recorded rather than dbh since palm diameters do not increase with age. The heights of palms and some trees were measured using a telescoping pole up to a maximum of ~6 m; taller trees were not measured (with the exception of some uprooted, but still living, trees that could be measured with measuring tapes).

Tree plots were recensused between 3 and 10 times each, with the last censuses completed in 2012 (the last censuses for most plots were prior to 2003; Figure 3). During the plot recensuses, it was determined whether trees were living or had died since the previous census, and the dbhs of all living individuals were remeasured (along with dead pine snags). Additional notes were taken on features such as basal fire scars on pine trees. Tags were replaced as necessary. In addition, all new recruits with dbh ≥ 5.0 cm were measured and tagged. Attempts were made to census plots immediately before and after prescribed burns (Figure 3).

In 2024, the original tree census datasets were cleaned and reformatted to facilitate data sharing and statistical analyses. Data cleaning consisted primarily of correcting typographic and data entry errors (e.g., remove leading and trailing blank spaces, etc.). In addition, we screened the dbh data for outlier values; if any value appeared to be erroneous based on the other measurements of the same individual tree, we adjusted it accordingly (in the vast majority of these cases the incorrect values had been recorded in the wrong unit—i.e., 10× too small or too large—so the required corrections were obvious). Notable to the reformatting, since the dbhs of palms were not measured or recorded in the censuses, they were set to 35 cm and 10 cm for *Sabal palmetto* and *Serenoa repens*, which are the approximate mean dbh's for these species, respectively (Jones 1995). Also, all cypress trees were reassigned to *Taxodium ascendens* in accordance with updated nomenclature. While the censuses did measure dead pine snags, the dbhs for all dead trees were set to “NA” in the reformatted data files to minimize the risk of including dead trees in aboveground biomass or growth estimates. The original dbh values for all stems are included, and the original datasets are provided as separate *.xls files.

## 3 Results

Across all plot census (*N* = 332), there were a total of 128,032 tree measurements. These include 80,633 dbh measurements of 13,949 living trees, 16,674 height measurements of 3163 living palms, and 30,725 dbh measurements of 5881 dead pine snags.

**FIGURE 4:**
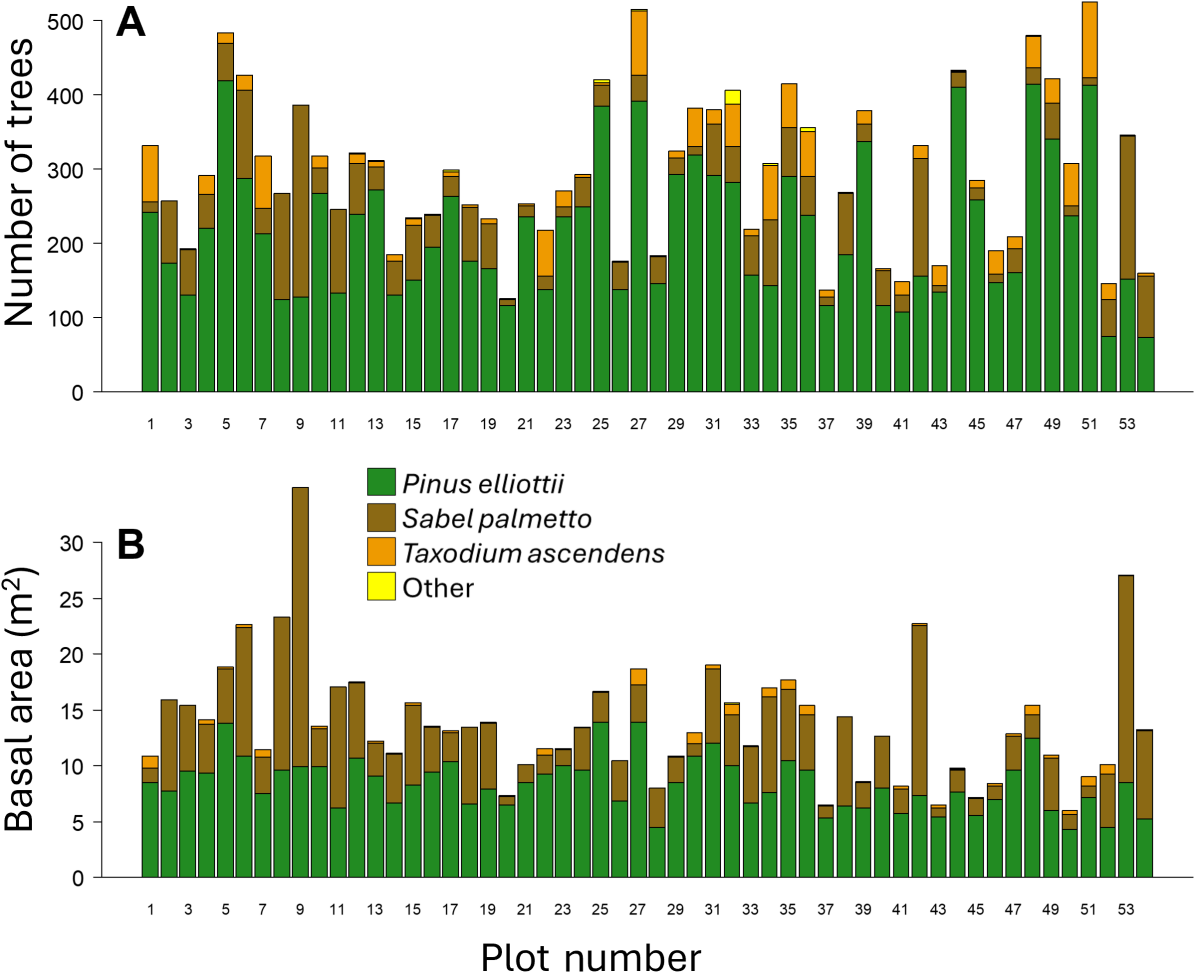
Bar charts showing (A) the mean stem density (stems ha^−1^) and (B) the mean basal area (m^2^ ha^−1^) in each of the 54 one‐ha tree inventory plots in the Racoon Point area of BCNP. Stem counts and basal area estimates are for living trees and were averaged across all censuses in each plot.

The mean stem density of plots (averaged across all available censuses) ranged from 123 to 531 living stems ha^−1^, with an overall mean of 295.5 stems ha^−1^. The total stem basal area (i.e., the cross‐sectional area at breast height) of living trees in the plots ranged from 5.78 m^2^ ha^−1^ to 35.26 m^2^ ha^−1^ (averaged across censuses), with an overall mean of 13.81 m^2^ ha^−1^ (Figure 4). Plot basal area estimates include *S. palmetto* and *S. repens* stems for which dbhs were all set to 35 and 10 cm, respectively.

In accordance with study design (Snyder and Belles 2000), *Pinus elliottii* accounted for the majority of stems and basal area in the plots. Specifically, *P. elliottii* accounted for between 32.28% and 95.06% of stems (mean = 73.49%) and between 28.14% and 90.41% of living basal area (mean = 64.71%). The next most abundant tree species were *Sabal palmetto* and *Taxodium ascendens. Sabel palmetto* accounted for between 1.89% and 67.72% of stems (mean = 19.16%) and between 7.38% and 71.86% of basal area (mean = 32.98%; keeping in mind that the dbh of *S. palmetto* stems were all set to 35 cm). *T. ascendens* accounted for between 0% and 29.91% of stems (mean = 7.14%) and between 0% and 10.33% of basal area (mean = 2.45%) per plot. Only three other tree species were recorded in the plot censuses. These species were *Myrica cerifera*, *Persea borbonia*, and *Serenoa repens*. Combined, these three treelet species accounted for between just 0% and 6.53% of stems (mean = 0.21%) and between 0.00% and 1.42% of basal area (mean = 0.04%) per plot (Figure 4).

There were strong negative correlations between the abundance and basal area of *P. elliottii* and *S. palmetto* (abundance: Pearson's *R* = −0.87, *p* < 0.001; basal area: *R* = −0.99, *p* < 0.001). The abundance and basal area of *S. palmetto* was also negatively correlated with *T. ascendens* (abundance: *R* = −0.39, *p* = 0.003; basal area: *R* = −0.34, *p* = 0.014). In contrast, there were no significant correlations between the abundance or basal area of *T. ascendens* and *P. elliottii*.

Across all plots and censuses, the mean radial growth rate of *P. elliottii* was 0.34 mm year^−1^. Across all plots and censuses, the mean radial growth rate of *T. ascendens* was 0.58 mm year^−1^. The mean annual mortality of *P. elliottii* was 1.47% year^−1^. The mean mortality of *S. palmetto* was 0.79% year^−1^, and the mean mortality of *T. ascendens* was 3.83% year^−1^.

## 4 Usage Notes

The following data files are provided in comma‐separated values (*.csv) format:

RP.Plot_data.csv:

- TREEKEY: a unique identifier for each tree stem combining the unit, unitplot, and tree tag numbers.
- UNIT: The burn unit 1–18 containing the plot.
- UNITPLOT: The plot number 1–3 within each burn unit.
- PLOT: A unique plot number between 1 and 54.
- TREETAG: Number recorded on the tree tag; tag numbers are repeated between plots.
- SP: Species binomial.
- XCOORD: East–West cartesian coordinate of tree within the plot (in m).
- YCOORD: North–South cartesian coordinate of tree within the plot (in m).
- DEAD: Code indicating if tree was alive (=0) or dead (=1) at the time of census.
- DBH: Diameter at breast height in cm.
- OBDAT: Observation date; date of measurement (yyyy‐mm‐dd).
- CENSUS: Number 1–12 indicating the census number. Since plots had different numbers of censuses and were not censused at the same time, CENSUS should not be used in place of OBDAT for temporal analyses.
- ORIGDBH: The dbh as recorded in the original census datafiles.
- HEIGHT: Height (m) of the tree or height of the palm's apical bud tip.

RP.Plot_locations.csv:

- UNIT: The burn unit 1–18 containing the plot.
- PLOT: A unique plot number between 1 and 54.
- LONGITUDE: Longitude (in decimal degrees; WGS84 Datum) of the approximate plot center.
- LATITUDE: Latitude (in decimal degrees; WGS84 Datum) of the approximate plot center.

RP.Plot_burndates.csv:

- UNIT: The burn unit 1–18 containing the plot.
- PLOT: A unique plot number between 1 and 54.
- BURNDATE: Recorded date of fire affecting the plot (yyyy‐mm‐dd).

In addition, we include the original plot census data files for each burn unit (in *.xls format) and a report (Snyder and Belles 2000) with expanded background and methods (*.pdf) in the file “RP.Original_Documents” folder.

## Author Contributions

**Kenneth J. Feeley:** data curation (lead), formal analysis (lead), writing – original draft (lead), writing – review and editing (lead). **Holly A. Belles:** conceptualization (supporting), investigation (supporting), methodology (supporting). **James R. Snyder:** conceptualization (lead), funding acquisition (lead), methodology (lead), project administration (lead), writing – review and editing (supporting).

## Conflicts of Interest

The authors declare no conflicts of interest.

## Code Availability

No custom code has been used in this study.

## Supporting information


Appendix S1

## Data Availability

All data are included as Supporting Information and are also accessible through Data Dryad (https://datadryad.org; doi: 10.5061/dryad.m0cfxppcf). These data are freely available for noncommercial scientific use under a Creative Commons license; users are encouraged to cite this paper when using the data.
