# Supplementary material for: Repeated Tree Inventories of Pine Forests in South Florida's Big Cypress National Preserve
Source: Ecol Evol. 2024 Oct 23;14(10):e70437. doi: 10.1002/ece3.70437 (PMC11496903; doi:10.1002/ece3.70437)
Supplement: Supplementary file 1 — Appendix S1 [file ECE3-14-e70437-s001.zip › Snyder_and_Belles.2000.Raccoon_Point_project_description.pdf]

Long-term Study of Fire Season and Frequency in Pine Forest and Associated Cypress Wetlands, Big Cypress National Preserve: Project Description and Preliminary Data

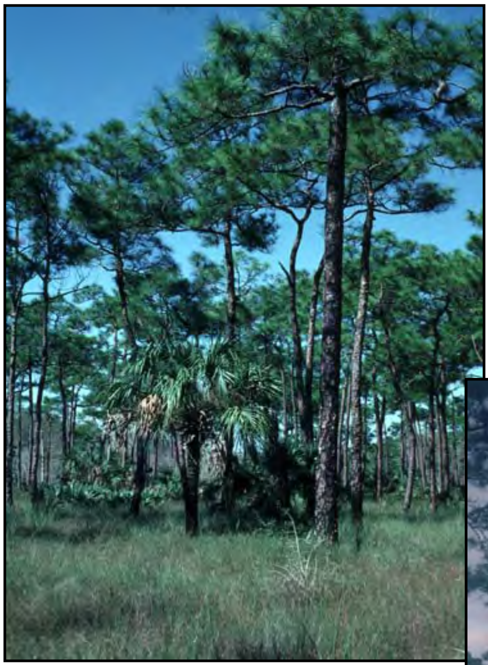

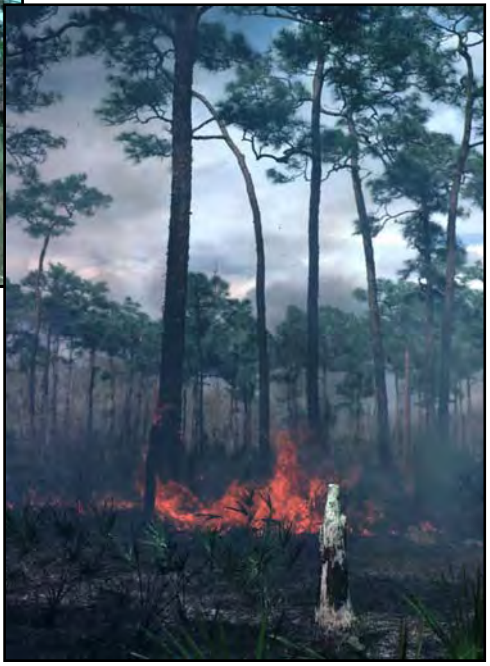

James R. Snyder
Principal Investigator
Holly A. Belles
Ecologist

U. S. Geological Survey
Biological Resources Division
Florida Caribbean Science Center
Big Cypress National Preserve Field Station
Ochopee, Florida 34141

June 2000

SUMMARY

South Florida slash pine forests represent one of the region's most fire-dependent and imperiled ecosystems. While lightning-ignited fires during the May to July period surely burned substantial areas before the arrival of Europeans, indigenous people had most likely been burning at other seasons for thousands of years. Even in large natural areas like Everglades National Park, a lightning-driven fire regime cannot be allowed to operate because of human health and safety concerns. Prescribed fire will be required to restore and maintain South Florida pinelands.

This long-term study will document the ecological effects of a wide range of potential fire management strategies on South Florida pinelands. The main objective of the project is to establish the baseline conditions and begin the experimental treatments for a long-term study of season and frequency of burning. The research will provide detailed data on vegetation response to different burning regimes that will be used to refine prescribed burning programs on Department of Interior lands in South Florida. Because many of the effects of fire regime will not become evident until after several repetitions of the experimental treatments, this study must be treated as a long-term ecological study.

The experimental study has been set up in the Raccoon Point area of eastern Big Cypress National Preserve, where the most extensive unlogged stands of South Florida slash pine (*Pinus elliottii* var. *densa*) remain. The pinelands exist as a mosaic of slightly elevated "islands" within a matrix of cypress domes and dwarf cypress prairies. The study site surrounds the Raccoon Point oil field and is divided into 18 experimental burn units. Each burn unit includes at least 50 ha of pine forest. Within each unit, three permanent 1.0 ha tree plots were established. Smaller 0.1 ha vegetation plots are located in the center of each tree plot and at two additional locations to sample herbaceous and shrubby vegetation. There are a total of 54 tree plots and 90 vegetation plots.

The experimental treatments consist of burning at three seasons (spring, or early wet season, when the largest human-caused or lightning-caused wildfires occur; summer, or mid wet season when there are frequent, but generally small, lightning-ignited fires; and winter, or mid dry season when conditions are frequently favorable for prescribed burning) and two frequencies (every 3 years and every 6 years) for a total of six treatment combinations. Each treatment is replicated three times, with one replicate being burned per year for three years. All the experimental prescribed burns are conducted by the Big Cypress National Preserve Fire Management Division. Beginning in 1996, two units have been burned at each season, representing both short- and long-frequency treatments. Some treatment burns have been postponed to subsequent years because of abnormally wet conditions or because of state-wide burning bans brought on by drought conditions. By spring 2000, all 18 of the initial experimental prescribed burns were completed and the second cycle of burns of the 3-year treatments was begun.

Some preliminary data from the tree plots are included in this report.

CONTENTS

Page

SUMMARY………………………………………………………………………………. iii

LIST OF TABLES……………………………………………………………………… v

LIST OF FIGURES…………………………………………………………………... v

1.0 INTRODUCTION ……………………………………………………………….. 1

2.0 BACKGROUND………………………………………………………………. 1
2.1 Fire History…………………………………………………………….. 1
2.2 Fire Effects……………………………..……………………………. 6
2.3 Study Area…………………………………… ……………………………. 8

3.0 OBJECTIVES………………………………………………………………………. 10

4.0 METHODS………………………………………………………………………. 11
4.1 Experimental Design……………………………………………………… 11
4.2 Prescribed Burning Treatments………………………………………………… 12
4.3 Selection of Burn Units………………………………………………………… 13
4.4 Permanent Vegetation Plots……………………………………………………. 13
4.4.1 Tree Plots……………………………………………………………. 14
4.4.2 Understory Plots…………………………………………………... 14
4.5 Fire Behavior……………………………………………………………... 17

5.0 PROGRESS AND RESULTS……………………………………………………. 19
5.1 Burn Units…………………………………………………………………... 19
5.2 Permanent Plots……………………………………………………………... 21
5.3 Treatment Burns……………………………………………………………….. 21

6.0 PLANS……………………………………………………………………………… 26

7.0 LITERATURE CITED…………………………………………………………..... 29

8.0 ACKNOWLEDGEMENTS………………………………………………………… 33

9.0 APPENDICES……………………………………………………………….… 34
9.1 National Park Service prescribed burn plan for experimental burns…………… 34
9.2 Tree plot maps showing locations of all trees……………………………….. 58
9.3 Size-class distribution of pines in tree plots……………………… ………... 114

LIST OF TABLES

Page

Table 1. Fire records for Everglades National Park, 1948-1997……………………………. 3

Table 2. Fire records for Big Cypress National Preserve, 1979-1998………………………. 3

Table 3. Two possible designs for frequency of burning in long-term fire study…………… 12

Table 4. Area (ha) of Raccoon Point burn units by vegetation class………………………... 19

Table 5. Number of trees per hectare by tree plot………………………………………….. 23

Table 6. Pine tree statistics by tree plot…………………………………………………….. 24

Table 7. Cypress tree statistics by tree plot………………………………………………… 25

Table 8. Burn schedule projected through 2007…………………………………………… 26

Table 9. Dates of completed burns………………………………………………………… 27

LIST OF FIGURES

Page

Figure 1. Seasonal distribution of fires, by cause, Everglades National Park, 1948-1997…... 4

Figure 2. Area burned by fire in Big Cypress National Preserve, 1979-1988 and 1989-1998 5

Figure 3. Location map, Raccoon Point long-term fire ecology project……………………. 9

Figure 4. Tree plot layout………………………………………………………………….. 15

Figure 5. Understory plot layout. Only points 1 and 11 as examples……………………..… 16

Figure 6. Temperature plate showing melting temperatures in degrees Fahrenheit…………. 18

Figure 7. Vegetation map showing burn unit boundaries…………………………………... 20

Figure 8. Map of tree plot and vegetation plot locations…………………………………… 22

Figure 9. Map of burn units showing treatments…………………………………………… 28

1.0 INTRODUCTION

A long history of lightning- and human-caused fire in south Florida has resulted in fire-dependent ecosystems over most of the region (Wade et al. 1980). Herbaceous wetlands, including the famous "river of grass", dominate the south Florida landscape, but upland pine forests and, to a lesser degree, cypress swamps also are fire-maintained.

South Florida contains a vast area of public lands subject to wildland fire, with the National Park Service managing over 5,000 km^2 of such lands in Big Cypress National Preserve and Everglades National Park. Other publicly owned lands with fire-dependent vegetation include the Florida Panther National Wildlife Refuge and the National Key Deer Refuge managed by the Fish and Wildlife Service and Fakahatchee Strand State Preserve and Collier-Seminole State Park managed by the Florida Department of Environmental Protection.

Fire management, both wildfire suppression and prescribed fire, is a major natural resource management activity throughout the area. The oldest prescribed fire program in the National Park Service began in Everglades National Park in 1958 (Taylor 1981) and today the majority of prescribed fire conducted by the National Park Service is in south Florida. Big Cypress National Preserve has developed the largest prescribed burning program in the National Park Service, burning an average of over 15,000 ha per year, primarily in prairies and pinelands.

The U.S. Geological Survey, in cooperation with the National Park Service, has initiated a long-term fire ecology project, the overall objective being to evaluate the effects of different fire regimes on South Florida pine forests. The study design consists of the application of prescribed fire at different seasons and frequencies in unlogged pineland in the Raccoon Point area of Big Cypress National Preserve. The information collected includes fire behavior data and detailed measurements of vegetation structure and species composition. The plots established for this study are, in effect, permanent vegetation monitoring plots that allow documentation of changes brought about by experimental prescribed fire regimes. Because many of the effects of fire regime will not become evident until after several repetitions of the experimental treatments, this must be treated as a long-term ecological study (Strayer et al. 1986, Likens 1989). Examples of this type of research include a more than 40-year study by the U.S. Forest Service in South Carolina (Waldrop et al. 1992) and a study begun in 1981 in St. Marks National Wildlife Refuge in the panhandle of Florida (Streng et al. 1993). The current study is also intended to provide a framework for other fire-related studies, including those relating to wildlife habitat values and life-history studies of endemic plant species.

This report provides background on South Florida fire ecology and the Raccoon Point study area, describes the experimental design and field methods, and presents some data on the experimental burn units and the permanent tree plots. Subsequent reports will describe the understory vegetation and the fire behavior of the first cycle of experimental burns.

2.0 BACKGROUND

2.1 Fire History

Fire plays an important role in controlling the distribution and composition of plant communities in south Florida (Egler 1952, Robertson 1953, Craighead 1971, Wade et al. 1980, Duever 1984,

Snyder et al. 1990). The frequent occurrence of lightning-caused fires today is generally considered to reflect a pattern that has existed since long before the arrival of Europeans. The strongest biological evidence for relatively frequent fire before European arrival relates to the habitat requirements of some of the endemic plants, as pointed out by Robertson (1953). More than 15 species of herbs are listed as endemic to the Miami rock ridge pinelands by Avery and Loope (1980) and conditions favorable to these species rapidly decline as the fire-free interval increases. Within a few decades of fire exclusion, pinelands succeed to closed hardwood hammock that contains none of the endemic herbs (Robertson 1953, Alexander 1967, Loope and Dunevitz 1981).

Given the inability to reconstruct pre-settlement fire history from physical evidence in south Florida (Taylor 1980a), it appears that the best way to determine the details of the natural fire regime is to analyze recent fire history and fire effects and to make inferences about the pre-Columbian situation (Snyder 1991).

The longest-term data set on recent fire history in south Florida is derived from the fire records of Everglades National Park, going back to 1948. These data through 1997 (a 50-yr record) are summarized in Table 1 and presented graphically in Figure 1. The data summarize all fires inside the park irrespective of vegetation type. The wildfire statistics are dominated by fires in graminoid communities (prairies and sawgrass marshes) because these constitute the bulk of the burnable area. The total area of burnable vegetation was about 219,000 ha until 1989 legislation added the so-called East Everglades and brought the fire-influenced area to 263,000 ha (Everglades National Park 1991).

The number of lightning-caused fires shows a more or less symmetrical distribution that mirrors thunderstorm activity (Duever et al. 1986, Komarek 1964)and peaks in July (Figure 1). About 92% of the lightning-caused fires occur from May to September. Over 97% of the area burned by lightning-caused fire burns during the four months of May through August. Lower water levels and drier fuels at the beginning of the lightning-ignited fire season result in larger mean fire sizes during that period than later in the season. The average size of lightning-ignited fires in May-June is 809 ha vs. 237 ha in July-August. By September, the mean lightning-ignited fire size drops to 33 ha. Human-caused wildfires occur mainly during the dry season months and are strongly correlated with moisture conditions. The area burned peaks in May when fuels are likely to be dry and water levels lowest. Forty-two percent of the total area burned in Everglades National Park over the period of record was burned by human-caused and lightning-caused wildfire in May.

Prescribed burning was begun in 1958 in the pinelands of Everglades National Park and expanded to other fuel types in 1971 (Klukas 1973; Bancroft 1976, 1977; Taylor 1981). Most of this management burning was conducted during the cooler months until 1980, when there was a shift to wet-season burning in the pinelands to coincide with the lightning-ignited fire season (Doren and Rochefort 1984). Prescribed burning during the dry season continues for hazard fuel reduction burning along park boundaries and for research.

Comparable fire statistics are available for Big Cypress National Preserve only since 1979 (Table 2), when the National Park Service assumed fire control responsibilities from the Florida Division of Forestry (Taylor 1980b). Most of the preserve, about 295,000 ha within its expanded

Table 1. Fire records for Everglades National Park, 1948-1997. Data courtesy of John Segar, Everglades fire management.

Mean Number of Fires

Cause	JAN	FEB	MAR	APR	MAY	JUN	JUL	AUG	SEP	OCT	NOV	DEC	TOTAL
Human-Caused Wildfire	0.7	0.9	1.1	1.3	1.1	0.2	0.1	0.1	0.1	0.1	0.6	0.6	6.8
Prescribed Fire	0.8	0.8	0.7	0.5	0.5	0.3	0.6	0.5	0.4	0.8	1.1	0.8	7.6
Lightning Fire	0.0	0.0	0.1	0.2	0.9	1.6	2.3	1.9	1.0	0.3	0.0	0.0	8.4
**Total**	1.4	1.7	1.9	2.0	2.5	2.0	3.0	2.5	1.5	1.1	1.7	1.4	22.7

Mean Area Burned (ha)

Cause	JAN	FEB	MAR	APR	MAY	JUN	JUL	AUG	SEP	OCT	NOV	DEC	TOTAL
Human-Caused Wildfire	187.8	235.7	476.1	683.5	1,950.1	373.7	2.4	8.3	0.5	4.5	16.4	38.9	3,977.9
Prescribed Fire	188.5	246.0	153.6	61.4	46.5	42.1	152.6	107.9	76.7	95.9	287.3	143.0	1,606.5
Lightning Fire	0.0	0.0	9.8	30.0	1,459.8	562.3	258.4	142.8	34.2	4.5	0.8	0.0	2,502.6
**Total**	376.2	481.7	639.4	774.9	3,456.4	978.1	413.5	259.1	111.4	104.8	304.6	181.9	8,087.1

Table 2. Fire records for Big Cypress National Preserve, 1979-1998. Data from Snyder (1991) and Department of Interior database in Boise, Idaho.

Mean Number of Fires

Cause	JAN	FEB	MAR	APR	MAY	JUN	JUL	AUG	SEP	OCT	NOV	DEC	TOTAL
Human-Caused Wildfire	8.3	5.8	13.7	6.4	5.8	2.1	1.4	1.1	1.2	2.7	10.1	10.8	69.3
Prescribed Fire	3.6	2.5	1.8	1.1	1.1	1.1	0.8	0.7	0.5	0.8	1.0	2.1	16.8
Lightning Fire	0.0	0.1	0.2	0.4	2.1	4.6	4.2	2.8	1.1	0.1	0.3	0.1	15.8
**Total**	11.9	8.4	15.7	7.9	8.9	7.8	6.4	4.6	2.8	3.5	11.4	13.0	101.8

Mean Area Burned (ha)

Cause	JAN	FEB	MAR	APR	MAY	JUN	JUL	AUG	SEP	OCT	NOV	DEC	TOTAL
Human-Caused Wildfire	646.0	411.7	1,835.4	358.8	4,187.1	320.6	34.0	13.9	13.6	37.1	194.0	571.8	8,623.6
Prescribed Fire	2,070.5	1,793.3	1,329.5	394.0	503.2	1,215.8	445.6	339.2	269.0	394.8	212.2	840.1	9,807.0
Lightning Fire	0.0	15.8	0.3	7.7	44.9	193.2	54.0	24.6	12.5	0.3	0.2	0.1	353.3
**Total**	2,716.5	2,220.7	3,165.1	760.5	4,735.1	1,729.5	533.6	377.7	295.0	432.1	406.3	1,411.9	18,783.8

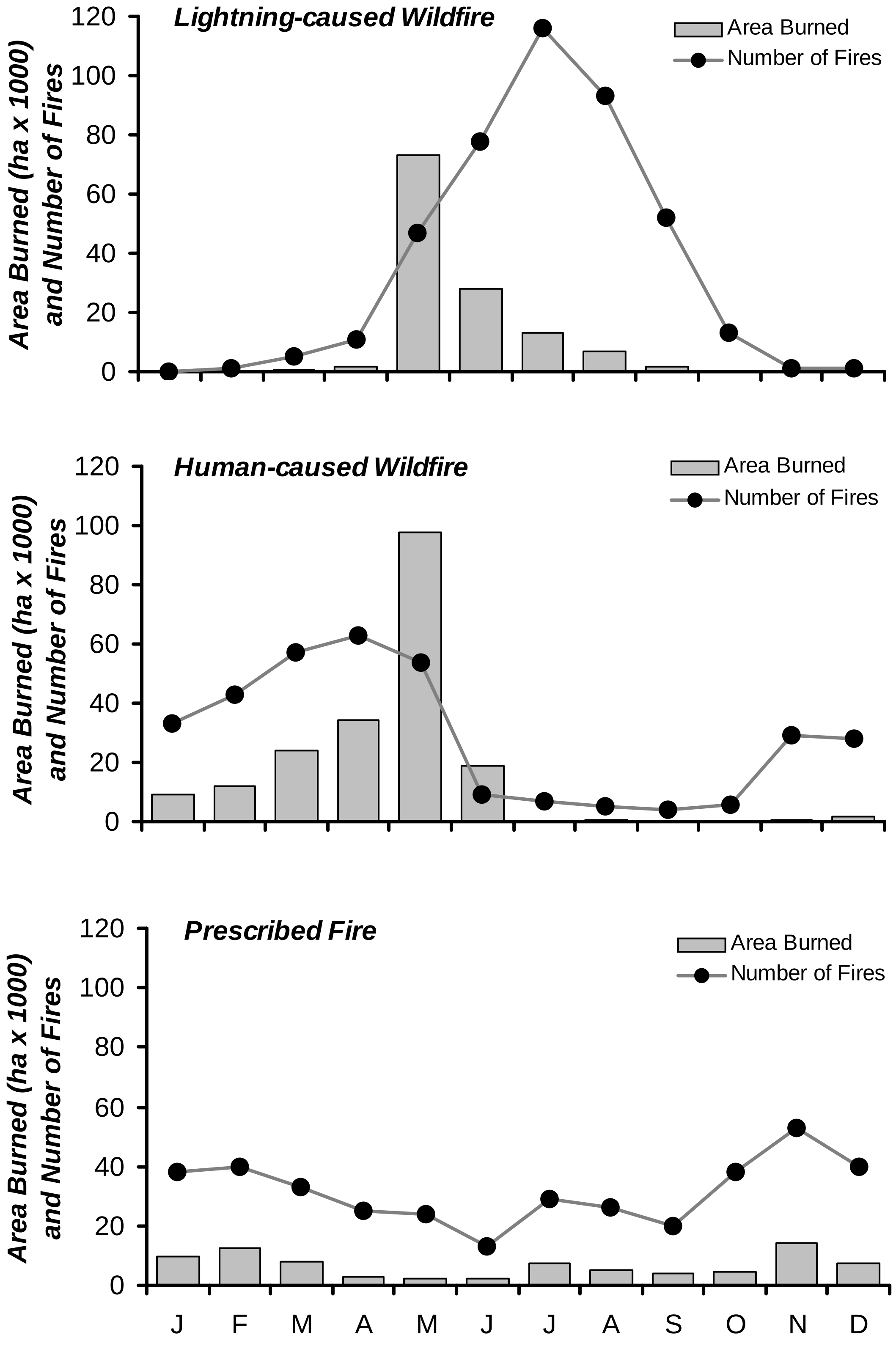

Figure 1. Seasonal distribution of fires, by cause, Everglades National Park, 1948-1997.

boundary, is covered by burnable vegetation so that it constitutes a larger potential area for fire than Everglades National Park.

One of the most obvious features of the Big Cypress fire data is the large number of fires (averaging over 100 per year) and the preponderance of human-caused wildfire (Table 2). Two-thirds of the 2,036 fires that occurred during the period 1979-1998 were intentional or accidental human-caused wildfires. The extremely high number of fires in Big Cypress is due largely to hunters and the presence of a few hundred backcountry camps (Taylor and Rochefort 1981). Particularly during the early years of the Preserve, much of the human-caused wildfire was intentionally set for informal wildlife habitat management, hazard reduction around camps, or to spite the government (Taylor 1980b, Taylor and Doren 1982). The peak in fire numbers that occurs in March has been attributed to human activities associated with the spring gobbler season. In addition, many a fire was accidentally started by a vehicle pulled off on the road shoulder of Alligator Alley.

There has been a shift from a preponderance of human-caused wildfire to dominance by prescribed fire over the last twenty years (Figure 2). The last year in which the area burned by wildfire exceeded the amount burned by prescribed fire was 1988. There has been a gradual shift in the habits of backcountry users, and the National Park Service has assumed an aggressive position in prescribed burning.

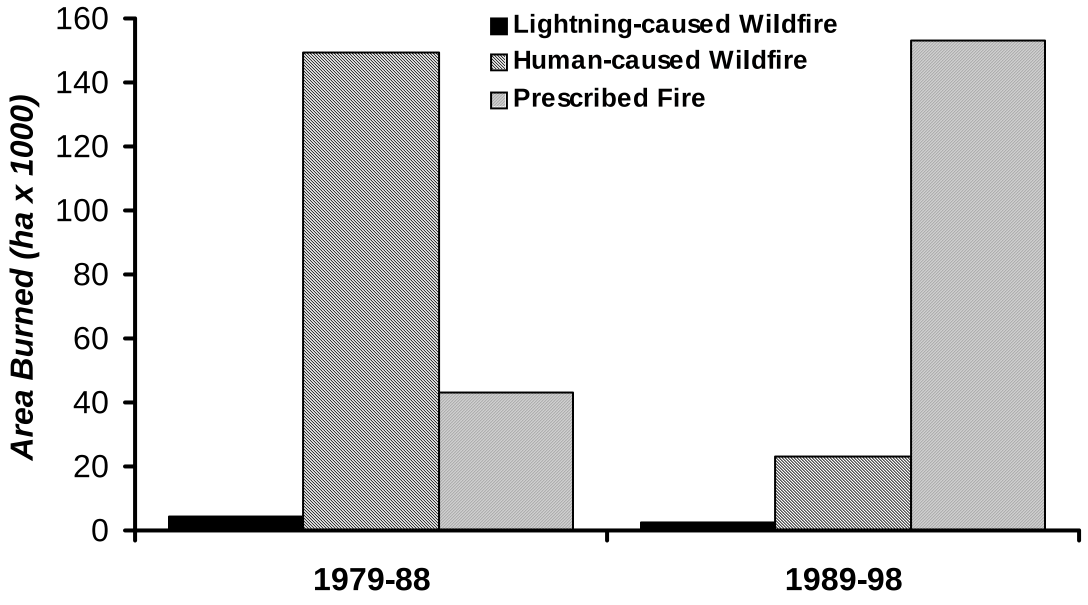

Figure 2. Area burned by fire in Big Cypress National Preserve, 1979-1988 and 1989-1998.

Another feature of the recent Big Cypress fire history is the minor contribution of lightning-caused fire. While lightning-ignited fire has been responsible for about 31% of the area burned in Everglades National Park (Table 1), lightning fire has burned <2% of the area burned in Big Cypress National Preserve during the 20-yr period of record (Table 2). The large amount of human-caused wildfire and prescribed fire may limit the spread of lightning-ignited fires.

The fire frequency for different Big Cypress habitats or fuel types varies considerably. Based on all fires during the 10-yr period from 1979-1988, the approximate fire-return intervals for major fuel types are 7 yr for both prairie/marsh and pineland, 24 yr for cypress prairie, 110 yr for cypress strand, and 177 yr for hammock (Snyder 1991). These numbers are not outside ranges that have been proposed for these vegetation types (Wade et al. 1980, Duever 1984), although they tend to reflect the upper limits. For example, 3-7 yr or as frequently as every 2 yr has been suggested as the fire frequency for south Florida pinelands (Wade et al. 1980). Duever et al. (1986) mentioned 3-10 yr between fires in Big Cypress pinelands. Ewel (1990) has suggested minimum fire return intervals >10 yr for cypress prairie and >20 yr for cypress domes and strands. Because the intervals presented by Snyder (1991) are based on such a brief period of record, the numbers are very tentative, especially for the fuel types with longer return intervals.

An important factor to be considered in inferring pre-Columbian fire regimes from present-day fire patterns is the possible influence of native Americans in shaping the south Florida landscape through fire. The general question of Indian burning and whether it should be treated as part of the "natural" fire regime has been widely debated (Lotan et al. 1985, Williams 2000). Even though historical accounts documenting Indian burning in south Florida are lacking, it seems that aboriginal influence should not be discounted (Myers and Peroni 1983).

In a review of Indian burning in Florida, Robbins and Myers (1992) found some, but not overwhelming, evidence that Indians practiced widespread burning. In northern Florida a good case has been made that the burning by prehistoric Indians maintained grassy islands of longleaf pine in the midst of sand pine scrub (Kalisz and Stone 1984, Kalisz et al. 1986). Both Egler (1952) and Robertson (1953) suggested that Indians increased the amount of fire in south Florida over the background level caused by lightning and that most Indian-caused fires occurred as early in the dry season as the grassy fuels would carry fire. It seems reasonable to suggest that relatively large areas also may have burned in the late dry season due to Indian-caused fires, much as large wildfires do today. Therefore, in pre-Columbian times, and perhaps as long as the south Florida environment has been similar to current conditions, it is likely that fire occurred at all times of the year, with the largest fires and greatest area burning during the spring and the least fire during late summer and fall, just as occurs now.

2.2 Fire Effects

Given the high incidence of fire in this environment, it is not surprising that most species, including the dominants, show an ability to resist or recover from fires. The pineland overstory species is south Florida slash pine, *Pinus elliottii* var. *densa* (Little and Dorman 1954). A very fire-tolerant and fire-adapted species, south Florida slash pine is the southernmost native and the only subtropical pine in the United States (Langdon 1963). Mature slash pines are fire resistant because of their thick bark and heavy, protected buds, as well as the fact that the generally open canopies do not carry crown fires. The distinctive seedlings of this variety (with very short stems, many crowded needles, thick hypocotyl, and thick tap root) also withstand fire better than those of the more widespread northern variety, *P. elliottii* var. *elliottii* (Ketcham and Bethune 1963). Cypress (*Taxodium ascendens*) also shows resistance to damage by fires and has the ability to sprout from the bole if branches are killed. The two common palm species, cabbage palm (*Sabal palmetto*) and saw palmetto (*Serenoa repens*), are highly resistant to fire because of their protected apical buds and monocotyledonous vasculature. Hardwoods typically resprout from below-ground parts if the stems are killed by fire.

Discussion of the ecological implications of different fire regimes for south Florida ecosystems has been presented by Wade et al. (1980) and Snyder et al. (1990). Robbins and Myers (1992) have thoroughly reviewed the literature on the effects of different seasons of burning in Florida.

Much of the research on the effects of season or frequency of burning has focused on specific management goals such as hardwood control in pine plantations or forage production, and almost all has been carried out in areas north of south Florida. A good example of this is a long-term study of prescribed burning in South Carolina coastal plain loblolly pine stands that has had results reported after 10 (Lotti et al. 1960), 20 (Lewis and Harshbarger 1976), 30 yr (Langdon 1981), and 41 yr (Waldrop et al. 1992) of treatments. The burning treatments, begun in 1946 and 1951, included annual burns and periodic burns (>3-yr intervals) in both summer and winter, and biennial summer burns. The study ended when the site was devastated by Hurricane Hugo in 1989. Interpretation of the results is not entirely straightforward because the treatments were sampled at different postburn ages. However, it is clear that summer burns result in reduced hardwood cover relative to winter burns done at the same frequency. The reduction is due to a combination of increased mortality and less vigorous regrowth. In fact, biennial summer burning is more effective than annual winter burning at reducing the number of stems and crown cover of woody undergrowth. The periodic summer burning reduced the cover of woody plants relative to winter burning in spite of producing a greater number of stems (Langdon 1981).

Research on hardwood response after fires in different seasons generally finds that growing-season fires result in more hardwood mortality, less resprouting, and topkill of more and larger stems than winter fires (Robbins and Myers 1992, Streng et al. 1993). In pinelands of subtropical south Florida, less is known about the seasonal effects of fire. Here the difference in ambient temperature between summer and winter is less than at higher latitudes and most of the hardwood understory species are evergreen, with a less pronounced winter dormancy than deciduous species. Higher fuel and soil moisture after the rains have begun in the summer should act to lessen the severity of growing-season fires. These factors may obscure or alter the effect of season of fire.

Two sets of paired summer and winter (wet- and dry-season) burns in pinelands of Everglades National Park showed conflicting results (Snyder 1986). In one case the recovery of hardwood biomass 1 yr after the summer burn was less than the 1-yr recovery after the winter burn, but in the other case the winter burn had less recovery than the summer burn. In both cases, however, it was the fire with the higher temperatures at ground level that had less hardwood regrowth. Snyder (1999) simulated fire by cutting stems of five hardwood species near ground level during different seasons in south Florida. There was an inherent seasonality in the ability of shrubs to recover by resprouting and recovery was generally least after top removal from March to October. Presumably this response is weak enough that it can be overridden by variation in fire severity.

The paired burns in Everglades National Park also showed that the season of burning affected success of pine seedling establishment (Snyder 1986). Seedlings were most numerous if fire occurred during the wet season prior to seedfall rather than the dry season after seedfall. Burning before seedfall creates a favorable seedbed and fires soon after seedfall kill seeds and seedlings. Virtually all large, mature pine trees survived burning in wet or dry season. Smaller trees showed some mortality, especially those < 7 cm diameter breast height (dbh) that were burned in the dry season on the site that had not burned in more than six years.

Increasing fire frequency generally favors herbaceous over woody species (Wade et al. 1980). Relatively small differences in fire frequency have been implicated in controlling herbaceous species diversity elsewhere (Walker and Peet 1983, Tester 1989) and the same may be true in south Florida. Fire often stimulates flowering of herbs in fire-prone ecosystems (Whelan 1995) and several south Florida pineland species flower very little except after fire (Snyder et al. 1990). A study comparing the effects of wet-and dry-season burns on the reproductive biology of *Jacquemontia curtisii*, an endemic south Florida pineland herb, showed increased flowering but higher mortality of adult plants after wet-season burns and higher seedling establishment after dry-season burns (Spier and Snyder 1998).

2.3 Study Area

Pine forests make up about 43,500 ha, or 14.7% of the land area of Big Cypress National Preserve. These pinelands are considered transitional between the rockland pine forests of the Miami rock ridge, with predominantly tropical shrubs, and the more widespread pine flatwoods, with temperate species, characteristic of areas to the north (Abrahamson and Hartnett 1990, Snyder et al. 1990). The Miami rockland pine forests (and Lower Florida Keys pine rocklands) grow directly on the Pleistocene Miami oolitic limestone. The Big Cypress pinelands generally are growing in a shallow layer of sandy soil on top of Miocene Tamiami limestone. Duever et al. (1986) estimated an annual hydroperiod of 20-60 days, although this seems to be an underestimate based on our experience of the last few years. Given their low elevation and frequent inundation, most Big Cypress pinelands qualify as hydric pine flatwoods (Beever and Dryden 1993).

The Raccoon Point area in eastern Big Cypress National Preserve (Figure 3) is the largest remaining area of virgin south Florida slash pine forest, apparently having escaped the logging activities of 1900-1960 (Patterson and Robertson 1981). The pinelands consist of a mosaic of small, slightly elevated "islands" isolated among wetland areas of dwarf cypress prairies and cypress domes. This, along with their distance from sawmills, probably protected them from being logged. The inaccessibility of the area disappeared in 1977 when Exxon Company, USA, constructed an access road to drill for oil. Oil was discovered, and today an all-weather access road runs north from U.S. Highway 41 to the five large oil well pads in the Raccoon Point oil field.
The pinelands in the entire Raccoon Point study area were burned twice by the National Park Service as initializing burns before the start of this study (January 3-February 9, 1990 and February 27-April 4, 1994). The recent fire history of the area is included in the burn plan, Appendix 9.1.

The vegetation of Raccoon Point has been characterized by Gunderson and Loope (1982), who separated the pinelands into pine-*Sabal-Serenoa* and pine-hardwood types. The pine forests have sparse overstories, with pine densities of 100-600 trees/ha and tree heights generally <24 m.

In the pine-*Sabal-Serenoa* type, *Sabal palmetto* is the only other tree present, attaining subcanopy heights (up to 6 m), while *S. palmetto*, *Serenoa repens*, and *Myrica cerifera* are the principal shrubs. The herbaceous flora is dominated by the grasses *Schizachyrium rhizomatum* and *Muhlenbergia filipes*, the sedge *Rhynchospora divergens*, and the parasitic vine *Cassytha filiformis*. The pine-hardwood areas have a subcanopy that contains shrubs and trees such as

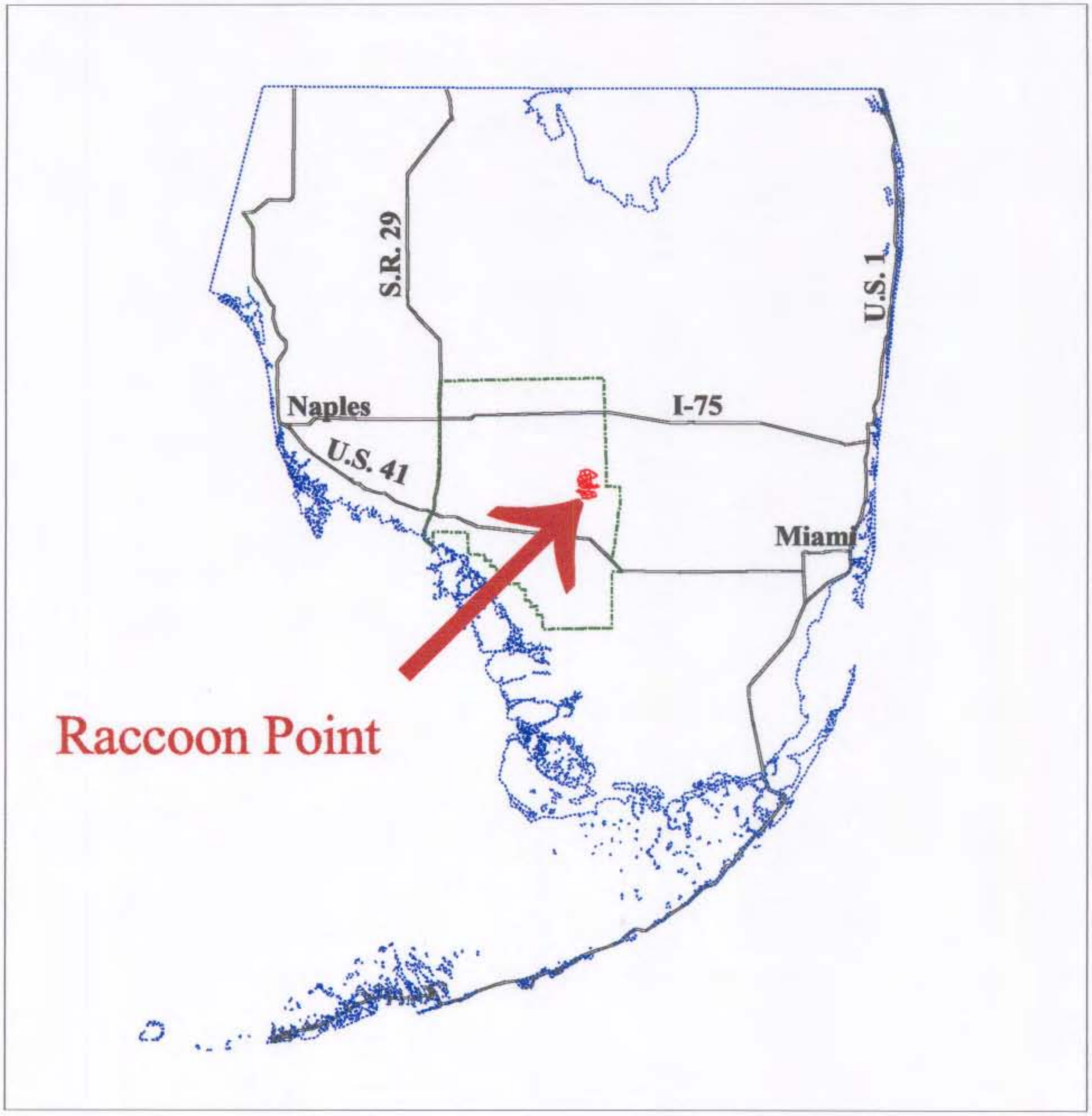

Big Cypress National Preserve
Major South Florida Roads

Location Map

Raccoon Point
Long-term Fire Ecology Project

30 0 30 60 90 120 Kilometers

N

BICY-GIS 10/98

Bumelia salicifolia, Chrysobalanus icaco, Ilex cassine, M. cerifera, Myrsine floridana, and *Persea borbonia*. The herb layer is dominated by hardwood seedlings, shrubs, and ferns, with far fewer graminoids.

Areas of stunted cypress prairie surround many of the pine islands and cypress domes. Gunderson and Loope (1982) found cypress density of about 1,000 trees/ha in cypress prairie, half that of nearby cypress domes that occupy the lowest elevations. Trees in the prairies are 2-7 m in height, while those in domes range from about 4 m at the edges to 20 m in the center. Cypress prairies have relatively sparse herb layers in spite of very open canopies and they share some species with pinelands. The herb cover in cypress domes is patchy and variable. Epiphytic bromeliads, especially *Tillandsia fasciculata* and *T. balbisiana* are quite common on the cypress trees.

The understory in the Raccoon Point pinelands contains at least eight plant taxa listed as endemic to South Florida by Avery and Loope (1980). There are no federally listed plant species, but a few are listed by the State of Florida as endangered (*Jacquemontia curtissii, Pteris bahamensis,* and *Tripsacum floridanum*) (Wood 1996). The nearby cypress domes and dwarf cypress forests contain several more state-listed species, mainly epiphytes.

The area provides important habitat for two endangered animal species--red-cockaded woodpecker and Florida panther--as well as the primary panther prey, white-tailed deer and feral hogs. Big Cypress National Preserve contains the southernmost remaining populations of the red-cockaded woodpecker, and six of the preserve's 42 known active woodpecker clusters occur within the Raccoon Point pinelands. Fire-maintained pinelands are critically important to the endangered red-cockaded woodpecker (Patterson and Robertson 1981). The species is closely adapted to open, mature pine forests of the Southeast and will abandon them when a substantial hardwood understory develops, even before the pineland has been completely replaced by hardwood forest.

3.0 OBJECTIVES

This long-term study will document the ecological effects of a wide range of potential fire management strategies on south Florida slash pinelands and associated cypress wetlands. The research will provide detailed data on vegetation responses (such as pine mortality and changes in species composition and dominance relationships) to different burning regimes. These results will be considered along with wildlife, public safety, and other management concerns in refining the prescribed burning program at Big Cypress National Preserve and elsewhere. The initial phase of the project will establish the baseline conditions and begin the experimental treatments. Specific objectives for the initial phase include:

1. Describe the vascular plant communities of all treatment units to document initial conditions and therefore provide a valid background against which to measure change.
2. Conduct all the initial experimental burning treatments by burning at three seasons.
3. Document the short-term effects (<1 yr) of season of burning and fire intensity on selected vegetation parameters.
4. Institutionalize burning and data collection schedules and protocols for the study of long-term effects of season and frequency of burning.

Figure 3 (opposite page). Location map, Raccoon Point long-term fire ecology project.

4.0 METHODS

4.1 Experimental Design

The basic design is a two-factor experiment combining three seasons and two frequencies of burning, resulting in six treatment combinations. The burning treatments are replicated three times, resulting in a total of 18 experimental burn units. Treatments were assigned in a completely randomized design.

The three seasonal treatments are defined as follows: 1) spring, or early wet season (May-June), 2) summer, or mid wet season (July-August), and 3) winter, or dry season (December 15- February 15). The early wet-season burn (actually at the transition from dry to wet season) is intended to represent the time when the largest areas burn by lightning-caused fire (Figure 1). The burns are carried out as soon as the first summer rains occur and before water levels rise appreciably. This season is expected to have the most severe burning conditions and the highest likelihood of fires entering cypress habitats. The mid wet-season burns occur after water levels have risen but before they reach their maximum late in the summer. Fuel conditions should be wettest during this treatment season. This season represents the period of less extensive lightning-caused fires. The dry-season burns are conducted after water levels have begun to recede and fuel conditions have dried appreciably. This season represents the period when the vegetation is most dormant as well as a period when substantial management burning has historically been done. The short-interval burn treatment is every 3 years and the long-interval treatment is every 6 years. Three years between fires is near the lower limit for pineland fires; it takes about 2 years for sufficient fuel to accumulate to consistently carry a fire. A fire interval much above 6 years results in high fuel loads and the potential for higher pine mortality and difficulty in control.

Two possible designs considered for carrying out the frequency treatments are shown in Table 3. The simplest design, in which all the replicates are burned in a single year (Table 3, A), was rejected because of the risk of unusual events masking treatment effects. For example, if a tropical depression brought large amounts of rain in early July of year 4, all the frequent early wet-season treatments would be flooded immediately after burning and all the hardwood shrubs could be killed. The decrease in hardwood stems seen upon sampling in year 8 in the frequent early wet-season plots (relative to the infrequent plots) could erroneously be attributed to frequency of burning. Other events that might cause this kind of problem are severe freezes, droughts, or hurricanes. By burning only one replicate per year this type of phenomenon is less likely to happen. Conversely, by burning one replicate per year (Table 3, B) there are burns every year, thus assuring that extreme abiotic events that influence ecological response will be documented. By spreading replicates over years there is also assurance that there will be burns soon before or after infrequent, yet important, biological events such as mast seeding. There is also practical value in spreading treatment burns over years: by having some burns every year there is a more constant level of effort and a greater likelihood that the experimental burns will become a routine part of the fire management program.

Table 3. Two possible designs for long-term fire study.

A. All replicates of each treatment burned the same year.

Year

	Replicate	1	2	3	4	5	6	7	8	9	0	1	2	3	4	5	6	7	8	9	0	1
Frequent	A	X			X			X			X			X			X			X		
	B	X			X			X			X			X			X			X		
	C	X			X			X			X			X			X			X		
Infrequent	A	X						X						X						X		
	B	X						X						X						X		
	C	X						X						X						X		

B. One replicate per treatment burned each year.

Year

	Replicate	1	2	3	4	5	6	7	8	9	0	1	2	3	4	5	6	7	8	9	0	1
Frequent	A	X			X			X			X			X			X			X		
	B		X			X			X			X			X			X			X	
	C			X			X			X			X			X			X			X
Infrequent	A	X						X						X						X		
	B		X						X						X						X	
	C			X						X						X						X

4.2 Prescribed Burning Treatments

The experimental treatments, prescribed fires, are conducted by the Big Cypress National Preserve Division of Fire and Aviation Management. The study and its experimental design have been incorporated into the preserve fire management plan (Big Cypress National Preserve 1994). A burn plan for the study with details of the burn prescription is included with this report as Appendix 9.1.

Water levels and rainfall are monitored before the burns at the permanent Raccoon Point hydrology station and the remote area weather station (RAWS) at Raccoon Point oil pad number 2.

The experimental units are burned by either ground or aerial ignition, depending on the circumstance. Often aerial ignition is followed by ground ignition of small, isolated areas of pineland. A concerted effort is made to burn all pineland fuels capable of carrying fire within the burn unit perimeter in each burn, and to ensure that all permanent plots burn. Fires are allowed to enter wetland areas but no special measures are taken to force fires through them.

Standard fire weather measurements are collected by the fire management staff. Data on fuel loads, fuel consumption, fire temperature, and other fire behavior measurements are collected by the research staff (see section 4.5).

4.3 Selection of Burn Units

The experimental units were delineated to contain 50 ha or more of pineland and at least one cypress dome. With units this large, edge effects are greatly reduced and the cost of burning per unit area remains reasonable. The burn unit boundaries were drawn so as to minimize the difficulty in containing the prescribed burns to individual units by utilizing roads, off-road vehicle (ORV) trails, and natural fire breaks such as cypress strands and domes. Special-use ORV trails will be designated around all the burn units to allow the use of swamp buggies and all-terrain vehicles for blackline construction. An effort was made to make the eighteen units as compact as possible while providing ready access from the all-weather roads around the oil well pads at Raccoon Point. Unit boundaries were initially drawn on 7.5 minute series USGS orthophoto maps at a scale of 1:24,000. The study area overlaps the Everglades 3 SW and North of Fiftymile Bend quadrangles. The unit boundaries were then transferred to April 1990 high altitude, infrared photos that had been enlarged to 1:10,080.

4.4 Permanent Vegetation Plots

Vegetation is sampled non-destructively in permanently marked plots. The initial sampling of the permanent plots provides the baseline conditions against which repeated measurements will be compared to detect changes brought about by the experimental treatments. The vegetation sampling methods are designed to allow detection of treatment effects in community composition (loss or gain of species) and, for each species, population size (density for woody plants, frequency for herbs), dominance (basal area and/or cover for woody species, cover for herbs), and size-class distribution (woody species only). Relatively little emphasis is placed on cover of herbaceous species because of the potential for seasonal or year-to-year variation to mask treatment effects.

Each burn unit is sampled by two types of permanent plots: 1.0 ha plots in which all trees ≥ 5 cm diameter breast height (dbh) are tagged and mapped (hereafter referred to as “tree plots”), and 0.1 ha plots in which the understory vegetation is sampled in addition to tagging and mapping of trees ≥ 2.5 cm dbh (hereafter “understory plots”). Each burn unit contains three tree plots, each with a nested understory plot, and two additional understory plots not inside tree plots.

Permanent plot markers are 60 or 90 cm pieces of 1.3 cm (0.5 inch) diameter steel reinforcement bar (rebar) that are driven 30-60 cm into the ground. Yellow plastic caps are placed on each piece of rebar for safety. In the frequent instances where there was limestone at or near the surface, a Bosch rotary hammer demolition drill with a 1.3 cm (0.5 inch) carbide drill bit was used to make holes for the rebar. This required a generator mounted on the back of an ATV and a 15 m (50 foot), 10 gauge extension cord. The Tamiami limestone is too hard to drive rebar into, and, in fact, is too hard for any of the portable, battery-operated drills we tested.

Plot locations were chosen by overlaying a grid on the enlarged aerial photograph and randomly choosing points that fell within what appeared to be pineland. If in the field it was determined that the area was not large enough to contain the plot or if more than 10% of the area was dominated by cypress, another point was chosen.

Plots were oriented in a north-south direction so that the permanent markers would be easier to locate in the future. In some cases the configuration of the pineland was such that the plot could not fit with a north-south orientation so they were put in as close to north-south as possible.

4.4.1 Tree Plots

To set up the 1.0 ha tree plots an east-west 100 m line was established by placing rebar at 0, 25, 75, and 100 m along a fiberglass tape. Two north-south 100 m lines were then established from the 25 and 75 m rebars using a double right angle prism to place 2 additional rebar stakes 50 m apart. The two remaining plot corners were put into place last. A total of 10 rebar stakes were placed in each plot (Figure 4). Round aluminum tags with unit and plot numbers were wired to the four corner stakes of the tree plot.

Each tree plot was divided into quarters in order to make tree mapping more efficient (Figure 4). The quarters were identified by compass orientation: NW, NE, SW, and SE. Each quarter was further subdivided into halves. A 50 m tape was laid out along the center line that separated these two halves. A right-angle prism was used to locate the position of each tree along the center line and a second tape was used to measure the distance from the center line to the tree. The field distances were entered into the computer and a dBase IV 1.5 program adjusted them to a single X,Y coordinate system.

Trees with a diameter at breast height (dbh) ≥ 5.0 cm and palms with a height to the apical bud ≥ 1.4 m were tagged with a round, 3.2 cm (1 1/4 inch) diameter, pre-numbered aluminum tag using a 5.4 cm (2 1/8 inch) aluminum nail. The tags were placed on live trees at breast height (approximately 1.4 m) and the diameter was measured just above the nail. Tags were placed in the most secure location on cabbage palms (preferably a smooth area without remnant leaf bases) and wherever it was possible to hammer a nail in the extremely hard pine snags (generally a hole or crack).

Data collected on individuals included tag number, mapping section, field X and Y coordinates, dbh, and species. For palms, height to the apical bud was recorded rather than dbh since palm diameters do not increase with age or size. Additional notes were taken on features such as basal fire scars on pine trees. Detailed field methods are contained in a laboratory notebook.

4.4.2 Understory Plots

One understory plot was nested in the center of each of the tree plots unless there was a low spot dominated by cypress trees or an ORV trail, in which case the plot was shifted to avoid these areas. Two additional understory plots were randomly located in pineland communities in each burn unit. The plots were 0.1 ha in size (20 x 50 m) and oriented north-south whenever possible.

A 50 m tape was laid out to establish the center line of the plots. A double right angle prism was used to position the four plot corners which are 10 m from the center line. A rebar was placed at the ends of the 50 m tape and at the four corners. A 50 m tape was then laid along both sides of the plot and rebar markers were placed at 5 m intervals. Sampling point number one was at the southwest corner with subsequent numbers along the line up to point 10. Sampling point number 11 was at the northeast corner and subsequent numbers move along the line in a southerly direction with 20 being the last point on the east line (Figure 5). Labeled aluminum tags were wired to each of the four corners in order to identify the unit and plot.

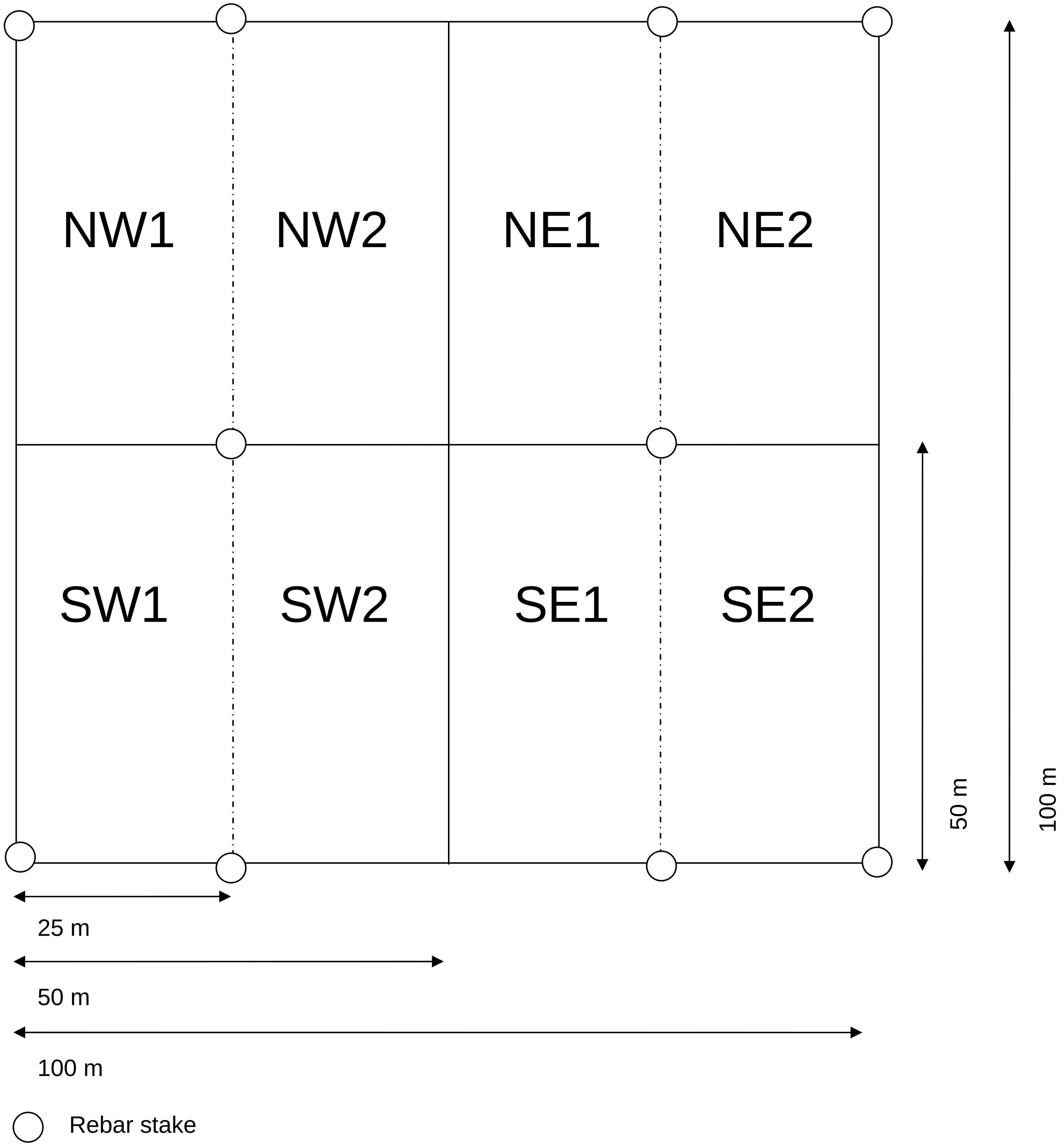

Figure 4. Tree plot layout.

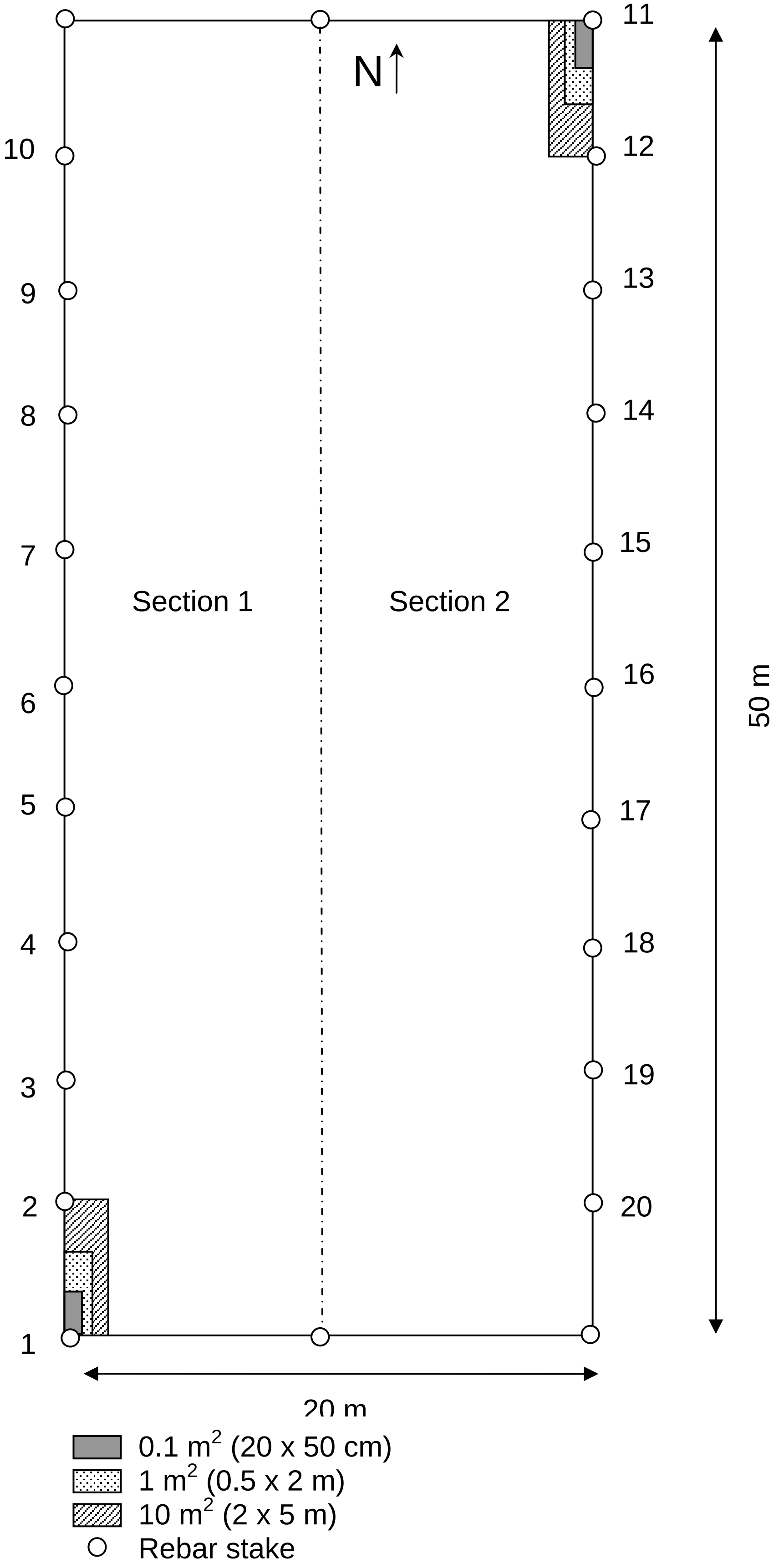

Figure 5. Understory plot layout. Nested quadrats shown only for points 1 and 11.

Live trees ≥ 2.5 cm dbh were tagged and mapped. A 50 m tape was laid out along the center line. A double right angle prism was used to map the trees to the west and east of this line. Tree plot data collection methods were applied to the vegetation plots.

The understory vegetation was sampled in a series of nested quadrats at each of the 20 sampling points (Figure 5). At each point there are three nested quadrats: 0.1 m^2 (20 x 50 cm), 1.0 m^2 (0.5 x 2.0 m), and 10 m^2 (2.0 x 5.0 m). Sampling frames are constructed out of 1.9 cm (3/4 inch) diameter pvc pipe. Some of the joints are not glued so the frame can be disassembled for ease of transporting and setting up.

Nylon twine is wrapped around and strung between each of the 11 pieces of rebar along each side of the vegetation plot. The 0.1 m^2 frame is hooked around the sample point (rebar) and the long side of the frame is lined up under the twine. All vascular plant species (herbaceous and woody) rooted inside the frame are listed using a four-letter species code and assigned a cover class (1 = <1%, 2 = 1-5%, 3 = 5-10%, 4 = 10-25%, 5 = 25-50%, 6 = 50-75%, and 7 = 75-100%). Total herbaceous cover is also assigned a cover class. The presence of exposed rock, logs, and tree roots is noted and a cover class is assigned to each. Tree and palm seedlings are tallied and their heights measured. The 1.0 m^2 frame is then put into place over the small frame and identical data are collected as for the 0.1 m^2 quadrat.

Shrubs, palms, woody vines, and trees are the only species listed for the 10 m^2 quadrat. The number and height of stems ≥ 50 cm tall is recorded. Cover classes are assigned as before to the species regardless of height. A total cover class is assigned to each sampling frame size. Heights are measured on all tree seedlings ≥ 20 cm tall and palm seedlings with leaf lengths ≥ 20 cm long in the 10 m^2 quadrat.

Details of vegetation sampling methods are maintained in laboratory files. Notebooks with line drawings and written descriptions of species encountered in the study plots have been put together and are available in the lab.

4.5 Fire Behavior

We measure the severity of the experimental burns by four methods: fuel consumption, fire temperature, and bark charring and needle scorching of pine trees. Fuel consumption is measured by sampling fuels before and after the burns. Fuel samples are collected outside the west and east understory plot boundaries. Prior to burning, a 0.5 x 1.0 m (0.5 m^2) pvc frame is placed 5 meters away from odd numbered vegetation sample points. All fuel in the frame and up to 2 m above ground level is clipped. Leaves and stems ≤ 6 mm (0.25 inch) diameter of woody species are collected and palm fronds are clipped at the petiole. All pine cones and dead fuel up to 2.5 cm (1 inch) in diameter are also collected. Collected fuel is put into plastic bags and sorted in the laboratory as soon as possible. The fuel is separated into several categories: live herbaceous, live palm leaves, live woody ≤ 0.6 cm (0.25 inch)in diameter, dead ≤ 0.6 cm (0.25 inch) in diameter, and dead > 0.6 cm and ≤ 2.5 cm in diameter. Fuel samples are dried to a constant weight at 70°C. Fuels are then sampled at the even-numbered points within a few days after burning and sorted in the field into the same categories as the preburn samples. The dry weights of these samples are subtracted from the preburn weights to calculate fuel consumption.

Relative fire temperature is measured with small steel plates on which a series of temperature-sensitive paints have been dabbed. Fifteen paints that melt at temperatures ranging from 66-454°C (150- 850° F in 50° F increments, Tempilaq, Big Three Industries) are placed on 7.5 cm x 7.5 cm x 3 mm steel plates.

The plates have a small hole drilled in one corner for hanging on a wire support. The lowest temperature paint is placed at the bottom of the plate and the highest temperature is placed in the upper right corner (Figure 6).

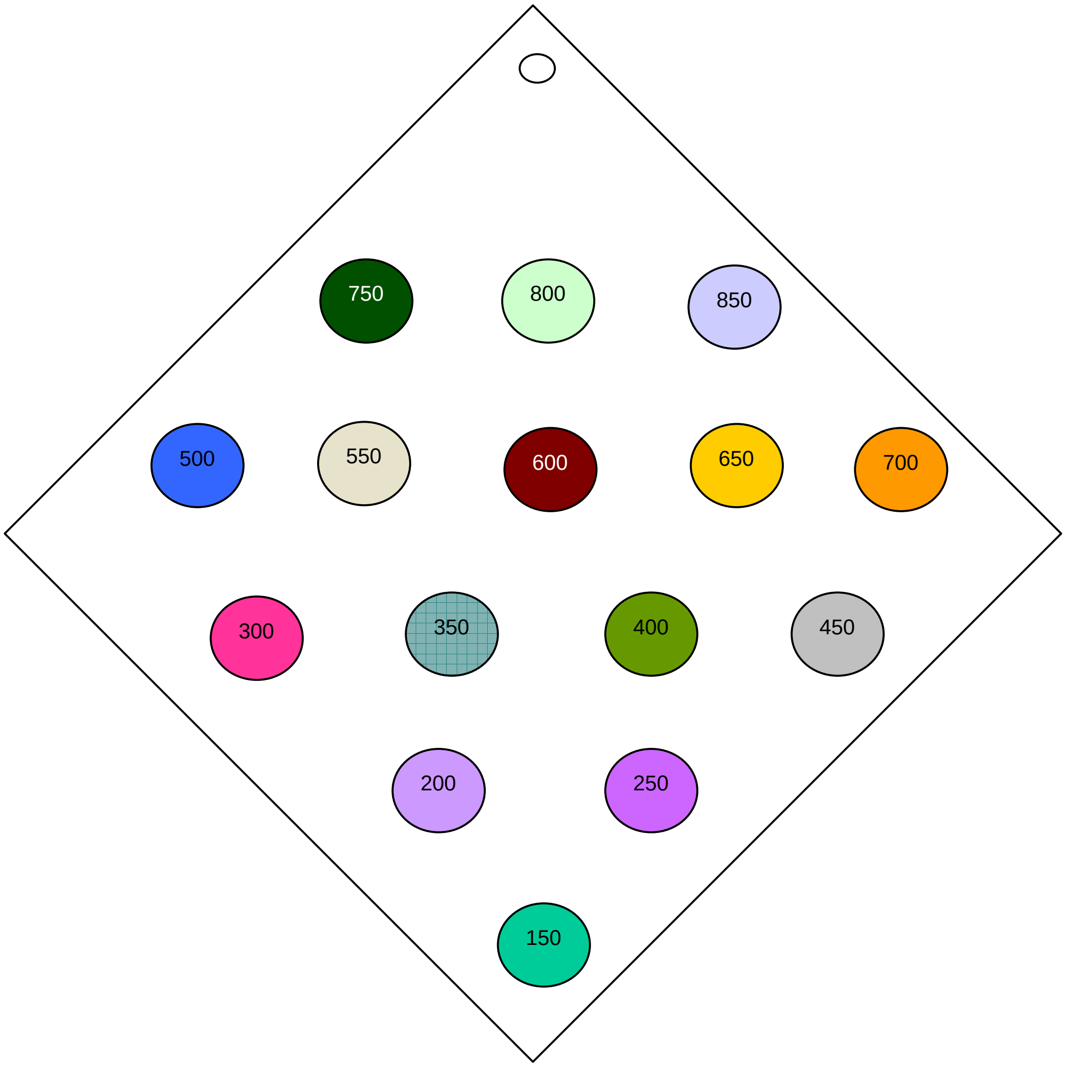

Figure 6. Temperature plate showing melting temperatures in degrees Fahrenheit.

A total of 148 plates are put out in each unit the morning of the burn. Four plates are put out at each corner of a tree plot, 5 m from the corner rebars in the four cardinal directions. There are 20 plates associated with each understory plot. A plate is placed 3 meters to the outside of the even numbered vegetation sample points. An additional 10 plates are located inside the vegetation plot. A 50 meter tape is stretched along the center line and the first plate is placed 3 meters to the west of the center line at the 0 meter mark (south end of plot). The next plate is placed 3 meters to the east of the center line at the 5 meter mark. The third plate is placed 3 meters to the west of the center line at the 10 meter mark. This layout continues north along the 50 meter tape. Temperature plates are collected and scored in the field as soon as the plots have finished burning. This is to insure that the plates are read before any damage can occur. The plates are also scored in the laboratory to confirm field readings and re-read ambiguous plates.

Within a few weeks of burning, all pine trees are evaluated for crown scorch and bark charring. The percentage of the needles in the tree canopy killed by the fire (they turn chestnut brown within a few days) are visually estimated. The maximum height and direction of bark charring are measured with three meter pole or visually estimated if > 3 m.

5.0 PROGRESS AND RESULTS

5.1 Burn Units

The 18 experimental units are shown in Figure 7. The cover of major vegetation types in each burn unit is given in Table 4. The smallest unit (#18) contains slightly less than 50 ha of pineland. The northernmost units (#1-3) each have more than 100 ha. Unit 17 is bisected by the 11-Mile Road. All the units have a mixture of pine and cypress cover, but only a few have hardwood hammock.

Table 4. Area (ha) of Raccoon Point burn units by vegetation class.

Unit	Pine forest	Cypress dome and strand	Cypress prairie	Scattered pine and cypress	Hardwood hammock	Total
1	107	36	22	4	0	169
2	119	43	26	27	0	215
3	142	30	59	0	3	236[1]
4	74	27	37	0	0	138
5	75	26	32	0	0	133
6	92	32	44	0	0	168
7	96	30	40	0	0	166
8	71	14	24	1	0	110
9	71	23	65	0	0	159
10	61	20	20	0	0	101
11	55	14	8	7	0	84
12	84	20	43	0	2	149
13	76	21	28	19	2	148[2]
14	70	17	33	1	0	121
15	83	49	14	0	2	148
16	59	26	50	0	1	136
17	53	13	21	15	7	110[3]
18	48	9	25	0	0	82

[1] Includes 1.5 ha open water.
[2] Includes 2.2 ha graminoid marsh.
[3] Includes 0.7 ha graminoid marsh.

Figure 7 (opposite page). Vegetation map showing burn unit boundaries.

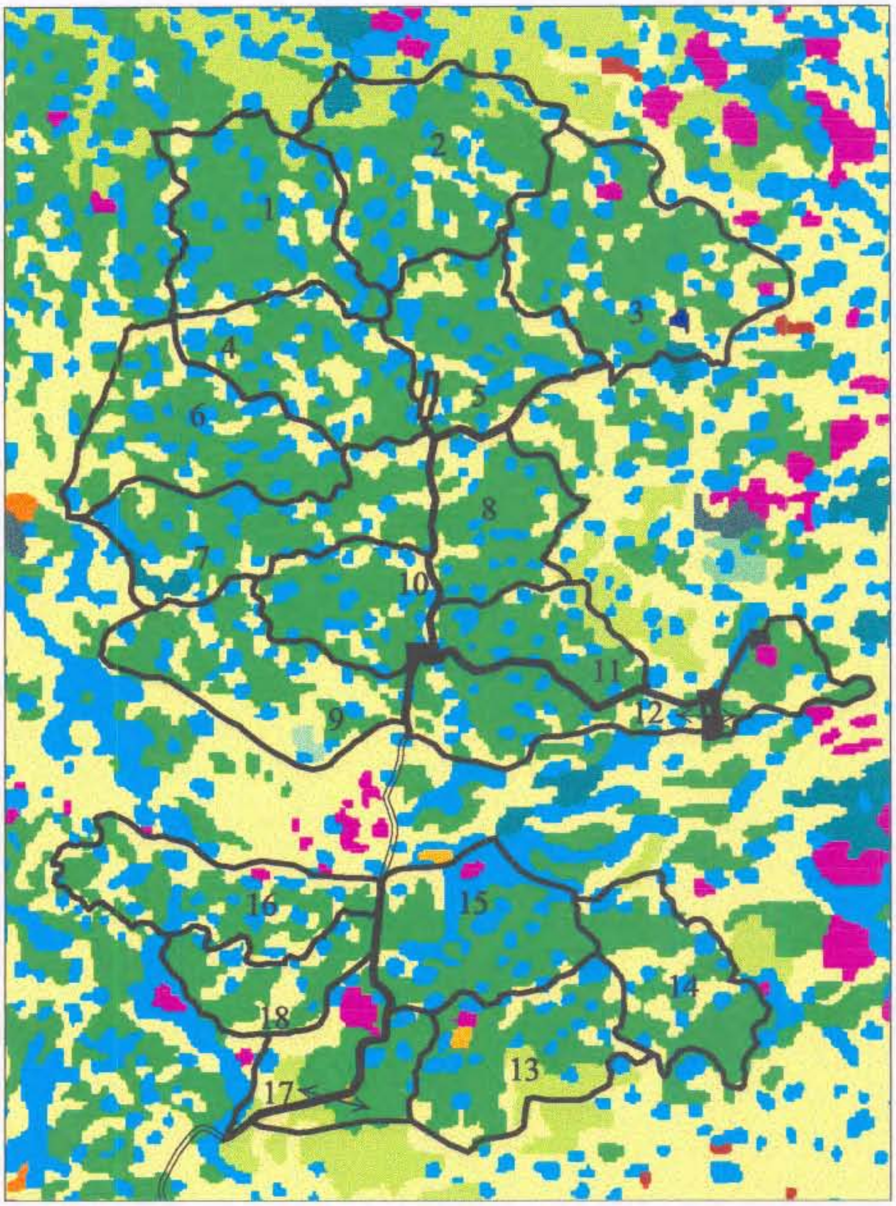

Mixed Hardwood-Cypress Strand
Graminoid Marsh
Scattered Pine and Cypress in Prairie
Cypress Prairie
Cypress Dome
Cypress Strand
Cypress Domes with Scattered Pine
Pine Forest with Cypress Associate
Low Density Pine Forest
Pine Forest
Pine Forest with Cypress Domes
Hardwood Hammock
Hardwood Scrub
Oil Drilling Pad
Water

Vegetation

Raccoon Point
Long-term Fire Ecology Project

1 0 1 2 3 4 Kilometers

N

BICY-GIS 10/98

5.2 Permanent Plots

The permanent vegetation plot locations are shown in Figure 8. The tree plots are represented by squares and the understory plots not in tree plots are marked by smaller rectangles. Some of the tree plot data are summarized in this report; no understory plot data included.

Table 5 shows the initial density of pine, cypress, and cabbage palms in the 54 permanent tree plots. The mean density of pines is 227 ha^{-1}, cypress 24 ha^{-1}, and cabbage palm 52 ha^{-1}. The hydric nature of the Raccoon Point pinelands is highlighted by the fact that only five of the plots did not have any cypress trees.

Additional data on pine and cypress are presented in Tables 6 and 7. The largest pines measured were just over 50 cm dbh, relatively large by south Florida standards. In the early 1980's the largest pines in the Long Pine Key area of Everglades National Park were 33 cm dbh (Snyder 1986). The second-growth, pre-Andrew stands in Everglades had much higher densities (458-1170 trees ha^{-1}) and basal areas up to 18.1 m^2 ha^{-1}. The stand characteristics of the Raccoon Point pinelands generally are close to the values for old-growth stands presented by Landers and Boyer (1999), although the number of snags is much greater than they report.

Maps of all the tree plots are included in Appendix 9.2. There is a wide range in the patterns of spatial distribution in the plots.

Graphs of the size-class distribution of the pine trees in the tree plots are included as Appendix 9.3. There is a wide range of size-class distributions in the Raccoon Point area. Doren et al. (1993) made much of the differences in size-class distribution among three areas of South Florida slash pine forest. They held that the size-class distribution in the Lostman's Pines area of Big Cypress National Preserve represented a model "unmanaged" and unlogged pine stand and that the other sites in Everglades National Park represented distributions resulting from logging or poor fire management. Unfortunately for their argument, all of these size-class distributions can be found in Raccoon Point which is unlogged and has experienced some form of fire management for a few decades.

5.3 Treatment Burns

The treatments assigned to the 18 experimental units are shown in Figure 9. Table 8 projects the burning schedule through 2007. All 18 units have received their initial treatment burns; dates of the burns are shown in Table 9.

Units 8 and 12 were to be burned in the spring of 1998, but severe wildfire conditions in northern Florida lead to a state-wide ban on burning that lasted until July. Conditions were not severe in south Florida, but it was politically impossible to get the burns done. The plan was to burn the plots in the spring of the following year to get them back on schedule. Unfortunately, a severe drought developed in South Florida in 1999 and it was too dry to burn in the spring. Summer rains began in July and broke the drought. We missed the window of opportunity, however, as a series of rainy days quickly lead to conditions becoming too wet to burn. Water levels rose and flooded most of the pinelands until November. The result was that in 1999 we were not only

---

Figure 8 (opposite page). Map of tree plot and vegetation plot locations.

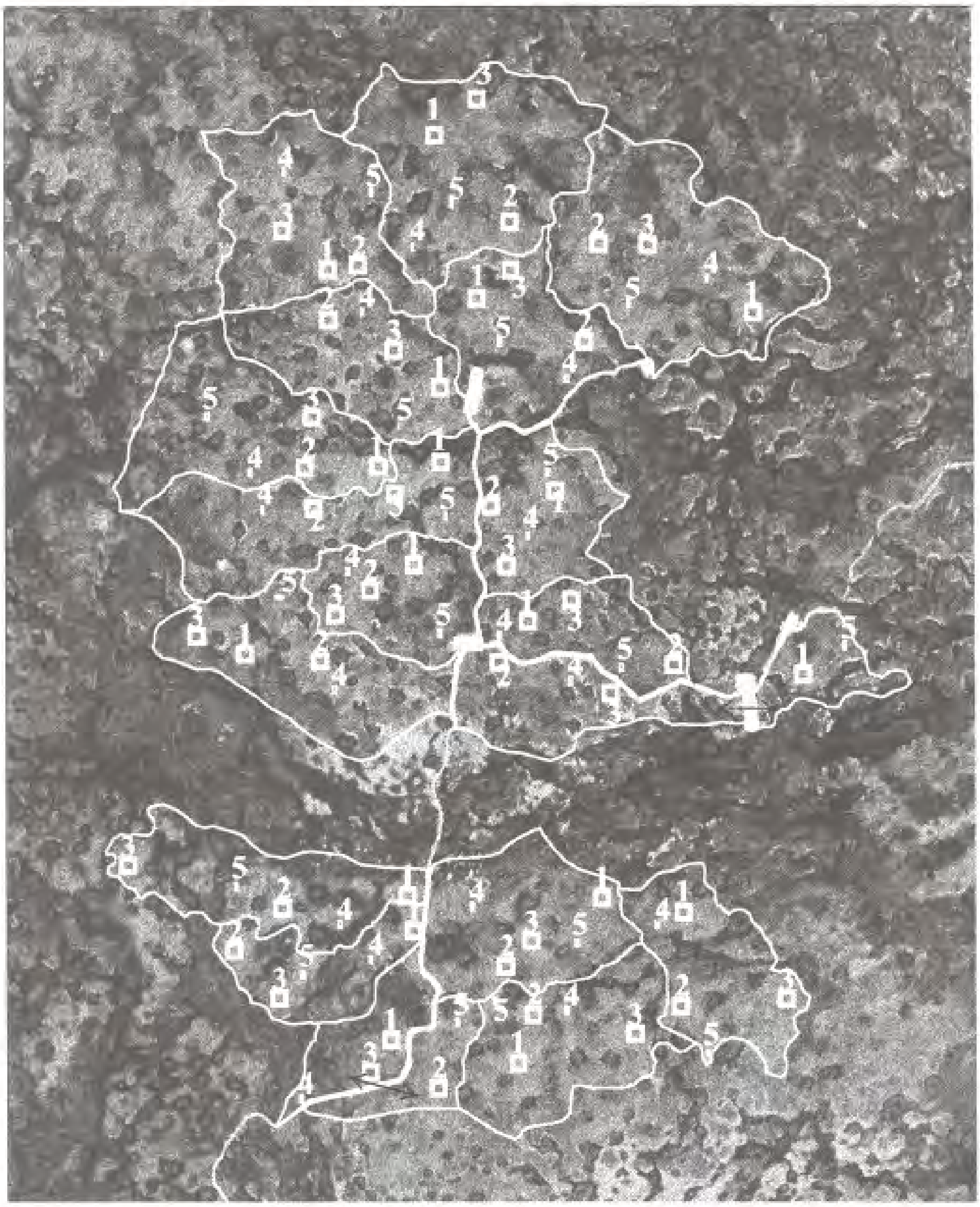

Tree and Vegetation Plots

Raccoon Point
Long-term Fire Ecology Project

1 0 1 2 3 4 Kilometers

N

BICY-GIS 10/98

Table 5. Number of trees per hectare by tree plot.

Unit	Plot	Pine	Cypress	Sabal	Snag
1	1	260	84	13	69
1	2	172	0	83	95
1	3	134	1	60	64
2	1	225	29	44	104
2	2	438	11	49	85
2	3	303	22	121	49
3	1	213	80	30	18
3	2	132	0	144	79
3	3	127	0	262	83
4	1	325	20	32	105
4	2	130	0	113	144
4	3	243	11	67	146
5	1	278	7	30	120
5	2	143	11	45	19
5	3	158	7	74	79
6	1	212	2	44	48
6	2	274	7	25	44
6	3	183	2	71	80
7	1	168	5	58	62
7	2	120	1	7	51
7	3	242	1	17	67
8	1	141	67	16	41
8	2	238	19	14	61
8	3	259	3	40	45
9	1	400	4	28	57
9	2	140	1	37	64
9	3	402	89	36	50
10	1	166	1	37	58
10	2	301	8	23	52
10	3	334	51	10	59
11	1	316	26	65	52
11	2	314	67	43	112
11	3	169	9	53	86
12	1	141	77	85	47
12	2	302	59	65	65
12	3	250	64	49	73
13	1	118	15	11	37
13	2	189	1	77	98
13	3	336	23	22	49
14	1	122	4	49	88
14	2	106	19	23	54
14	3	168	38	158	31
15	1	134	27	9	31
15	2	420	1	22	63
15	3	248	10	16	47
16	1	159	31	12	42
16	2	169	16	32	109
16	3	431	46	22	53
17	1	337	33	46	39
17	2	228	57	14	62
17	3	419	101	11	34
18	1	79	21	47	49
18	2	154	0	192	154
18	3	72	4	82	110

Table 6. Pine tree statistics by tree plot.

Unit	Plot	Density (Trees/ha)	Diameter (cm): Mean	Diameter (cm): Range	Basal Area (m^2/ha)
1	1	260	18.9	5.1 - 42.8	8.74
1	2	172	21.9	5.0 - 44.4	7.72
1	3	134	29.5	12.8 - 43.1	9.46
2	1	225	22.0	6.2 - 47.2	9.35
2	2	438	19.2	8.1 - 44.2	13.72
2	3	303	20.2	6.4 - 46.0	10.96
3	1	213	19.0	5.1 - 47.1	6.91
3	2	132	29.8	9.8 - 43.3	9.80
3	3	127	29.8	5.4 - 50.8	9.91
4	1	325	19.0	5.7 - 42.1	10.57
4	2	130	23.1	5.0 - 35.2	5.81
4	3	243	22.4	8.1 - 47.5	10.39
5	1	278	19.4	5.9 - 36.7	8.92
5	2	143	24.1	5.0 - 42.6	7.34
5	3	158	25.2	7.8 - 41.2	8.48
6	1	212	23.0	8.7 - 45.8	9.73
6	2	274	20.9	7.6 - 40.2	10.32
6	3	183	19.7	5.0 - 43.2	6.56
7	1	168	23.9	6.0 - 46.7	8.27
7	2	120	25.0	9.3 - 45.8	6.45
7	3	242	20.3	9.2 - 43.9	8.47
8	1	141	28.3	5.8 - 47.9	9.36
8	2	238	22.1	8.5 - 44.1	9.85
8	3	259	20.7	8.5 - 48.0	9.71
9	1	400	20.3	7.3 - 46.7	14.05
9	2	140	24.4	13.1 - 40.8	6.83
9	3	402	20.1	6.1 - 46.2	14.01
10	1	166	15.9	5.0 - 42.0	4.57
10	2	301	18.3	7.9 - 37.0	8.59
10	3	334	19.7	7.0 - 35.9	11.06
11	1	316	21.3	7.8 - 37.7	12.23
11	2	314	19.8	7.7 - 35.2	10.22
11	3	169	22.5	11.2 - 32.2	7.00
12	1	141	24.0	5.2 - 41.8	7.51
12	2	302	20.1	6.0 - 43.5	10.43
12	3	250	21.6	5.0 - 40.9	9.78
13	1	118	22.5	5.0 - 41.2	5.29
13	2	189	18.4	5.0 - 38.9	6.45
13	3	336	12.9	5.0 - 38.8	5.77
14	1	122	27.7	5.1 - 45.4	8.22
14	2	106	25.1	10.8 - 42.0	5.56
14	3	168	23.2	8.0 - 42.1	7.70
15	1	134	20.4	5.2 - 43.5	5.24
15	2	420	12.3	5.0 - 49.3	7.30
15	3	248	13.0	5.0 - 45.9	5.29
16	1	150	23.6	5.1 - 51.6	7.26
16	2	169	26.7	12.1 - 39.2	9.82
16	3	431	18.2	6.7 - 41.3	12.60
17	1	337	12.3	5.0 - 44.0	5.92
17	2	228	13.1	5.0 - 37.7	4.16
17	3	419	13.1	5.0 - 38.6	7.28
18	1	79	26.2	6.2 - 44.8	4.80
18	2	154	25.5	6.0 - 48.5	8.36
18	3	72	28.3	7.9 - 44.8	5.14

Table 7. Cypress tree statistics by tree plot.

Unit	Plot	Density (Trees/ha)	Diameter (cm): Mean	Diameter (cm): Range	Basal Area (m^2/ha)
1	1	84	11.9	5.4 - 24.9	1.06
1	2	0	---	---	---
1	3	1	18.0	---	0.03
2	1	29	13.4	6.8 - 21.9	0.45
2	2	11	11.9	5.0 - 28.7	0.17
2	3	22	12.5	6.1 - 20.6	0.29
3	1	80	9.1	5.0 - 23.9	0.60
3	2	0	---	---	---
3	3	0	---	---	---
4	1	20	12.8	5.0 - 20.2	0.29
4	2	0	---	---	---
4	3	11	9.3	5.4 - 20.1	0.09
5	1	7	14.6	9.2 - 22.5	0.13
5	2	11	10.6	5.8 - 15.3	0.11
5	3	7	19.1	11.7 - 33.3	0.23
6	1	2	6.1	5.3 - 6.9	0.01
6	2	7	16.3	5.3 - 23.8	0.17
6	3	2	6.5	5.0 - 7.9	0.01
7	1	5	10.1	5.6 - 17.5	0.05
7	2	1	11.9	---	0.01
7	3	1	7.3	---	0.004
8	1	67	9.7	5.2 - 19.1	0.55
8	2	19	7.1	5.0 - 11.6	0.08
8	3	3	11.7	6.5 - 19.8	0.04
9	1	4	6.8	6.1 - 8.3	0.01
9	2	1	15.1	---	0.02
9	3	89	12.8	5.0 - 39.0	1.39
10	1	1	11.0	---	0.01
10	2	8	13.6	6.0 - 19.6	0.13
10	3	51	15.0	5.2 - 28.6	1.01
11	1	26	12.6	5.9 - 23.3	0.38
11	2	67	12.6	6.2 - 22.5	0.92
11	3	9	10.2	5.3 - 22.1	0.09
12	1	77	10.1	5.0 - 27.0	0.76
12	2	59	11.8	5.2 - 24.9	0.74
12	3	64	11.4	5.4 - 25.0	0.75
13	1	15	6.6	5.0 - 9.3	0.05
13	2	1	5.6	---	0.002
13	3	23	7.1	5.0 - 10.7	0.09
14	1	4	14.3	11.3 - 16.8	0.07
14	2	19	10.0	5.0 - 19.1	0.19
14	3	38	11.8	5.0 - 25.1	0.48
15	1	27	10.8	5.1 - 23.9	0.31
15	2	1	32.4	---	0.08
15	3	10	9.5	6.8 - 15.0	0.08
16	1	31	10.0	5.2 - 20.0	0.28
16	2	16	12.5	5.0 - 27.1	0.24
16	3	46	13.7	5.6 - 31.1	0.81
17	1	33	8.3	5.1 - 18.6	0.21
17	2	57	7.8	5.0 - 17.1	0.31
17	3	101	9.2	5.0 - 22.6	0.80
18	1	21	19.8	5.7 - 43.2	0.81
18	2	0	---	---	---
18	3	4	13.8	10.4 - 21.4	0.07

Table 8. Raccoon Point burn schedule projected through 2007. S = short interval and L = long interval. O = burn completed, X = future burn, ( ) = unable to burn.

SEASON/FREQ	UNIT	96	97	98	99	00	01	02	03	04	05	06	07
Winter/S	6		O			O			X			X	
Winter/S	3			O			X			X			X
Winter/S	13				O			X			X		
Winter/L	17		O						X				
Winter/L	1			O						X			
Winter/L	2				O						X		
Spring/S	5	O			( )	O		X			X		
Spring/S	11		O			O			X			X	
Spring/S	8			( )	( )	O		X		X			X
Spring/L	9	O						X					
Spring/L	16		O						X				
Spring/L	12			( )	( )	O				X			
Summer/S	10	O			( )	O		X			X		
Summer/S	7		O			O			X			X	
Summer/S	14			O			X			X			X
Summer/L	18	O						X					
Summer/L	15		O						X				
Summer/L	4			O						X			

unable to burn units 8 and 12 which awaited their first burns, but we also could not perform the second burns of units 5 and 10, spring and summer burns, respectively. These units were all successfully burned in 2000.

6.0 PLANS

We will meet with the Big Cypress fire management staff to discuss the initial round of treatment burns to see if there are any problems or concerns that need attention.

We are back on schedule for all burns except for Unit 8, which was due to be burned again next year. Experience has shown that Raccoon Point pinelands will not burn well with less than two years of fuel accumulation, so we will re-burn Unit 8 in 2002 and then get back on schedule (Table 8). From the perspective of the long-term design we can still refer to the "3-year" treatment as short-interval and the "6-year" treatment as the long-interval fire frequency.

Table 9. Dates of completed burns. SPR = spring, SUM = summer, W = winter; S = short interval, L = long interval.

Unit	Treatment	1st Burn	2nd Burn	3rd Burn
5	(SPR/S)	06/01/96	3/30/00	
9	(SPR/L)	06/02/96		
10	(SUM/S)	07/31/96	07/19/00	
18	(SUM/L)	07/29/96		
6	(W/S)	02/12/97	02/16/00	
17	(W/L)	02/04/97		
11	(SPR/S)	05/26/97	05/04/00	
16	(SPR/L)	05/28/97		
7	(SUM/S)	08/19/97	07/19/00	
15	(SUM/L)	10/24/97		
1	(W/L)	03/13/98		
3	(W/S)	03/11/98		
4	(SUM/L)	08/13/98		
14	(SUM/S)	08/12/98		
2	(W/L)	02/25/99		
13	(W/S)	02/17/99		
8	(SPR/S)	05/04/00		
12	(SPR/L)	07/05/00		

A paper on pine tree mortality after different seasons of burning will be presented at the Fire Conference 2000, November 27-December 1, 2000 in San Diego. The paper will be published as part of the conference proceedings. Two additional papers on the initial vegetation of the Raccoon Point study will be completed in the next few months: one on the old-growth forest structure and one on the understory vegetation. Posters (and published abstracts) describing the study have been presented at the George Wright Society Biennial Conference, March 22-26, 1999, Asheville, North Carolina and the South Florida Restoration Science Forum, May 17-19, 1999, Boca Raton, Florida.

Over the next year there will be a marked decrease in some aspects of data collection since all the initial conditions have been measured and there are fewer burns scheduled. We will be collecting new types of data on flowering to relate reproductive activity to season of and time since burning. We will also be establishing permanent transects from pineland into cypress domes to document the elevation and vegetation gradient. We will continue to encourage outside investigators to initiate research that takes advantage of the experimental design.

Figure 9 (opposite page). Map of burn units showing treatments.

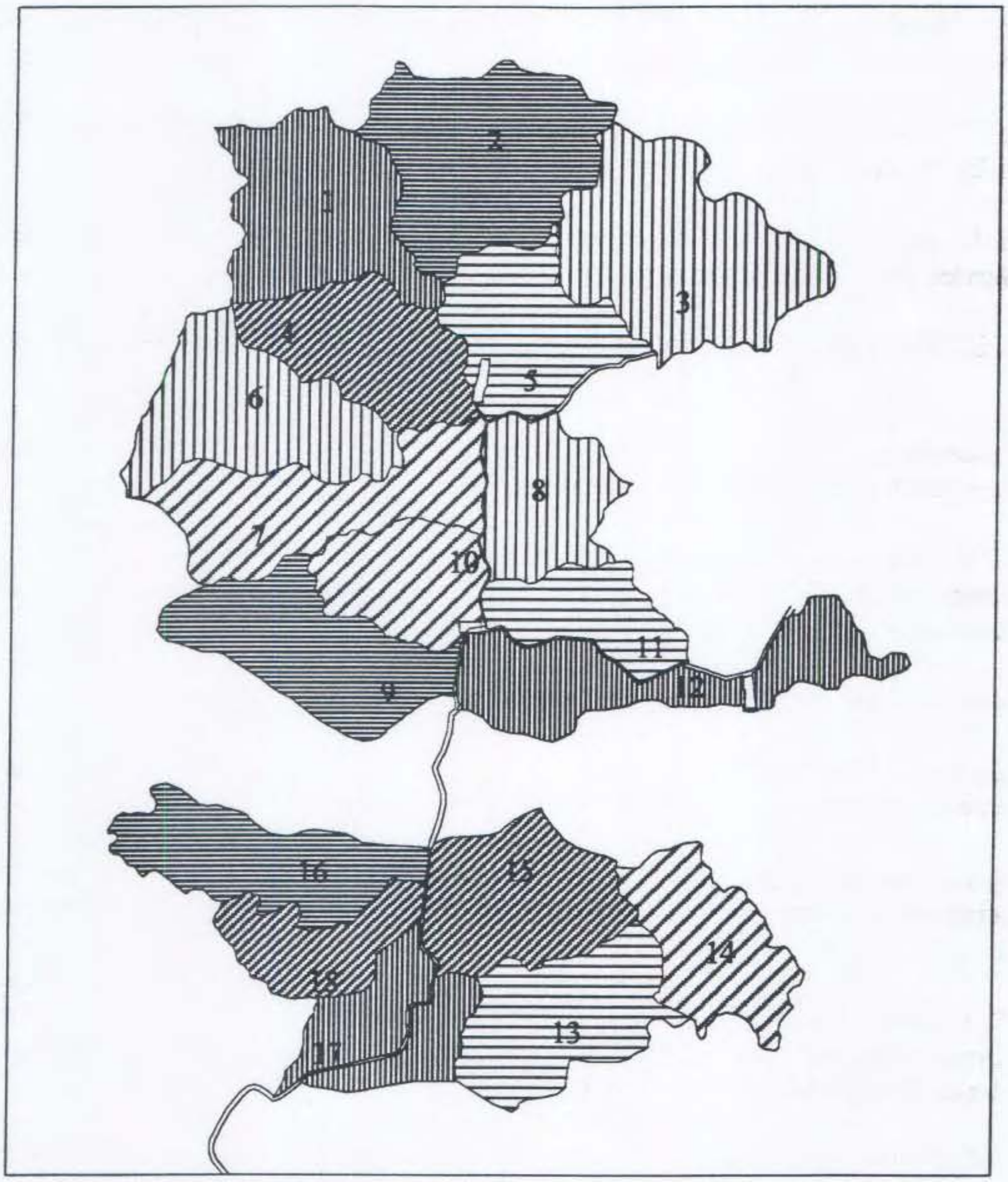

Burn Season and Interval

- spring, 3 yrs.
- spring, 6 yrs.
- summer, 3 yrs.
- summer, 6 yrs.
- winter, 3 yrs.
- winter, 6 yrs.

Raccoon Point
Long-term Fire Ecology Project

1 0 1 2 3 4 Kilometers

N

BICY-GIS 10/98

7.0 LITERATURE CITED

Abrahamson, W. G., and D. C. Hartnett. 1990. Pine flatwoods and dry prairies. Pages 103-149 *in* R. L. Myers and J. J. Ewel, editors. Ecosystems of Florida. University of Central Florida Press, Orlando, Florida.

Alexander, T. R. 1967. A tropical hammock on the Miami (Florida) limestone: a twenty-five-year study. Ecology 48:863-867.

Avery, G. N., and L. L. Loope. 1980. Endemic taxa in the flora of south Florida. National Park Service, South Florida Research Center Report T-558.

Bancroft, L. 1976. Fire management in Everglades National Park. Fire Management 37:18-21.

Bancroft, L. 1977. Natural fire in the Everglades. Pages 47-60 *in* Fire by Prescription Symposium proceedings, October 13-15 1976, Atlanta, Georgia, USA. U.S. Department of Agriculture, Forest Service, Washington, D.C.

Beever, J. W., and K. A. Dryden. 1993. The hydric pine flatwoods of southwest Florida: A community profile (draft). Unpublished report, Florida Game and Fresh Water Fish Commission, Punta Gorda, Florida.

Big Cypress National Preserve. 1994. Fire management plan. Ochopee, Florida.

Craighead, F. C. 1971. The trees of south Florida, Volume I, The natural environments and their succession. University of Miami Press, Coral Gables, Florida.

Doren, R. F., Platt, W. J., and L.D. Whiteaker. 1993. Density and size structure of slash pine stands in the everglades region of south Florida. Forest Ecology and Management 59:295-311.

Doren, R. F., and R. M. Rochefort. 1984. Summary of fires in Everglades National Park and Big Cypress National Preserve, 1981. National Park Service, South Florida Research Center Report SFRC-84/01.

Duever, M. J. 1984. Environmental factors controlling plant communities of the Big Cypress Swamp. Pages 127-137 *in* P. J. Gleason, editor. Environments of south Florida: present and past II. Miami Geological Society, Coral Gables, Florida.

Duever, M. J., J. E. Carlson, J. F. Meeder, L. C. Duever, L. H. Gunderson, L. A. Riopelle, T. R. Alexander, R. L. Myers, and D. P. Spangler. 1986. The Big Cypress National Preserve. The National Audubon Society Research Report No. 8.

Egler, F. E. 1952. Southeast saline Everglades vegetation, Florida, and its management. Vegetatio Acta Geobotanica 3:213-265.

Everglades National Park. 1991. Fire management plan and environmental assessment. Unpublished report, September 16, 1991.

Ewel, K. C. 1990. Swamps. Pages 281-323 *in* R. L. Myers and J. J. Ewel, editors. Ecosystems of Florida. University of Central Florida Press, Orlando, Florida.

Gunderson, L. H., and L. L. Loope. 1982. A survey and inventory of the plant communities in the Raccoon Point area, Big Cypress National Preserve. National Park Service, South Florida Research Center Report T-665.

Kalisz, P. J., A. W. Dorian, and E. L. Stone. 1986. Prehistoric land-use and the distribution of longleaf pine on the Ocala National Forest, Florida: an interdisciplinary synthesis. The Florida Anthropologist 39:183-193.

Kalisz, P. J., and E. L. Stone. 1984. The longleaf pine islands of the Ocala National Forest, Florida: a soil study. Ecology 65:1743-1754.

Ketcham, D. E., and J. E. Bethune. 1963. Fire resistance of south Florida slash pine. Journal of Forestry 61:529-530.

Klukas, R. W. 1973. Control burn activities in Everglades National Park. Proceedings of the Tall Timbers Fire Ecology Conference 12:397-425.

Komarek, E. V. 1964. The natural history of lightning. Proceedings of the Tall Timbers Fire Ecology Conference 3:139-183.

Landers, J. L., and W. D. Boyer. An old-growth definition for upland longleaf and south Florida slash pine forests, woodlands, and savannas. U.S. Department of Agriculture, Forest Service, Southern Research Station, General Technical Report SRS-29.

Langdon, O. G. 1963. Growth patterns of *Pinus elliottii* var. *densa*. Ecology 44:825-827.

Langdon, O. G. 1981. Some effects of prescribed fire on understory vegetation in loblolly pine stands. Pages 143-153 *in* Prescribed fire and wildlife in southern forests, Proceedings of a symposium, April 6-8 1981, Myrtle Beach, South Carolina, USA. Belle W. Baruch Forest Science Institute of Clemson University, Georgetown, South Carolina.

Lewis, C. E., and T. J. Harshbarger. 1976. Shrub and herbaceous vegetation after 20 years of prescribed burning in the South Carolina Coastal Plain. Journal of Range Management 29:13-18.

Likens, G. E., editor. 1989. Long-term studies in ecology, approaches and alternatives. Springer-Verlag New York, Inc., New York, New York.

Little, E. L., and K. W. Dorman. 1954. Slash pine (*Pinus elliottii*), its nomenclature and varieties. Journal of Forestry 50:918-923.

Loope, L. L., and V. L. Dunevitz. 1981. Impact of fire exclusion and invasion of *Schinus terebinthifolius* on limestone rockland pine forests of southeastern Florida. National Park Service, South Florida Research Center Report T-645.

Lotan, J. E., B. M. Kilgore, W. C. Fischer, and R. W. Mutch, technical coordinators. 1985. Proceedings--symposium and workshop on wilderness fire, November 15-18 1983, Missoula, Montana, USA. U.S. Department of Agriculture, Forest Service, Intermountain Forest and Range Experiment Station General Technical Report INT-182.

Lotti, T., R. A. Klawitter, and W. P. LeGrande. 1960. Prescribed burning for understory control in loblolly pine stands of the coastal plain. U.S. Department of Agriculture, Forest Service, Southeastern Forest Experiment Station Paper 116.

Myers, R. L., and P. A. Peroni. 1983. Approaches to determining aboriginal fire use and its impact on vegetation. Bulletin of the Ecological Society of America 64:217-218.

Patterson, G. A., and W. B. Robertson. 1981. Distribution and habitat of the red-cockaded woodpecker in Big Cypress National Preserve. National Park Service, South Florida Research Center Report T-613.

Robbins, L. E., and R. L. Myers. 1992. Seasonal effects of prescribed burning in Florida: a review. Tall Timbers Research, Inc., Miscellaneous Publication No. 8, Tallahassee, Florida.

Robertson, W. B. 1953. A survey of the effects of fire in Everglades National Park. National Park Service, Homestead, Florida.

Snyder, J. R. 1986. The impact of wet season and dry season prescribed fires on Miami Rock Ridge pineland, Everglades National Park. National Park Service, South Florida Research Center Report SFRC-86/06.

Snyder, J. R. 1991. Fire regimes in subtropical south Florida. Proceedings of the Tall Timbers Fire Ecology Conference 17:303-319.

Snyder, J. R. 1999. Seasonal variation in resprouting ability of native and exotic hardwoods in south Florida. Pages 257-269 *in* D. T. Jones and B. W. Gamble, editors. Florida's Garden of Good and Evil: Proceedings of the 1998 Joint Symposium of the Florida Exotic Pest Plant Council and the Florida Native Plant Society.

Snyder, J. R., A. Herndon, and W. B. Robertson. 1990. South Florida rockland. Pages 230-277 *in* R. L. Myers and J. J. Ewel, editors. Ecosystems of Florida. University of Central Florida Press, University Presses of Florida, Orlando, Florida.

Spier, L. P. and J. R. Snyder. 1998. Effects of wet- and dry-season fires on *Jacquemontia curtisii*, a south Florida pine forest endemic. Natural Areas Journal 18:350-357.

Strayer, D., J. S. , C. G. Jones, J. Kolasa, G. E. Likens, M. J. McDonnell, G. G. Parker, and S. T. A. Pickett. 1986. Long-term ecological studies: an illustrated account of their design, operation, and importance to ecology. Occasional Publication of The Institute of Ecosystem Studies Number 2. Institute of Ecosystem Studies, New York Botanical Garden, Mary Flagler Cary Arboretum, Millbrook, New York.

Streng, D. R., J. S. Glitzenstein, and W. J. Platt. 1993. Evaluating effects of season of burn in longleaf pine forests: a critical literature review and some results from an ongoing long-term study. Proceedings of the Tall Timbers Fire Ecology Conference 18:227-263.

Taylor, D. L. 1980a. Fire history and man-induced fire problems in subtropical south Florida. Pages 63-68 *in* Proceedings of the Fire History Workshop, October 20-24 1980, Tucson, Arizona, USA. U.S. Department of Agriculture, Forest Service, Rocky Mountain Forest and Range Experiment Station General Technical Report RM-81.

Taylor, D. L. 1980b. Summary of fires in Everglades National Park and Big Cypress National Preserve, 1979. National Park Service, South Florida Research Center Report T-595.

Taylor, D. L. 1981. Fire history and fire records for Everglades National Park 1948-1979. National Park Service, South Florida Research Center Report T-619.

Taylor, D. L. and Doren. 1982. Summary of fires in Everglades National Park and Big Cypress National Preserve, 1980. National Park Service, South Florida Research Center Report T-663.

Taylor, D. L., and R. Rochefort. 1981. Fire in the Big Cypress National Preserve, Florida. Fire Management Notes Spring:15-18.

Tester, J. R. 1989. Effects of fire frequency on oak savanna in east-central Minnesota. Bulletin of the Torrey Botanical Club 116:134-144.

Wade, D., J. Ewel, and R. Hofstetter. 1980. Fire in south Florida ecosystems. U.S. Department of Agriculture, Forest Service, General Technical Report SE-17.

Waldrop, T. A., D. L. White, and S. M. Jones. 1992. Fire regimes for pine-grassland communities in the southeastern United States. Forest Ecology and Management 47:195-210.

Walker, J., and R. K. Peet. 1983. Composition and species diversity of pine-wiregrass savannas of the Green Swamp, North Carolina. Vegetatio 55:163-179.

Whelan, R. J. 1995. The ecology of fire. Cambridge University Press, New York.

Williams, G. W. 2000. Introduction to aboriginal fire use in North America. Fire Management Today 60:8-12.

Wood, D. A. 1996. Florida’s endangered species, threatened species, and species of special concern: official lists. Florida Game and Fresh Water Fish Commission, Tallahassee, Florida.

8.0 ACKNOWLEDGEMENTS

A project this large requires the efforts and contributions of many individuals and organizations. Financial support has been provided by the USGS, Biological Resources Division, Florida Caribbean Science Center. Additional funding has been provided by the Natural Resource Preservation Program that shifted from the National Park Service to the National Biological Survey. Since 1997 additional funding has been provided by the Critical Ecosystems Studies Initiative of the Department of Interior's South Florida Ecosystem Restoration Program.

Dale Wade, US Forest Service, and the late Dr. Bill Robertson, Everglades National Park, reviewed the original proposal. Dr. Dale Taylor began a similar, but unreplicated, study of fire season and frequency in the early days of Big Cypress National Preserve that inspired this work.

The fire management staff of Big Cypress National Preserve has ably and professionally carried out all burning associated with the project. Chief Larry Belles, Kevin Walsh, Jack Finley, Dan Flaherty, Pilot Bill Evans, and numerous fire management staff members have burned what needed to be burned.

The hard work to get a unit burned only takes a day or two. The effort to get plots set up and sampled can take weeks—often under oppressively hot and humid conditions. Many individuals have assisted with the data collection aspect of the study. The following worked as USGS employees or contractors: Marlena Hovorka, Amy Borrelli, Steve Brodeur, Scott Cooper, Jim Dodson, Angela Foreman, Lance Koch, Robyn Koch, Elizabeth Mickler, Dana (Madsen) Schulze, and Debra Siembieda. The following assisted as members of the Americorps program: Lamonica Carter, Debbie Duvall, Angela Foreman, Stacey Halpen, Yolanda Lukaszewski, Sandra Turbes, and Daniel Wright.

Jim Burch produced the figures in Appendix 9.2, as well as innumerable other graphs while he was with the USGS.

Big Cypress fire management staff often assisted with fieldwork in addition to carrying out the burns. Big Cypress resource management was particularly helpful in providing a buggy when it was too wet to use ATV’s and in treating melaleuca found within the study boundary.

JoAnn McAbe provided invaluable assistance with data entry as a National Park Service VIP (Volunteer in the Parks). John Link, VIP, was our handyman in the shop.

Calumet Florida (and Exxon before them) has graciously allowed us to house a trailer and storage shed on pad 2. Their employees, especially Tommy Saxon, have helped out in emergencies and have kept an eye on things while we were away. Tommy knows how good a glass of sweet tea can be after a hot day in the field.

Keith Bradley and George Gann of the Institute for Regional Conservation helped put names on some of the plants during the early days of plot sampling. Alan Herndon spent many days bending over herb plots using his vast knowledge of South Florida plants to identify sterile herbs. In spite of their efforts, there were still some little green things we couldn't figure out.

Scott Cooper was invaluable in getting this report in final form.

9.0 APPENDICES

9.1 National Park Service Prescribed Burn Plan for Experimental Burns

I. AGENCY AND GEOGRAPHIC INFORMATION

BURN NAME: Raccoon Point FIRE NUMBER:

FMU: Interior Pinelands

LATITUDE: LONGITUDE:

LEGAL DESCRIPTION: T.51S, R.34E SEC. 3-36
T.52S, R.34E SEC. 1-5, 8-17

BURN UNIT SIZE (ACRES)		AREA TO BE TREATED (ACRES)
Total area	26,000	**3-6 research blocks per year of approx. 300 acres each. Additional acreage will be treated around the boundary of the unit to provide a buffer to fire spread.**

BURN COMPLEXITY: moderate

(see complexity rating worksheet)

DESCRIPTION OF BURN UNIT:

This burn will include portions of the pinelands in the area surrounding the Raccoon Pt. oil developments, north approximately 5 miles to the boundary of lands currently managed by the preserve, east to the Dade/Collier county line and south to the northern boundary of the Jetport. The western boundary of the burn plan runs through sections 6, 7, 18,19, 30, 31, 32 of T51s R34e and sections 5, 8, 17 of T52s R34e and adjoins the boundary of the Lost Dog burn plan.

The northern portion of the unit has a natural barrier of cypress and sparse prairie on the east and western edges. There are numerous cypress domes scattered throughout the burn area. The southern portion of the burn area is bordered by cypress on the east and west and to the south is dwarf cypress prairie with very sparse fuels.

II. BURN OBJECTIVES

	TF-1202 CODE		TF-1202 CODE
CULTURAL SCENE MAINTENANCE:		HAZARD REDUCTION:	12
NATURAL SYSTEMS MAINTENANCE:		OTHER: 21,11 Fuel reduction/Habitat improvement	

NARRATIVE:

The objective of this **multi-year** burn is to assist research in the assessment of the effects of fire, during various seasons, on vegetation. Designated portions of the study area will be treated with fire at intervals specified by research and at various times of the year.

There are several Real properties and the Calumet Oil operations, within the burn area, which are at risk from wildfires and are a potential source of ignitions which may spread to the rest of the unit. In addition to treating the research plots these burns will reduce fuel accumulations which will limit intensities of wildfires and the probability of ignition. This will minimize tree mortality from wildfires and risk to the above mentioned properties.

Burning activities will be focused on small burn blocks designated by research for treatment during specified months of the year. Each year there will be two spring burns (May-June), two summer burns (July-Aug), and two winter burns (Jan). Each of these burn blocks is approximately 300 acres but the area actually burned will probably be larger to take advantage of trails and natural barriers for holding. The need to burn during specific months may require us to burn at the hotter end of our prescription limits.

APPROXIMATE FUEL LOADINGS BY FUEL TYPE:

	1 hr.	10 hr.	100 hr.
Model 7	1 ton acre	2 ton acre	1.5 ton acre
Model 3	3 ton acre		

III. BURNING PRESCRIPTION

TIME OF YEAR (MONTHS) Any time of year may be appropriate for portions of the burn unit (see matrix). Any special timing constraints, such as T & E species, will be addressed in the section on Sensitive Issues and Constraints (Other Constraints).

PARAMETER	HOT END OF PRESCRIPTION	COOL END OF PRESCRIPTION
Drought Index	625	0
Temperature	95° F	30
Midflame Wind Speed	12 mph	2 mph
Relative Humidity	35%	75%
Night Time Relative Humidity	85% minimum	100%
Water Levels	Not used currently	Not used currently
Soil Moisture	Not used currently	Not used currently
Smoke Dispersion: Day Burn	Average (41-60) or better *	
Smoke Dispersion: Night Burn	Above Average (7-12) or better *	

* small areas, such as securing around individual camps or remote small acreages (500 or less), may be burned at the next lower Dispersion level and with lower transport wind speeds if in the professional opinion of the Burn Boss this presents no smoke hazard to any sensitive targets.

WIND DIRECTION: Any wind direction may be appropriate for a portion of this burn. Directions will not be selected which will impact sensitive areas with smoke.

	FUEL MODEL 3		FUEL MODEL 7	
PARAMETER	HOT BURN	COOL BURN	HOT BURN	COOL BURN
1 Hr Fuel Moisture	8	22	8	22
10 Hr Fuel Moist.			11	23
100 Hr Fuel Moist.			12	24
Live Fuel Moisture			80	250
Rate of Spread - **Flanking fire**	10	3	3	1
Flame Length	4.2	1.7	2.0	1.1
Fireline Intensity BTU/Ft/Sec	126	17	26	6
Heat Per Unit Area BTU/Ft	689	332	497	387
Probability of Ignition (90°, 50% shading, 1 Hr Fuel Moisture 10%)	30%		30%	

OTHER PARAMETERS: Mixing height of 1700 feet or greater. (see note above)
Transport winds of 10 mph or greater (see note above)

Narrative: The intent of the prescription is to allow reduction of fuels while not creating an unacceptable level of tree scorch and mortality, and not proceeding with a burn which may become uncontrollable. Models noted above do not precisely describe South Florida fire behavior but do serve as a general guide to be balanced with local knowledge.

IV. WEATHER NARRATIVE:

In order to accomplish the rapid reduction of fuels with a minimum of scorch, midflame winds of 2 to 12 miles per hour are desired. Because of the small block size any wind direction may be acceptable for burning in this area. Any limit on wind direction would likely be based on holding requirements rather than smoke concerns. No burning will take place under "red flag" conditions or with the predicted likelihood of frontal passage wind shifts during the burn period.

A spot weather forecast will be requested prior to ignition of the unit. Recognizing the difficulties in forecasting in S. Florida and past experience in this area, monitoring on site will probably be the most reliable source of burn weather information.

FIRE BEHAVIOR NARRATIVE:

A moderate intensity fire is desired. It is anticipated in localized areas of palmetto and pine there will be some instances of higher intensity fire resulting in increased pine scorch, this is typical fire behavior in these areas when they have not been burned for an extended time period(more than 3-5 years). The local species of pine is highly adapted to this effect and will generally survive quite well even though apparently near 100% scorched. As the project progresses some blocks will not be burned for a number of years and on these we expect more active fire behavior with additional complexity for holding and firing crews.

Research has requested that we use flanking fire as consistently as possible as the primary ignition pattern for all of these burn blocks. Shifting winds occasionally modify flanking fires temporarily into head fires with some increase in impact to vegetation. This effect is usually short lived and will be monitored by the burn boss who may shift firing patterns if needed to minimize this occurrence. The intent of the firing pattern is to reduce the fuels in the research plots rapidly while allowing good control by holding forces.

V. FIRE HISTORY:

FIRE NUMBER:	DATES:	ACRES:	RESULTS:
88-010	Jan 1988	280	HUMAN
89-034	MAR 1989	143	HUMAN
89-036	MAR 1989	14	HUMAN
89-063	MAY 1989	24	HUMAN
89-090	AUG 1989	1	LIGHTNING
89-135	NOV 1989	7	HUMAN
90-003	JAN 1990	6695	PRESCRIBED
90-010	FEB 1990	1	HUMAN
90-024	FEB 1990	27	HUMAN
90-034	MAR 1990	59	HUMAN
90-037	MAR 1990	35	HUMAN
90-038	MAR 1990	1596	HUMAN
90-041	MAR 1990	13	HUMAN
90-065	JUN 1990	16	LIGHTNING
90-088	SEP 1990	1	HUMAN
90-104	NOV 1990	5	HUMAN
90-105	NOV 1990	60	HUMAN
91-013	FEB 1991	1	HUMAN
91-014	FEB 1991	17	HUMAN
91-063	OCT 1991	1	HUMAN
92-022	MAR 1992	3	HUMAN
92-023	MAR 1992	21	HUMAN
92-065	OCT 1992	10	PRESCRIBED
92-067	NOV 1992	7	HUMAN
93-005	JAN 1993	20	PRESCRIBED
93-018	MAR 1993	7	HUMAN
93-019	MAR 1993	195	HUMAN
93-056	OCT 1993	.5	HUMAN
93-060	OCT 1993	.1	LIGHTNING
93-061	OCT 1993	.1	LIGHTNING
94-012	APR 1994	6719.5	PRESCRIBED

1996 through 2000 Several units have been treated, each year, using prescribed fire as part of the research project.

NARRATIVE: This area and surrounding areas have a long history of human caused fires. Most are believed to have been started by hunters and camp owners burning around their property. Most of these have been in fall and early winter months and have been low intensity. The majority of the research area was burned in 1989-90 and again in 1994 as part of a prescribed burn.

VI. SENSITIVE ISSUES AND CONSTRAINTS

SMOKE MANAGEMENT:

This burn is planned for areas without smoke sensitive targets. The small size of the burn blocks should generate a minimum of smoke. Dilution and avoidance strategies will be used to mitigate whatever smoke problems may exist. The Burn Boss will monitor smoke drift from the ground or the helicopter to insure no problem areas exist. A minimum mixing height of 1700 feet will increase smoke dispersion. A dispersion adjective of 41 or higher is prescribed to minimize daytime smoke problems and an adjective of 7 or higher for night burning. Night time burning will only take place when the above conditions are met and if the Division of Forestry will issue burn permits. Because of the remote location and the distance to smoke sensitive targets small areas of less than 300 acres may be burned with a dispersion of 30 or higher.

OTHER CONSTRAINTS: (T&E SPECIES, STRUCTURES, CULTURAL RESOURCES, SIGNS, TRAILS, BACK COUNTRY USE)

This burn is within the range of the Florida Panther, burn activities should have no negative impacts on this endangered species. Improved forage created by the burn for Panther food species should provide a net benefit for any Panthers using this area. The Preserves Wildlife Biologist will be contacted prior to burning to assure no collared panthers or other species of special management interest are within the days planned burning boundaries or that appropriate mitigation takes place before the burn. Five known Red-cockaded Woodpecker colonies are within the burn unit. Backing and flanking fire will be used in these areas, along with any necessary mechanical fuel reduction, to insure colony trees are not mortally damaged by burn activities. Resources staff will be contacted as to anticipated needs at individual sites and will be invited to participate in burn implementation in these areas.

The Vegetation Specialist will be contacted to determine if melaleuca is within the burn area and what mitigation may need to be accomplished prior to burning.

Fire activities along the buggy trails will for brief periods inconvenience backcountry users, however, the extensive network of trails in this area should make this impact minimal. Fire personnel on the burn will contact visitors in the area to insure their safety while traveling near activity areas.

Oil flow lines are a concern for both Calumet and the fire program. These lines will be protected when we burn in those blocks which contain flowlines.

SAFETY: (PUBLIC, BURN PERSONNEL AND AVIATION)

Visitors will be excluded from the interior of the burn during the ignition phase and any camp occupants in residence within or directly adjacent to the burn will be contacted by the fire crew prior to ignition.

All NPS personnel within the burn unit will wear proper fire protective clothing and will carry fire shelters during the ignition and holding phases of the burn. A safety briefing will be part of the prefire briefing, and firing and holding forces will constantly monitor for potentially hazardous situations.

The most qualified medically trained (EMT, First aid, etc) person on the burn will serve as the lead in Emergency Medical Situations and will contact dispatch directly for additional support not available on the burn should need arise. The helicopter will shift priority to emergency evacuation if deemed necessary by the personnel on site. Ground evacuation will be accomplished by BICY vehicle or ambulance as appropriate.

The Holding boss will insure that all burn personnel know the location of safety zones and escape routes for the particular block being burned.

If any helicopter landing on the oil pads is anticipated as part of the normal burning activities, Calumet personnel will be notified to insure no safety conflicts exist with their ongoing operations.

Heat, smoke, poisonous snakes and vegetation, caprock and solution holes, and thick vegetation are recognized as known safety concerns on all our prescribed fires._ All burn personnel will keep these concerns in mind as they complete their assigned task. Everyone is responsible for safety on the burn!

Aerial ignition requires low level flight operations which involve recognized risks. A special use plan will be prepared annually to evaluate risks and benefits and alternatives. All required personal protective clothing and equipment will be used during flight operations as required by the Special Use Plan, the Interagency Helicopter Operation Guide and NPS policy. Personnel involved in aerial ignition will be entitled to Hazardous duty pay for the day (excluding pilots).

A member of the permanent fire staff will be designated as the Safety Officer for each period of burning and will focus their attention on potential hazards to the crew and the public and on mitigating those risks.

VII. EQUIPMENT AND SUPPLIES:

ITEM:	#	BURN PHASE-PREP,IGNITION,MOP-UP,MONITOR
PREMO MK III	1	Ignition Phase as needed
HELICOPTER	1	As needed
DRIP TORCHES	2-4	Ignition Phase

***See holding matrix for specific holding equipment needs by burn block.

BURN ORGANIZATION

BURN BOSS - Type 1 or 2 Burn Boss, if Type 2 must have burned previously in this burn unit as part of the management team (ie. Burn boss, Ignition spec., Holding boss)

IGNITION SPEC. - Position will likely be covered by the burn boss.

HOLDING SPEC. - Level of needed qualification will vary by number of resources assigned and time of year.

PLDO - A qualified Plastic Sphere Dispenser Operator will be assigned when aerial ignition is used.

SAFETY OFFICER - see safety section

Other positions will be filled on the day of the burn based on need and available personnel. Holding will be designated a early as possible to allow that individual to review the plan, recon the burn area, assess needs and participate in briefing the crew. Personnel on lieu days may be brought on duty to insure adequate forces for completion of the project. A burn team may vary from a few personnel to ten or twelve dependent on season and holding needs. Some overtime is likely for completion of the burn, patrol and equipment refitting.

VIII. PREBURN PREPARATION:

Burn block will be evaluated by the Burn Boss and the Holding Specialist prior to conducting the burn.

All needed fire equipment will be in fire ready condition.

The Burn team will be briefed by the Burn boss and Holding boss prior to the commencement of the burn.

No line construction (other than buggy line) is anticipated prior to the burn as buggy trails and natural features are being used for much of the burn block boundaries. A minimum of impact on the research plots by vehicles or foot traffic is essential to insure good data from the research project.

Mechanical clearance around red cockaded woodpecker colony trees will be completed prior to burning, where needed.

Treatment of any melaleuca within burn blocks will be completed prior to burning. Any areas that can not be treated will be excluded from the burn.

IX. IGNITION PLAN:

Ignition will be accomplished with a combination of hand and aerial methods. Aerial ignition will be used to ignite the interior portions of the burn blocks and along block boundaries where natural barriers and fuels allow. Hand ignition will be used where needed along firelines, around camps, oil developments and along buggy trails. Flanking fire will be used for the majority of firing on the unit, backing fire may be used in securing lines or in sensitive habitat (RCW). Exact firing patterns will be determined on site based on wind directions, relative positions of structures and improvements, vegetative patterns and fire behavior of test burns**. A test burn will be conducted on each burn day to evaluate on-site fire conditions and validate prescription and weather information**. *Pending a successful test burn a typical firing plan would start by securing the downwind lines with backing fire lit adjacent to the line. Ignition would continue from this downwind line up the flanks to secure these lines, then the interior would be lit using aerial ignition from the downwind line to the upwind line directly into the wind. Flanking fire from this ignition and from the ignition along the flank fire lines would then burn out the interior fuels. Any additional firing needed to secure the upwind lines would be accomplished when the bulk of the interior fuels had been treated in order to avoid putting too much headfire into the block.* Actual daily firing patterns will be documented in the unit log along with personnel assigned and other significant events. As areas of the burn are ignited, the Burn boss will monitor effects and adjust firing to meet burn objectives. Ignition will start after the dew has dried off leaves and grass and morning humidities have come within prescription parameters.

We will likely burn with a range of wind and humidity variables. Ignition patterns and methods will be adjusted to compensate for these anticipated changes.

The Burn boss will be in control of the firing either from the helicopter or on the ground in visual and radio contact with the ship or firing teams.

The Helicopter will be beneficial to allow rapid burning of the unit and to provide an aerial platform for observation. It is, however, not essential for accomplishing these burns and the absence of the helicopter would not rule out burning under the appropriate conditions.

X. HOLDING AND CONTINGENCY PLAN:

**** SEE ALSO HOLDING PLAN MATRIX; ATTACHED ****

Much of the area around the burn blocks is comprised of natural barriers or buggy trails and will require limited or no holding forces. Potential for escape during most of the year is very low. The natural barriers and trails plus the mosaic of burned areas from other prescribed fire activities combined with the remoteness of this burn area make the likelihood of impact from an escape to other than an isolated camp extremely low.   Buggy lines and/or hose lays will be placed as necessary to facilitate protection of properties and to contain the fire within the designated perimeter. A minimum of impact on the research plots by vehicles or foot traffic is essential to insure good data from the research project.   Cypress domes will provide a water source for many of the burn blocks at certain times of the year. A bouywall tank may be necessary to provide an adequate water source for holding on some blocks or during drier periods of time. Holding, in cooperation with the burn boss, will assess water, equipment, and personnel needs for each block prior to each days ignition and will implement needed actions to insure perimeter security. The Holding boss will be present with the buggy or hand crews as firing commences to insure adequacy of protection measures. The holding crew will insure fire does not spread outside the designated fire perimeter, that the public stays outside the burn area and that safety hazards (to public and employees) from smoke or fire activities are recognized and mitigated. In the event of short range spotting (not unlikely in these fuels) the holding boss will designate personnel to attack the spot(s) and will insure needed forces remain on the line to contain the original burn. The burn boss will be apprized of the situation (if not already aware) so that ignition may be halted or altered as needed during attack on these spot fires. Burning may continue if holding and the burn boss agree that it is safe and prudent to do so.

In the event of a slop over which cannot be contained by forces on hand within 30 minutes, the Burn Boss will declare the fire escaped. Should the fire escape control the Burn Boss (unless/until someone else is designated by the Wildfire Specialist or the FMO) will become the wildfire Incident Commander. All firing will cease except as used for burnout to establish fire lines or as necessary to prevent fire already ignited within the burn unit from threatening other burn block boundaries. Holding personnel and those Firing personnel, not essential to maintain the original burn perimeter, will become the initial attack force. Additional forces will be requested, if necessary, from Preserve fire qualified personnel, the USFWS or from EVER. A wildland fire situation analysis will be completed if the fire is not contained in the first burn period. Buggies and engines will be staffed and used as needed based on the current fire situation.

Typically we will have all needed Contingency Resources on site as part of the burn team.   During the driest period burns the Burn boss, Wildfire Specialist, Prescribed Fire Specialist and the FMO will assess additional contingency needs. Additional forces will be requested, if necessary, from Preserve fire qualified personnel, the USFWS or from EVER (availability and commitment will be confirmed prior to ignition). Buggies and engines will be staffed and used as needed based on the current fire situation.

Burning of each unit will be accomplished in one day to meet the requirements of the research project. All of the units are planned for burning during the daytime to allow the researchers time to recover their temperature plates prior to dark. At the completion of each days burning the burn boss and holding boss will determine any needed monitoring/patrol for the evening or next day to insure containment and visitor safety.

XI. MONITORING PLAN:

PRE-BURN MONITORING:

All preburn monitoring is being accomplished by research (USGS) as part of their project. Vegetation is being identified and mapped for each block in great detail.

BURN MONITORING:

While the burn is in progress hourly recordings will be made of pertinent weather parameters, fuel moistures, fire behavior and rates of spread.

The Burn Boss will be informed of any adverse changes in weather or fire effects as they occur in order to modify firing to achieve proper effects.

Research personnel will be on site during these burns to provide input on block boundaries, research plot locations and other concerns relevant to their project.

POST-BURN MONITORING:

Post burn monitoring will be conducted as part of the research project by research personnel. Fire management monitoring personnel may assist in block monitoring if requested and if time allows.

XII. EXTERNAL & INTERNAL NOTIFICATION LIST

* see Regional Office notification (go-no-go) at the end of this listing

WHO?	PHONE #	DATE	INITIALS
**OTHER AGENCIES:**			
Fla. Division of Forestry (Permit; give certified burner #)	1-941-694-2536		
Ochopee Fire Control	695-4450		
EVER dispatch	1-305-242-7740		
Collier Sheriffs Office	774-6328		
Big Cypress Seminole Reservation	1-941-983-7029		
**LAND OWNERS:**		Tract #	
Dade-Collier Jetport	695-3300		
Calumet Oil Co. (Oil Pads)	695-3595		
Land Owners of properties directly adjacent to burn activities will be notified onsite if they are present on ignition days. (holding boss)			
Baxter, Et Al		553-53	
Himrod, Et Ux		572-34	
Peacock, Et Al		575-04	
Pinder		928-34	
Murphy		931-60	
Strayhorn, G		932-25	
Gresham		937-92	
Young, Et Al		948-61	
Rosher, Et Al		950-57	
Dawson, Et Al		952-59	
Strayhorn, N		963-33	

PRESERVE NOTIFICATIONS:			
Fire Management Officer			
Chief Rangers Office			
Superintendents Office			
Wildlife Biologist			
Research (USGS)			
Resource Mgt. Officer			

***As of this date (6/20/2000) the Regional office (FMO) will need to be contacted for concurrence before we conduct any burn. Also during regional planning levels 4 and 5, if and when the above concurrence requirement is lifted, we need to contact the regional office for concurrence on burning.**

		Date	Initials
Regional Office (FMO)	404-562-3108 x653		

XIII. PRESCRIBED BURN CHECKLIST:

**ITEM**	DATE:	INITIALS
Burn Boss has conducted burn block Preinspection		
Favorable general fire forecast (attach to plan file)		
Spot forecast obtained and within prescription (attach to plan file)		
Off-site contingency forces, if any, are available and committed.		
**Preburn briefing:**		
- Values at risk		
- Spot Weather Forecast		
- Ignition Plan		
- Burn Organization		
- Escape routes/Safety zones		
- Contingency Plan		
- Smoke Mgt.		
- Questions & Safety Discussion		
- Burn plans and Maps distributed		
**Other items of concern:**		
- Supplies & Equip. ready		
- Personnel qualified & equipped for assigned position		
- Load Calcs & manifests completed (heli Ops)		
- Smoke Mgt. Issues addressed		
**Reports:**		
- Performance Evals. completed for burn personnel		
- TF-1202 completed		
- Fire file on Burn completed (unit logs, costs)		

HOLDING MATRIX FOR RACCOON POINT BURN PLAN

This burn will be conducted over a period of about 10 years. Eighteen burn units will be treated in a rotation, which will have some being burned under winter, spring and summer conditions. The time between burns will vary by unit. Weather conditions will vary over the years of the project resulting in water level differences from one year to the next. Fuel loadings and the ratio of dead to live fuels will vary on the units depending on the length of time from the last burn treatment on that particular site. The holding actions listed in the matrix must therefore be somewhat generic and will provide a starting point from which the Burn Boss and the Holding Boss will adjust according to the conditions existing at the time of each burn treatment. It should be obvious but the older the rough and the drier the conditions the more complex the holding activity and the more forces required to complete the job (ie. a dry winter and an older rough may necessitate a helicopter on standby for holding). *** See attached map for research unit boundaries ***

The numbers of personnel and equipment noted in this matrix are intended as a guideline. If water levels, weather and fuel conditions are favorable the burn boss may determine that less resources are needed to safely burn any given unit. Conversely if conditions are not favorable, the minimums noted in the matrix may not be satisfactory and the burn boss will adjust to meet the situation.

UNIT	WINTER	SPRING	SUMMER
1<br><br>years<br>1998<br>2004	Trails form the boundaries on most of three sides of this unit; cypress domes connect much of the remaining edges. Buggies and/or ATCs will be used for holding along the trail areas. ATC lines will be established in sections of the line where there are gaps between domes or trail sections. (If water levels are still established in the prairies ATC lines may not be necessary and will not be used). RCW site #25 will be mechanically treated prior to the burn. 2 buggies and 1 ATC will be on site and 6 fire qualified personnel. Burn boss will also be ignition spec.		
2<br><br>1999<br>2005	Trails form the boundaries on the south and east sides of the unit, cypress domes connect much of the remaining edges. Buggies and/or ATCs will be used for holding along the trail areas. ATC lines will be established in sections of the line where there are gaps between domes or trail sections. (If water levels are still established in the prairies ATC lines may not be necessary and will not be used). 2 buggies and 1 ATC will be on site and 6 fire qualified personnel. Burn boss will also be ignition spec.		
3<br><br>1998<br>2001<br>2004<br>2007	Trails form the boundaries on the north, south and part of the east sides of the unit; cypress domes connect much of the remaining edges. Buggies and/or ATCs will be used for holding along the trail areas. ATC lines will be established in sections of the line where there are gaps between domes or trail sections. (If water levels are still established in the prairies ATC lines may not be necessary and will not be used). 2 buggies and 1 ATC will be on site and 6 fire qualified personnel. Burn boss will also be ignition spec.		

4<br><br>years<br>2098<br>2004			This should be the wettest burn period. Buggy trails and pad 4 will serve as fire lines on the north, east and south. Cypress domes will be used for most of the rest of the unit boundary. Helicopter with bucket will standby at N. end of pad 4 if needed (heli may be used for ignition or observation, in which case bucket will be on site for use if needed). Buggies and/or ATCs will be used for holding on trails. One buggy, one engine and 1 ATC will be on site and 5 fire qualified personnel.
5<br><br>1996<br>2000<br>2002<br>2005		This should be the driest burn period. Buggy trails and pad 4 will serve as fire lines on the north, west and south; cypress domes will cover most of the rest of the unit boundary. Buggies and/or ATCs will be used for holding along the trail areas; ATC lines will be used to connect areas between domes. Oil flow line between pad 4 and 5 will be foamed with the engine where needed. Helicopter with bucket will standby at N. end of pad 4 if needed (heli may be used for ignition or observation, in which case bucket will be on site for use if needed). Buoywall tank may be set up, if needed, to supply water for bucket. 1 buggy, 1 engine and 1 ATC will be on site and 6 fire qualified personnel. Burn boss will also be ignition spec. *	
6<br><br>1997<br>2000<br>2003<br>2006	Buggy trails will be used as lines on all but the northeast side. Cypress domes will be use for most of the rest of the line. ATC lines will be used to connect areas between domes (If water levels are still established in the prairies ATC lines may not be necessary and will not be used). RCW site #14 will be mechanically treated prior to the burn. Buggies and ATCs will be used for holding along the trail areas. 2 buggies and 1 ATC will be on site and 6 fire qualified personnel. Burn boss will also be ignition spec.		
7<br><br>1997<br>2000<br>2003<br>2006			This should be the wettest burn period. Trails and oil pad roads will be used as lines on three sides. The southern line will go between domes and may require some limited ATC line to connect the domes Buggies and/or ATCs will be used for holding on trails. One buggy, one engine and 1 ATC will be on site and 5 fire qualified personnel. Oil flow line along the road between pads 1 and pad 4 will be foamed with the engine where needed. Burn boss will also be ignition spec.

8<br><br>years<br>2000<br>2001<br>2004<br>2007		This should be the driest burn period. Trails and oil pad roads will be used as lines on the west, north and northeast sides. The southern and southeastern line will go between domes and may require some limited ATC line to connect the domes. Buggies and/or ATCs will be used for holding on trails. RCW site #10 will be mechanically treated prior to the burn. One buggy, one engine and 1 ATC will be on site and 6 fire qualified personnel. Oil flow line along the road between pad 1 and pad 4 and pad 5 will be foamed with the engine where needed. Helicopter with bucket will standby at N. end of pad 4 if needed (heli may be used for ignition or observation, in which case bucket will be on site for use if needed). Burn boss will also be ignition spec.	
9<br><br>1996<br>2002		This should be the driest burn period. This is also a long rotation plot so fuels should be heavier or contain more dead component. The oil pad road and buggy trails will serve as the firelines on the east and northeast side of the unit; wet cypress and domes will serve as the boundary on the rest of the unit. Buggies and/or ATCs will be used for holding along the trail areas; ATC lines will be used to connect areas between domes. Buoywall tank may be set up to supply water for bucket as needed. 1 buggy, 1 engine and 2 ATC will be on site and 6 fire qualified personnel. Burn boss will also be ignition spec. *	
10<br><br>1996<br>2000<br>2002<br>2005			This should be the wettest burn period. Trails and oil pad roads will be used as lines on the east, and south sides. The north and western line will go between domes and may require some limited ATC line to connect the domes. Buggies and/or ATCs will be used for holding on trails. One buggy, one engine and 1 ATC will be on site and 5 fire qualified personnel. Oil flow line along the road between pad 1 and pad 4 will be foamed with the engine where needed. Burn boss will also be ignition spec.
11<br><br>1997<br>2000<br>2003<br>2006		This should be the driest burn period. Oil pad roads will serve as fire lines on the west and south; cypress domes will cover most of the rest of the unit boundary. Buggies and/or ATCs will be used for holding along the trail areas; ATC lines will be used to connect areas between domes. Oil flow line between pad 1 and 4 and between 1-2 will be foamed with the engine where needed. Helicopter with bucket will standby at W. end of pad 1 if needed (heli may be used for ignition or observation, in which case bucket will be on site for use if needed). Buoywall tank will be set up to supply water for bucket as needed. 1 buggy, 1 engine and 1 ATC will be on site and 6 fire qualified personnel. Burn boss will also be ignition spec. *	

12<br><br>years<br>2000<br>2004		This should be the driest burn period. Trails and oil pad roads will be used as lines on the west, north and east. The southern line will go between domes and may require some ATC line to connect the domes. Buggies and/or ATCs will be used for holding on trails. One buggy, one engine and 1 ATC will be on site and 6 fire qualified personnel. Helicopter with bucket will standby at W. end of pad 1 if needed (heli may be used for ignition or observation, in which case bucket will be on site for use if needed). Oil flow line along the road between pad 1 and pad 4 will be foamed with the engine where needed. Burn boss will also be ignition spec.	
13<br><br>1999<br>2002<br>2005	Cypress domes and wet areas form all of the boundaries on this unit, ATC or buggy lines will be established in sections of the line where there are gaps between domes or wet sections. (If water levels are still established in the prairies ATC lines may not be necessary and will not be used). RCW site #11 will be mechanically treated prior to the burn. 1 buggy and 2 ATCs will be on site and 6 fire qualified personnel. Burn boss will also be ignition spec.		
14<br><br>1998<br>2001<br>2004<br>2007			This should be the wettest burn period. Cypress domes and wet areas form all of the boundaries on this unit, ATC or buggy lines will be established in sections of the line where there are gaps between domes or wet sections. Several buggy trails run through the unit. RCW site #16 will be mechanically treated prior to the burn. 1 buggy and 2 ATCs will be on site and 6 fire qualified personnel. Burn boss will also be ignition spec.
15<br><br>1997<br>2003			This should be the wettest burn period. Eleven mile road will be used as the line on the west. The rest of the units firelines will go between domes and wet areas and may require some limited ATC or buggy line to connect the domes Buggies and ATCs will be used for holding. RCW site #11 will be mechanically treated prior to the burn. One buggy, one engine and 1 ATC will be on site and 5 fire qualified personnel. Burn boss will also be ignition spec.
16<br><br>1997<br>2003		This should be the driest burn period. This is also a long rotation plot so fuels should be heavier or contain more dead component. Cypress domes and wet areas form most of the boundaries on this unit, eleven mile road forms the east line and several areas of buggy trail connect some of the wet areas. ATC or buggy lines will be established in sections of the line where there are gaps between domes or wet sections. 1 buggy and 2 ATCs will be on site and 6 fire qualified personnel. Burn boss will also be ignition spec.	

17<br><br>years<br>1997<br>2003	Cypress domes and wet areas form most of the boundaries on this unit, eleven mile road serves as line in 2 small sections. ATC or buggy lines will be established in sections of the line where there are gaps between domes or wet sections. (If water levels are still established in the prairies ATC lines may not be necessary and will not be used). Eleven mile road runs through the middle of the unit. 1 buggy, 1 engine and 2 ATCs will be on site and 6 fire qualified personnel. Burn boss will also be ignition spec.		
18<br><br>1996<br>2002			This should be the wettest burn period. Eleven mile road will be used as the line on the east. The rest of the units firelines will go between domes and wet areas and may require some limited ATC or buggy line to connect the domes. Buggies and ATCs will be used for holding. One buggy, one engine and 1 ATC will be on site and 5 fire qualified personnel. Burn boss will also be ignition spec.

- Where Helicopter or equipment is listed as needed the decision to use these items will be based on current water levels, fuel and weather conditions at the time of the burn. The decision will be coordinated between the Holding and Burn bosses.

The numbers of personnel and equipment noted in this matrix are intended as a guideline. If water levels, weather and fuel conditions are favorable the burn boss may determine that less resources are needed to safely burn any given unit. Conversely if conditions are not favorable, the minimums noted in the matrix may not be satisfactory and the burn boss will adjust to meet the situation.

RACCOON POINT FIRE ECOLOGY RESEARCH PROJECT

WILDLAND AND PRESCRIBED FIRE COMPLEXITY RATING WORKSHEET

Complexity element	*Weighting factor*	*Complexity value*	*Total points*
**Safety**	5	3	15
**Threats to boundaries**	5	3	15
**Fuels and fire behavior**	5	3	15
**Objectives**	4	3	12
**Management organization**	4	3	12
**Improvements**	3	3	9
**Natural, cultural, social values**	3	3	9
**Air quality values**	3	3	9
**Logistics**	3	1	3
**Political concerns**	2	3	6
**Tactical operations**	2	3	6
**Interagency coordination**	1	1	1
**Overall complexity points**			112
**Complexity Rating (circle)**	L	M	H

Complexity Value Breakpoints:

Low	*40 - 90*
Moderate	*91 – 140*
High	*141 – 200*

The Wildland and Prescribed Fire Complexity Analysis provides a method to assess the complexity of both wildland and prescribed fires. The analysis incorporates an assigned numeric rating complexity value for specific complexity elements that are weighted in their contribution to overall complexity. To use the worksheet, assign a Complexity Value ranging from 1 (low) to 5 (high) for each Complexity Element. Complete the total points column by multiplying the Weighting Factor by the Complexity Value. Add the values in the Total Points column to arrive at the value for the Overall Complexity Points. Compare the Overall Complexity Points with the Complexity Value Breakpoints shown above to determine the Complexity Rating.

*** All of the elements in the above rating are estimated for burning during the driest periods and would be considerably lower for burns at other times of the year.**

9.2 Tree Plot Maps Showing Locations of All Trees

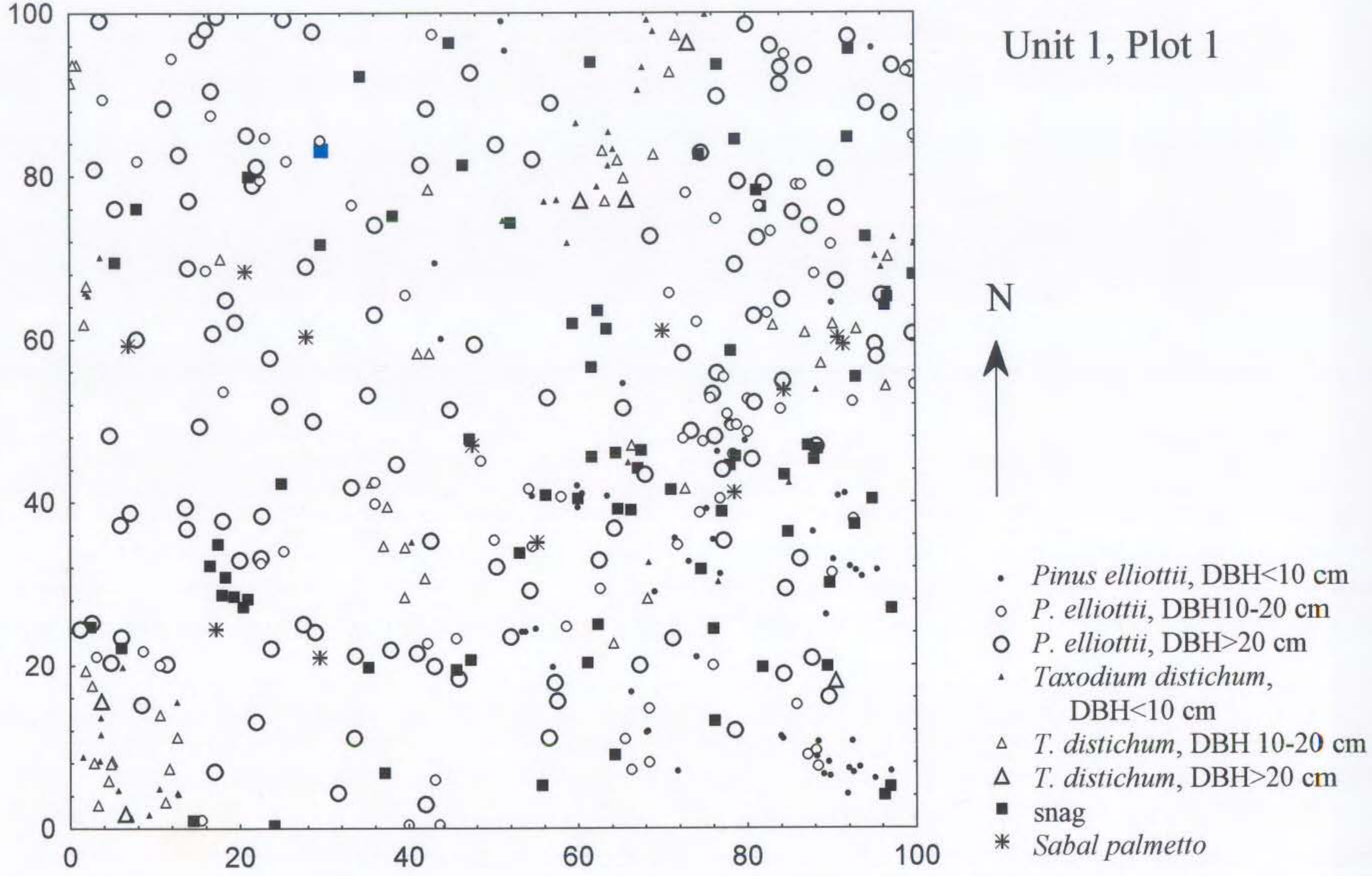
Unit 1, Plot 1
N
100
80
60
40
20
0
0
20
40
60
80
100
Pinus elliottii, DBH<10 cm
P. elliottii, DBH10-20 cm
P. elliottii, DBH>20 cm
Taxodium distichum, DBH<10 cm
T. distichum, DBH 10-20 cm
T. distichum, DBH>20 cm
snag
Sabal palmetto

Unit 1, Plot 2

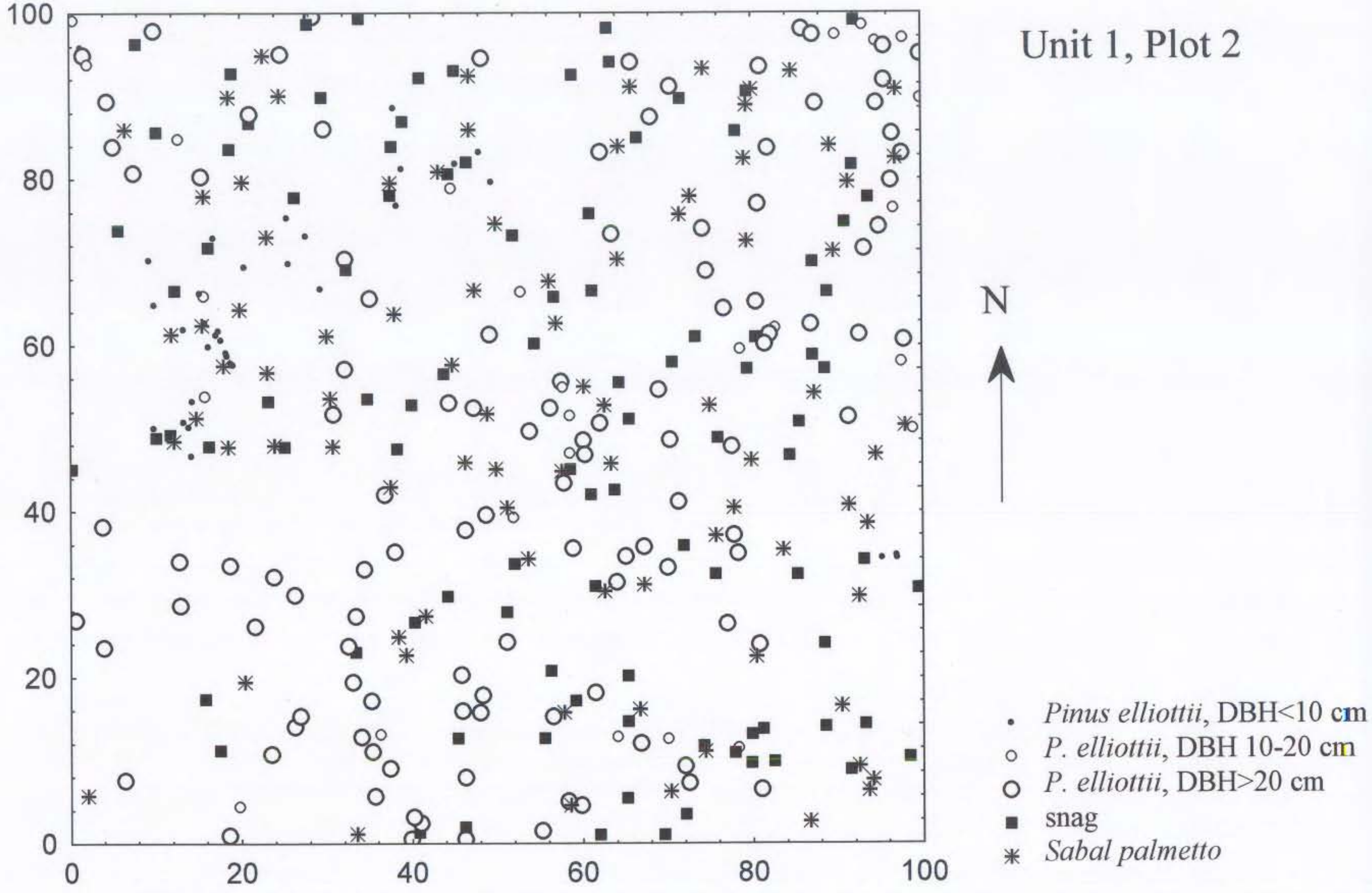
0
20
40
60
80
100
N
• Pinus elliottii, DBH<10 cm
○ P. elliottii, DBH 10-20 cm
○ P. elliottii, DBH>20 cm
■ snag
✱ Sabal palmetto

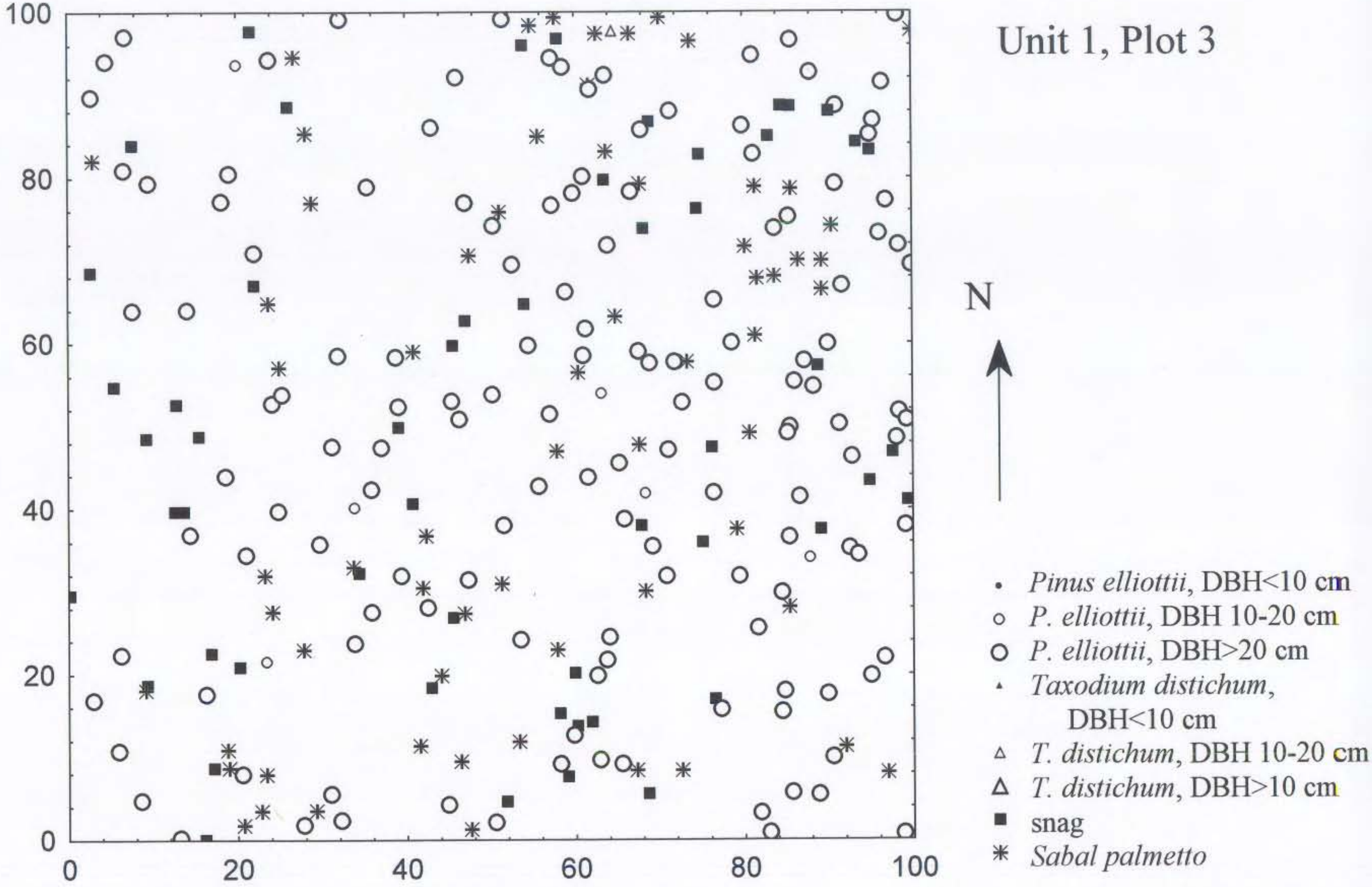

Unit 1, Plot 3
100
80
60
40
20
0
0
20
40
60
80
100
N
• *Pinus elliottii*, DBH<10 cm
○ *P. elliottii*, DBH 10-20 cm
O *P. elliottii*, DBH>20 cm
▴ *Taxodium distichum*, DBH<10 cm
△ *T. distichum*, DBH 10-20 cm
Δ *T. distichum*, DBH>10 cm
■ snag
✳ *Sabal palmetto*

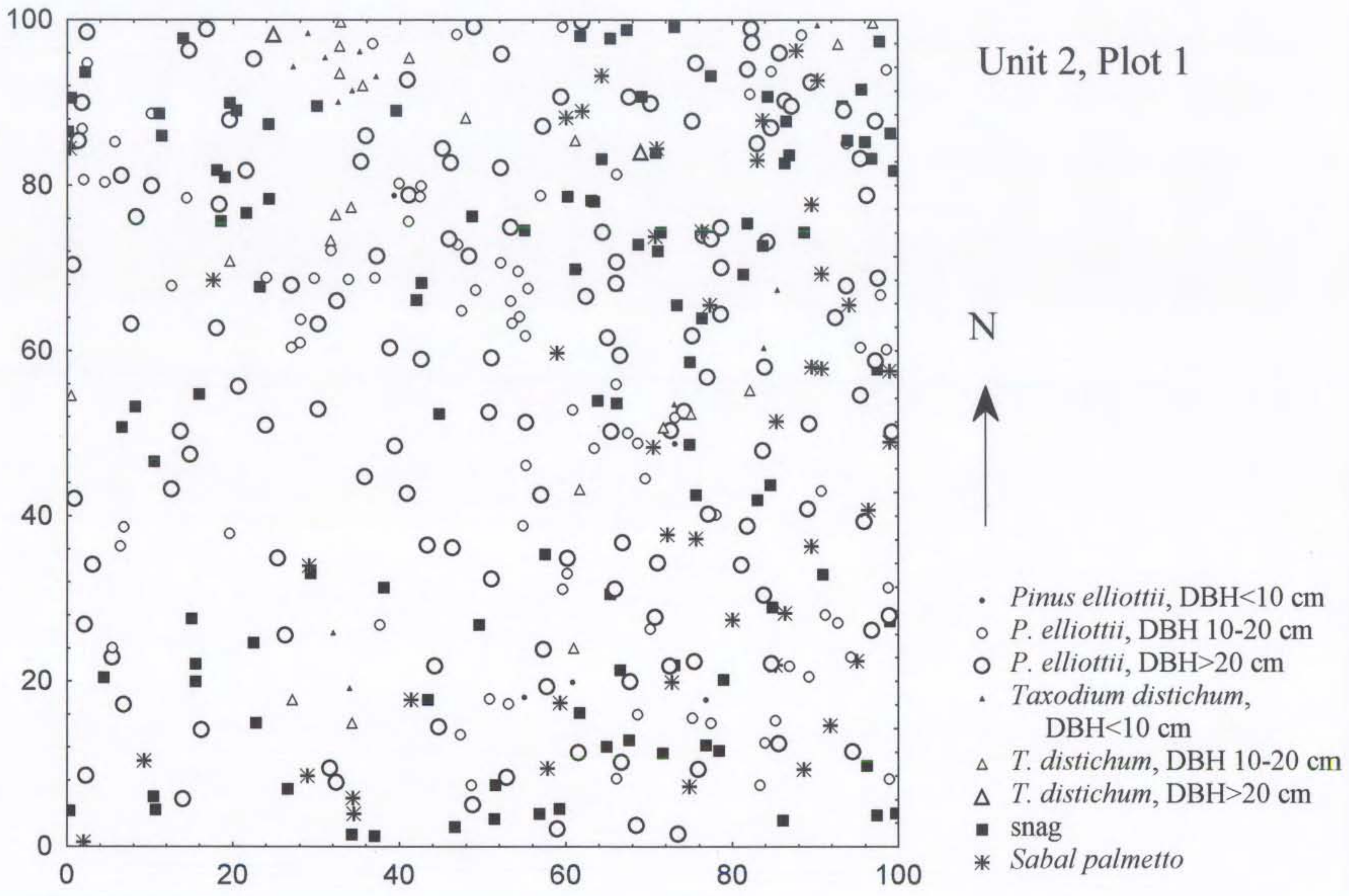
Unit 2, Plot 1
N
0
20
40
60
80
100
Pinus elliottii, DBH<10 cm
P. elliottii, DBH 10-20 cm
P. elliottii, DBH>20 cm
Taxodium distichum, DBH<10 cm
T. distichum, DBH 10-20 cm
T. distichum, DBH>20 cm
snag
Sabal palmetto

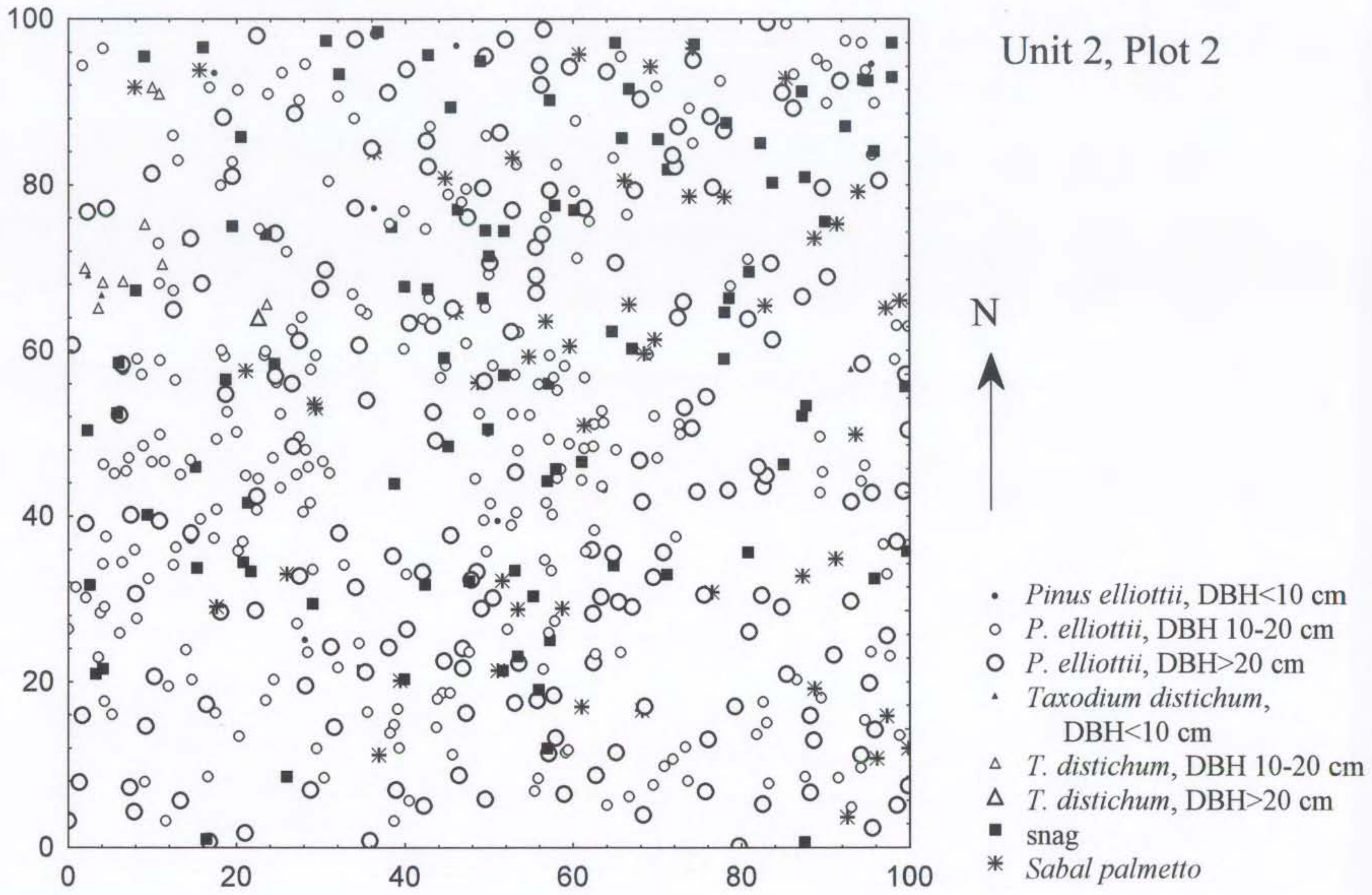
N
0
20
40
60
80
100
Pinus elliottii, DBH<10 cm
P. elliottii, DBH 10-20 cm
P. elliottii, DBH>20 cm
Taxodium distichum, DBH<10 cm
T. distichum, DBH 10-20 cm
T. distichum, DBH>20 cm
snag
Sabal palmetto

Unit 2, Plot 2

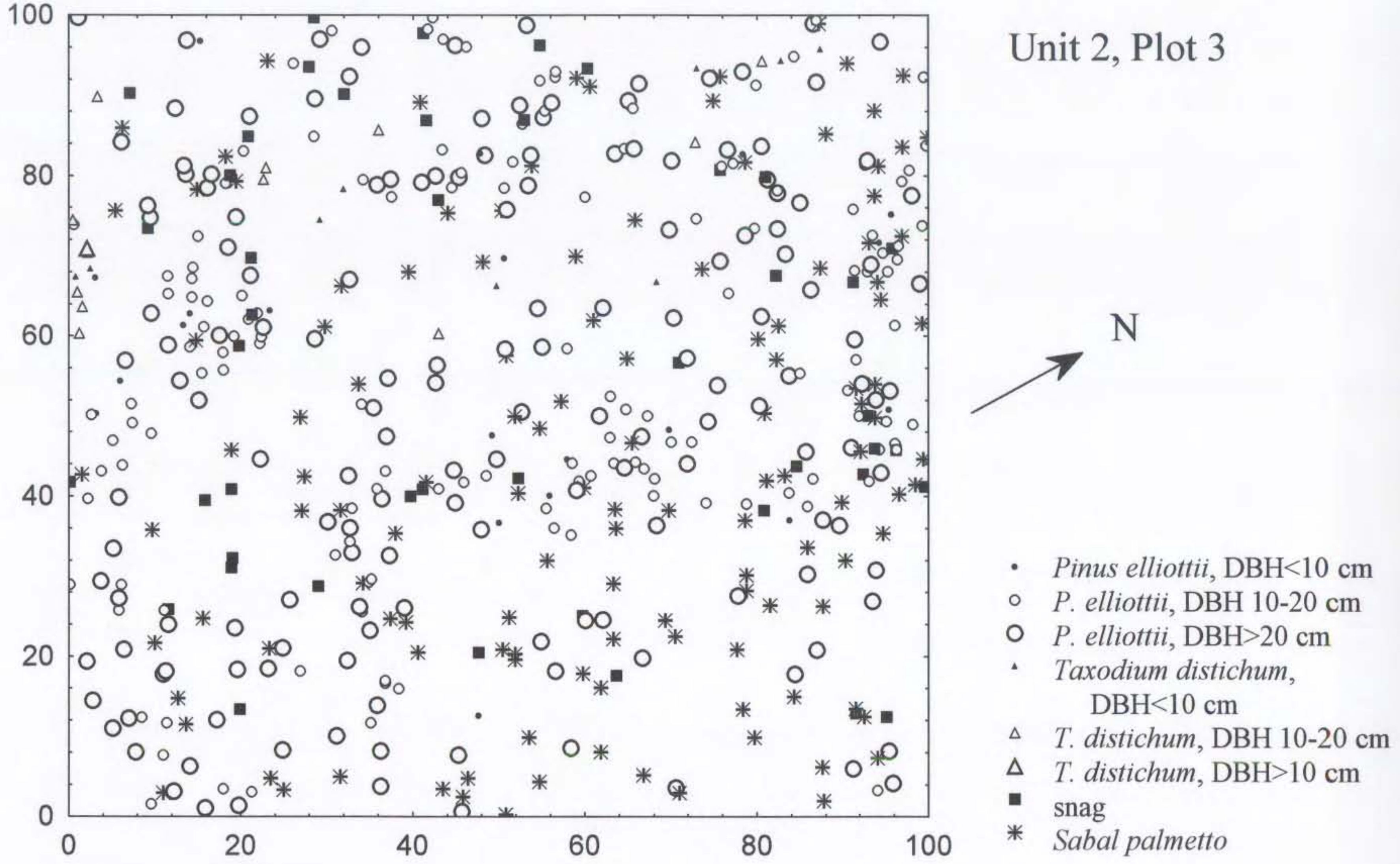

Unit 2, Plot 3
N
0
20
40
60
80
100
Pinus elliottii, DBH<10 cm
P. elliottii, DBH 10-20 cm
P. elliottii, DBH>20 cm
Taxodium distichum, DBH<10 cm
T. distichum, DBH 10-20 cm
T. distichum, DBH>10 cm
snag
Sabal palmetto

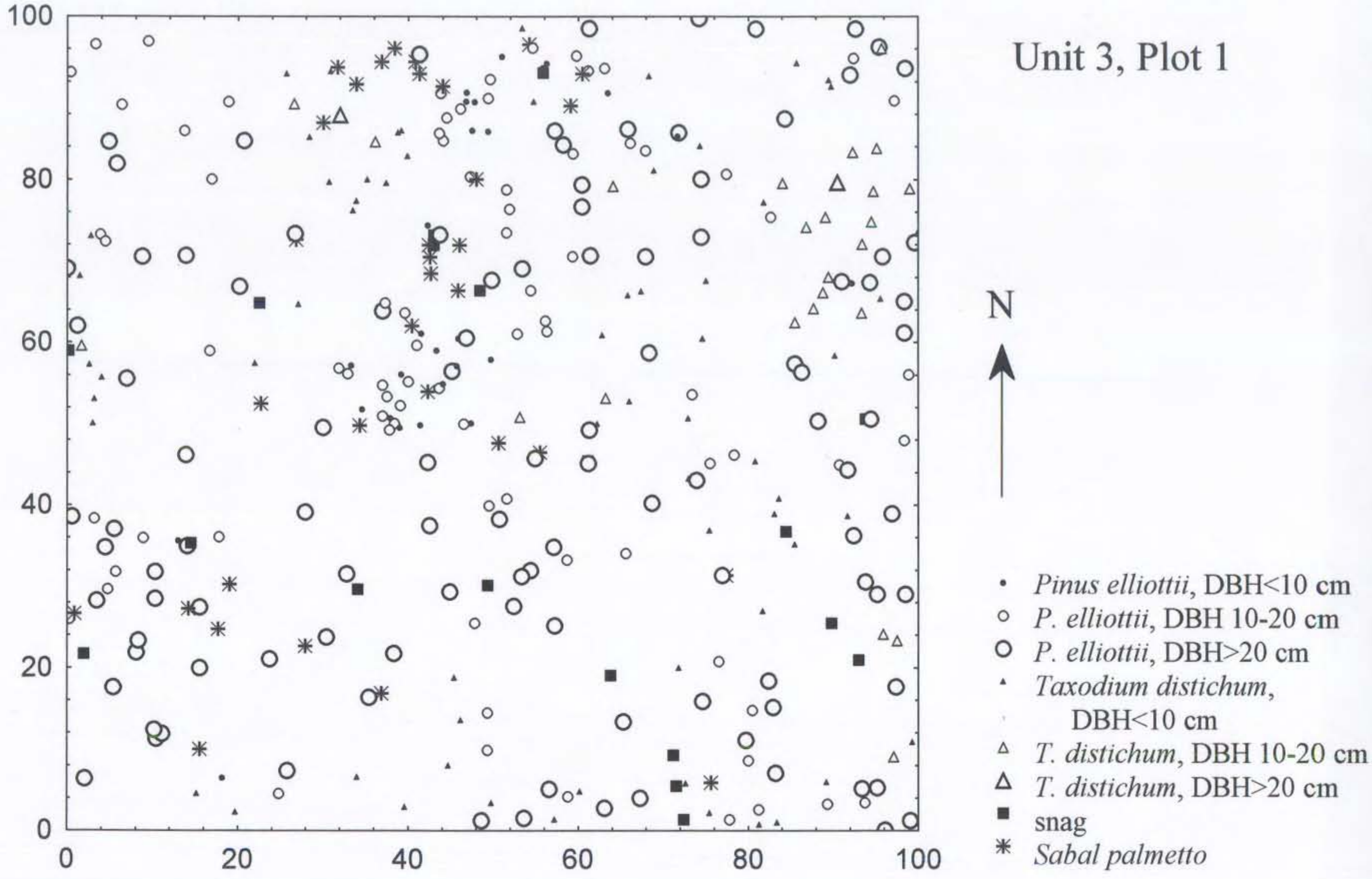
Unit 3, Plot 1
N
0
20
40
60
80
100
• Pinus elliottii, DBH<10 cm
○ P. elliottii, DBH 10-20 cm
O P. elliottii, DBH>20 cm
▴ Taxodium distichum, DBH<10 cm
△ T. distichum, DBH 10-20 cm
Δ T. distichum, DBH>20 cm
■ snag
✳ Sabal palmetto

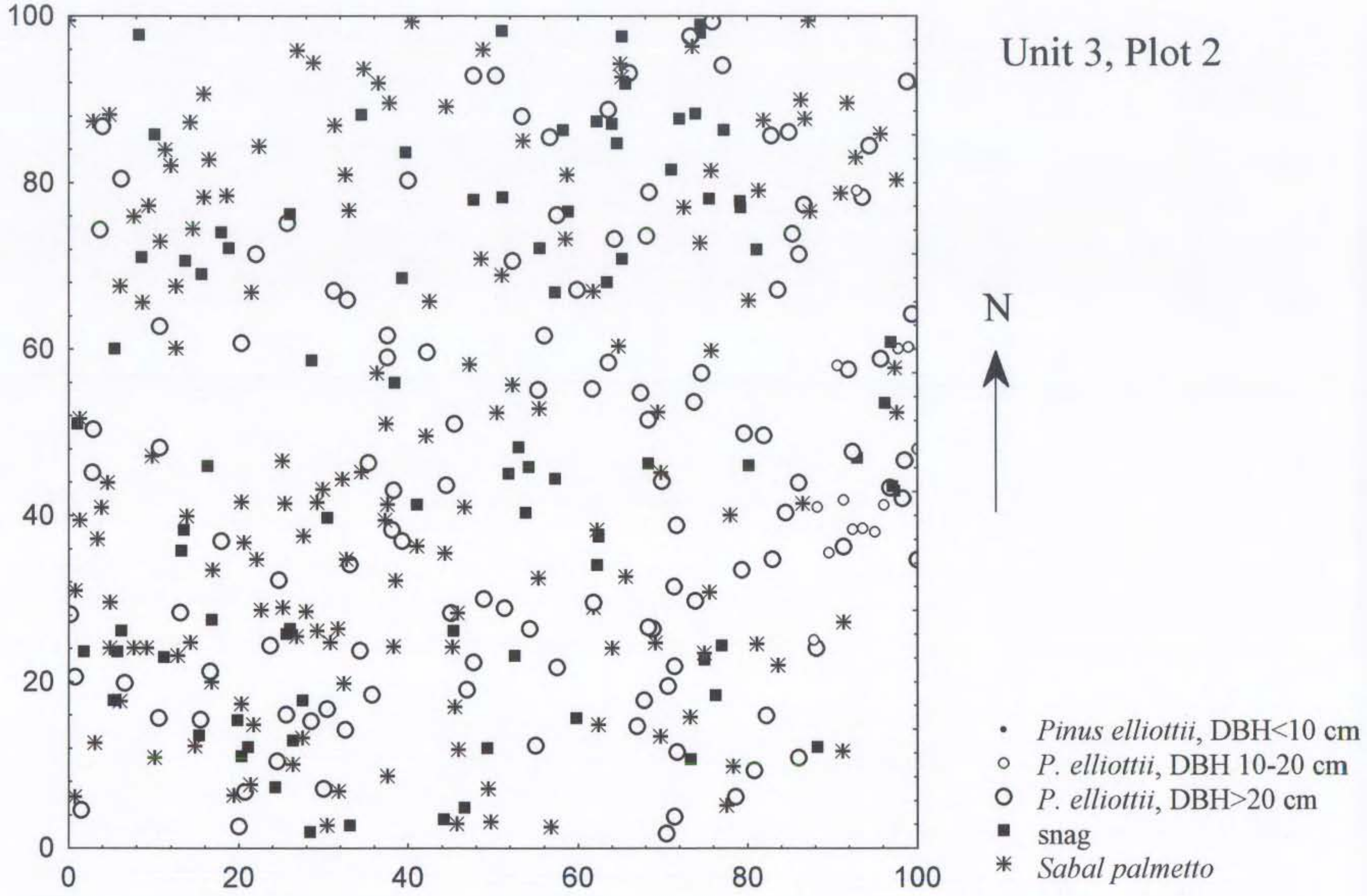

Unit 3, Plot 2
N
100
80
60
40
20
0
0
20
40
60
80
100
Pinus elliottii, DBH<10 cm
P. elliottii, DBH 10-20 cm
P. elliottii, DBH>20 cm
snag
Sabal palmetto

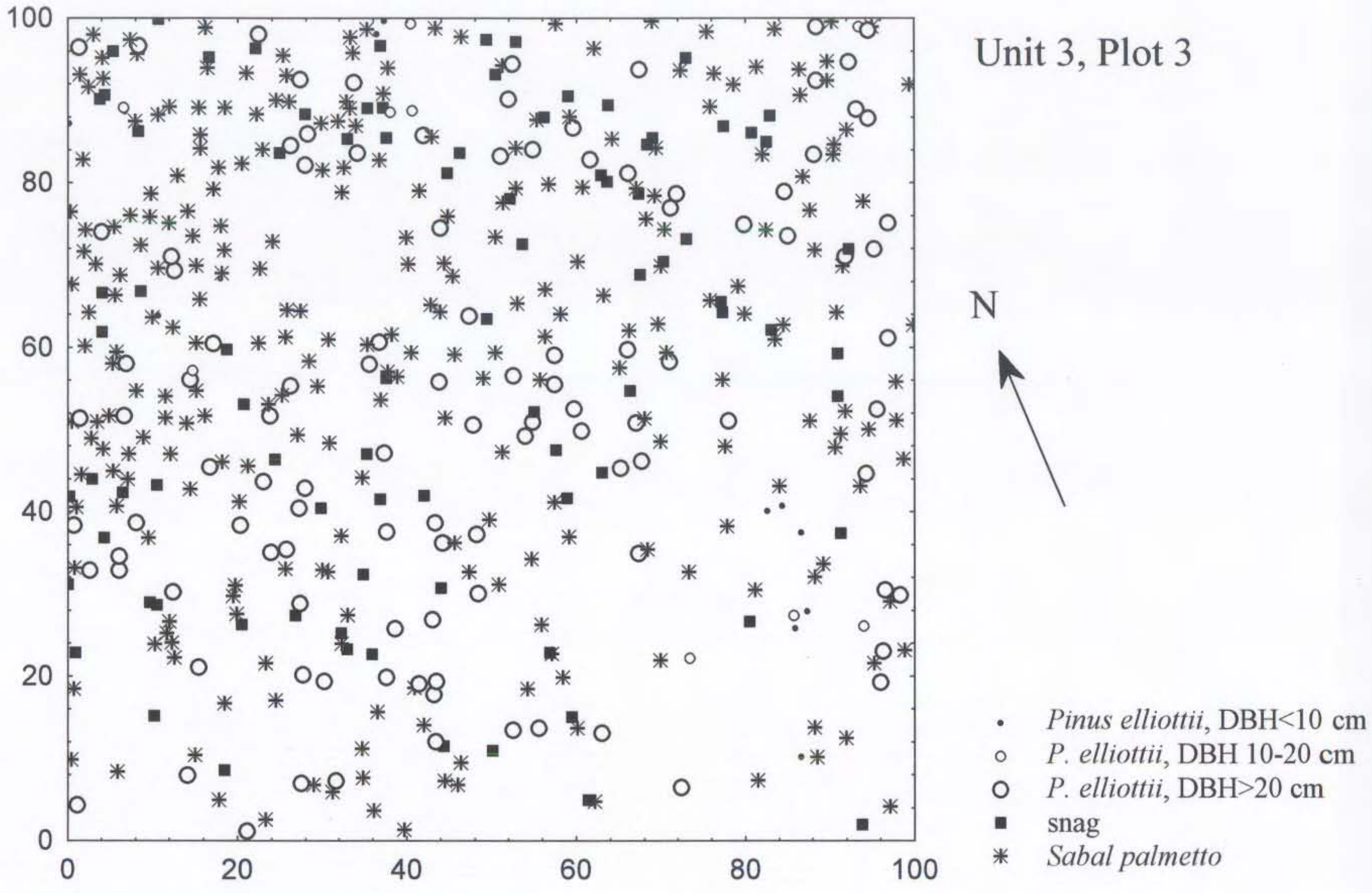
Unit 3, Plot 3
N
100
80
60
40
20
0
0
20
40
60
80
100
Pinus elliottii, DBH<10 cm
P. elliottii, DBH 10-20 cm
P. elliottii, DBH>20 cm
snag
Sabal palmetto

Unit 4, Plot 1

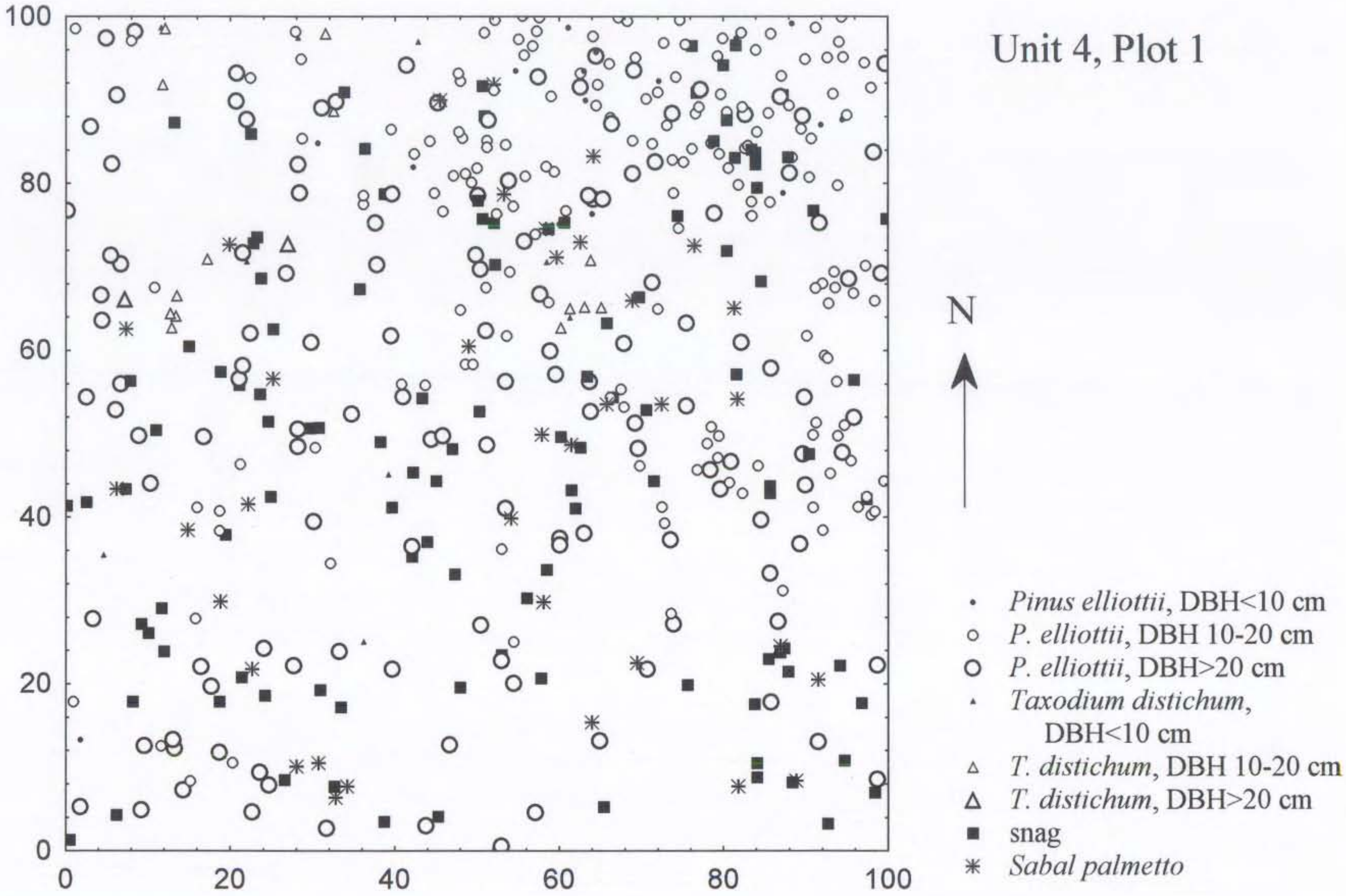

100
80
60
40
20
0
0
20
40
60
80
100
N
Pinus elliottii, DBH<10 cm
P. elliottii, DBH 10-20 cm
P. elliottii, DBH>20 cm
Taxodium distichum, DBH<10 cm
T. distichum, DBH 10-20 cm
T. distichum, DBH>20 cm
snag
Sabal palmetto

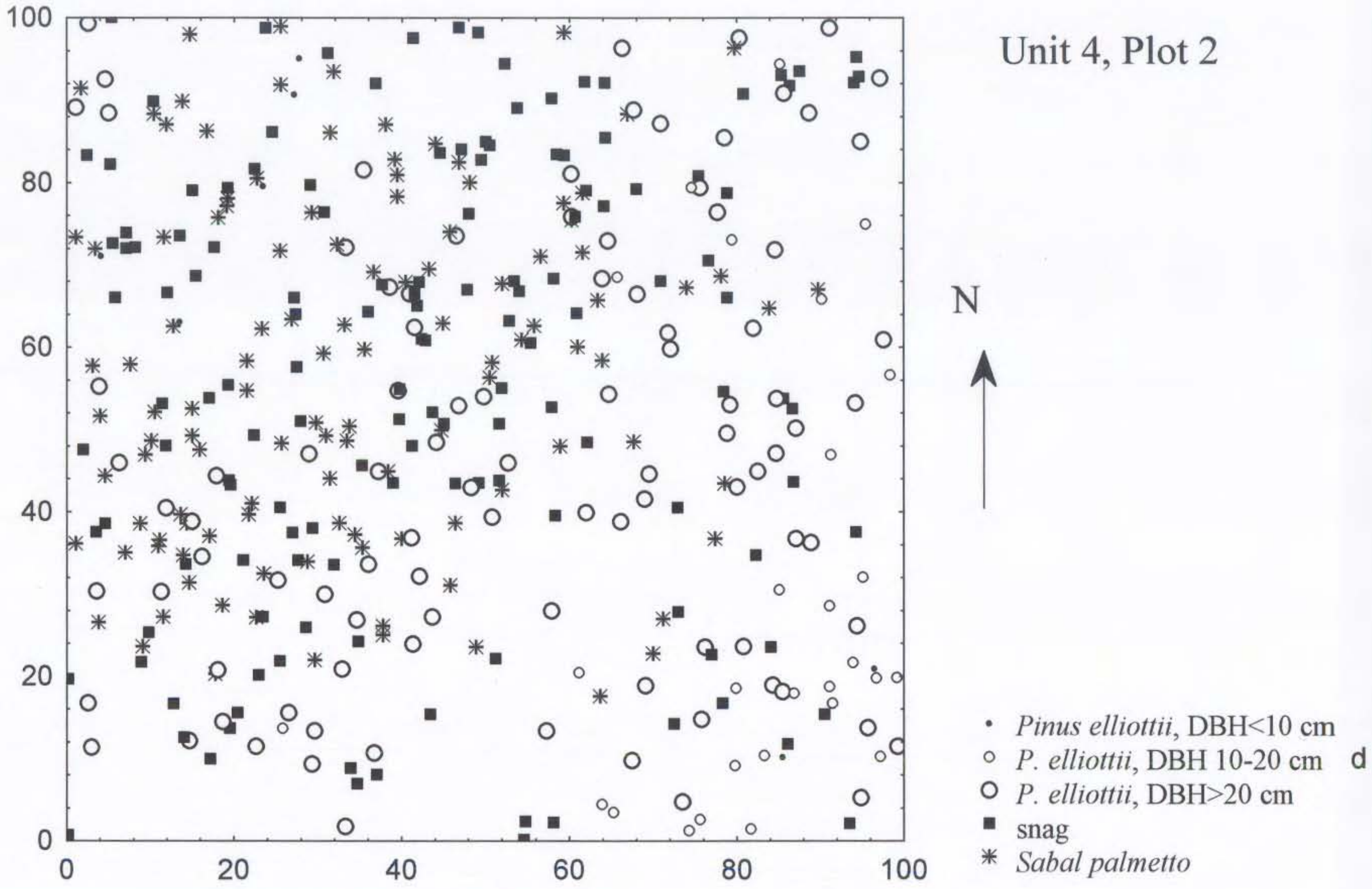

Unit 4, Plot 2
N
100
80
60
40
20
0
0
20
40
60
80
100
• Pinus elliottii, DBH<10 cm
○ P. elliottii, DBH 10-20 cm
O P. elliottii, DBH>20 cm
■ snag
* Sabal palmetto
d

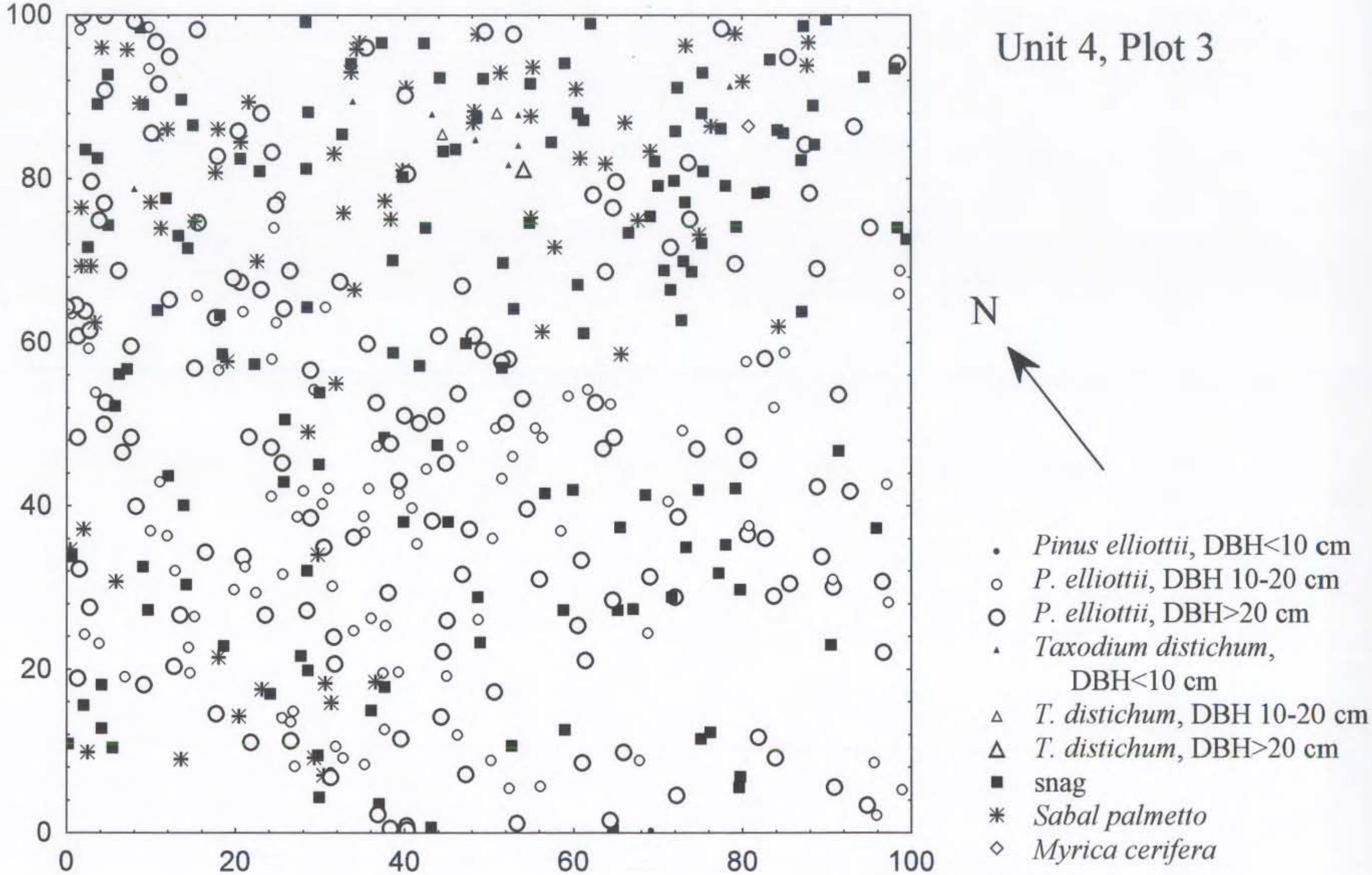
Unit 4, Plot 3
N
0
20
40
60
80
100
Pinus elliottii, DBH<10 cm
P. elliottii, DBH 10-20 cm
P. elliottii, DBH>20 cm
Taxodium distichum, DBH<10 cm
T. distichum, DBH 10-20 cm
T. distichum, DBH>20 cm
snag
Sabal palmetto
Myrica cerifera

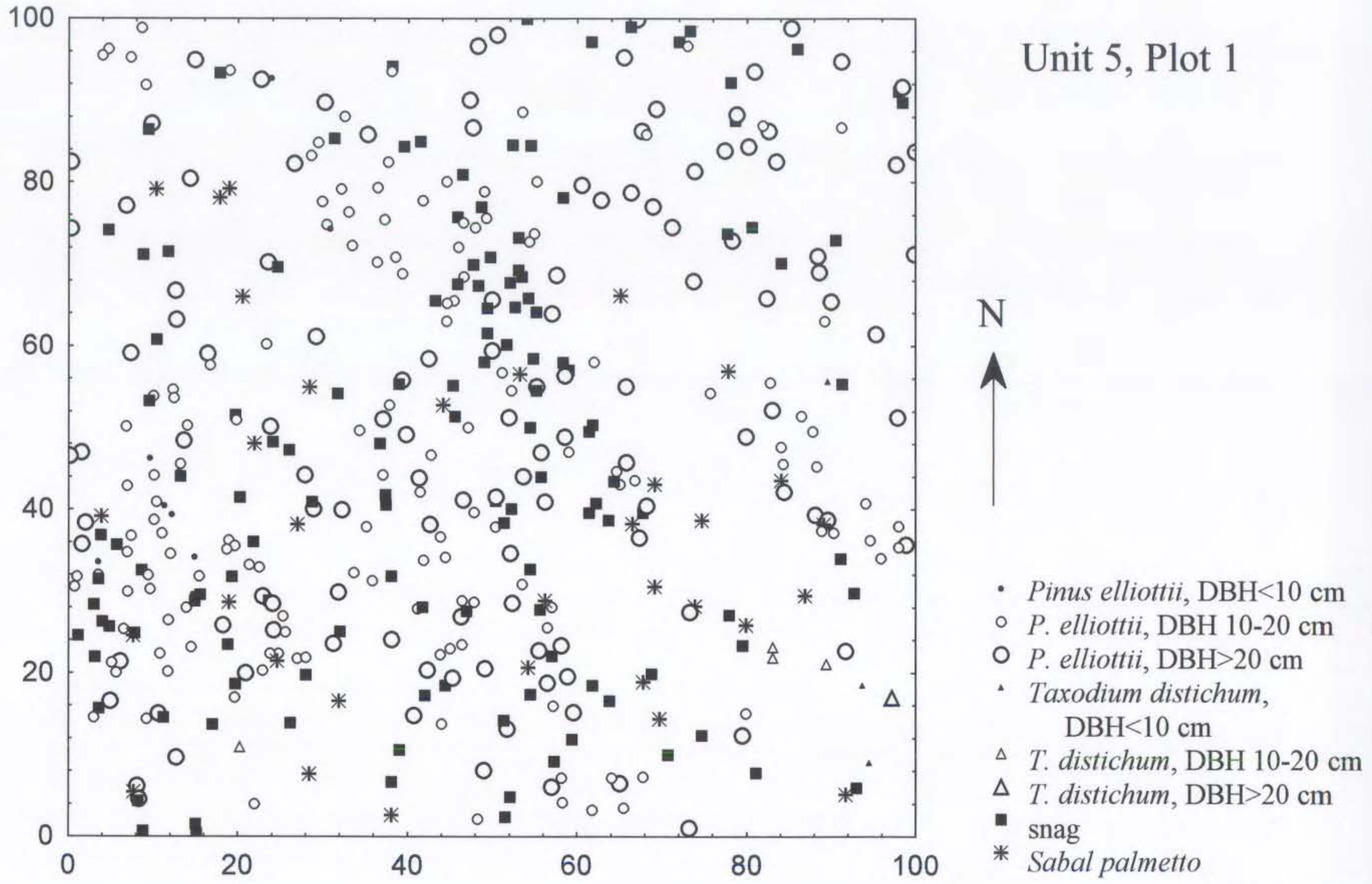
Unit 5, Plot 1
N
100
80
60
40
20
0
0
20
40
60
80
100
Pinus elliottii, DBH<10 cm
P. elliottii, DBH 10-20 cm
P. elliottii, DBH>20 cm
Taxodium distichum, DBH<10 cm
T. distichum, DBH 10-20 cm
T. distichum, DBH>20 cm
snag
Sabal palmetto

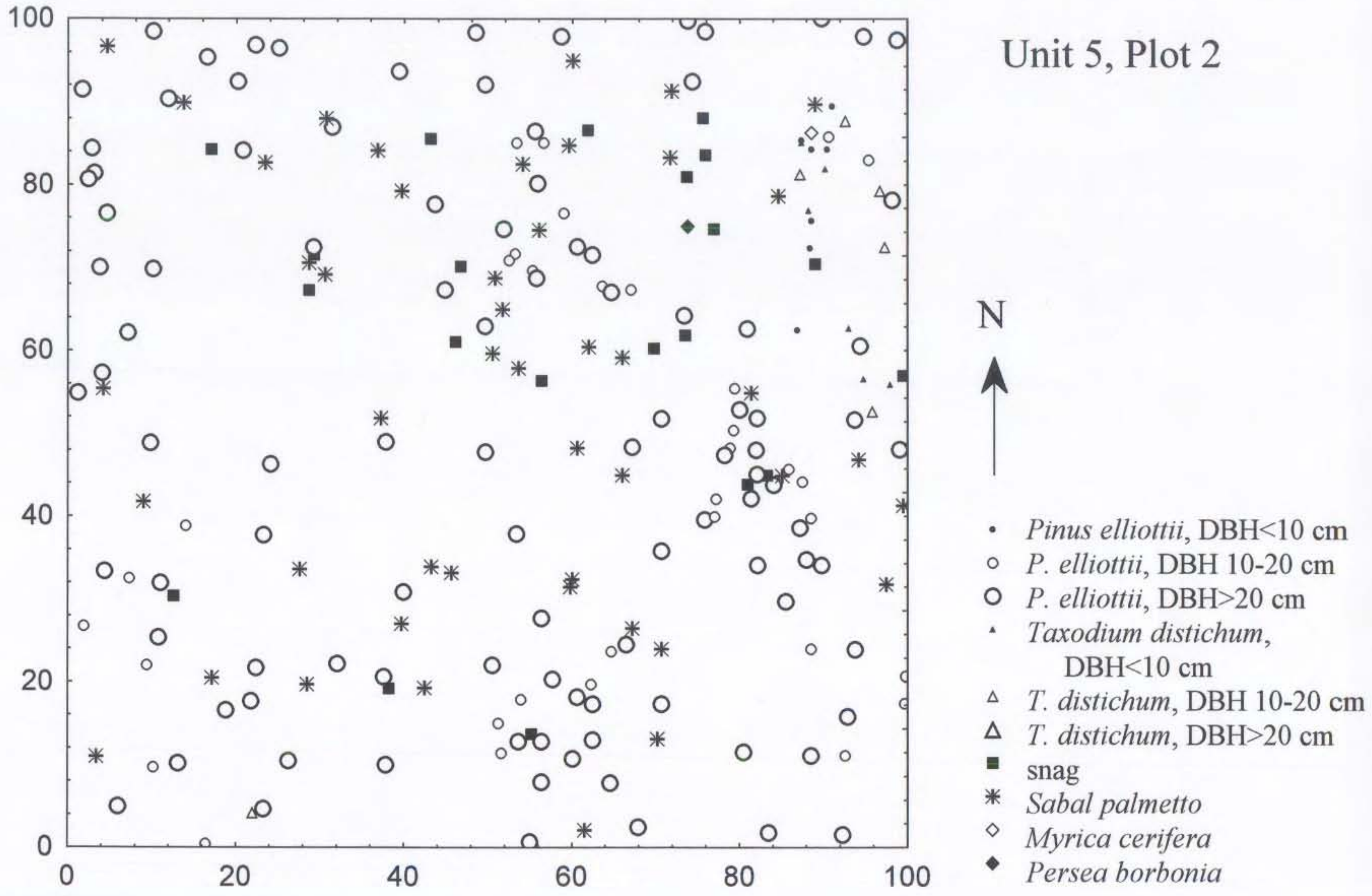
Unit 5, Plot 2
N
0
20
40
60
80
100
• Pinus elliottii, DBH<10 cm
○ P. elliottii, DBH 10-20 cm
O P. elliottii, DBH>20 cm
▴ Taxodium distichum, DBH<10 cm
△ T. distichum, DBH 10-20 cm
Δ T. distichum, DBH>20 cm
■ snag
✳ Sabal palmetto
◇ Myrica cerifera
◆ Persea borbonia

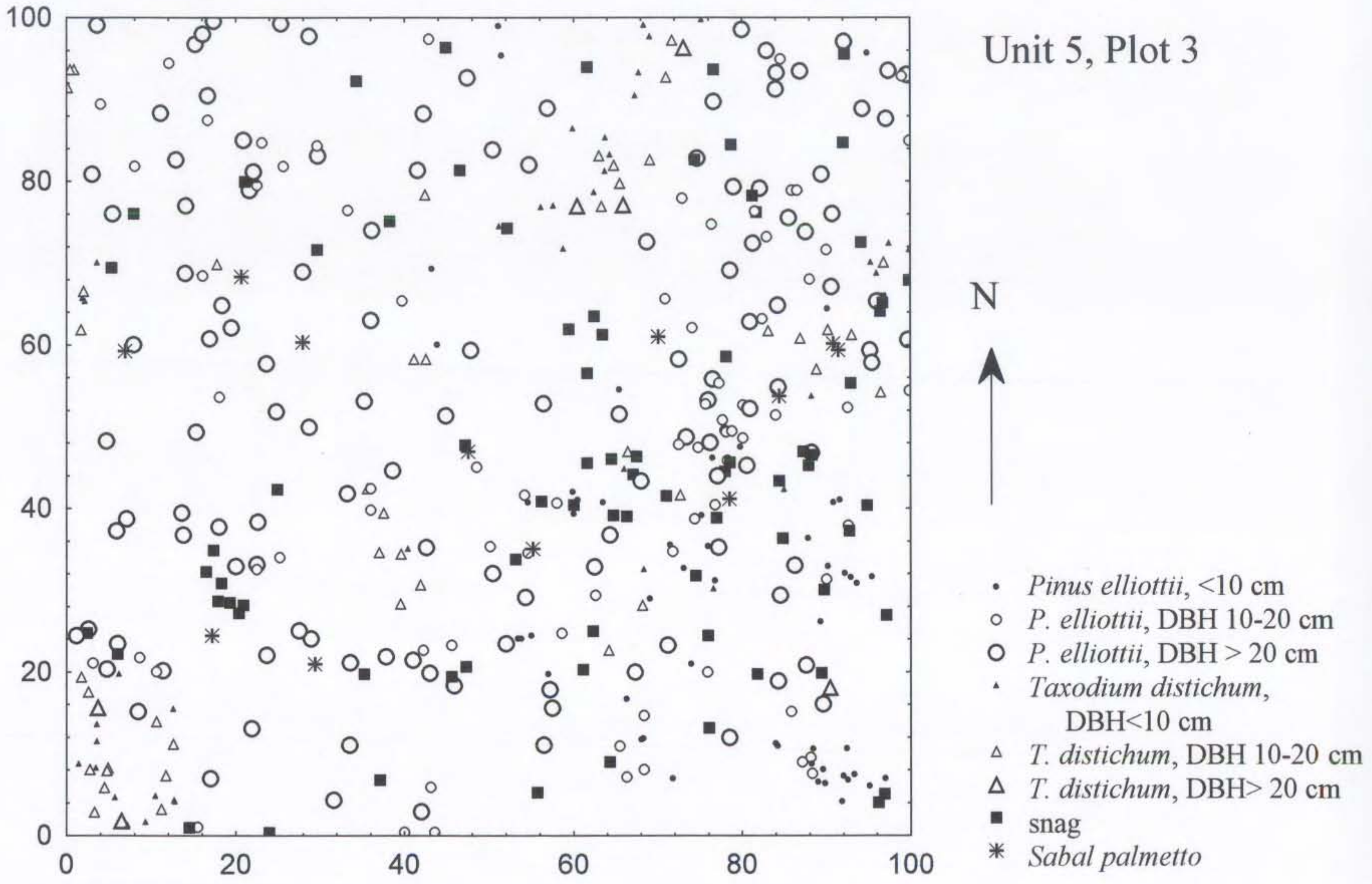

Unit 5, Plot 3
N
0
20
40
60
80
100
Pinus elliottii, <10 cm
P. elliottii, DBH 10-20 cm
P. elliottii, DBH > 20 cm
Taxodium distichum, DBH<10 cm
T. distichum, DBH 10-20 cm
T. distichum, DBH> 20 cm
snag
Sabal palmetto

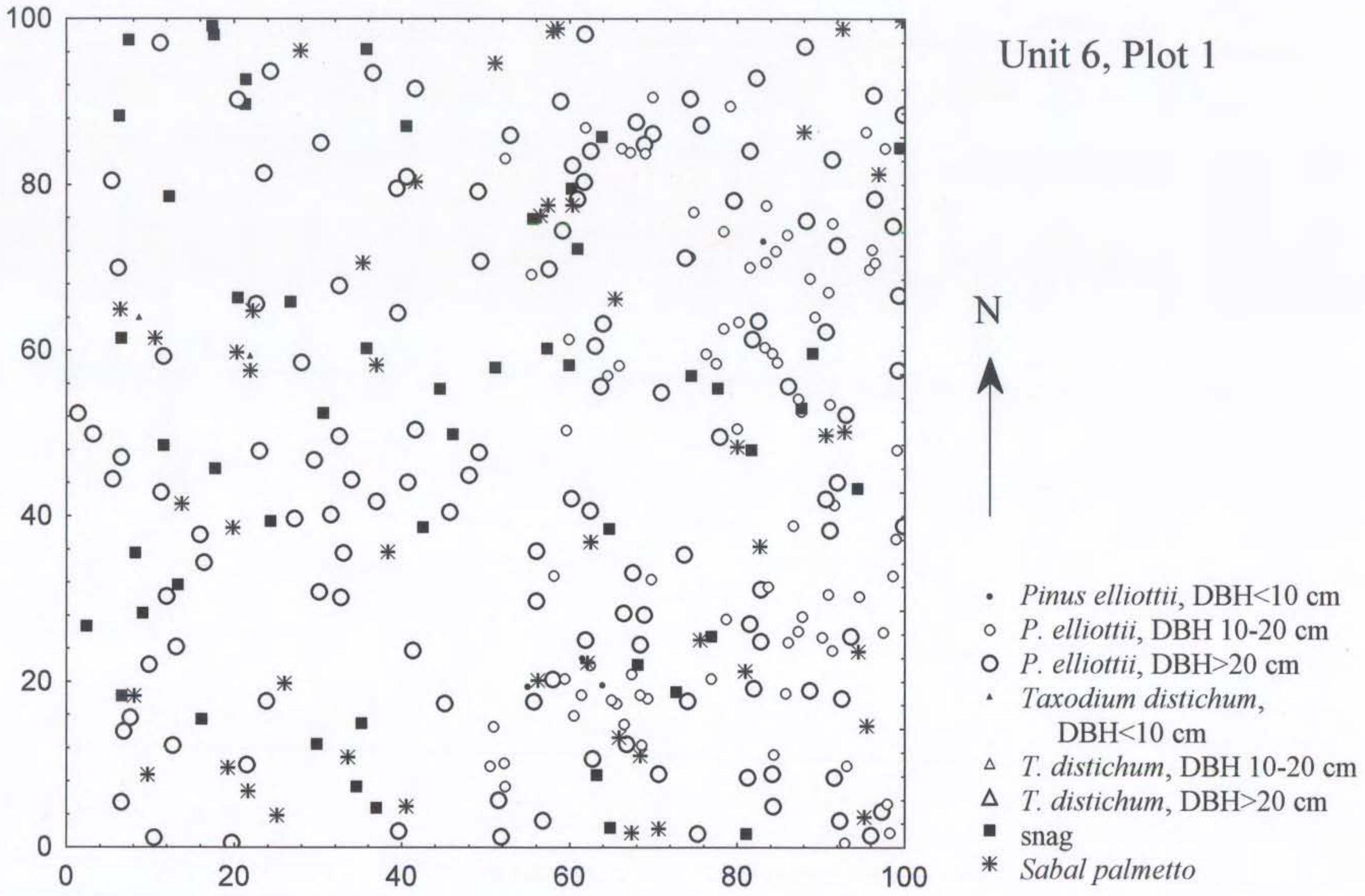
Unit 6, Plot 1
N
0
20
40
60
80
100
• Pinus elliottii, DBH<10 cm
○ P. elliottii, DBH 10-20 cm
O P. elliottii, DBH>20 cm
▴ Taxodium distichum, DBH<10 cm
△ T. distichum, DBH 10-20 cm
Δ T. distichum, DBH>20 cm
■ snag
✳ Sabal palmetto

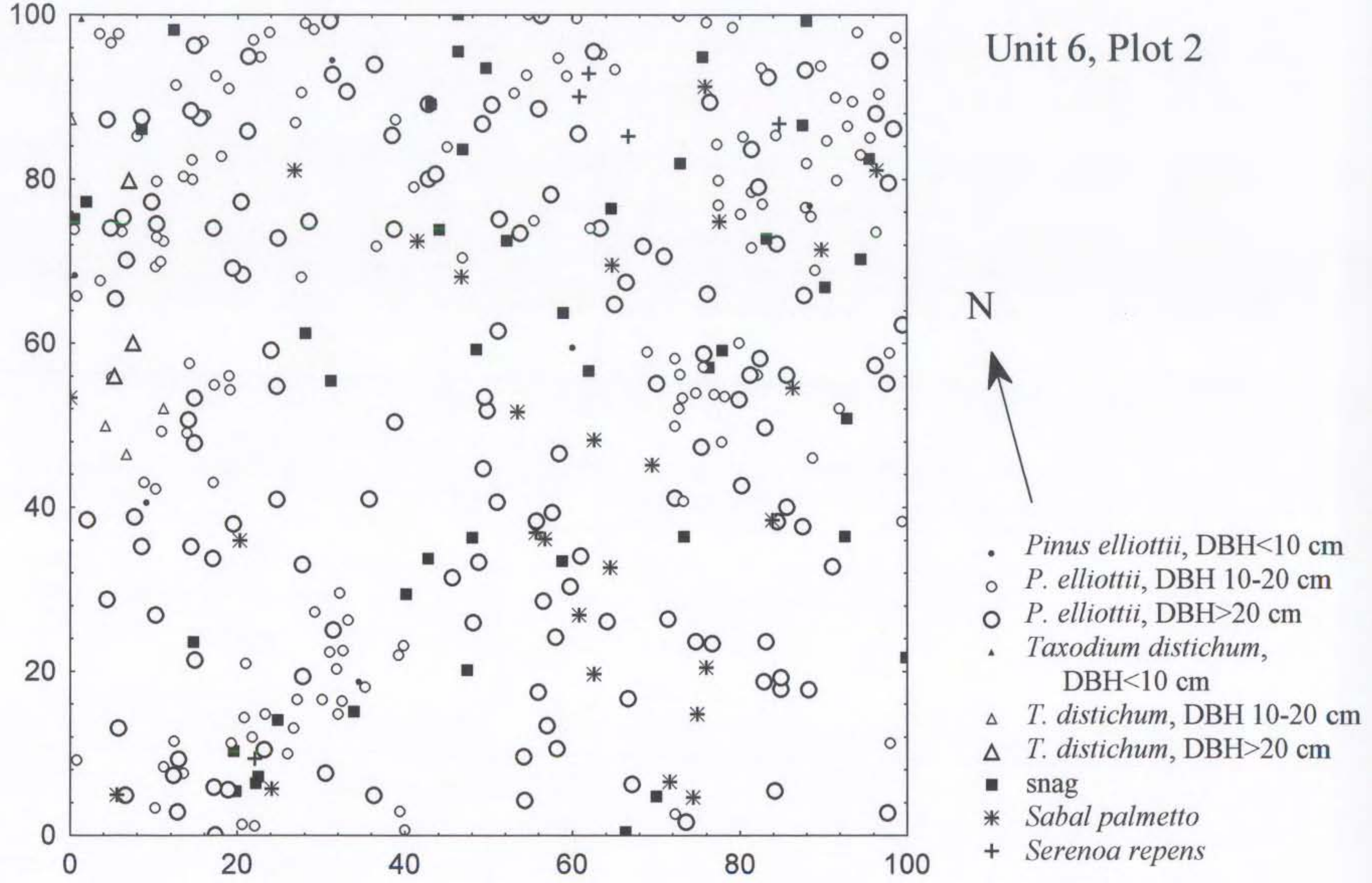
Unit 6, Plot 2
N
Pinus elliottii, DBH<10 cm
P. elliottii, DBH 10-20 cm
P. elliottii, DBH>20 cm
Taxodium distichum, DBH<10 cm
T. distichum, DBH 10-20 cm
T. distichum, DBH>20 cm
snag
Sabal palmetto
Serenoa repens
0
20
40
60
80
100

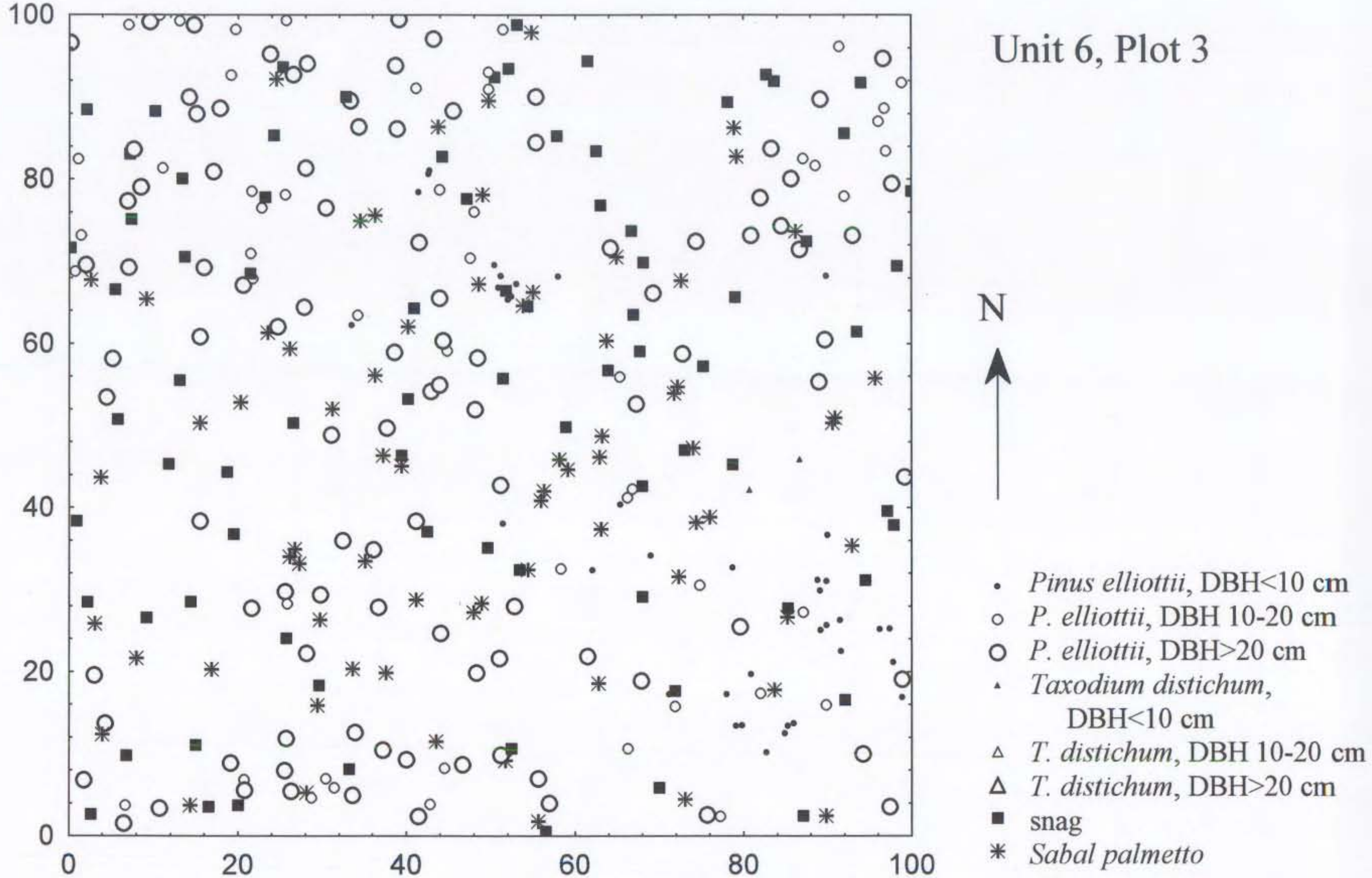
Unit 6, Plot 3
N
100
80
60
40
20
0
0
20
40
60
80
100
Pinus elliottii, DBH<10 cm
P. elliottii, DBH 10-20 cm
P. elliottii, DBH>20 cm
Taxodium distichum, DBH<10 cm
T. distichum, DBH 10-20 cm
T. distichum, DBH>20 cm
snag
Sabal palmetto

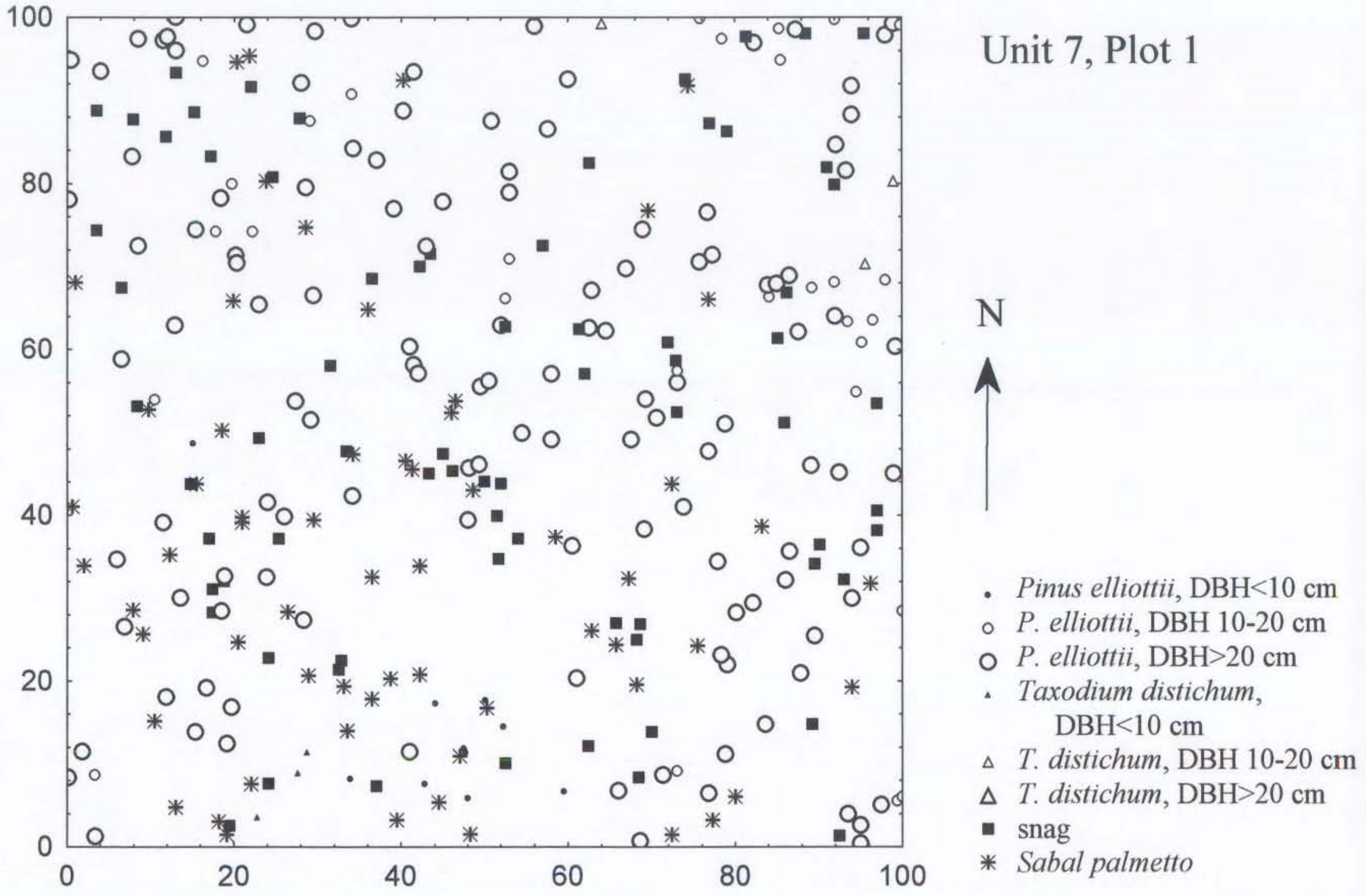
Unit 7, Plot 1
N
0
20
40
60
80
100
Pinus elliottii, DBH<10 cm
P. elliottii, DBH 10-20 cm
P. elliottii, DBH>20 cm
Taxodium distichum, DBH<10 cm
T. distichum, DBH 10-20 cm
T. distichum, DBH>20 cm
snag
Sabal palmetto

Unit 7, Plot 2

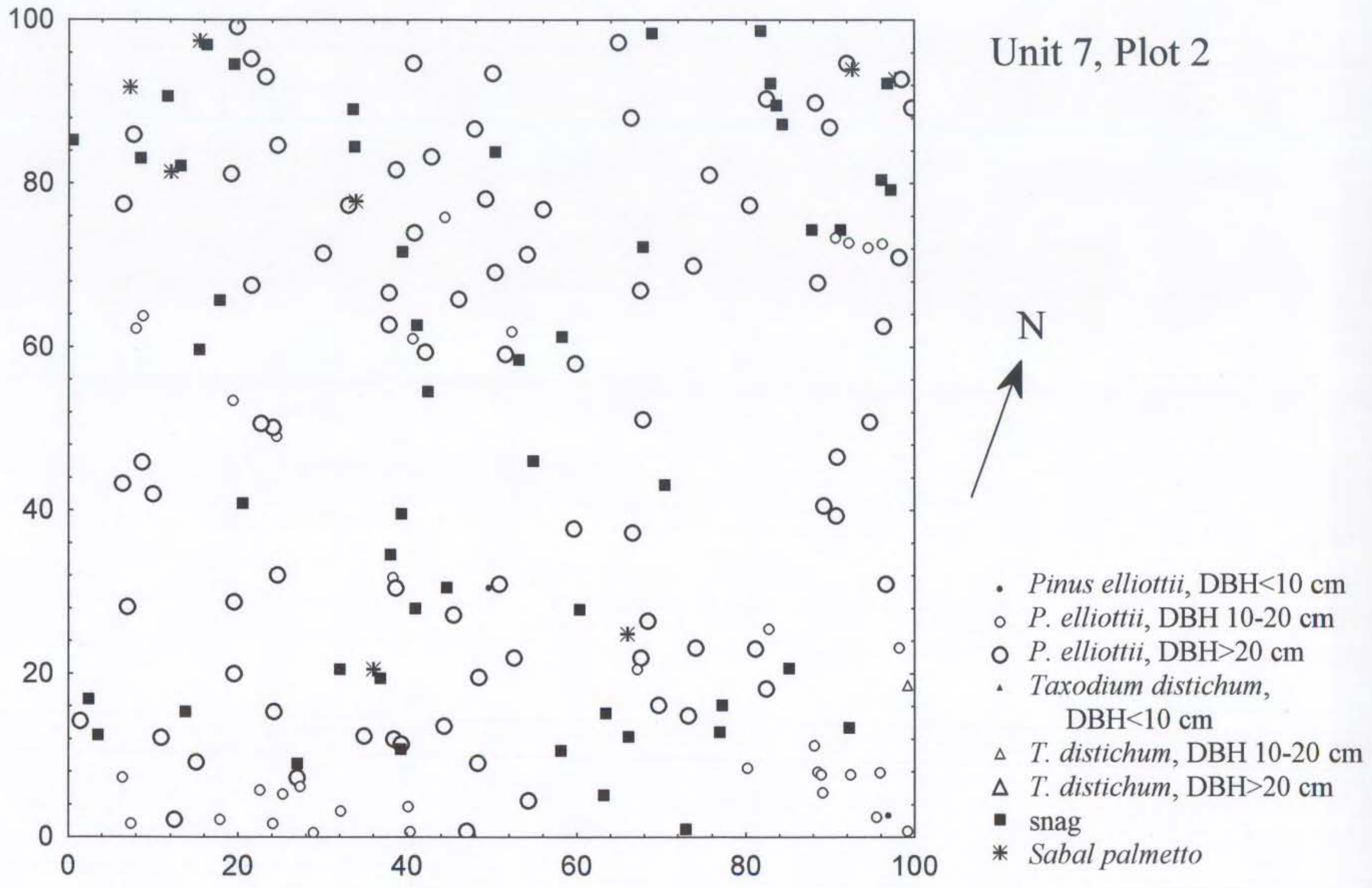
100
80
60
40
20
0
0
20
40
60
80
100
N
Pinus elliottii, DBH<10 cm
P. elliottii, DBH 10-20 cm
P. elliottii, DBH>20 cm
Taxodium distichum, DBH<10 cm
T. distichum, DBH 10-20 cm
T. distichum, DBH>20 cm
snag
Sabal palmetto

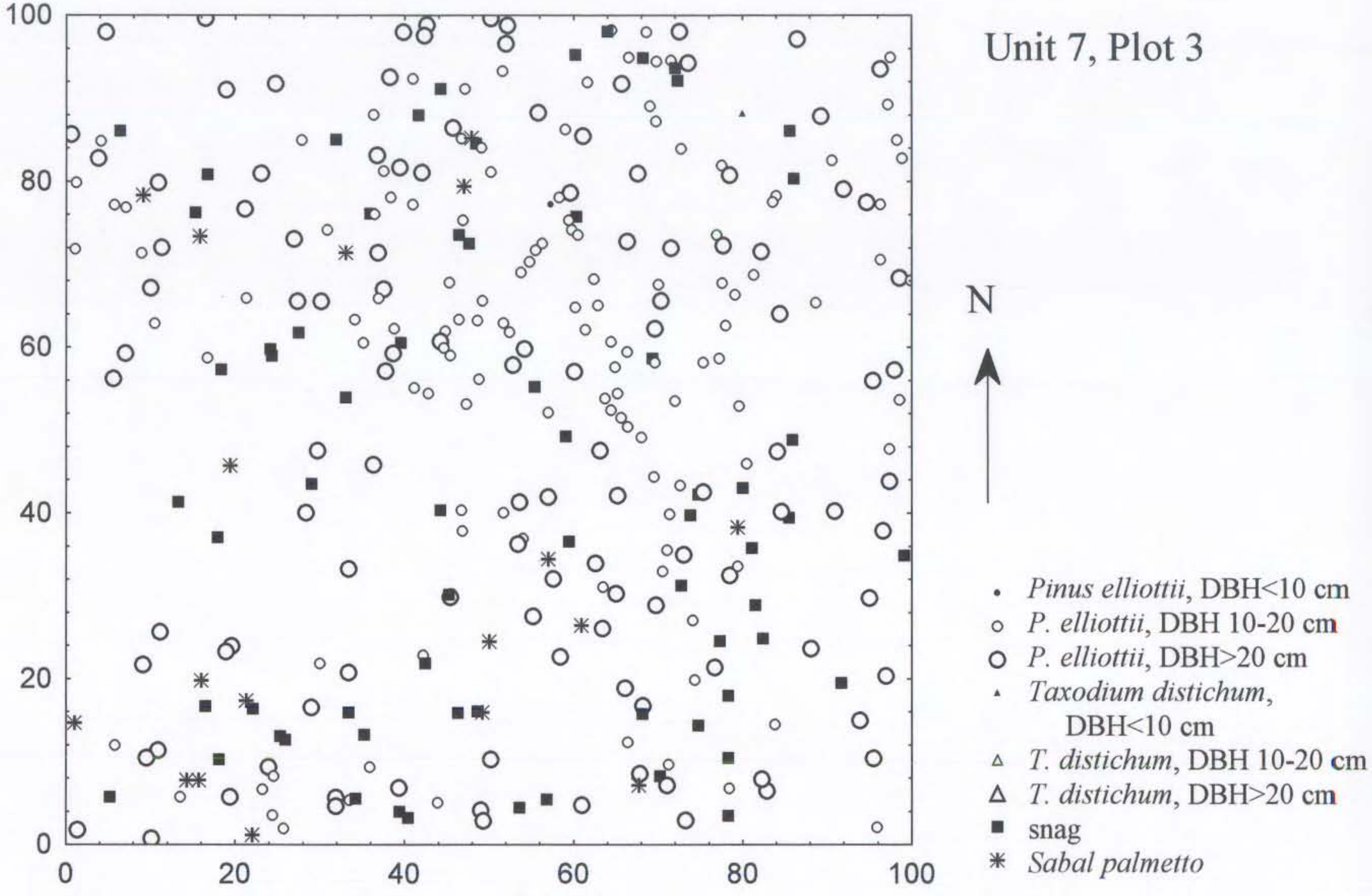
Unit 7, Plot 3
N
100
80
60
40
20
0
0
20
40
60
80
100
Pinus elliottii, DBH<10 cm
P. elliottii, DBH 10-20 cm
P. elliottii, DBH>20 cm
Taxodium distichum, DBH<10 cm
T. distichum, DBH 10-20 cm
T. distichum, DBH>20 cm
snag
Sabal palmetto

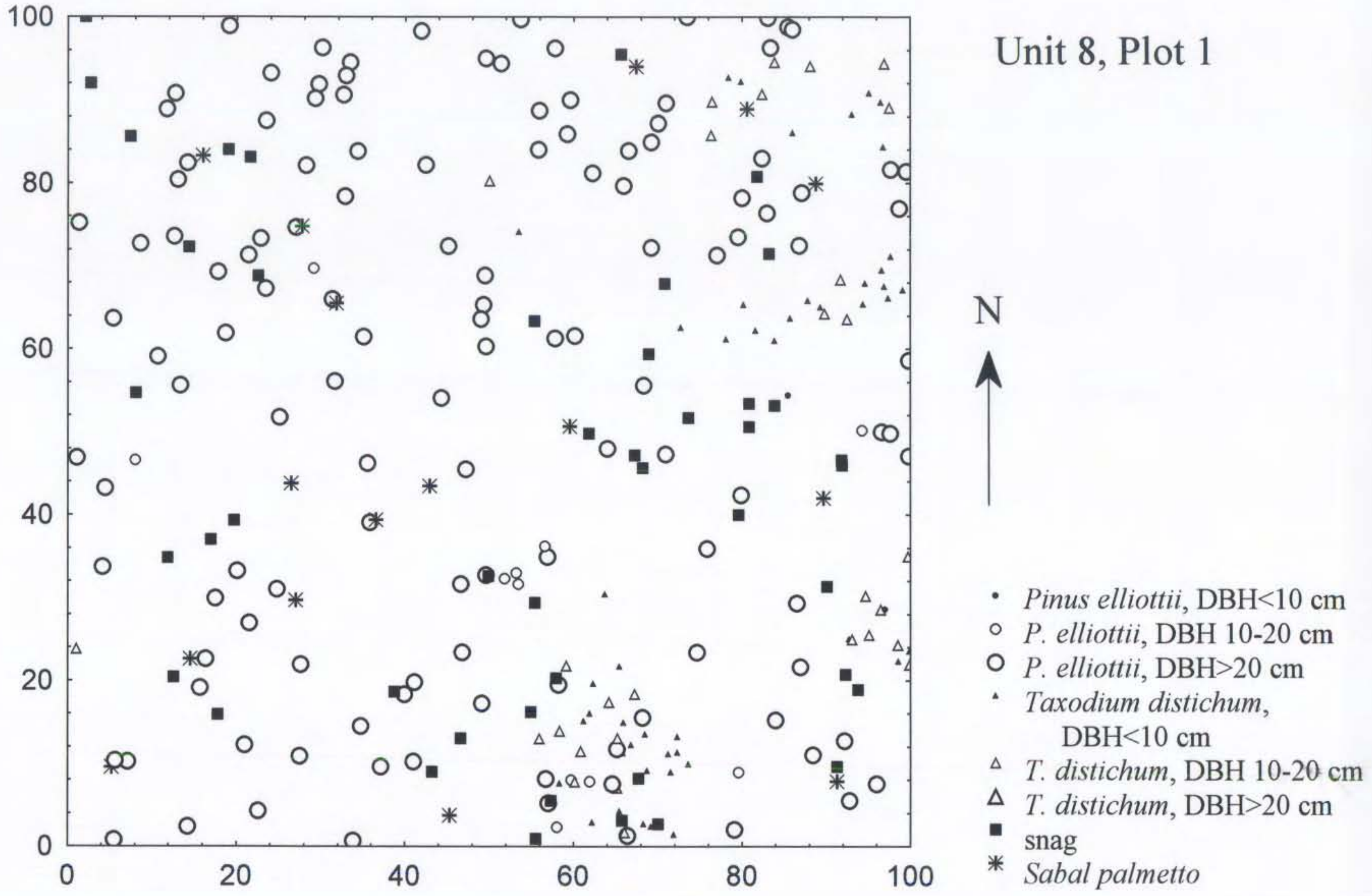

Unit 8, Plot 1
N
Pinus elliottii, DBH<10 cm
P. elliottii, DBH 10-20 cm
P. elliottii, DBH>20 cm
Taxodium distichum, DBH<10 cm
T. distichum, DBH 10-20 cm
T. distichum, DBH>20 cm
snag
Sabal palmetto
0
20
40
60
80
100

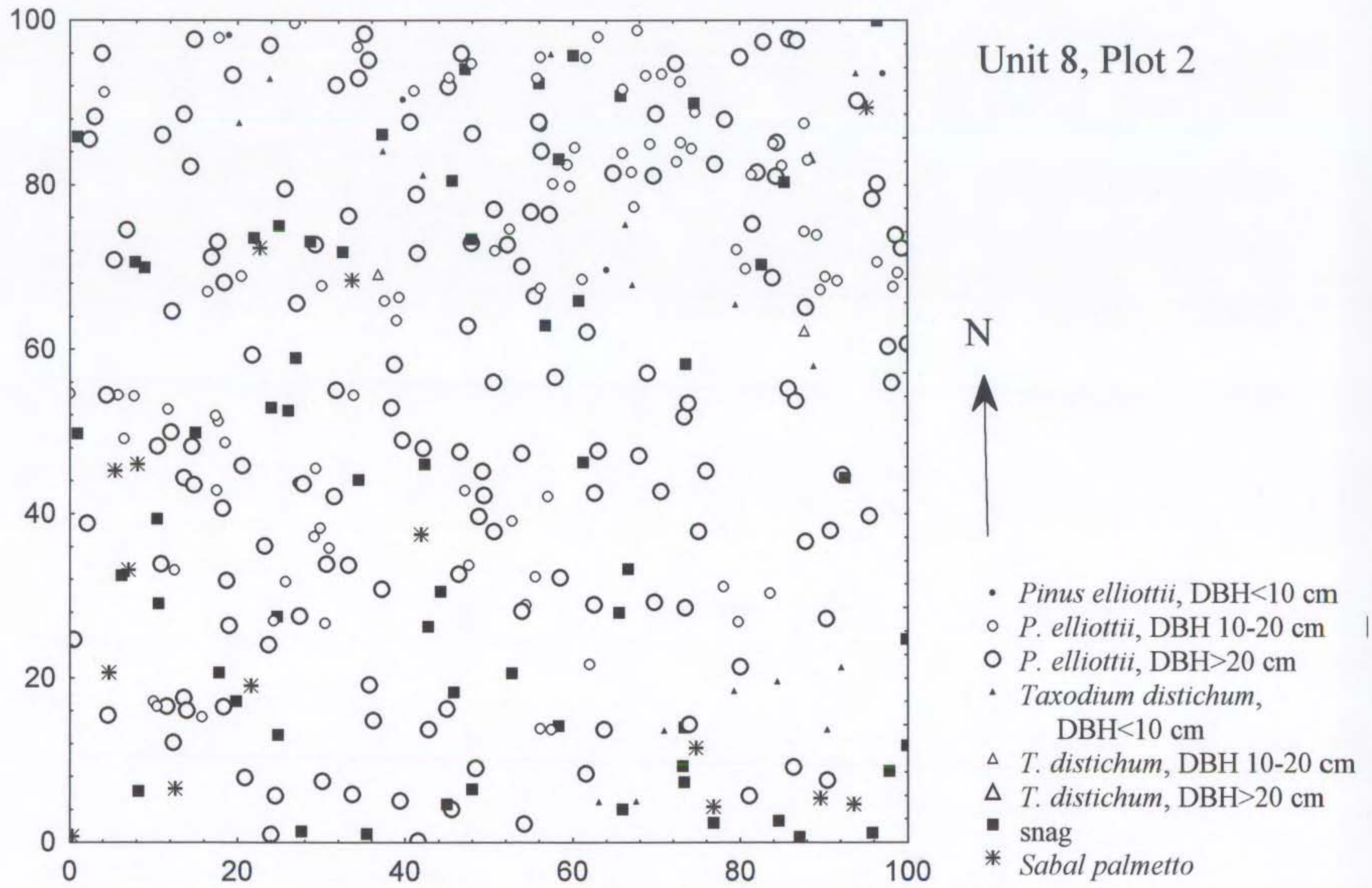

Unit 8, Plot 2
N
0
20
40
60
80
100
Pinus elliottii, DBH<10 cm
P. elliottii, DBH 10-20 cm
P. elliottii, DBH>20 cm
Taxodium distichum, DBH<10 cm
T. distichum, DBH 10-20 cm
T. distichum, DBH>20 cm
snag
Sabal palmetto

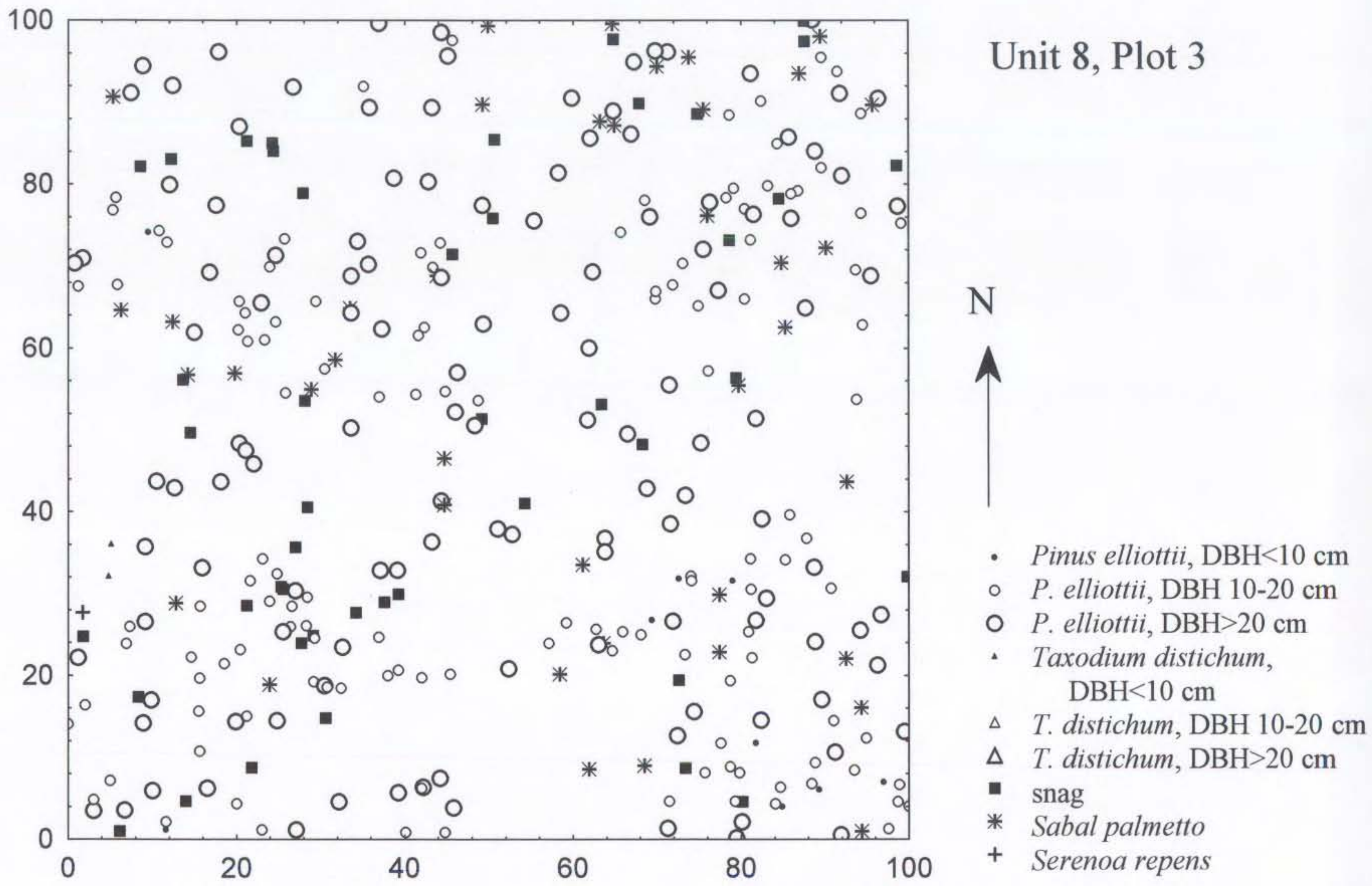
Unit 8, Plot 3
100
80
60
40
20
0
0
20
40
60
80
100
N
Pinus elliottii, DBH<10 cm
P. elliottii, DBH 10-20 cm
P. elliottii, DBH>20 cm
Taxodium distichum, DBH<10 cm
T. distichum, DBH 10-20 cm
T. distichum, DBH>20 cm
snag
Sabal palmetto
Serenoa repens

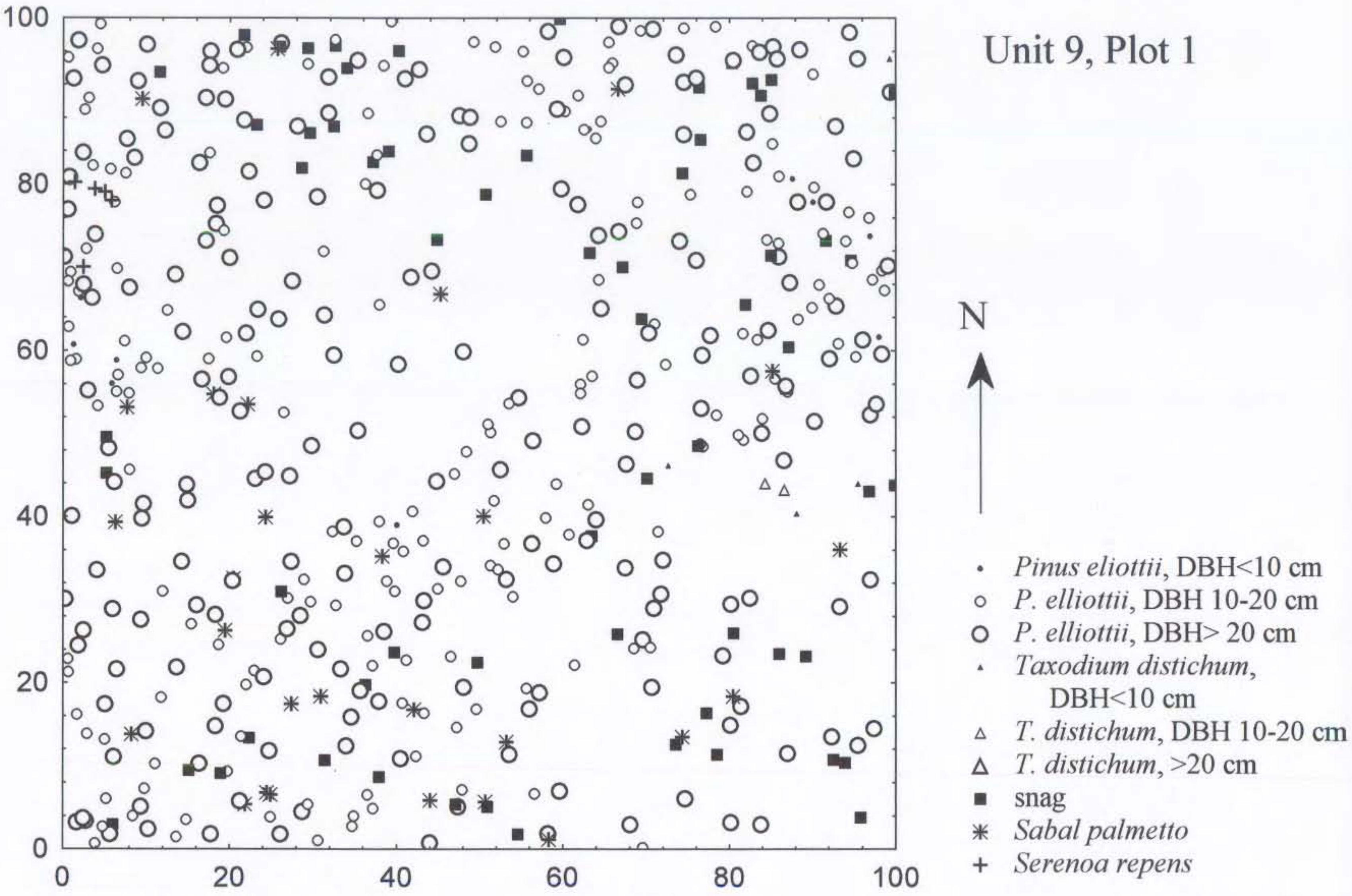
Unit 9, Plot 1
N
0
20
40
60
80
100
0
20
40
60
80
100
Pinus eliottii, DBH<10 cm
P. elliottii, DBH 10-20 cm
P. elliottii, DBH> 20 cm
Taxodium distichum, DBH<10 cm
T. distichum, DBH 10-20 cm
T. distichum, >20 cm
snag
Sabal palmetto
Serenoa repens

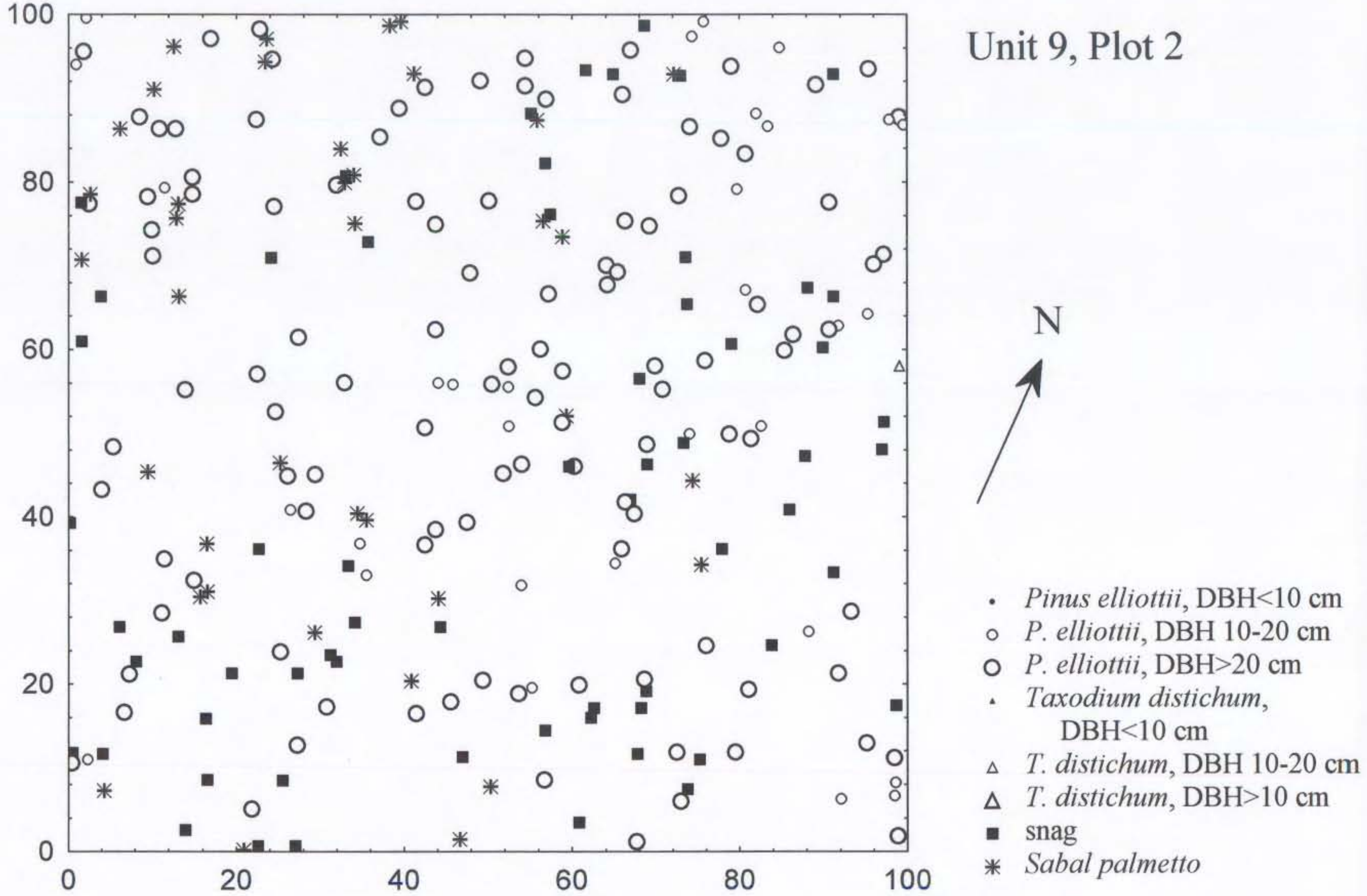

Unit 9, Plot 2
N
0
20
40
60
80
100
Pinus elliottii, DBH<10 cm
P. elliottii, DBH 10-20 cm
P. elliottii, DBH>20 cm
Taxodium distichum, DBH<10 cm
T. distichum, DBH 10-20 cm
T. distichum, DBH>10 cm
snag
Sabal palmetto

Unit 9, Plot 3

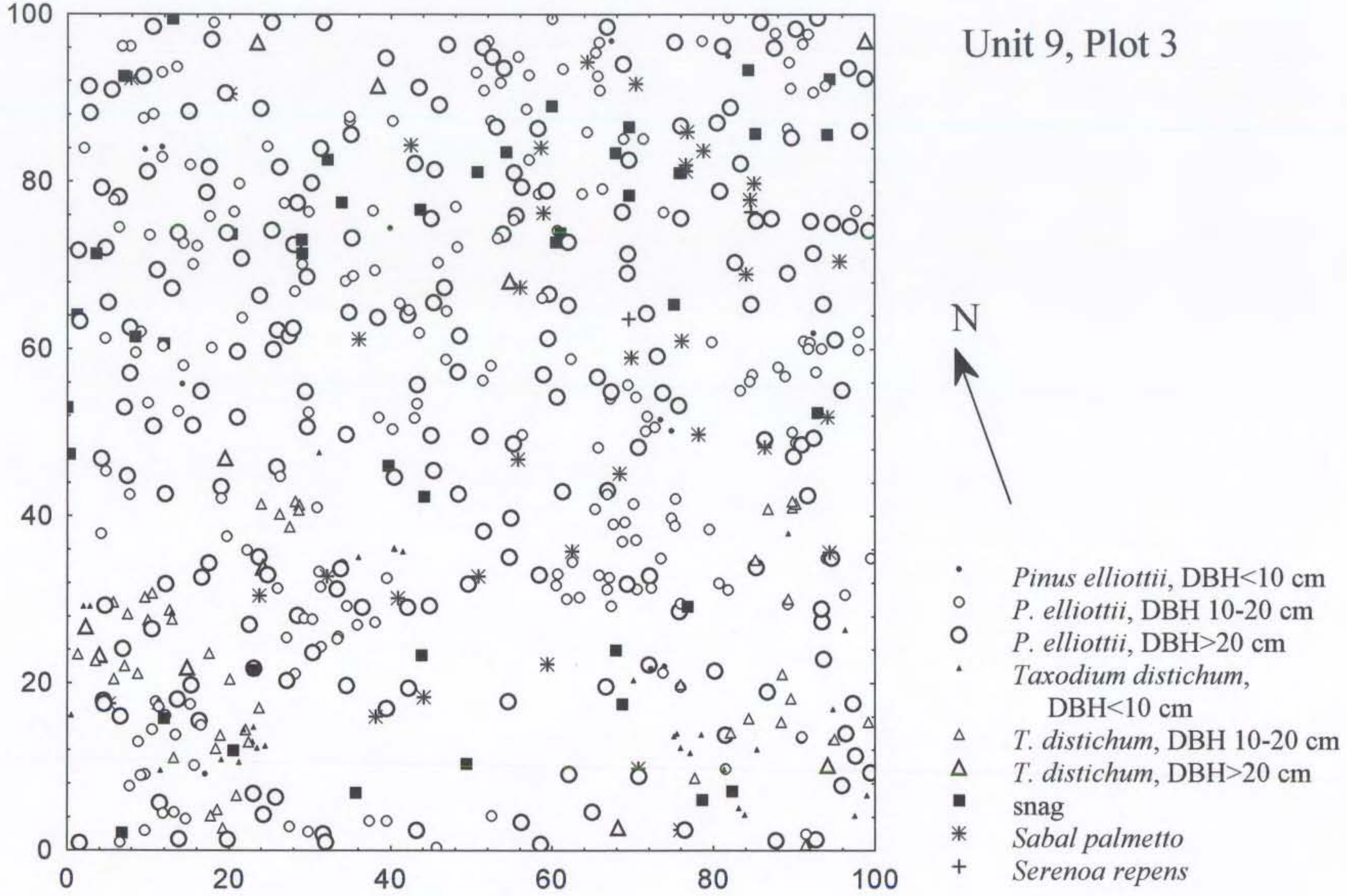

100
80
60
40
20
0
0
20
40
60
80
100
N
Pinus elliottii, DBH<10 cm
P. elliottii, DBH 10-20 cm
P. elliottii, DBH>20 cm
Taxodium distichum, DBH<10 cm
T. distichum, DBH 10-20 cm
T. distichum, DBH>20 cm
snag
Sabal palmetto
Serenoa repens

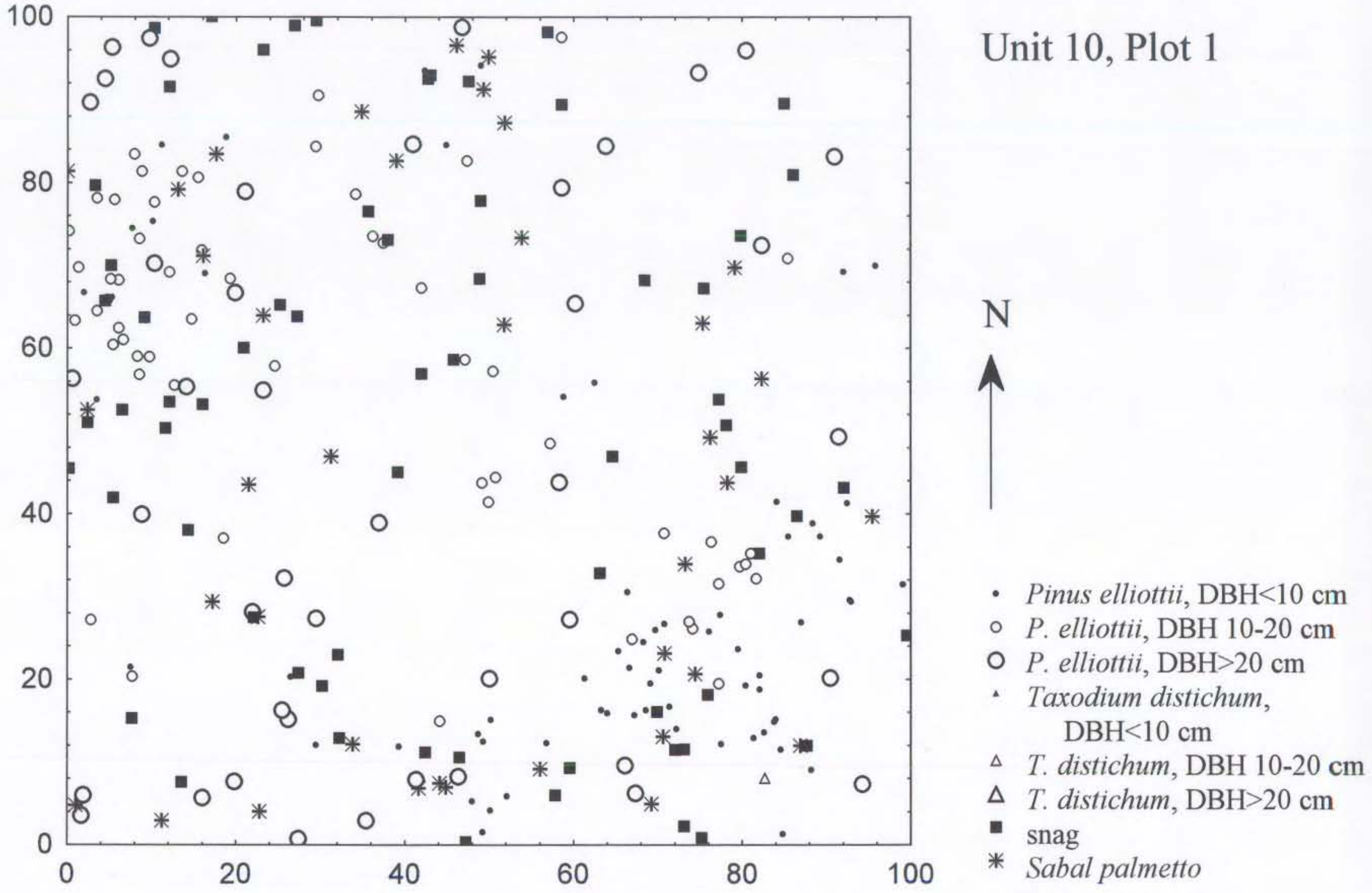
Unit 10, Plot 1
N
0
20
40
60
80
100
Pinus elliottii, DBH<10 cm
P. elliottii, DBH 10-20 cm
P. elliottii, DBH>20 cm
Taxodium distichum, DBH<10 cm
T. distichum, DBH 10-20 cm
T. distichum, DBH>20 cm
snag
Sabal palmetto

Unit 10, Plot 2

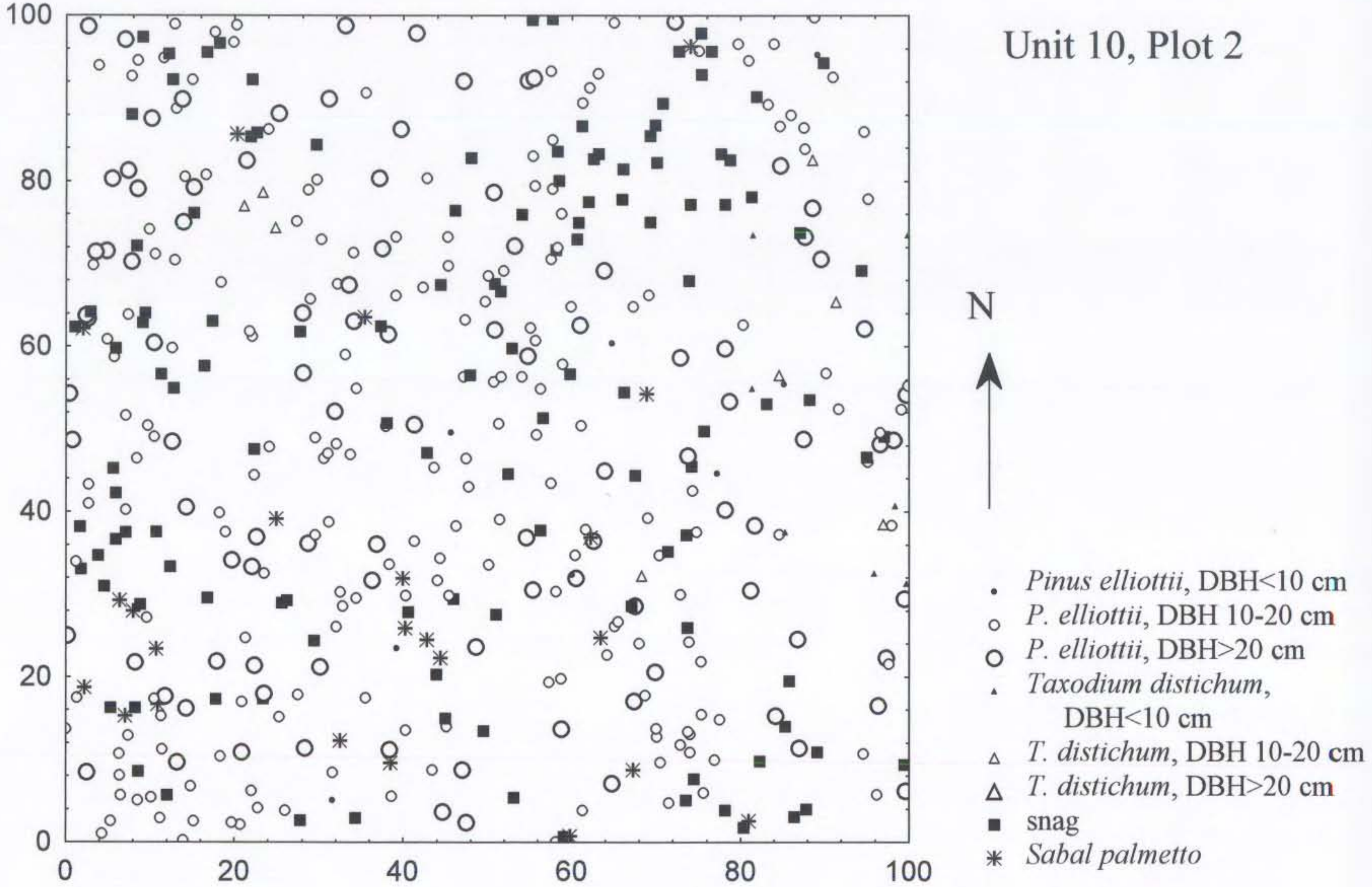

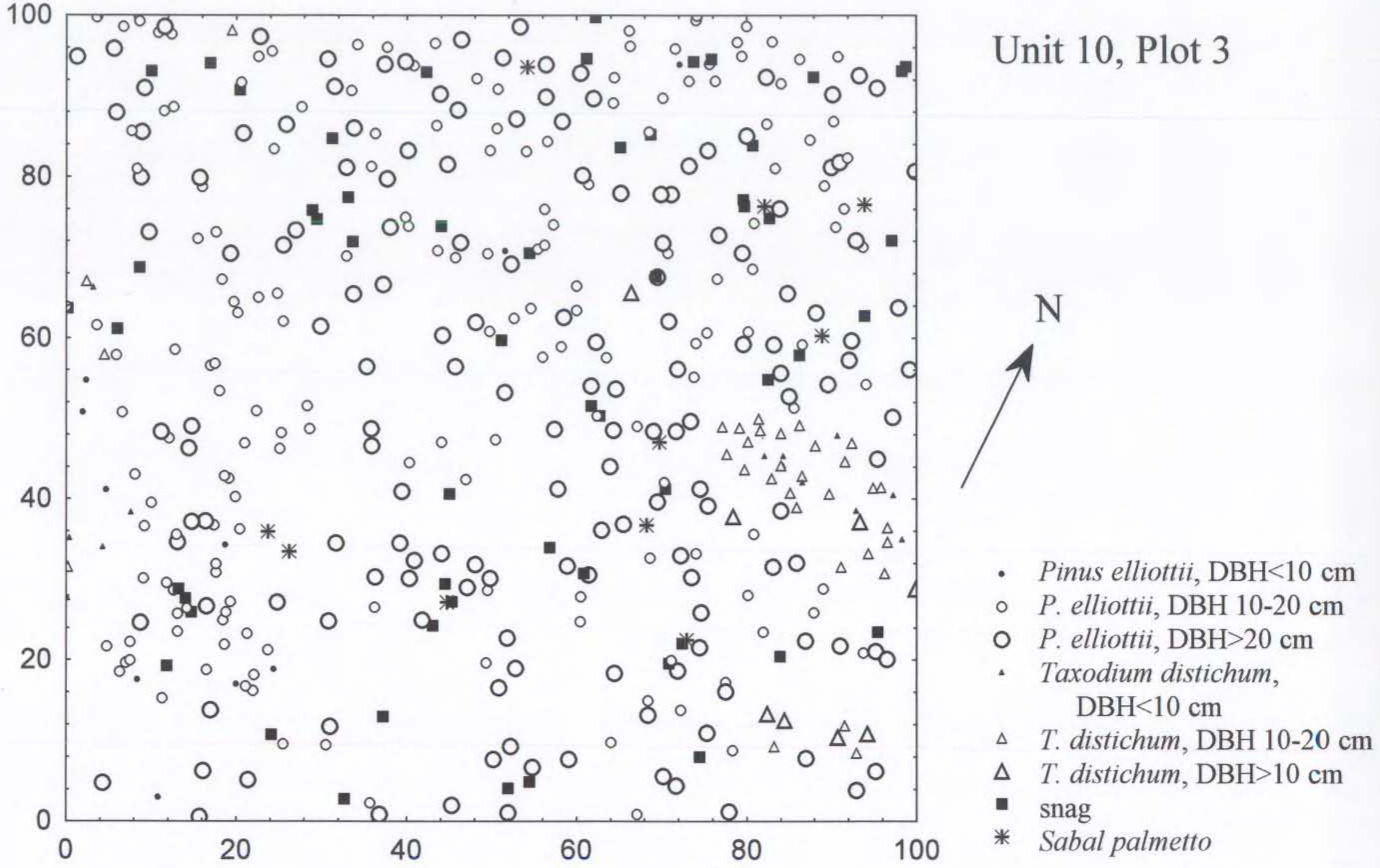
Unit 10, Plot 3
N
0
20
40
60
80
100
Pinus elliottii, DBH<10 cm
P. elliottii, DBH 10-20 cm
P. elliottii, DBH>20 cm
Taxodium distichum, DBH<10 cm
T. distichum, DBH 10-20 cm
T. distichum, DBH>10 cm
snag
Sabal palmetto

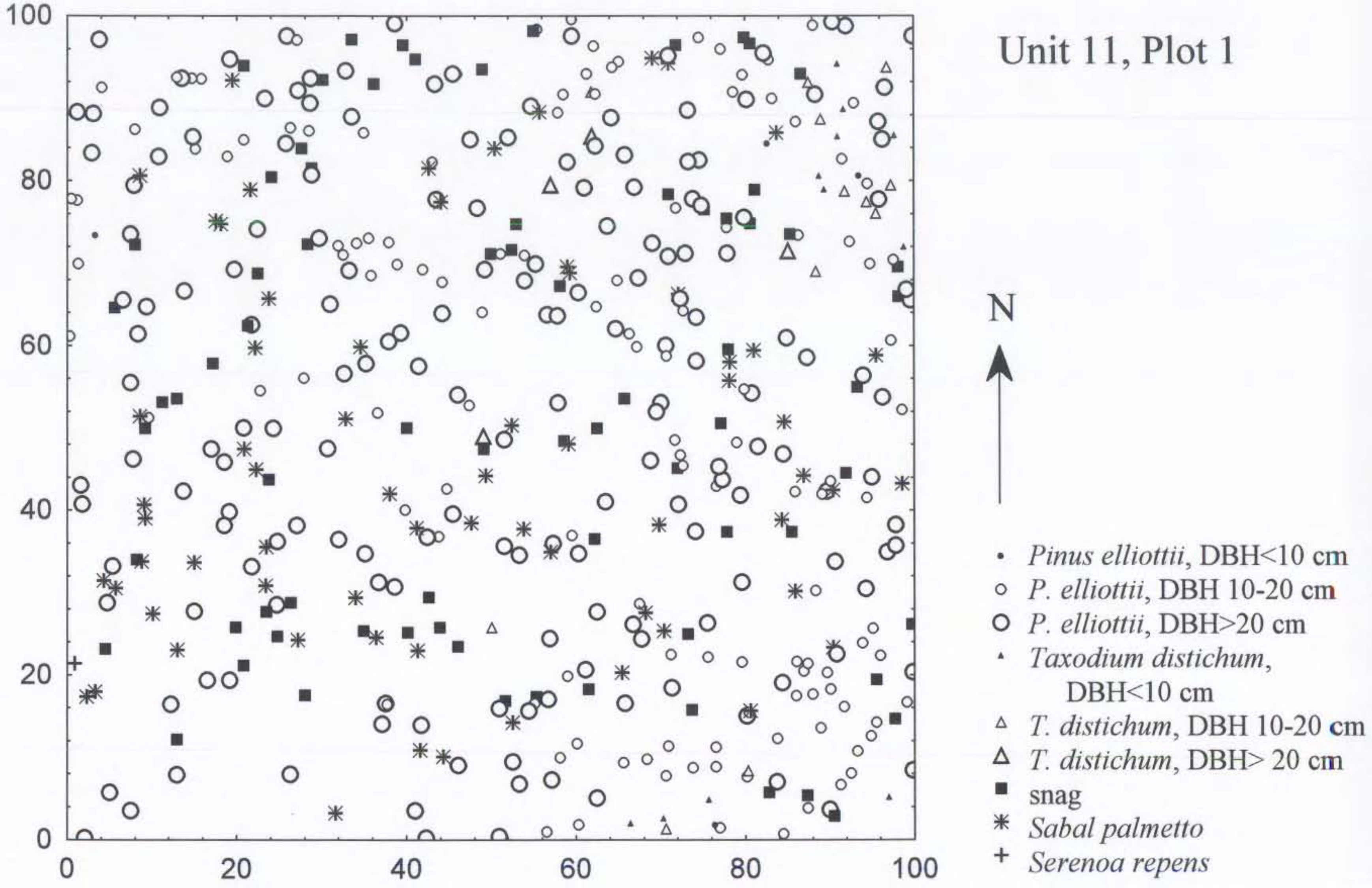
Unit 11, Plot 1
N
Pinus elliottii, DBH<10 cm
P. elliottii, DBH 10-20 cm
P. elliottii, DBH>20 cm
Taxodium distichum, DBH<10 cm
T. distichum, DBH 10-20 cm
T. distichum, DBH> 20 cm
snag
Sabal palmetto
Serenoa repens
0
20
40
60
80
100

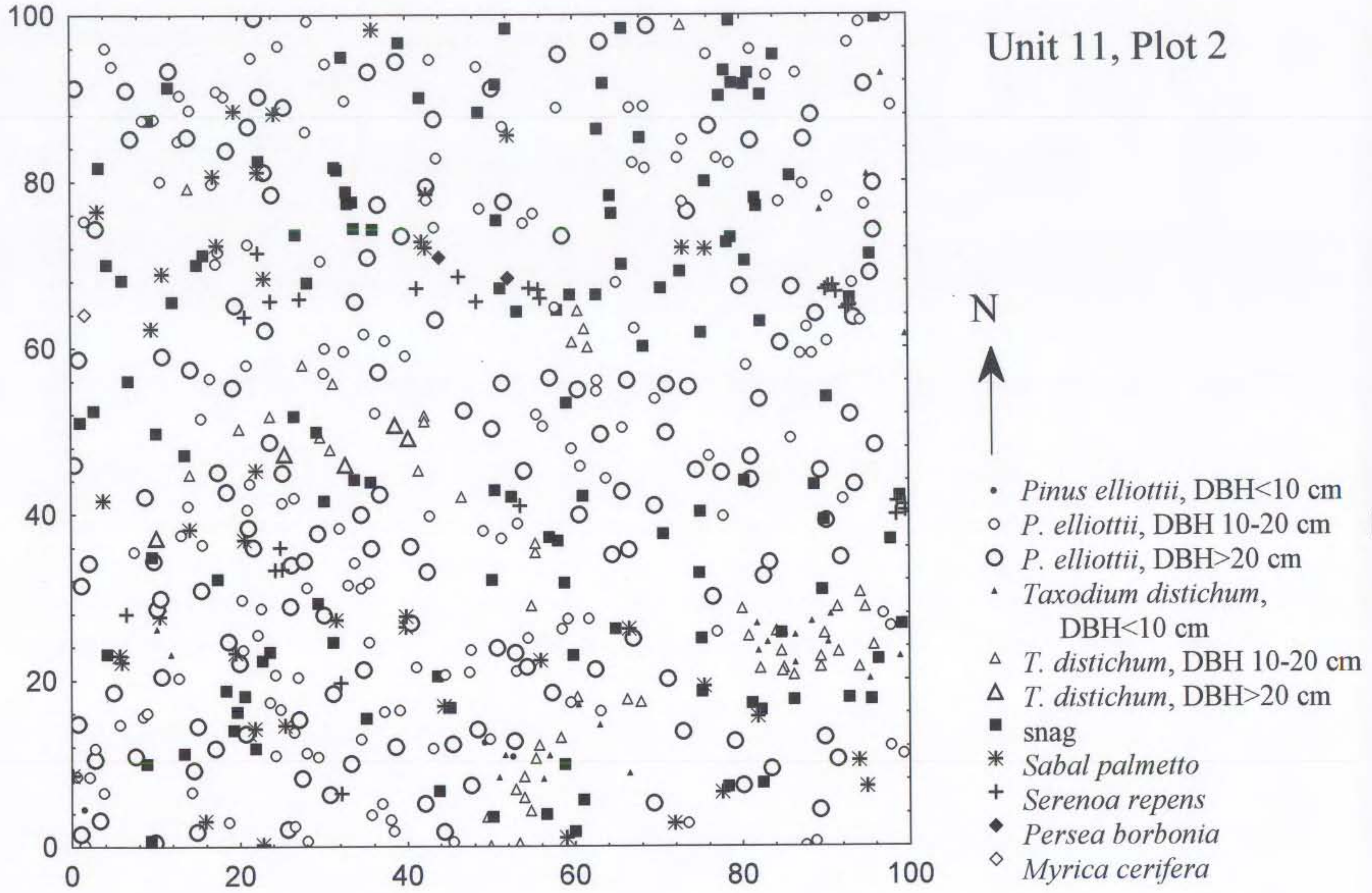
Unit 11, Plot 2
N
0
20
40
60
80
100
0
20
40
60
80
100
Pinus elliottii, DBH<10 cm
P. elliottii, DBH 10-20 cm
P. elliottii, DBH>20 cm
Taxodium distichum, DBH<10 cm
T. distichum, DBH 10-20 cm
T. distichum, DBH>20 cm
snag
Sabal palmetto
Serenoa repens
Persea borbonia
Myrica cerifera

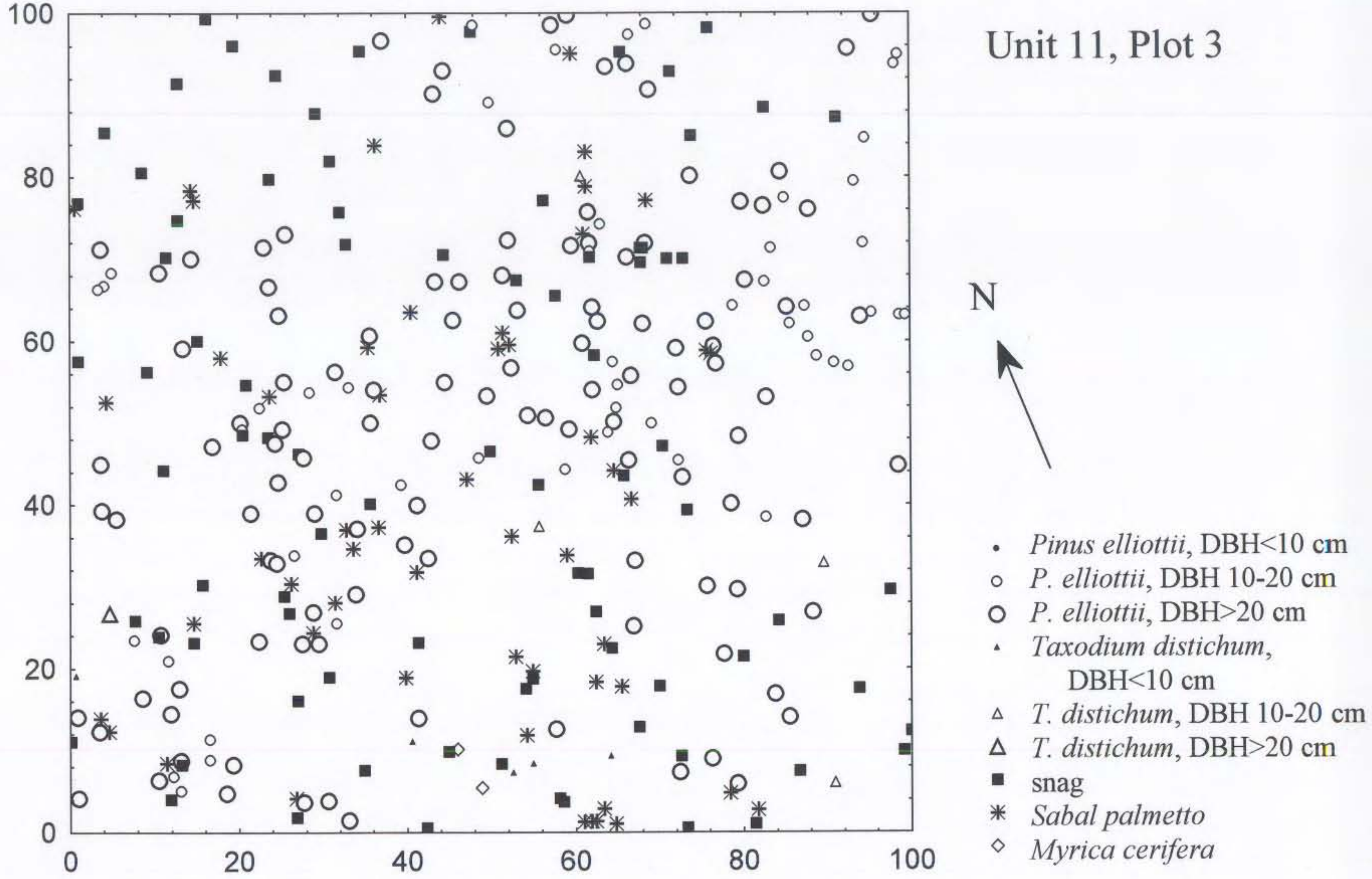

Unit 11, Plot 3
N
0
20
40
60
80
100
Pinus elliottii, DBH<10 cm
P. elliottii, DBH 10-20 cm
P. elliottii, DBH>20 cm
Taxodium distichum, DBH<10 cm
T. distichum, DBH 10-20 cm
T. distichum, DBH>20 cm
snag
Sabal palmetto
Myrica cerifera

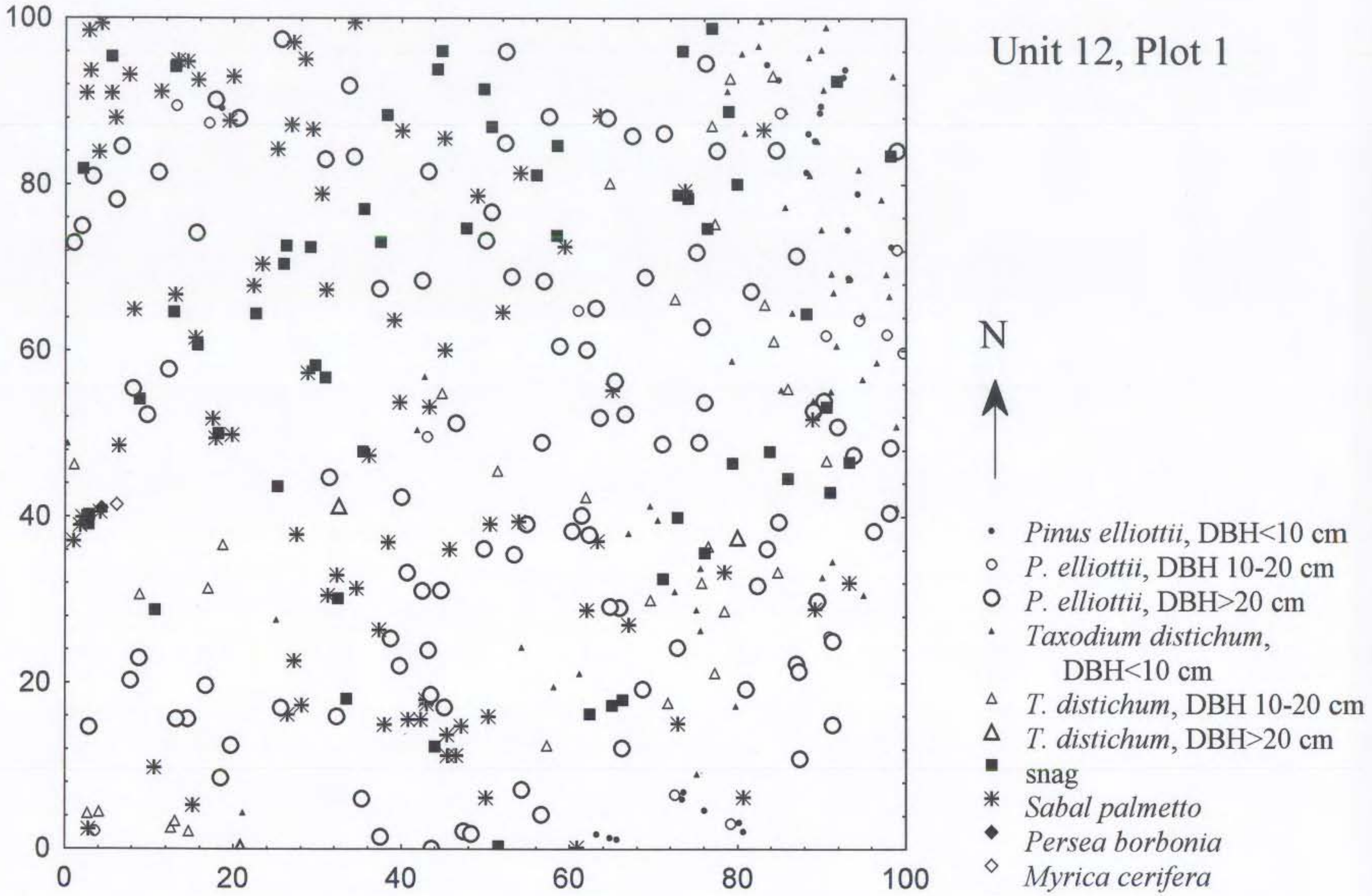

Unit 12, Plot 1
N
0
20
40
60
80
100
Pinus elliottii, DBH<10 cm
P. elliottii, DBH 10-20 cm
P. elliottii, DBH>20 cm
Taxodium distichum, DBH<10 cm
T. distichum, DBH 10-20 cm
T. distichum, DBH>20 cm
snag
Sabal palmetto
Persea borbonia
Myrica cerifera

Unit 12, Plot 2

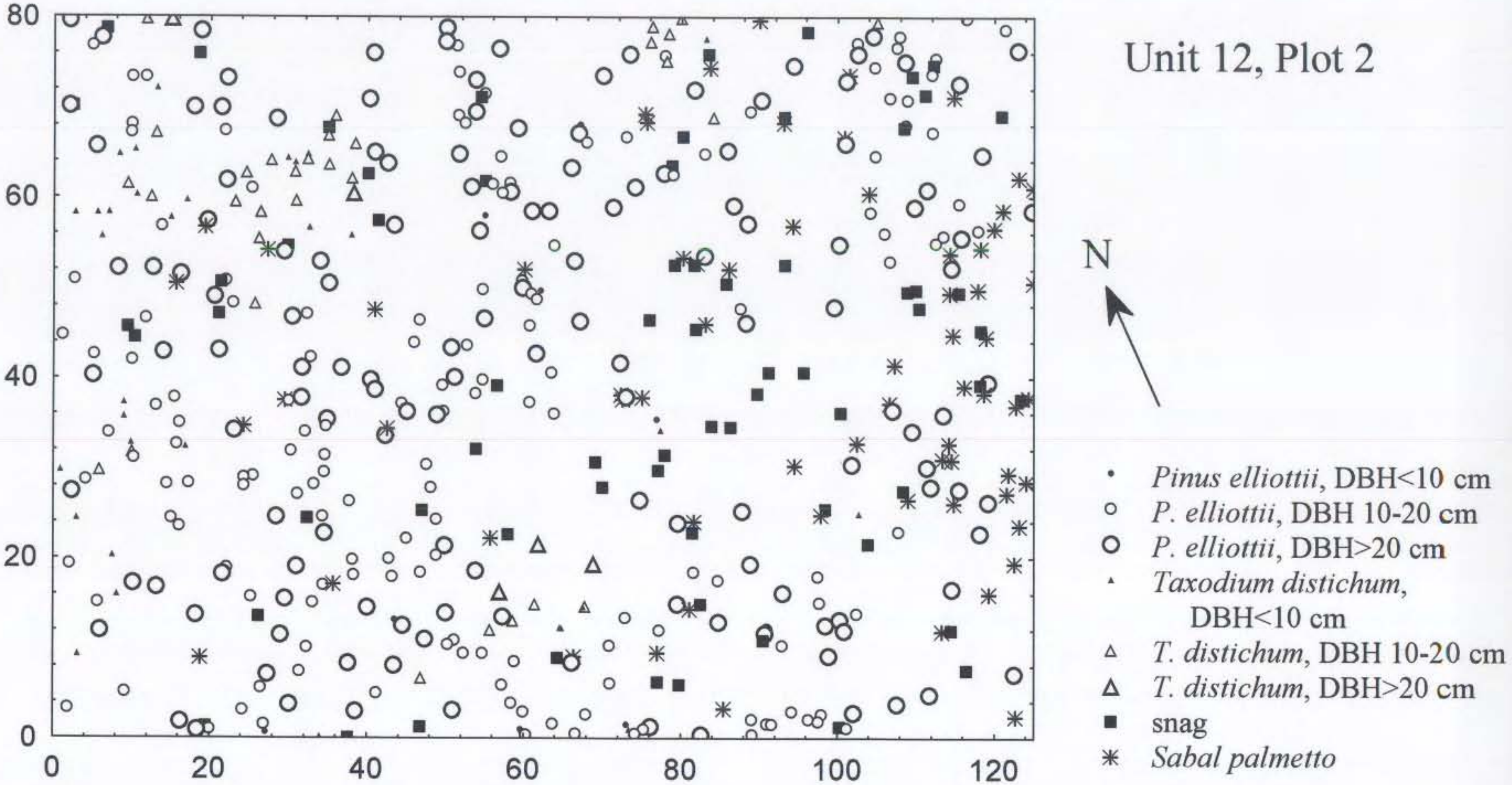

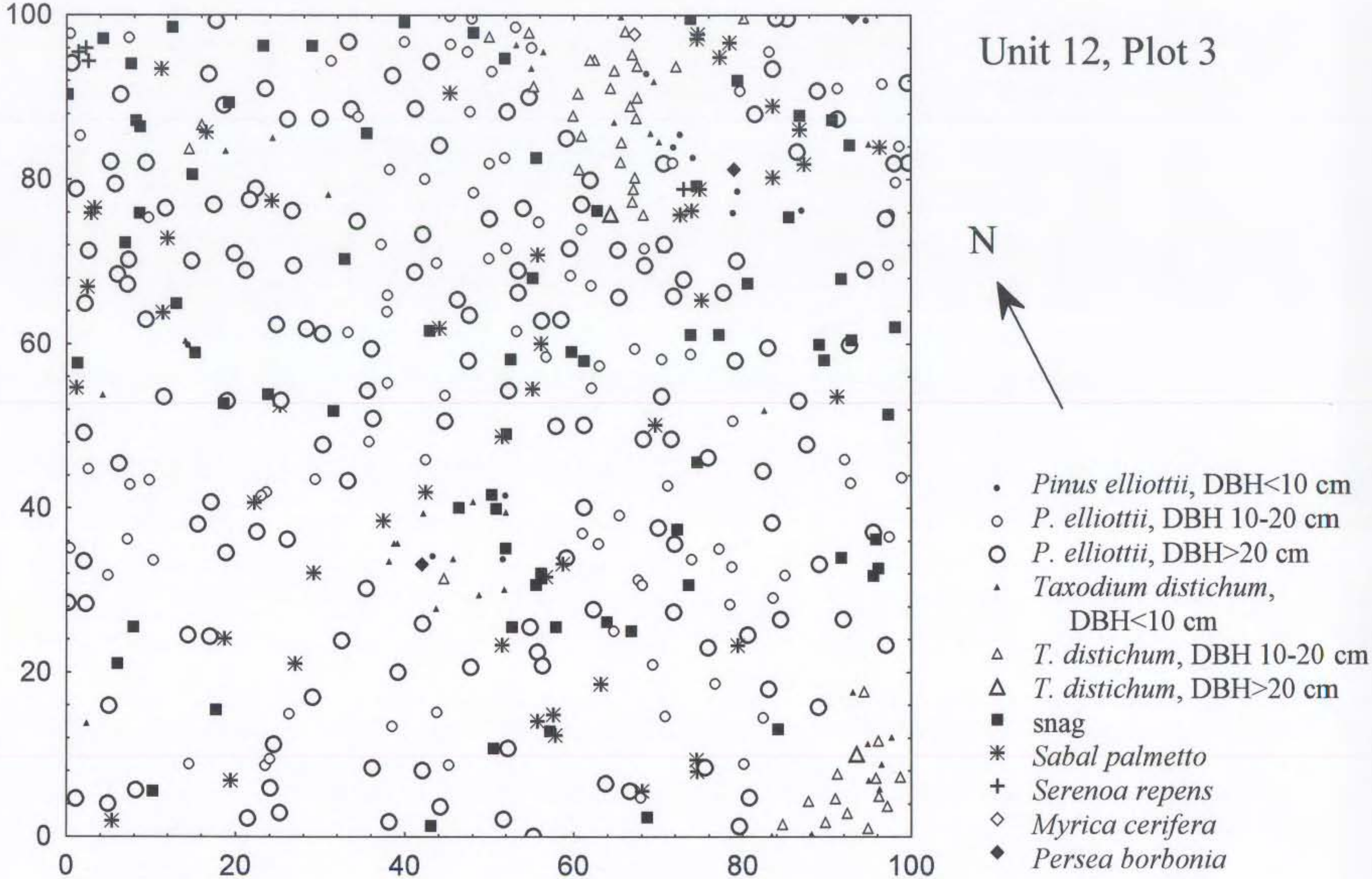
Unit 12, Plot 3
N
0
20
40
60
80
100
Pinus elliottii, DBH<10 cm
P. elliottii, DBH 10-20 cm
P. elliottii, DBH>20 cm
Taxodium distichum, DBH<10 cm
T. distichum, DBH 10-20 cm
T. distichum, DBH>20 cm
snag
Sabal palmetto
Serenoa repens
Myrica cerifera
Persea borbonia

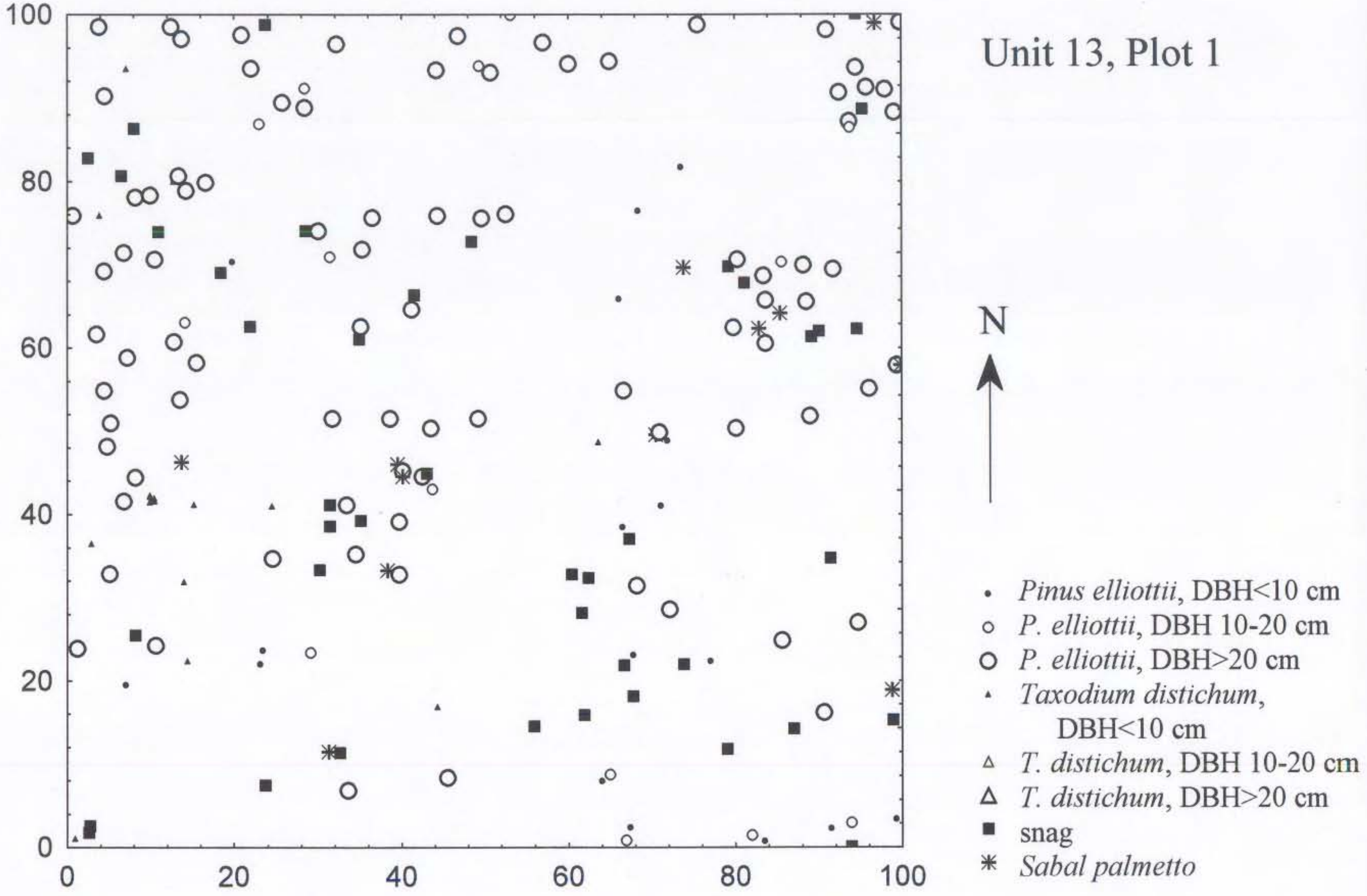
Unit 13, Plot 1
N
100
80
60
40
20
0
0
20
40
60
80
100
Pinus elliottii, DBH<10 cm
P. elliottii, DBH 10-20 cm
P. elliottii, DBH>20 cm
Taxodium distichum, DBH<10 cm
T. distichum, DBH 10-20 cm
T. distichum, DBH>20 cm
snag
Sabal palmetto

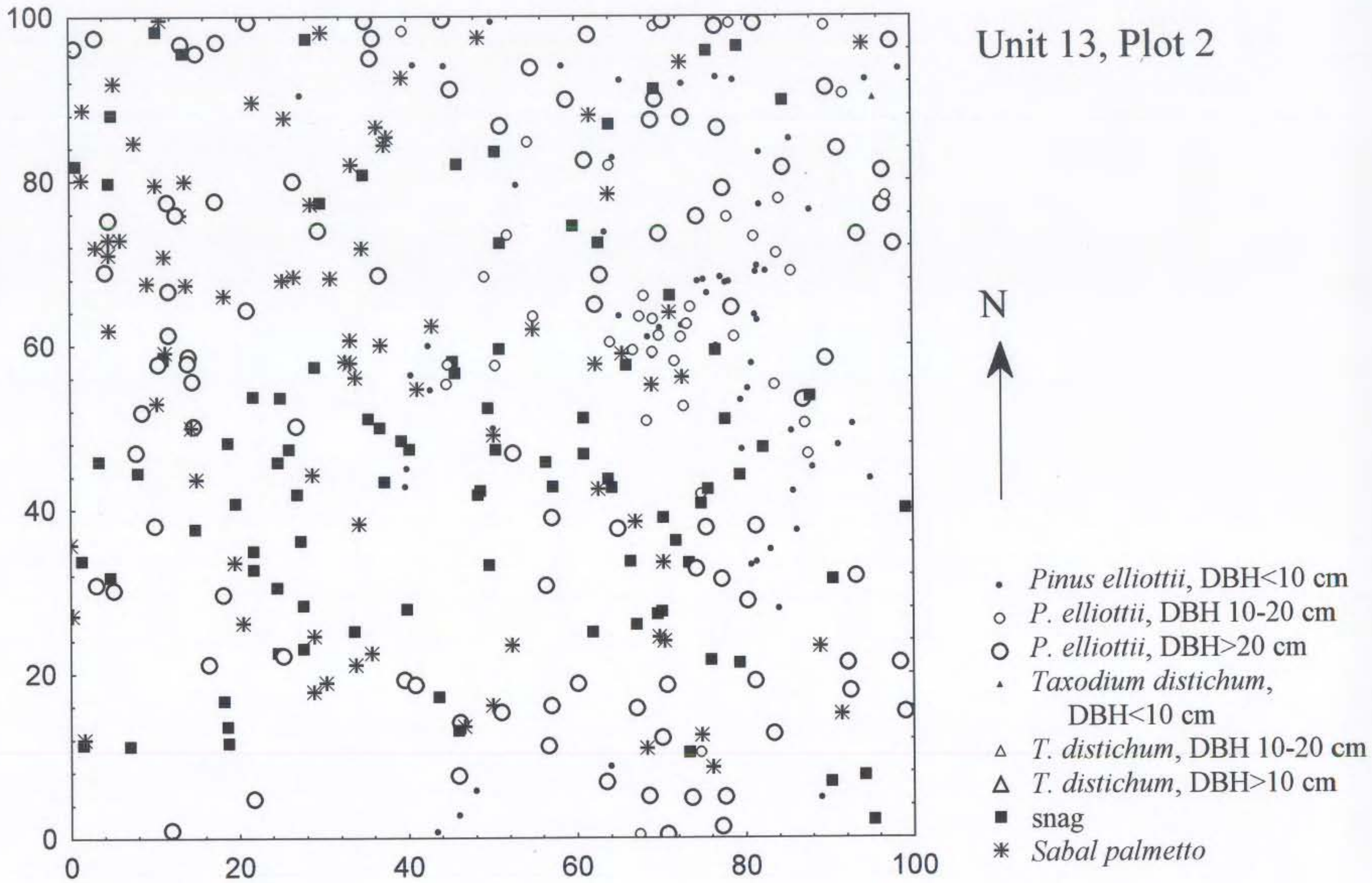

Unit 13, Plot 2
N
0
20
40
60
80
100
Pinus elliottii, DBH<10 cm
P. elliottii, DBH 10-20 cm
P. elliottii, DBH>20 cm
Taxodium distichum, DBH<10 cm
T. distichum, DBH 10-20 cm
T. distichum, DBH>10 cm
snag
Sabal palmetto

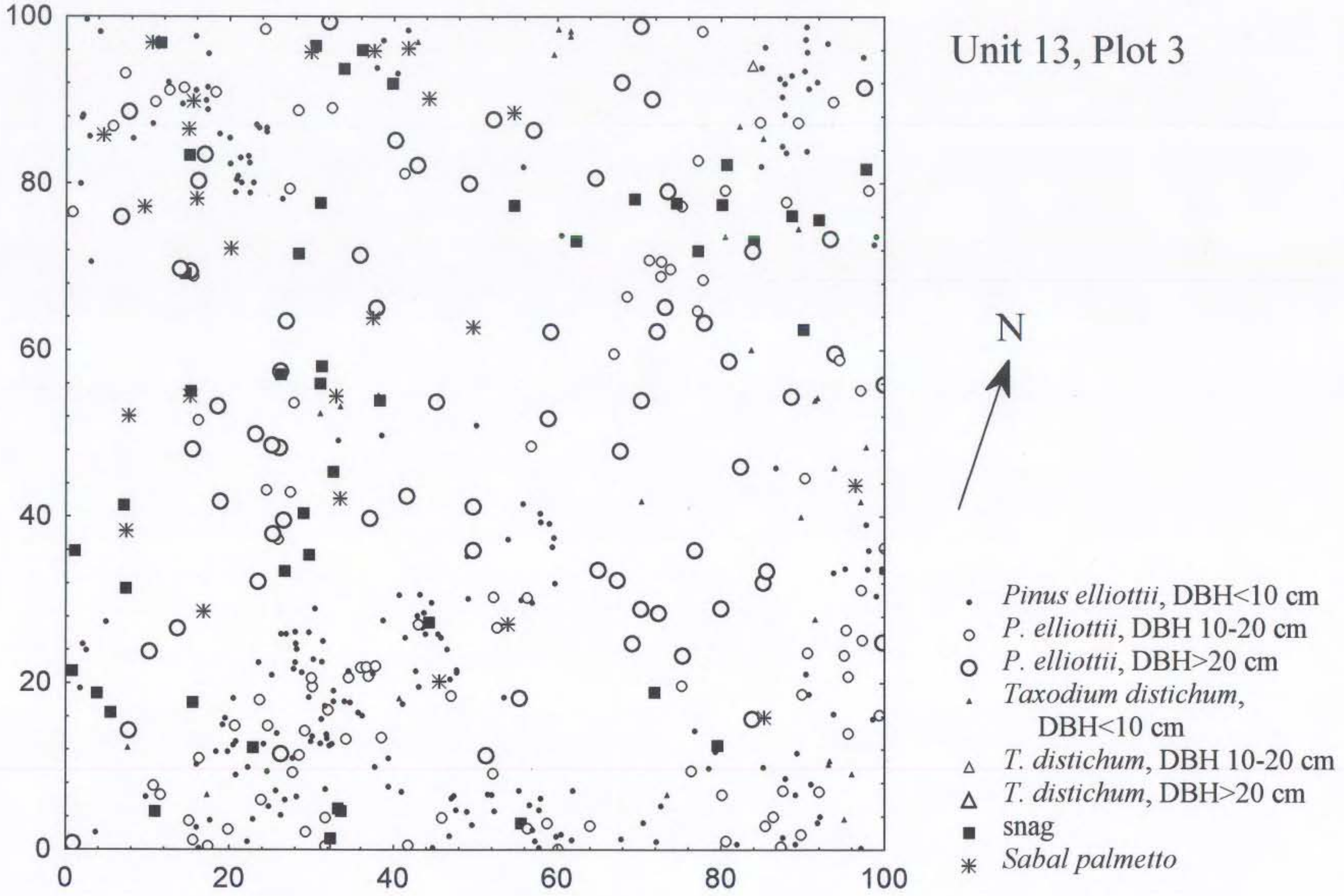
Unit 13, Plot 3
N
0
20
40
60
80
100
Pinus elliottii, DBH<10 cm
P. elliottii, DBH 10-20 cm
P. elliottii, DBH>20 cm
Taxodium distichum, DBH<10 cm
T. distichum, DBH 10-20 cm
T. distichum, DBH>20 cm
snag
Sabal palmetto

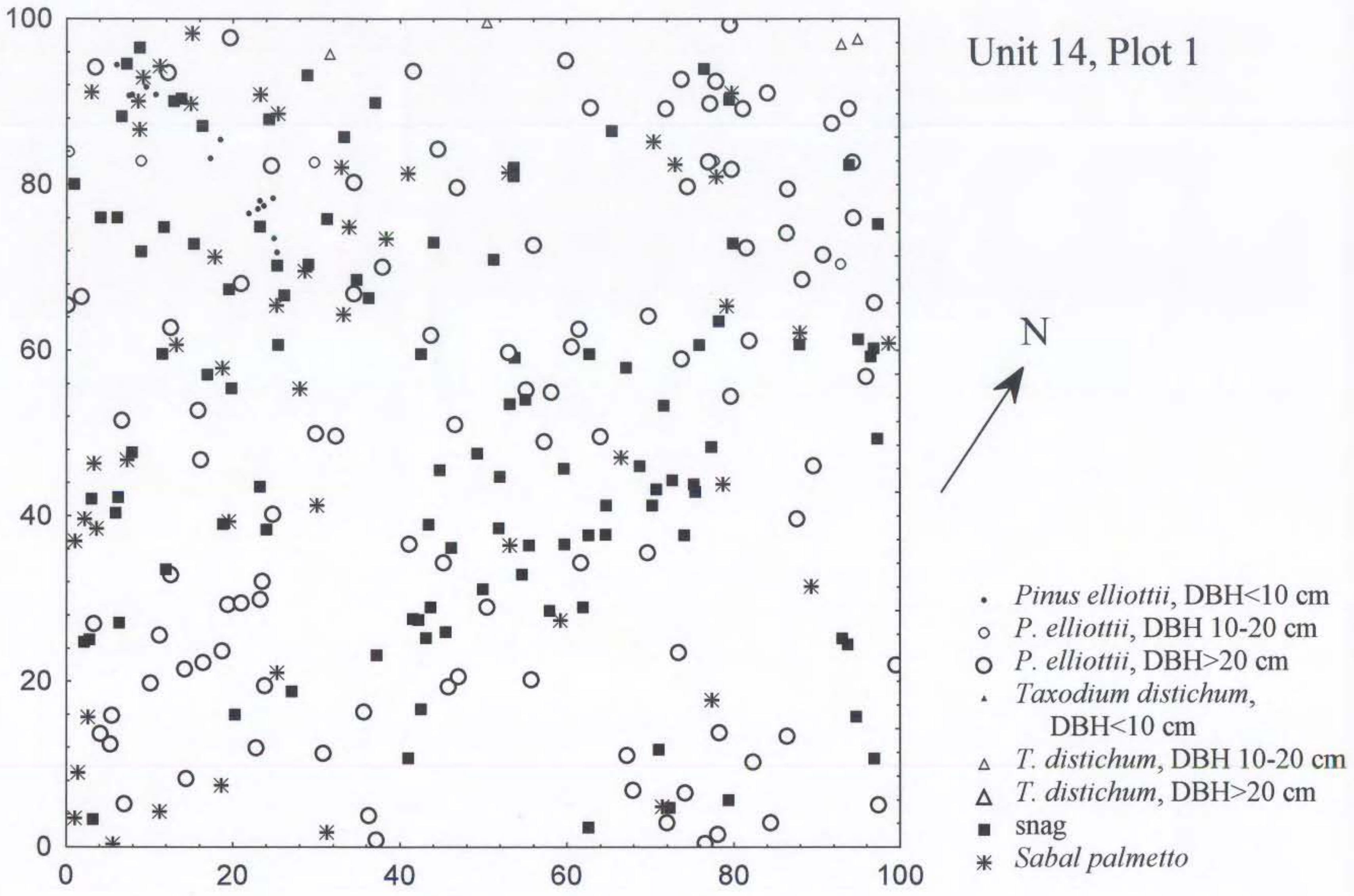
Unit 14, Plot 1
N
0
20
40
60
80
100
Pinus elliottii, DBH<10 cm
P. elliottii, DBH 10-20 cm
P. elliottii, DBH>20 cm
Taxodium distichum, DBH<10 cm
T. distichum, DBH 10-20 cm
T. distichum, DBH>20 cm
snag
Sabal palmetto

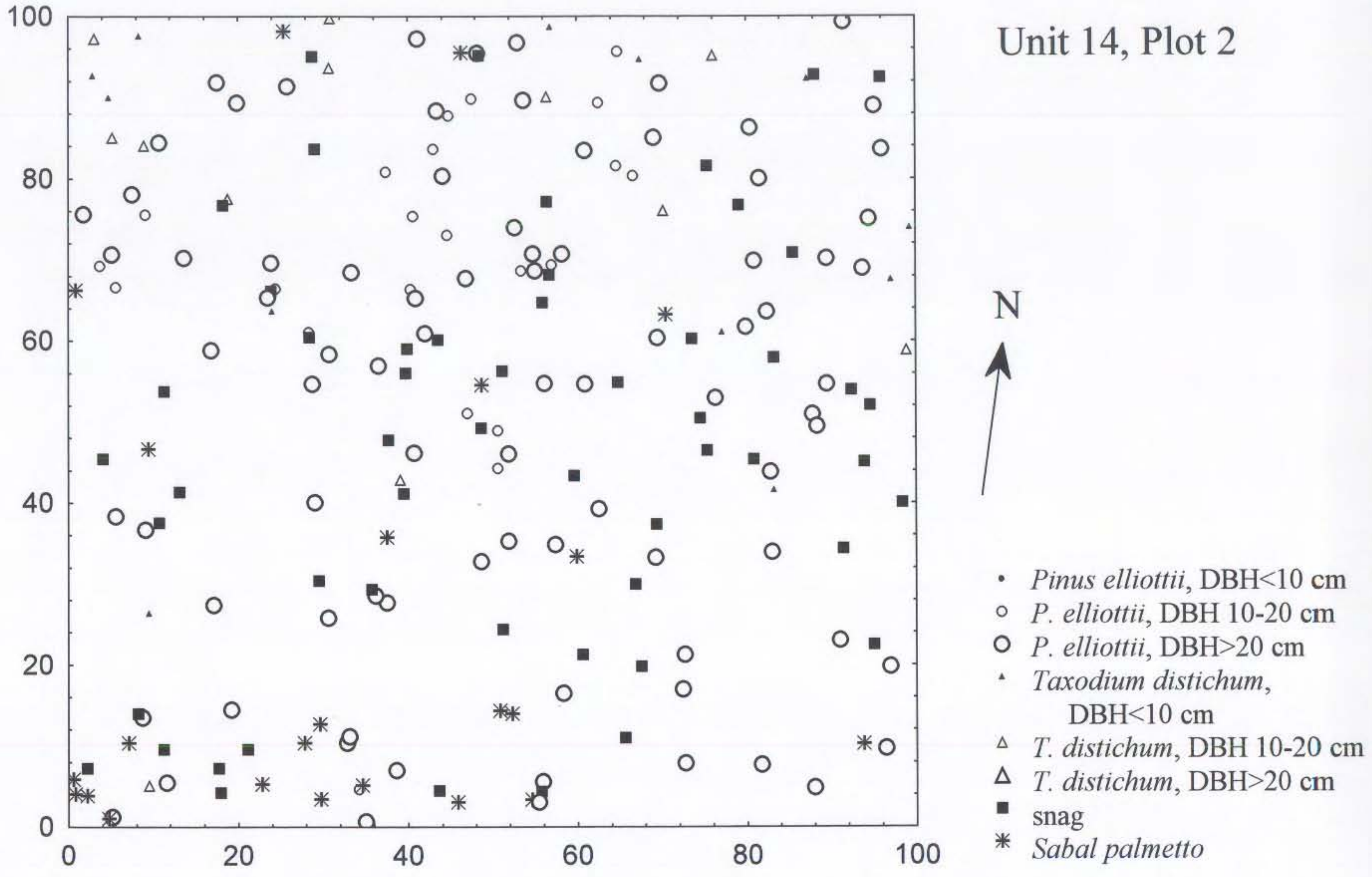
Unit 14, Plot 2
N
0
20
40
60
80
100
0
20
40
60
80
100
Pinus elliottii, DBH<10 cm
P. elliottii, DBH 10-20 cm
P. elliottii, DBH>20 cm
Taxodium distichum, DBH<10 cm
T. distichum, DBH 10-20 cm
T. distichum, DBH>20 cm
snag
Sabal palmetto

100

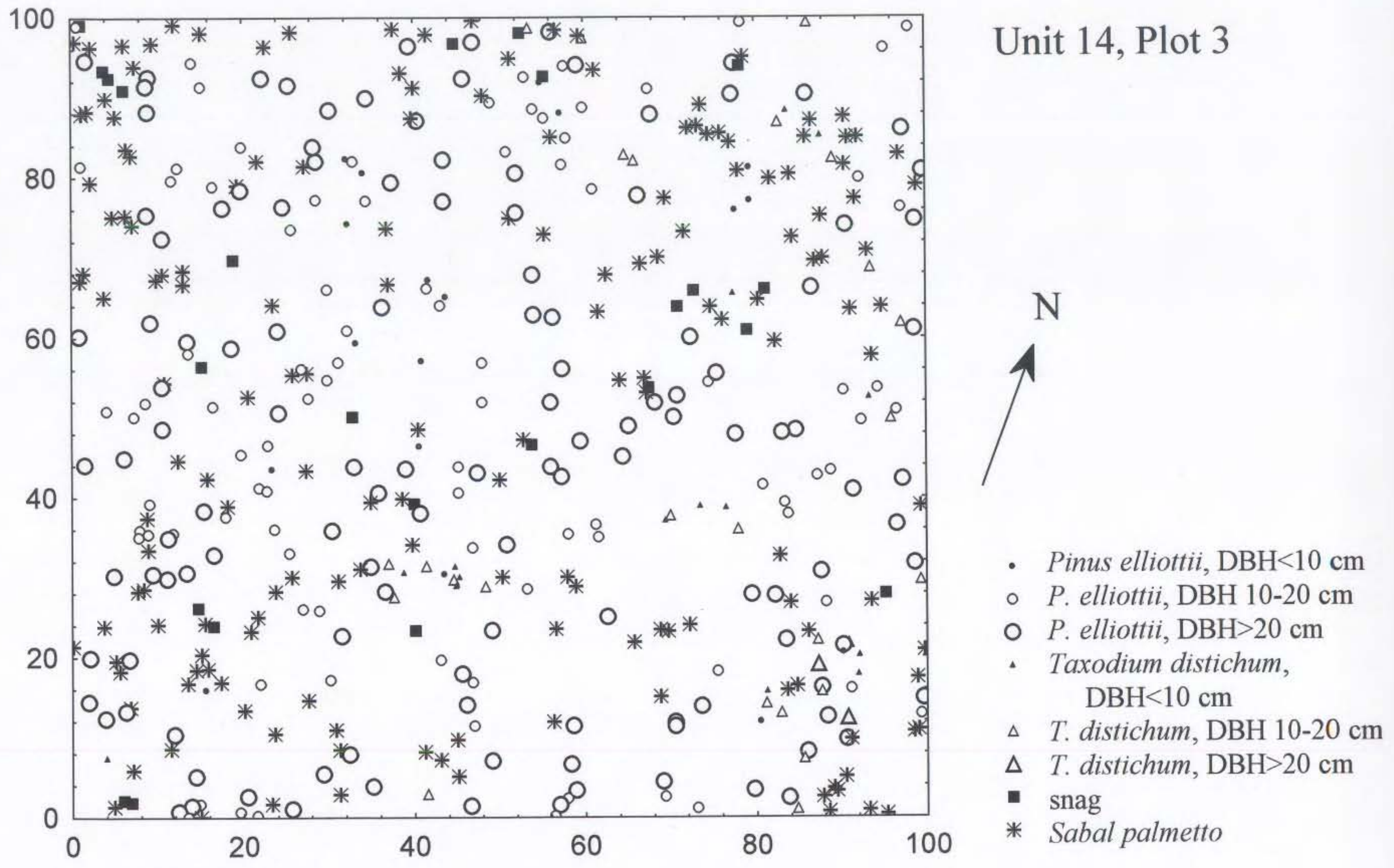
Unit 14, Plot 3
N
0
20
40
60
80
100
0
20
40
60
80
100
Pinus elliottii, DBH<10 cm
P. elliottii, DBH 10-20 cm
P. elliottii, DBH>20 cm
Taxodium distichum, DBH<10 cm
T. distichum, DBH 10-20 cm
T. distichum, DBH>20 cm
snag
Sabal palmetto

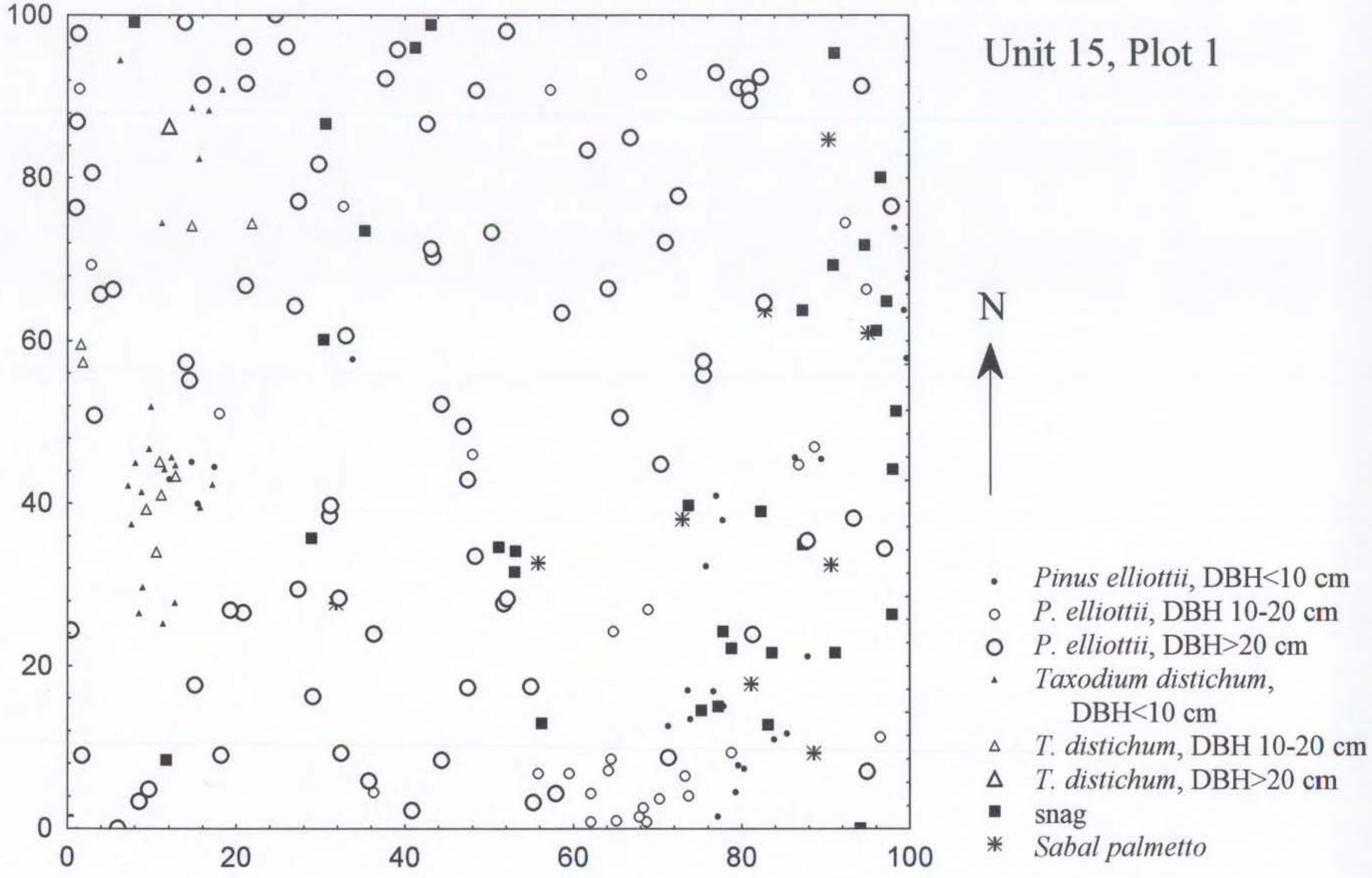
Unit 15, Plot 1
N
0
20
40
60
80
100
Pinus elliottii, DBH<10 cm
P. elliottii, DBH 10-20 cm
P. elliottii, DBH>20 cm
Taxodium distichum, DBH<10 cm
T. distichum, DBH 10-20 cm
T. distichum, DBH>20 cm
snag
Sabal palmetto

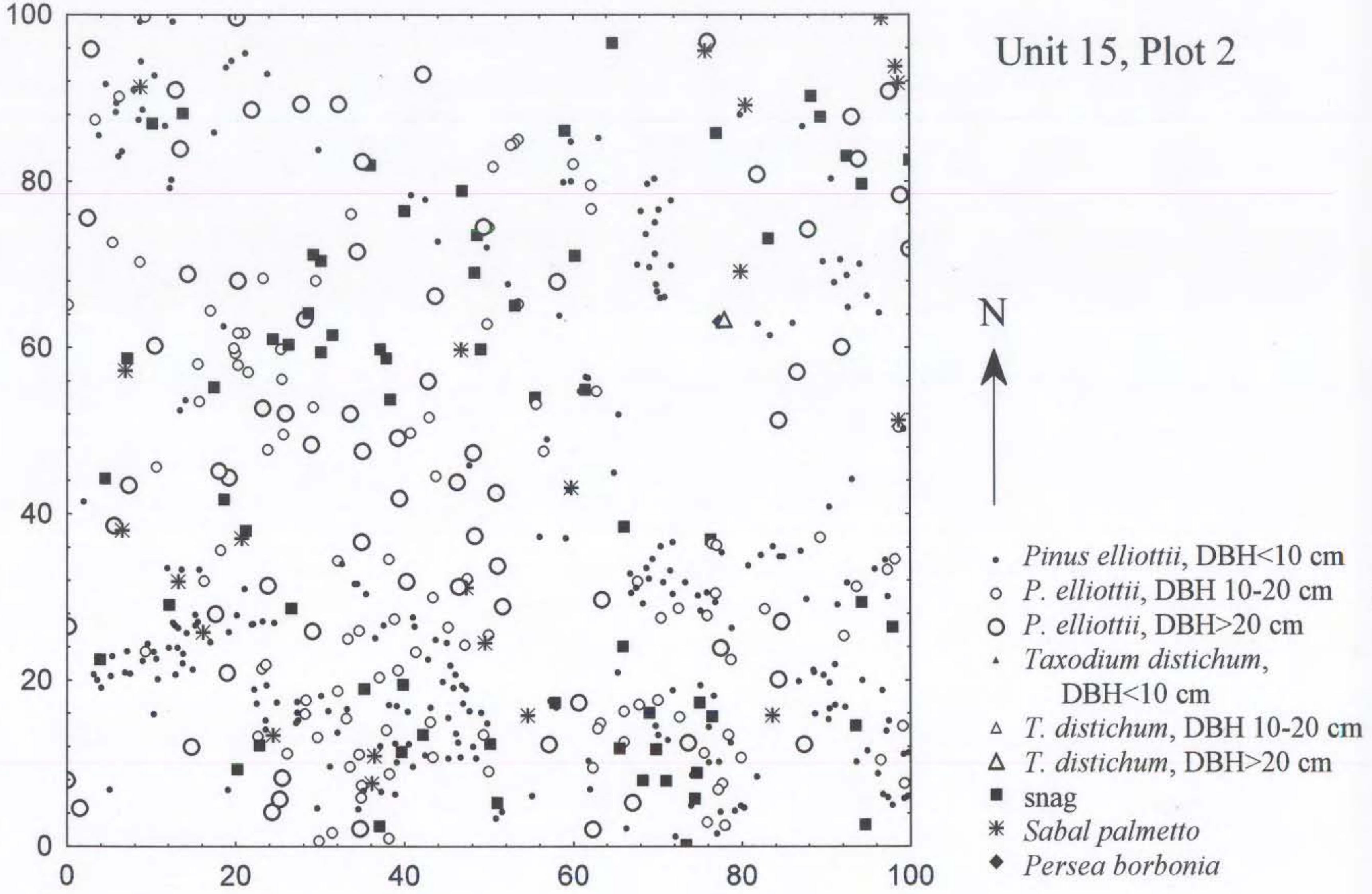
Unit 15, Plot 2
N
0
20
40
60
80
100
Pinus elliottii, DBH<10 cm
P. elliottii, DBH 10-20 cm
P. elliottii, DBH>20 cm
Taxodium distichum, DBH<10 cm
T. distichum, DBH 10-20 cm
T. distichum, DBH>20 cm
snag
Sabal palmetto
Persea borbonia

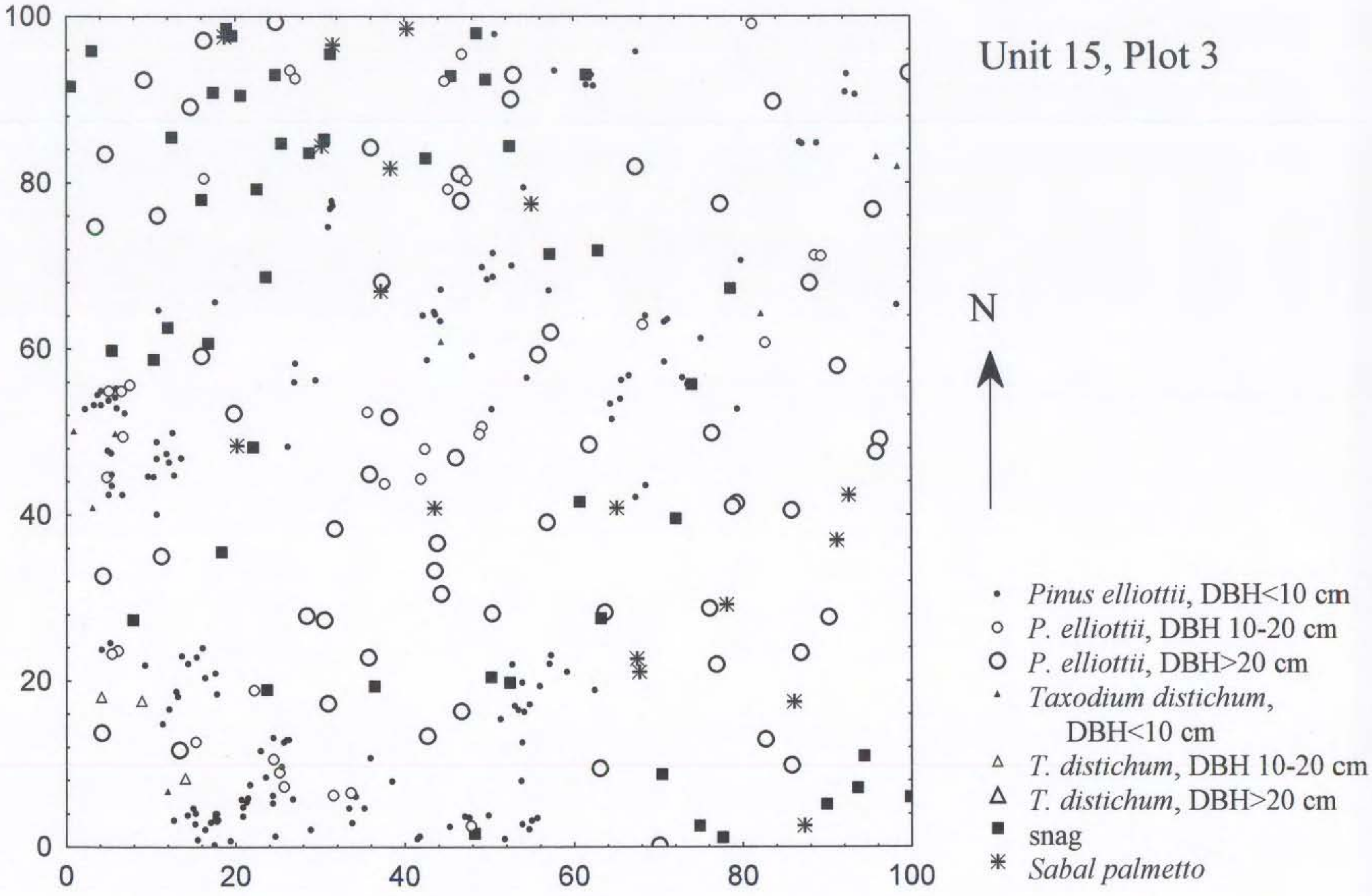

Unit 15, Plot 3
N
0
20
40
60
80
100
Pinus elliottii, DBH<10 cm
P. elliottii, DBH 10-20 cm
P. elliottii, DBH>20 cm
Taxodium distichum, DBH<10 cm
T. distichum, DBH 10-20 cm
T. distichum, DBH>20 cm
snag
Sabal palmetto

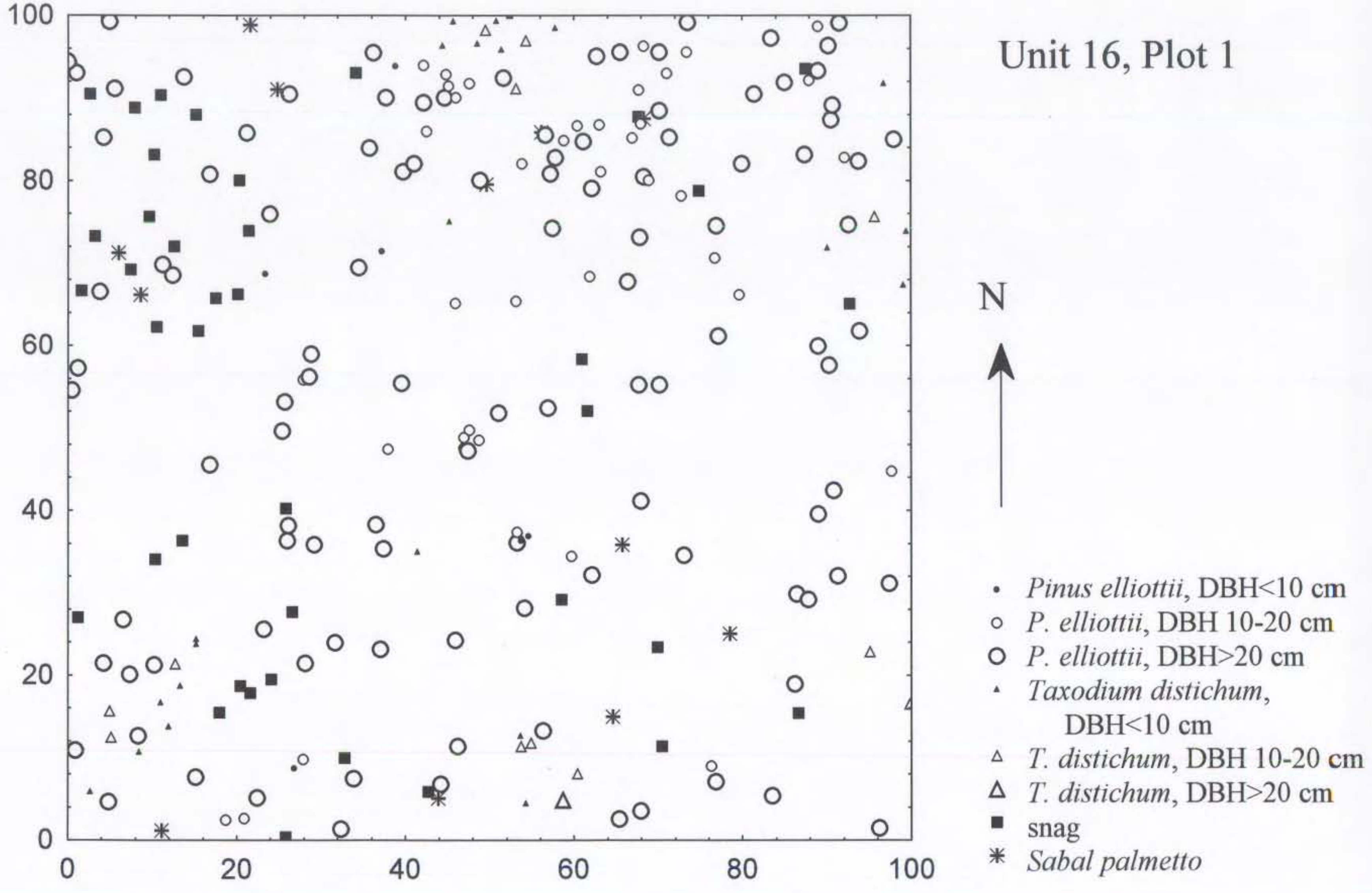

Unit 16, Plot 1
N
• Pinus elliottii, DBH<10 cm
○ P. elliottii, DBH 10-20 cm
O P. elliottii, DBH>20 cm
▲ Taxodium distichum, DBH<10 cm
△ T. distichum, DBH 10-20 cm
△ T. distichum, DBH>20 cm
■ snag
✳ Sabal palmetto

Unit 16, Plot 2

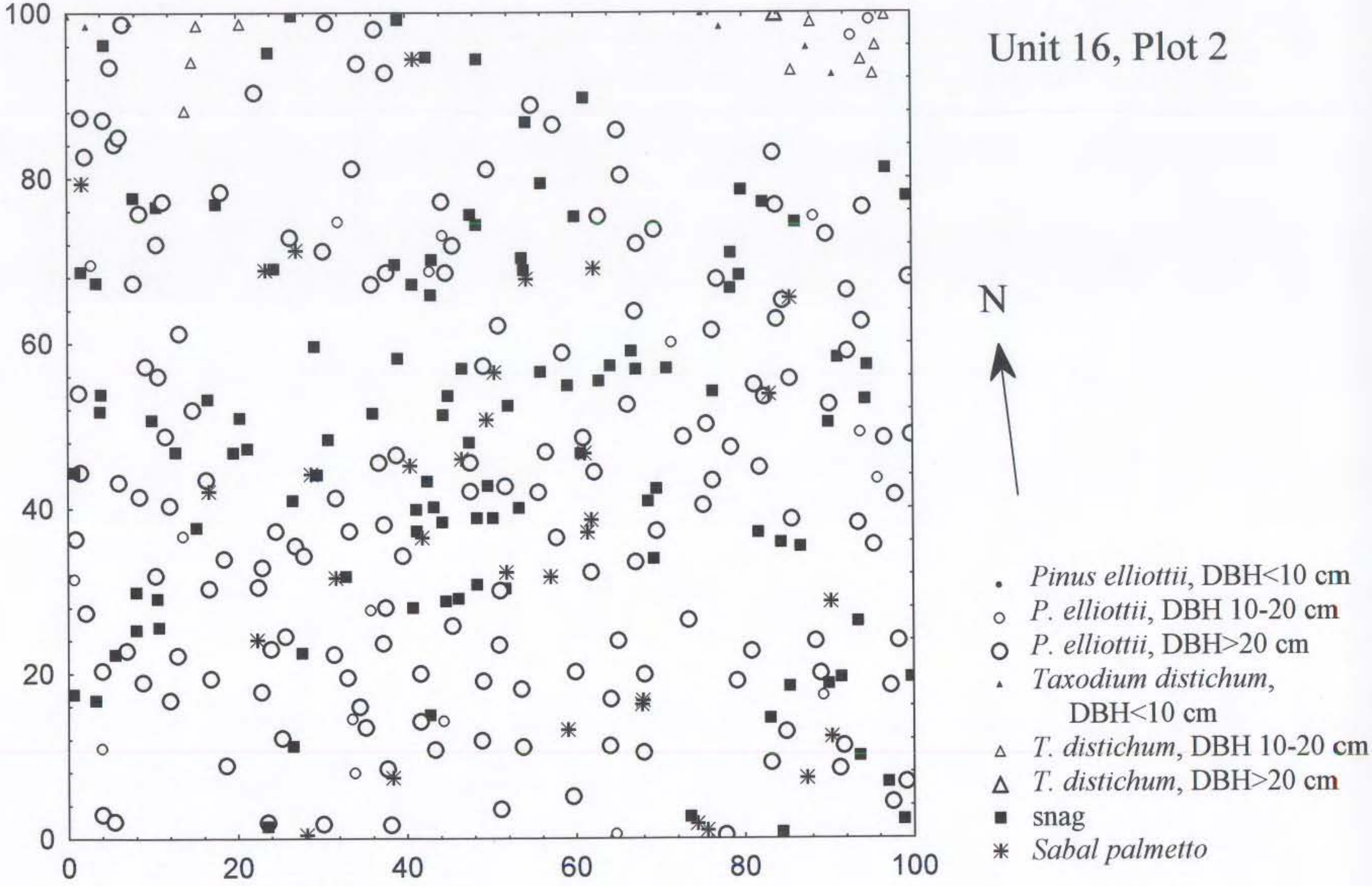

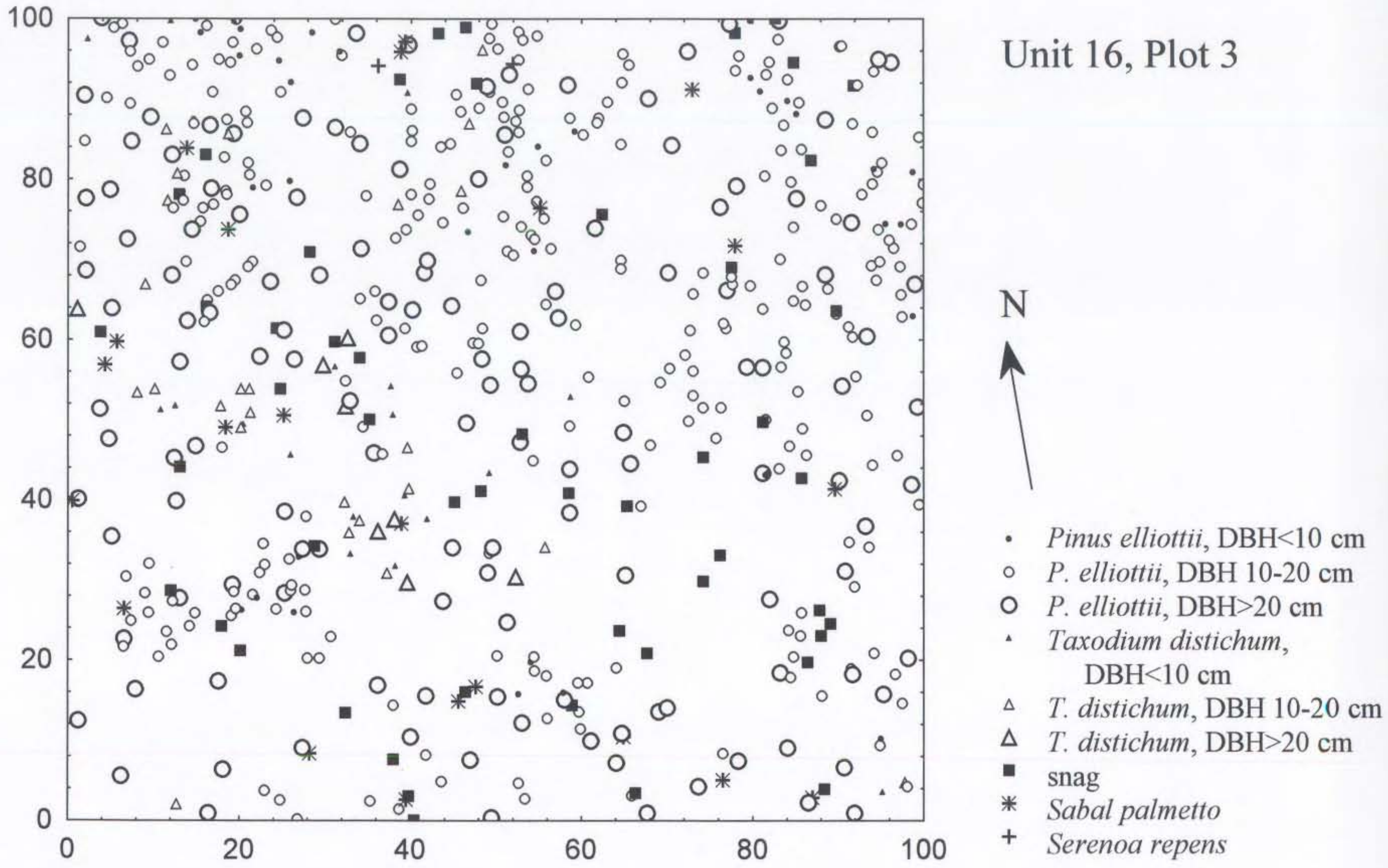
Unit 16, Plot 3
N
100
80
60
40
20
0
0
20
40
60
80
100
Pinus elliottii, DBH<10 cm
P. elliottii, DBH 10-20 cm
P. elliottii, DBH>20 cm
Taxodium distichum, DBH<10 cm
T. distichum, DBH 10-20 cm
T. distichum, DBH>20 cm
snag
Sabal palmetto
Serenoa repens

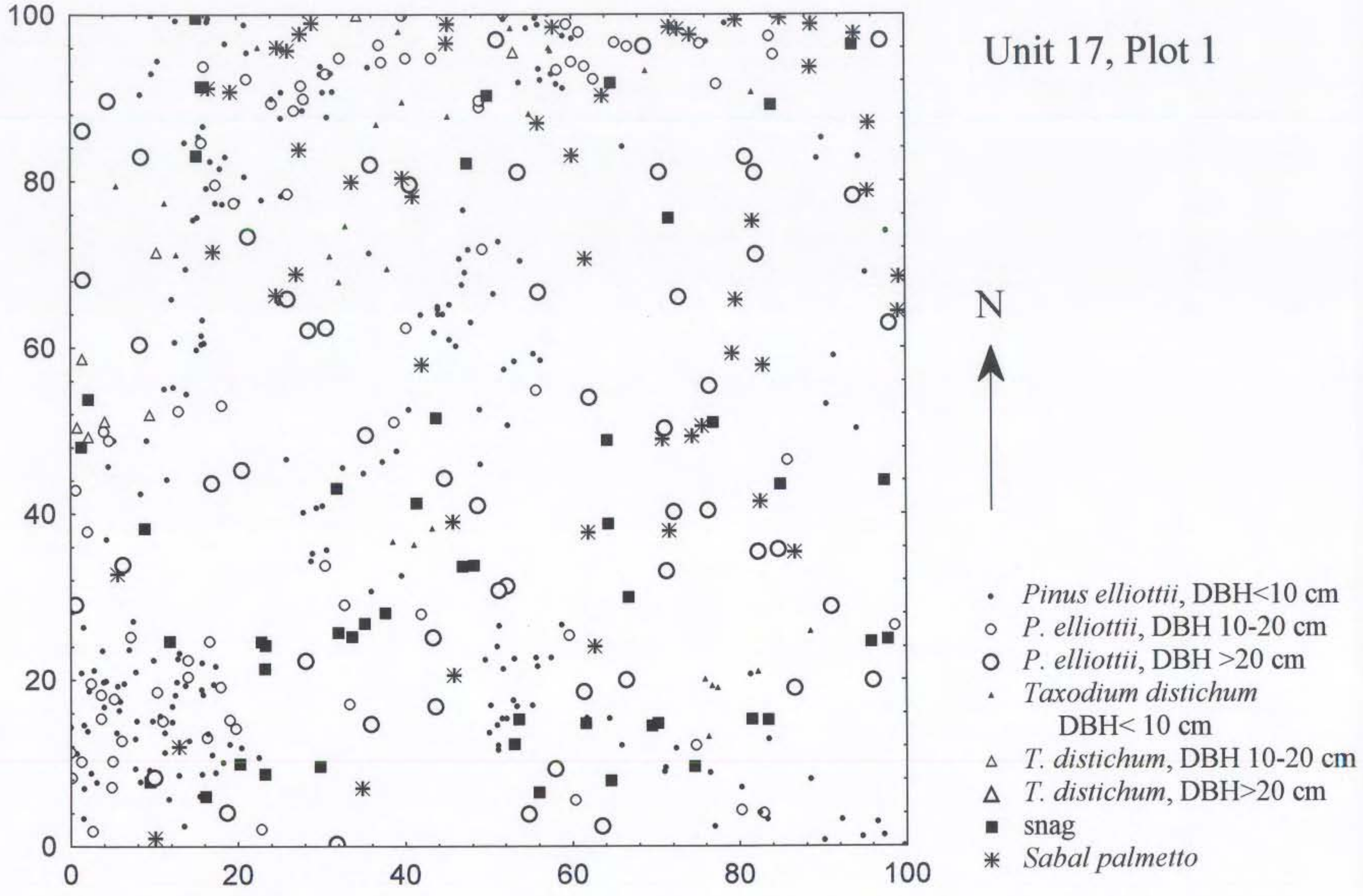
Unit 17, Plot 1
N
0
20
40
60
80
100
0
20
40
60
80
100
Pinus elliottii, DBH<10 cm
P. elliottii, DBH 10-20 cm
P. elliottii, DBH >20 cm
Taxodium distichum DBH< 10 cm
T. distichum, DBH 10-20 cm
T. distichum, DBH>20 cm
snag
Sabal palmetto

Unit 17, Plot 2

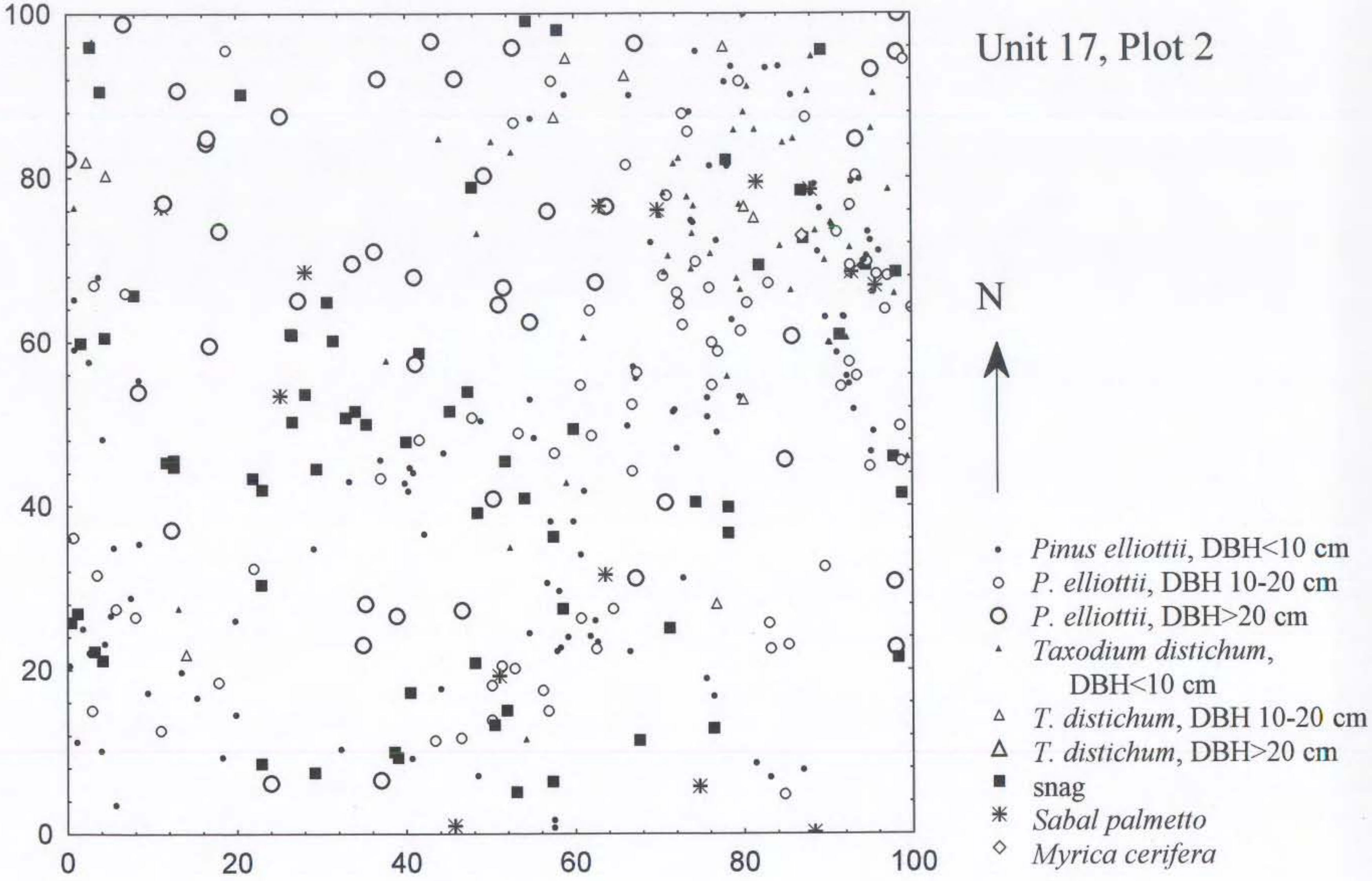

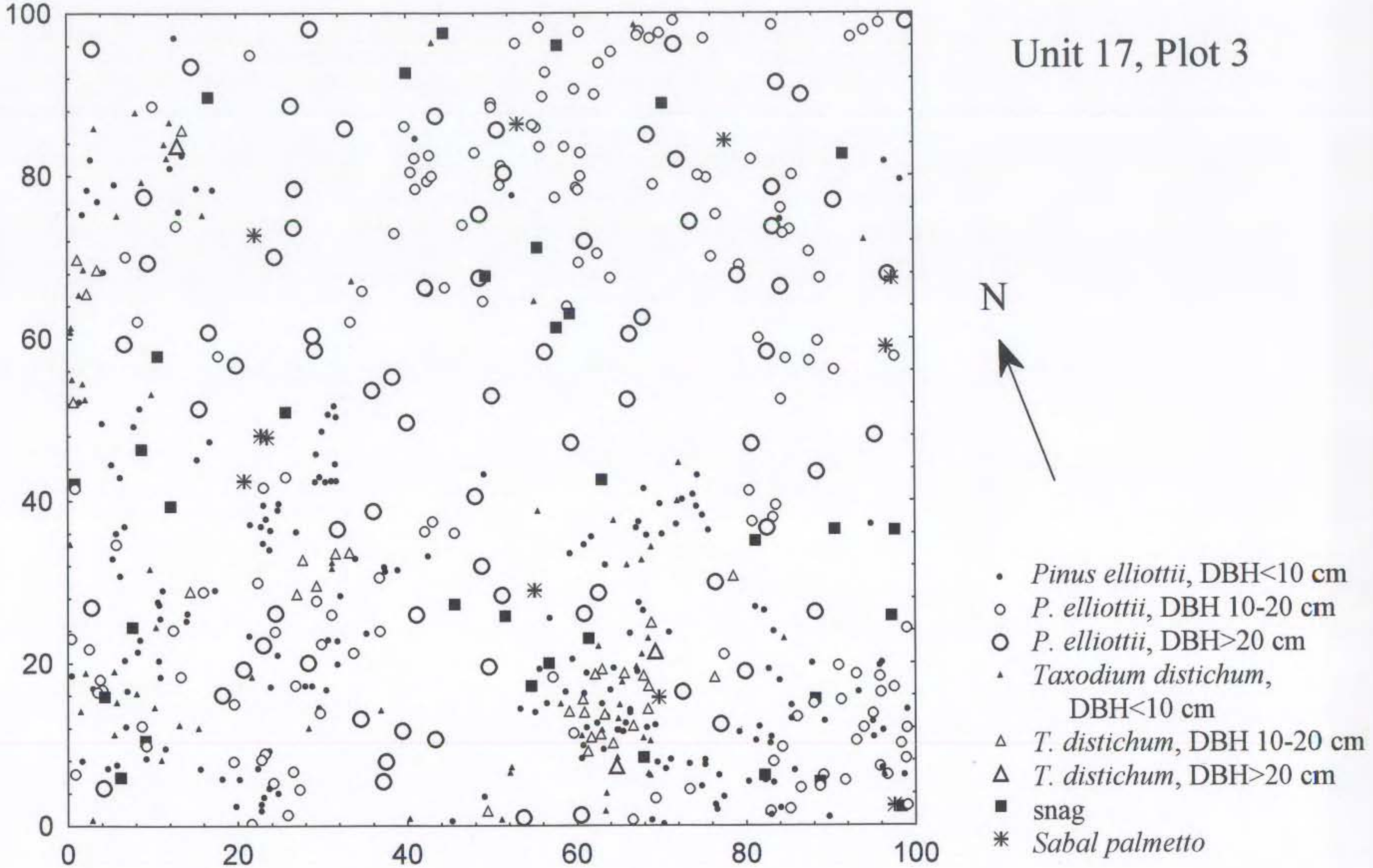
Unit 17, Plot 3
N
0
20
40
60
80
100
Pinus elliottii, DBH<10 cm
P. elliottii, DBH 10-20 cm
P. elliottii, DBH>20 cm
Taxodium distichum, DBH<10 cm
T. distichum, DBH 10-20 cm
T. distichum, DBH>20 cm
snag
Sabal palmetto

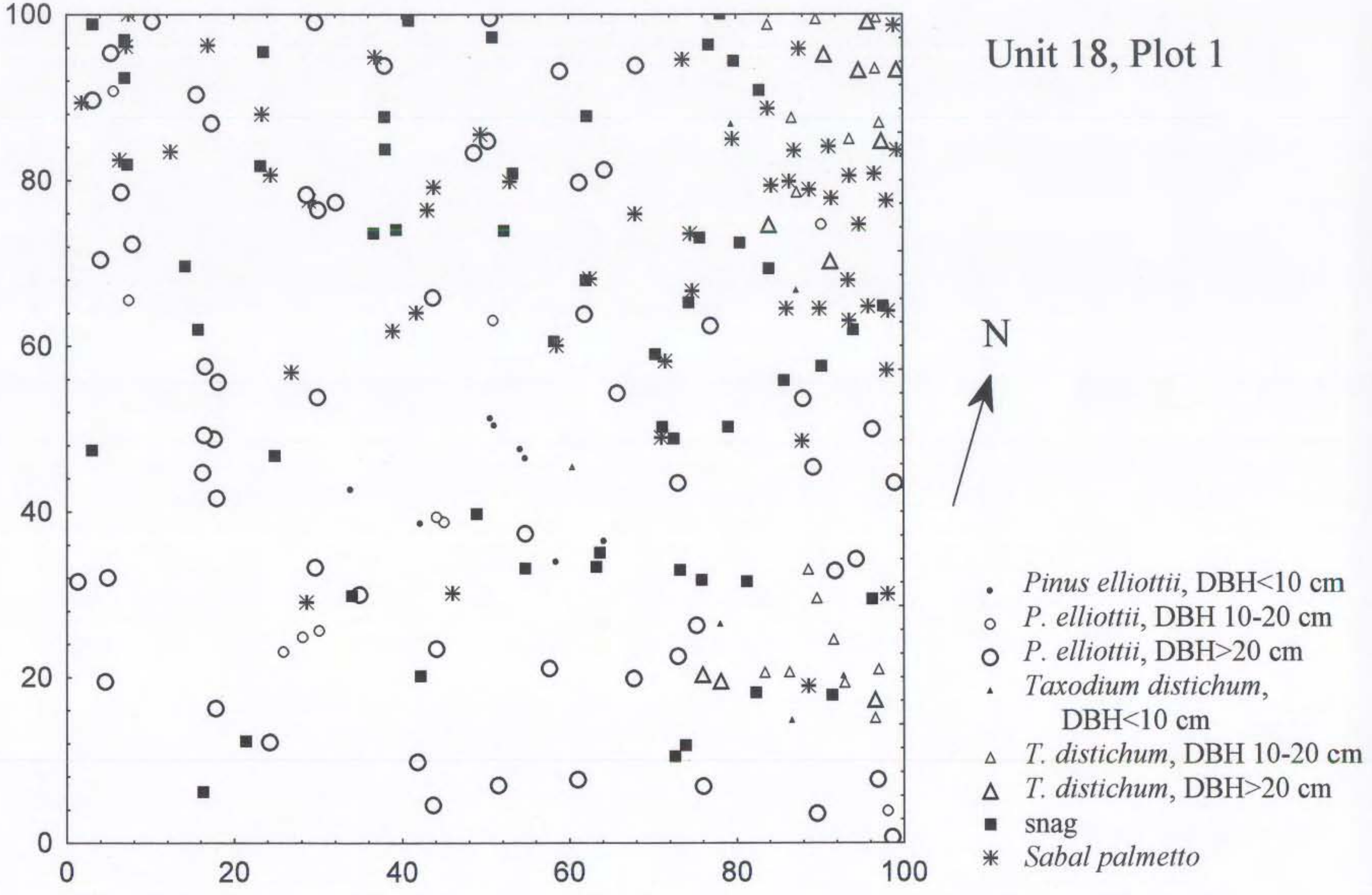
Unit 18, Plot 1
N
Pinus elliottii, DBH<10 cm
P. elliottii, DBH 10-20 cm
P. elliottii, DBH>20 cm
Taxodium distichum, DBH<10 cm
T. distichum, DBH 10-20 cm
T. distichum, DBH>20 cm
snag
Sabal palmetto
0
20
40
60
80
100

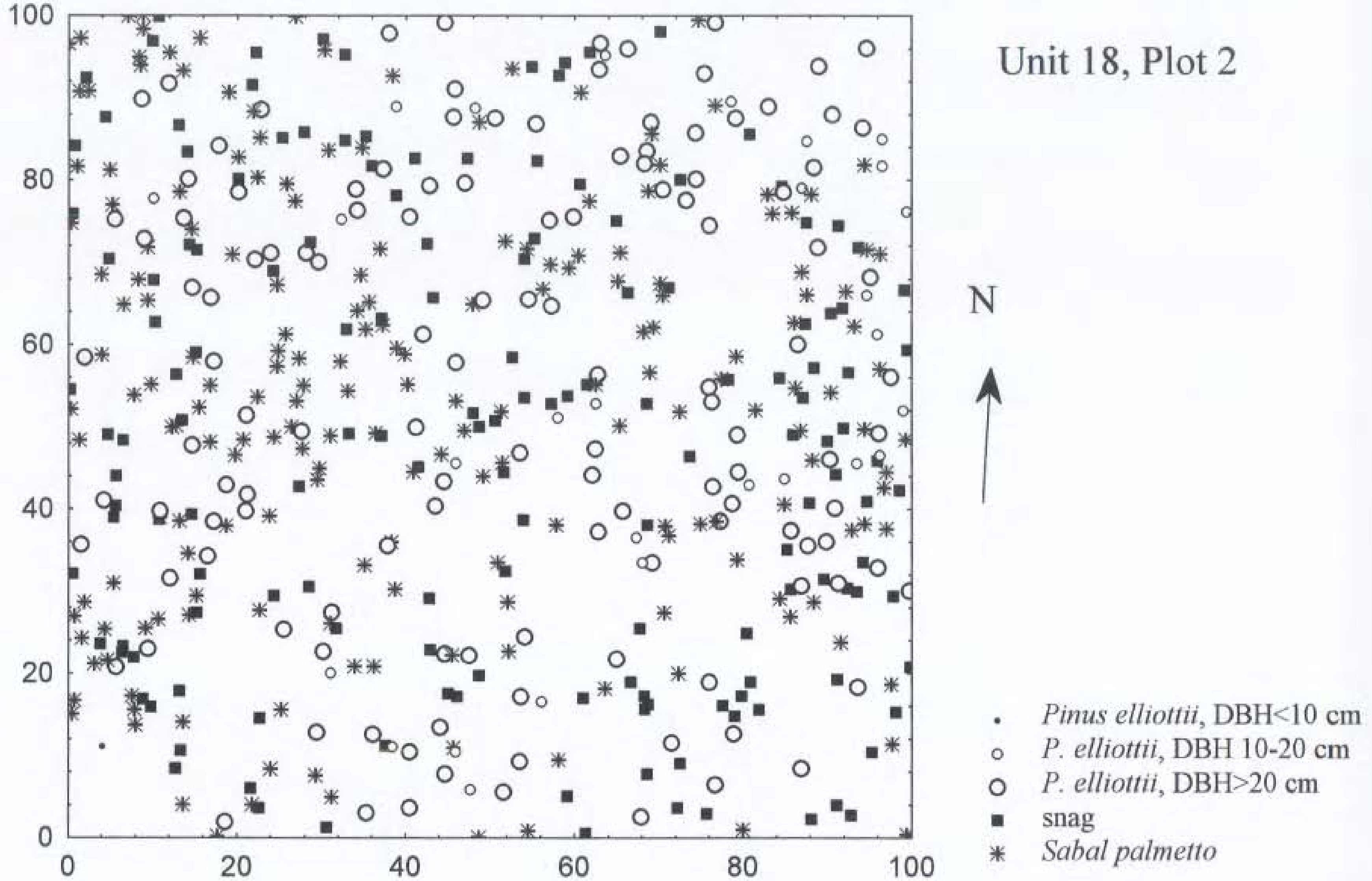
Unit 18, Plot 2
N
0
20
40
60
80
100
Pinus elliottii, DBH<10 cm
P. elliottii, DBH 10-20 cm
P. elliottii, DBH>20 cm
snag
Sabal palmetto

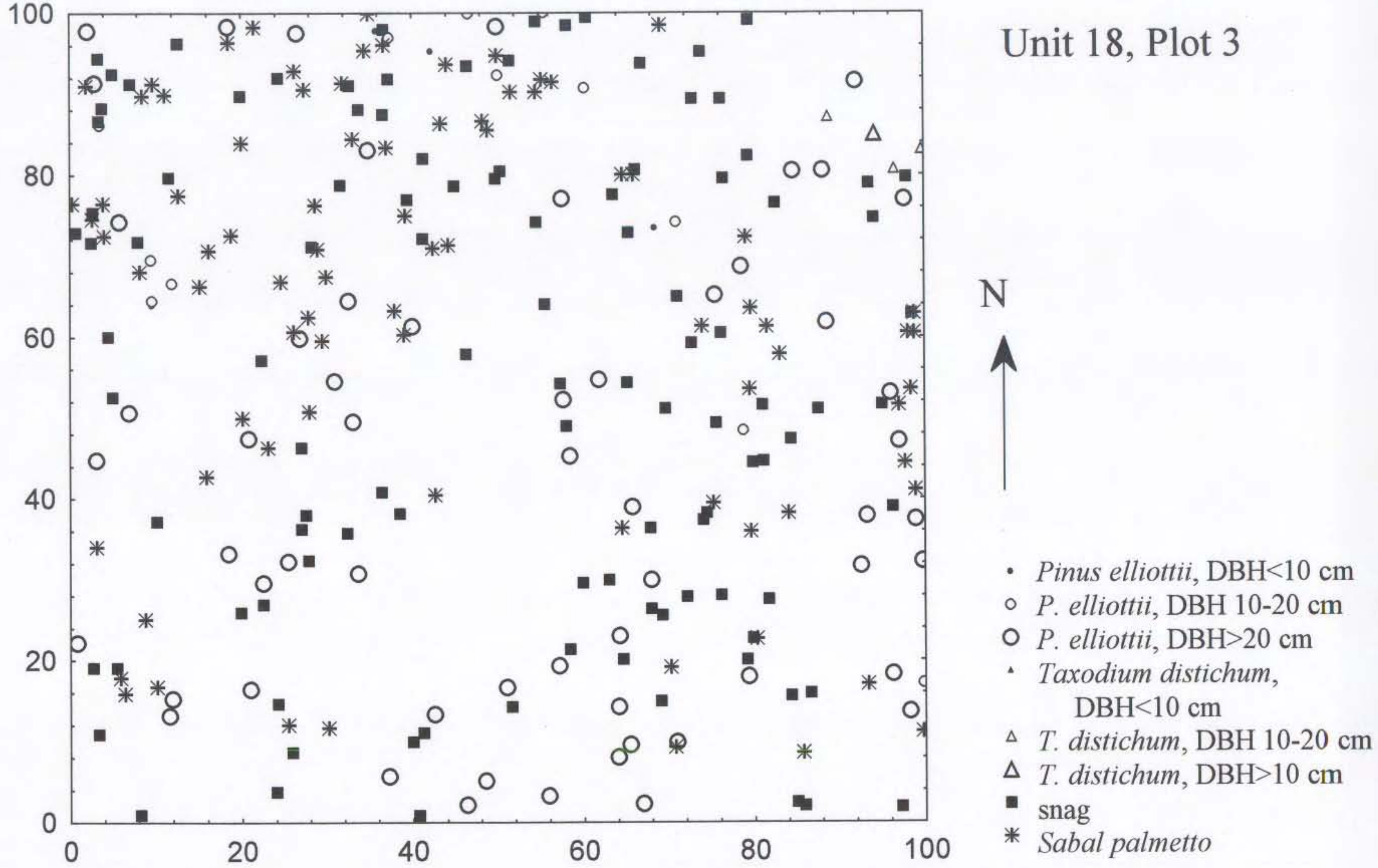
Unit 18, Plot 3
N
0
20
40
60
80
100
0
20
40
60
80
100
Pinus elliottii, DBH<10 cm
P. elliottii, DBH 10-20 cm
P. elliottii, DBH>20 cm
Taxodium distichum, DBH<10 cm
T. distichum, DBH 10-20 cm
T. distichum, DBH>10 cm
snag
Sabal palmetto

9.3 Size-class Distribution of Pines in Tree Plots

UNIT 1 PLOT 3

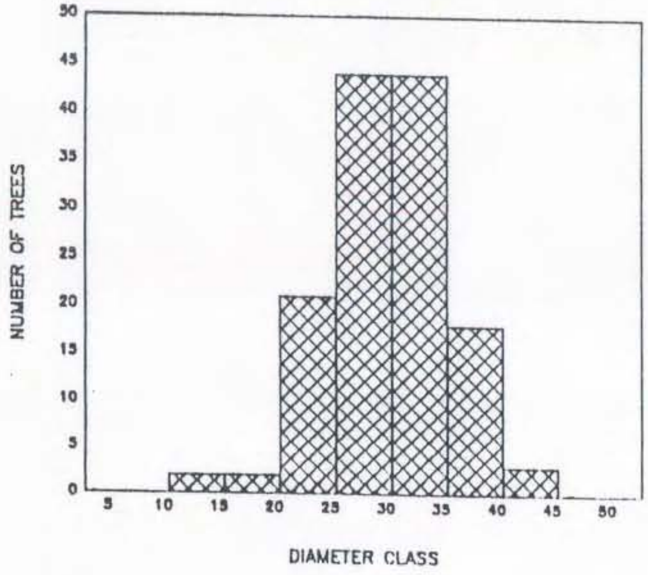

UNIT 1 PLOT 2

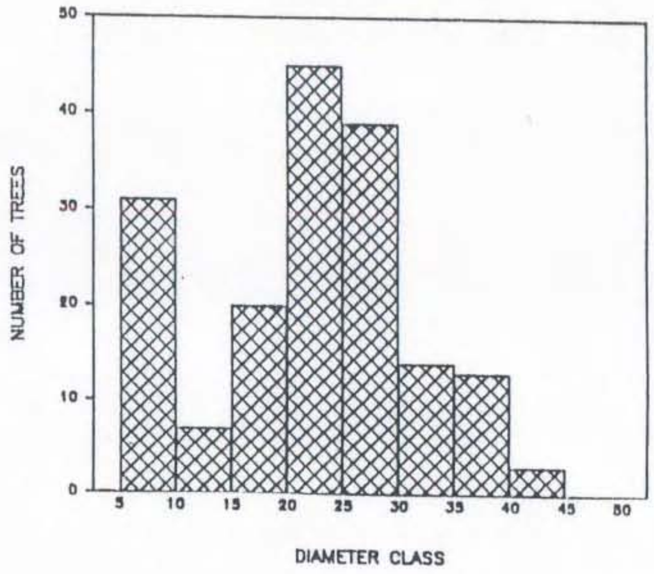

UNIT 1 PLOT 1

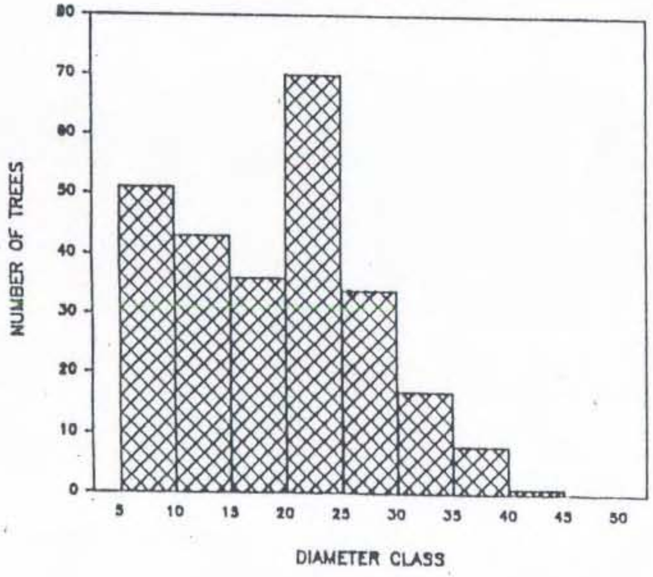

UNIT 2 PLOT 1

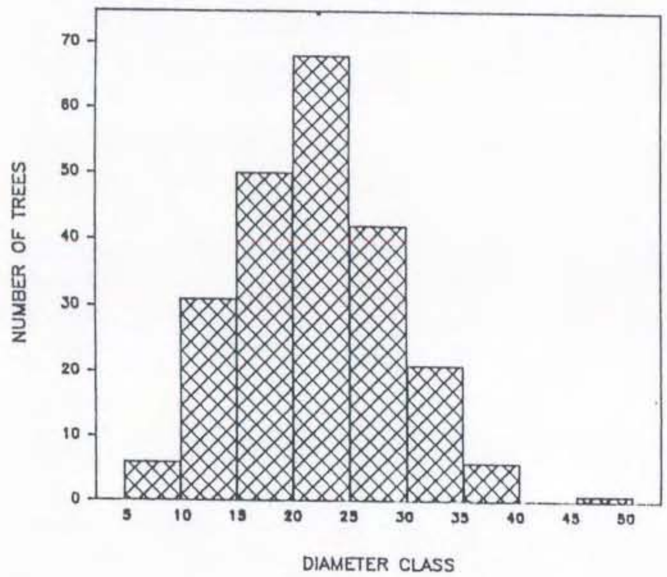

UNIT 2 PLOT 2

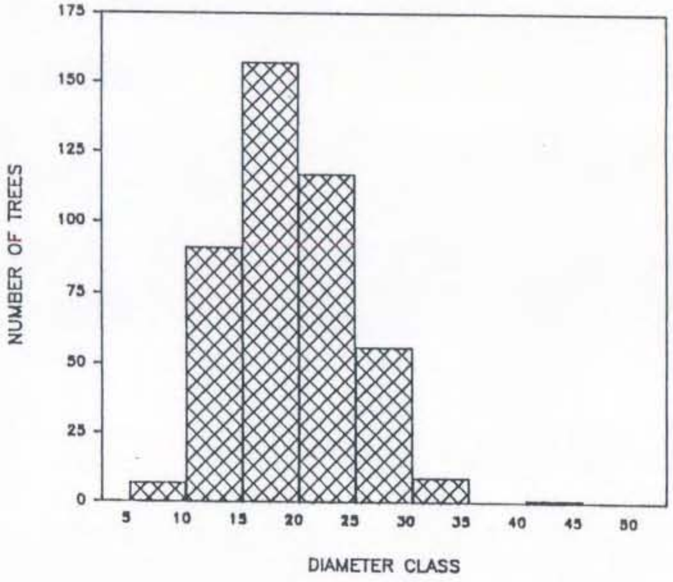

UNIT 2 PLOT 3

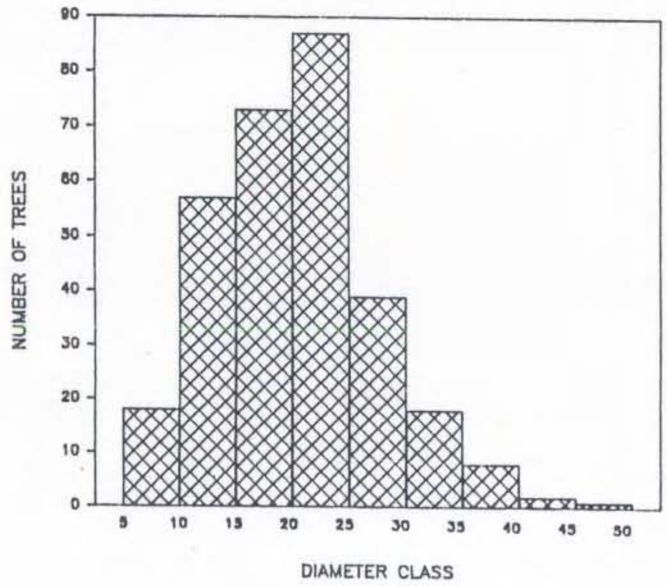

UNIT 3 PLOT 2

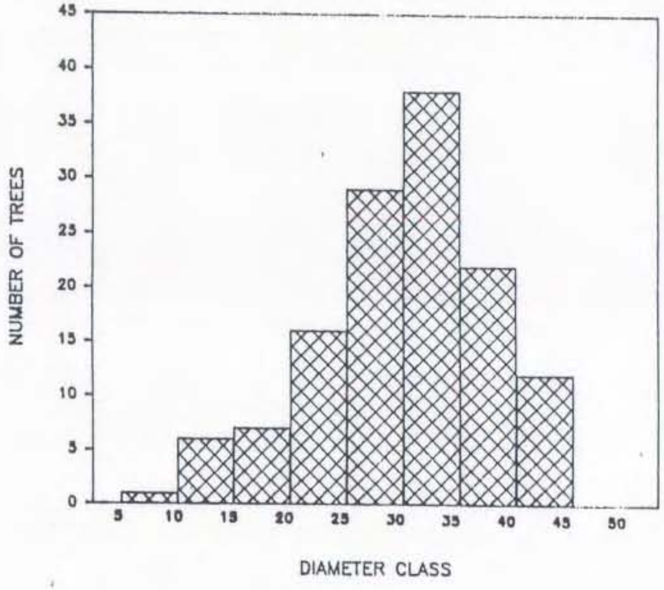

UNIT 3 PLOT 1

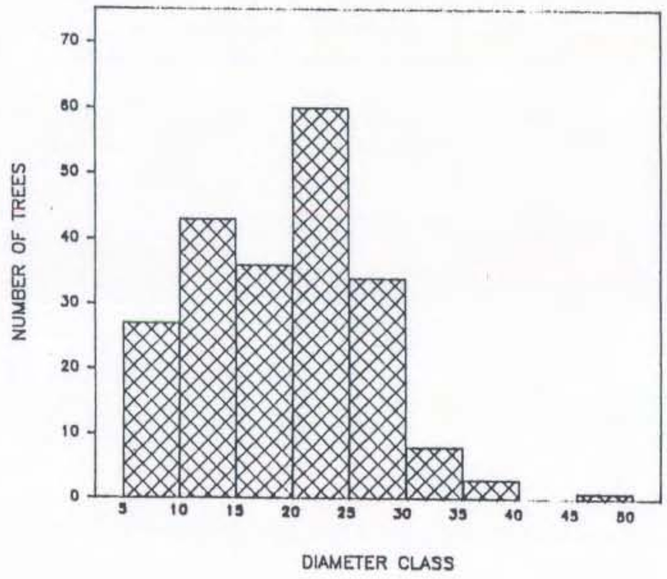

UNIT 3 PLOT 3

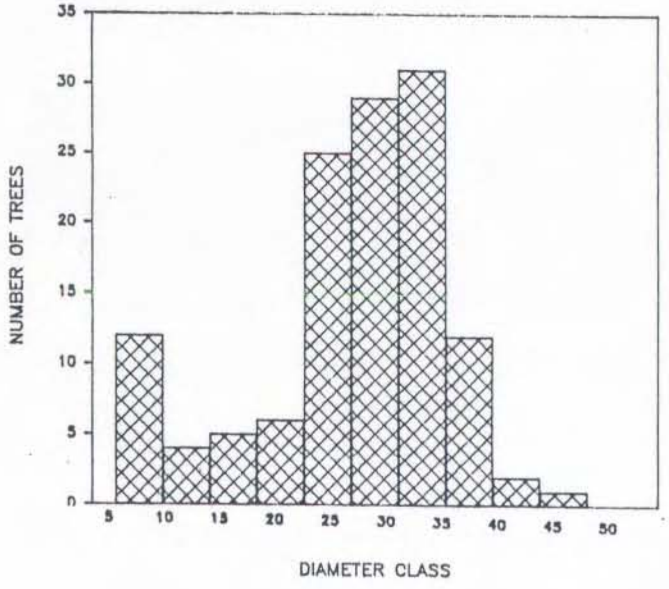

UNIT 4 PLOT 3

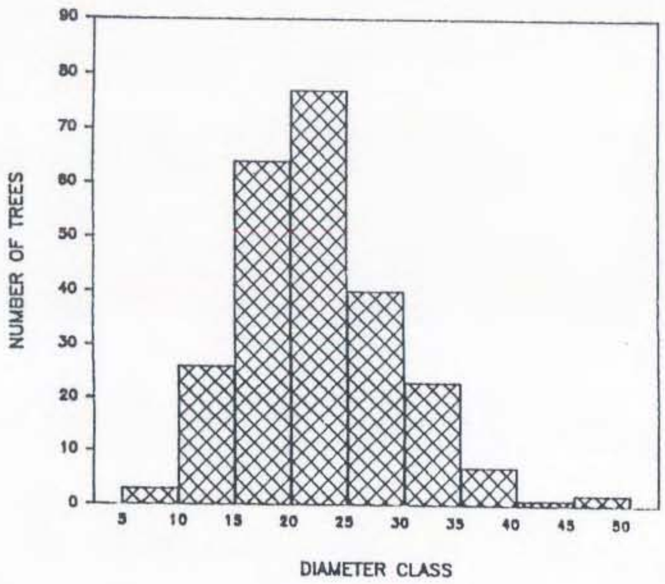

UNIT 4 PLOT 1

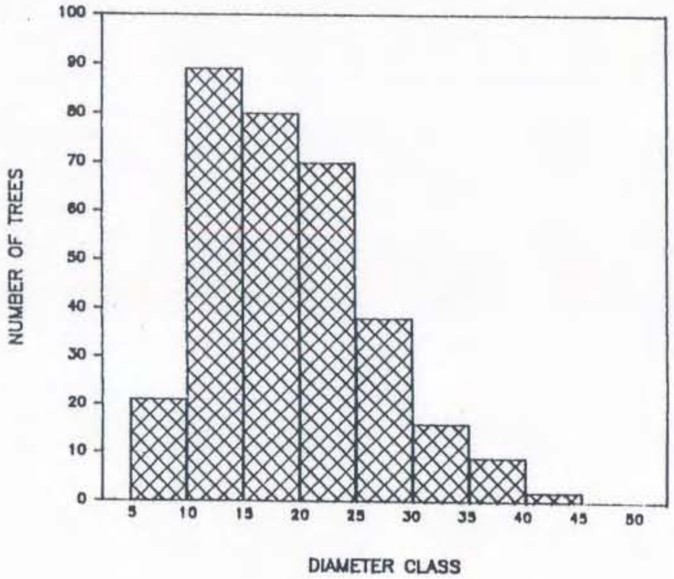

UNIT 4 PLOT 2

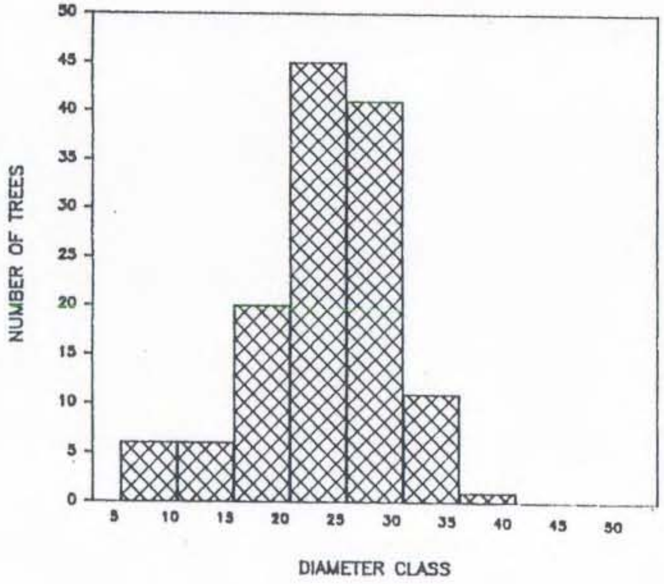

UNIT 5 PLOT 2

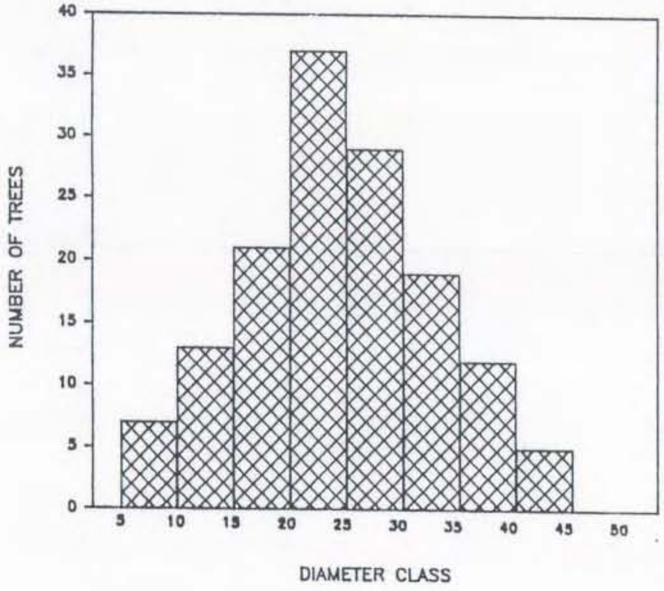

UNIT 5 PLOT 1

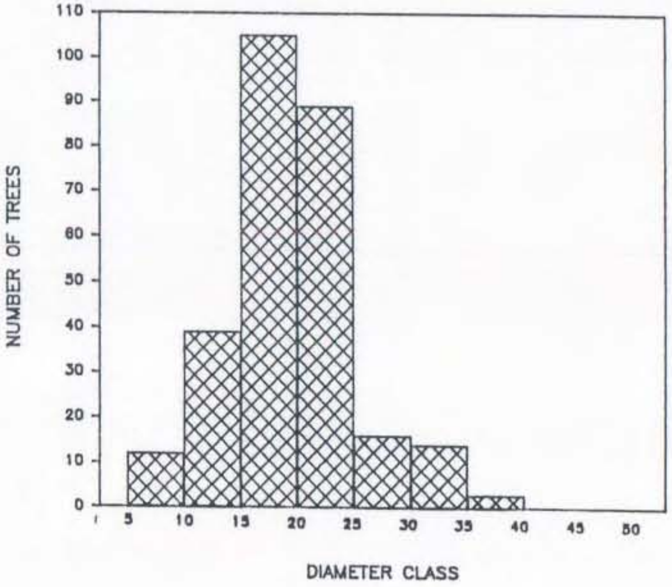

UNIT 5 PLOT 3

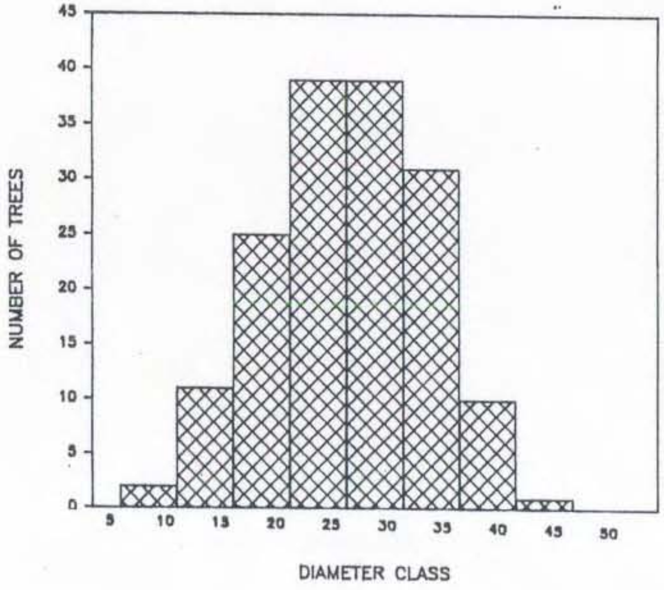

UNIT 6 PLOT 2

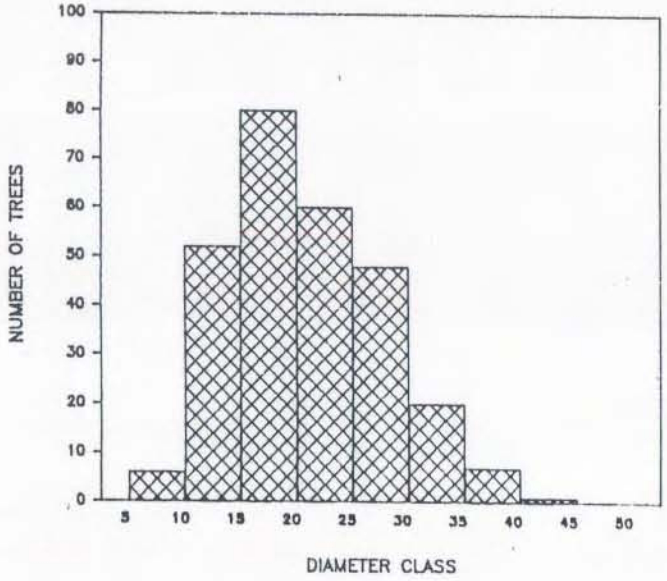

UNIT 6 PLOT 3

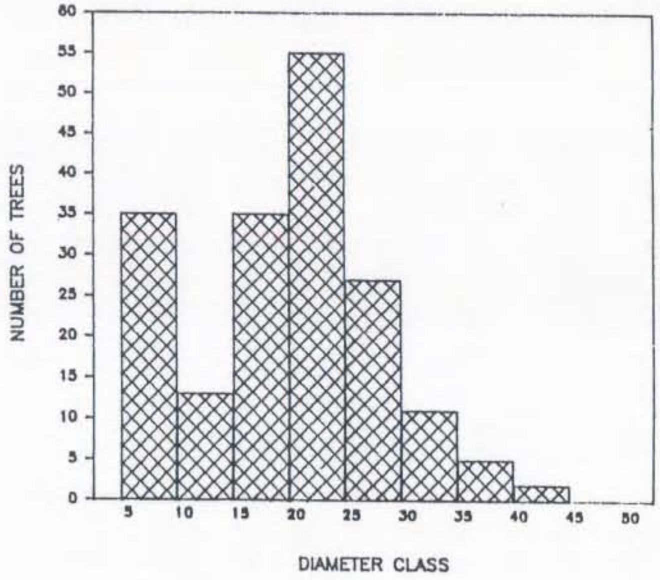

UNIT 6 PLOT 1

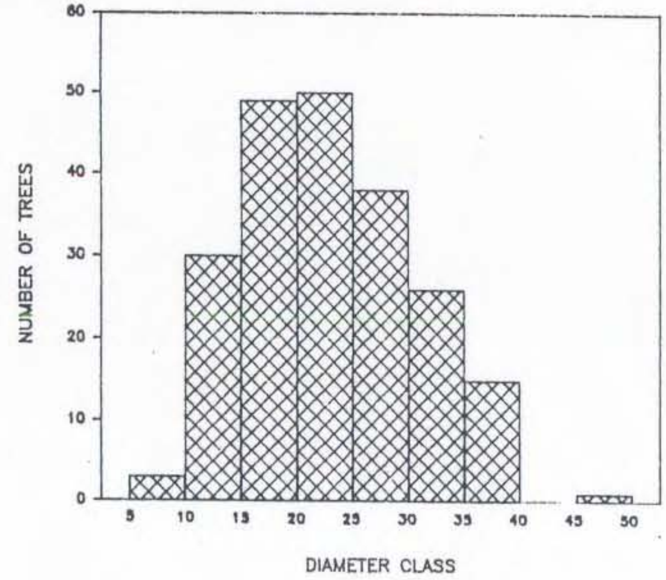

UNIT 7 PLOT 2

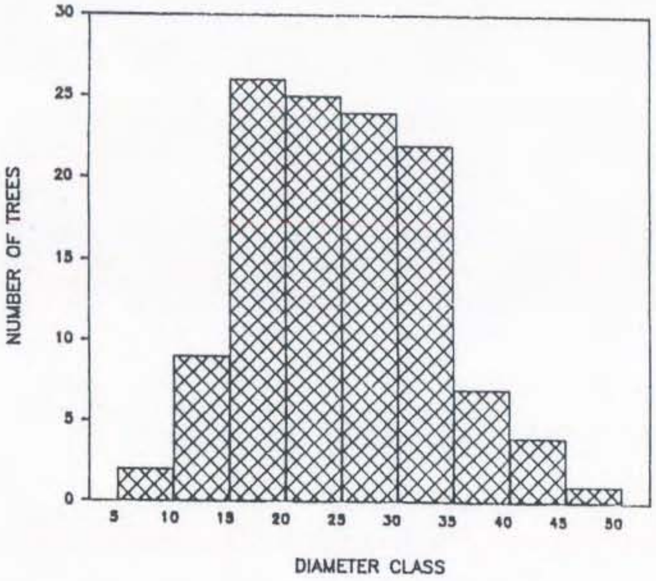

UNIT 7 PLOT 3

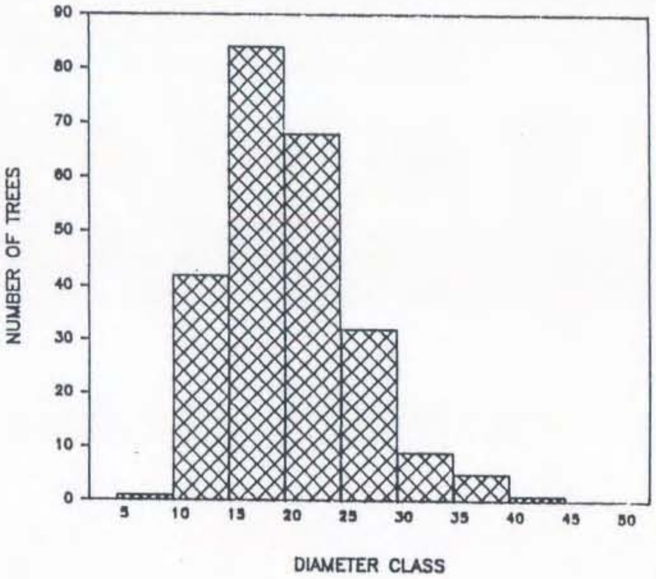

UNIT 7 PLOT 1

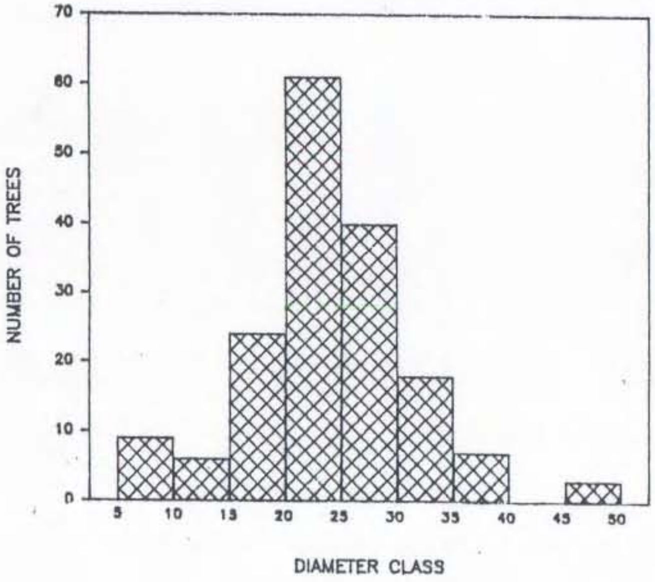

UNIT 8 PLOT 2

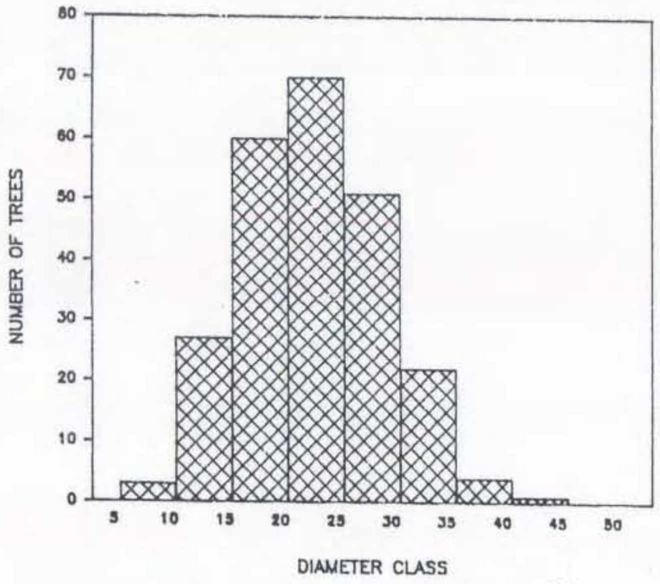

UNIT 8 PLOT 3

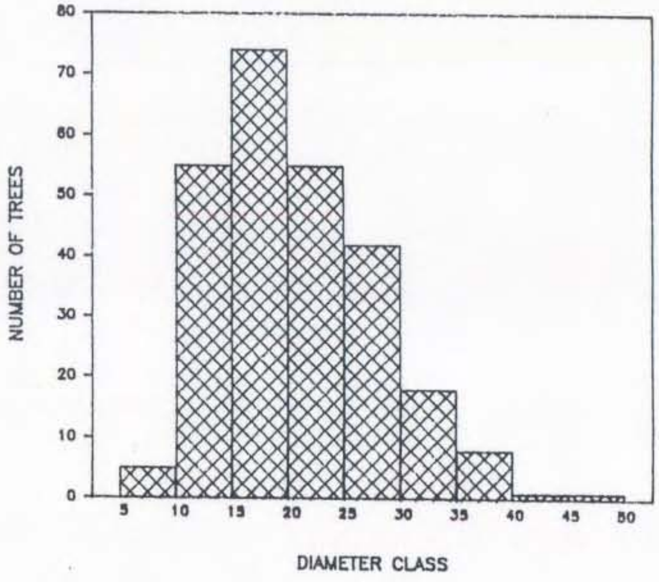

UNIT 8 PLOT 1

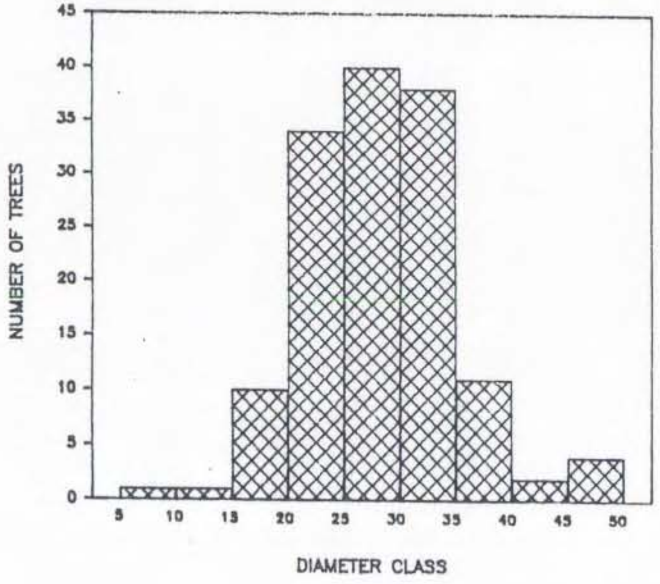

UNIT 9 PLOT 2

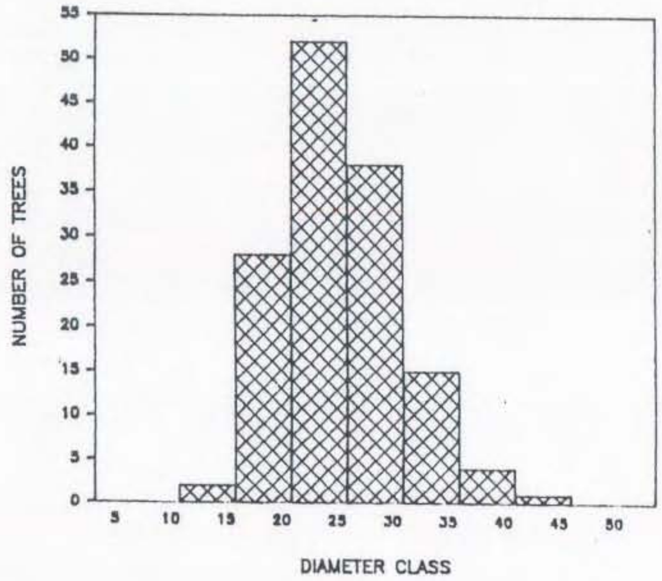

UNIT 9 PLOT 3

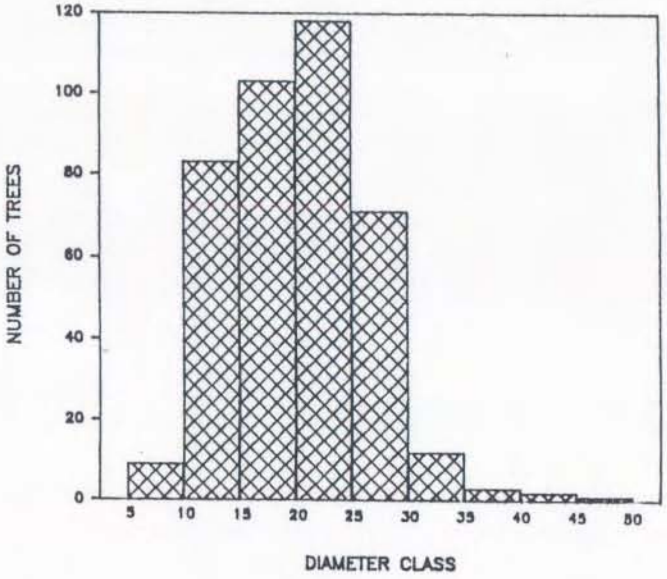

UNIT 9 PLOT 1

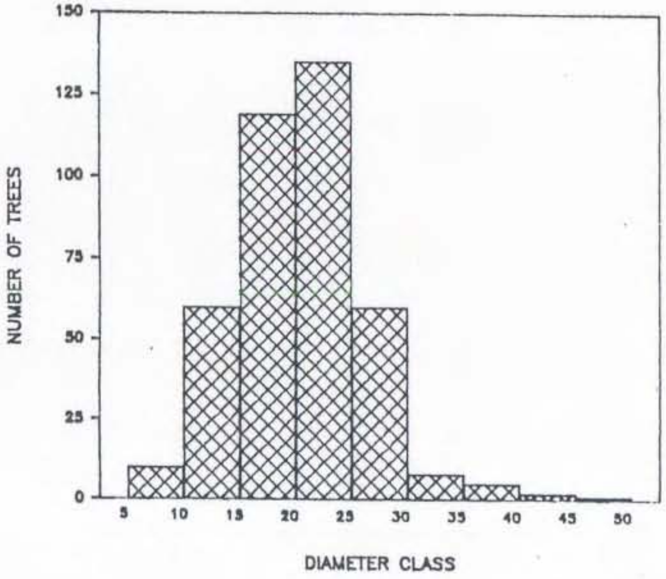

UNIT 10 PLOT 1

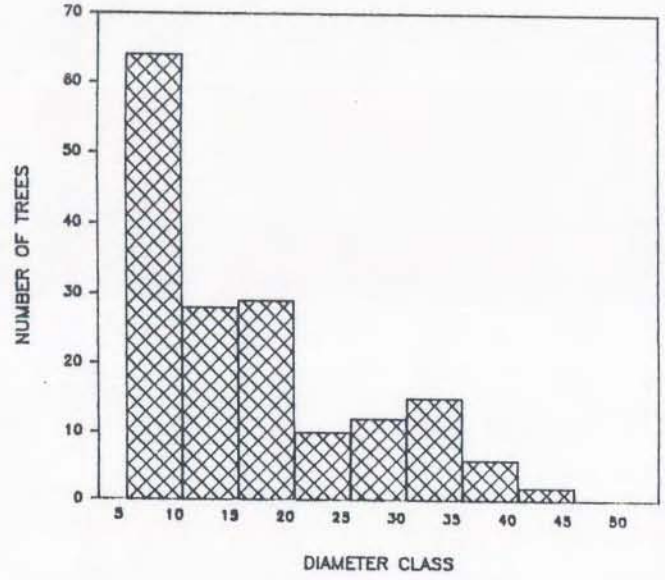

UNIT 10 PLOT 3

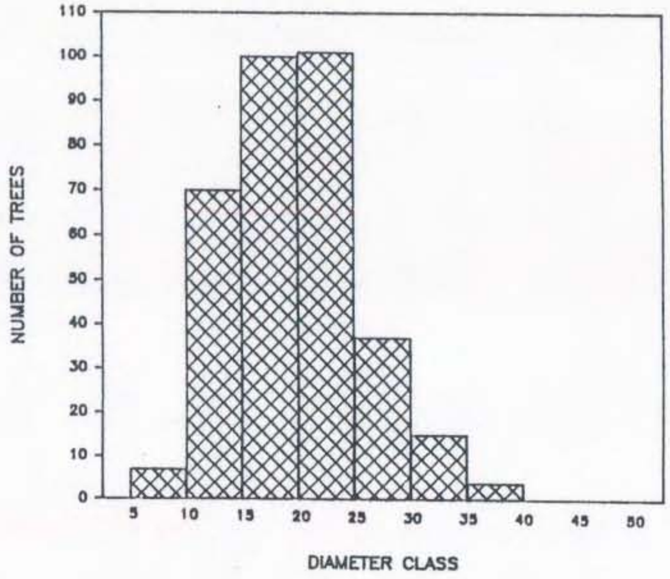

UNIT 10 PLOT 2

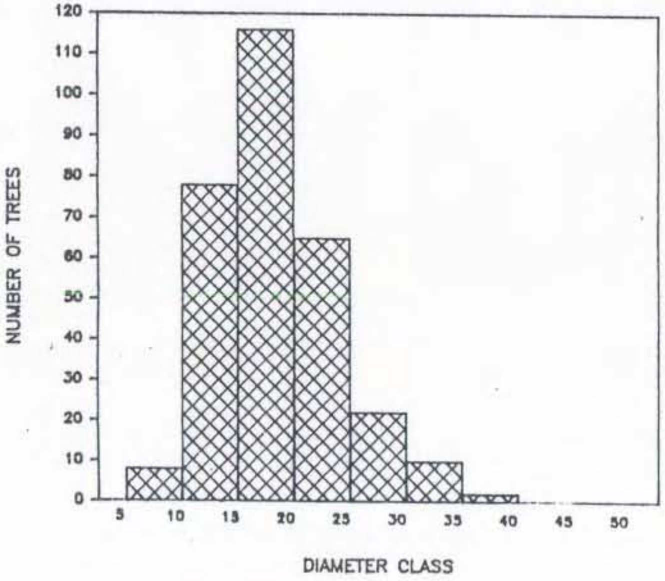

UNIT 11 PLOT 1

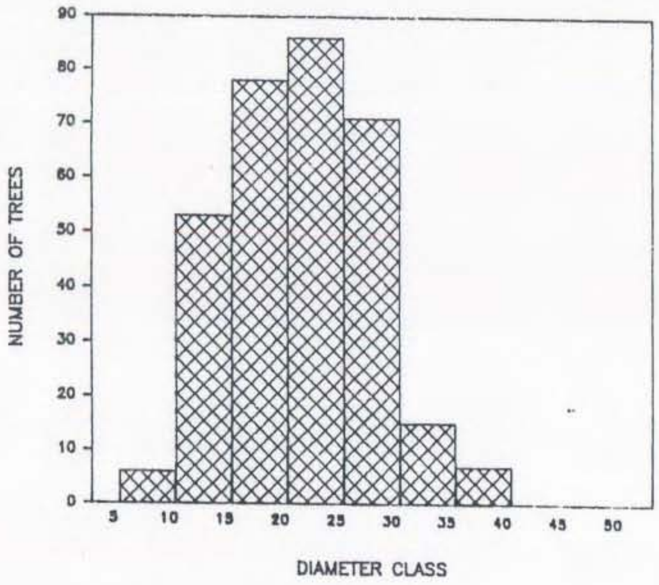

UNIT 11 PLOT 3

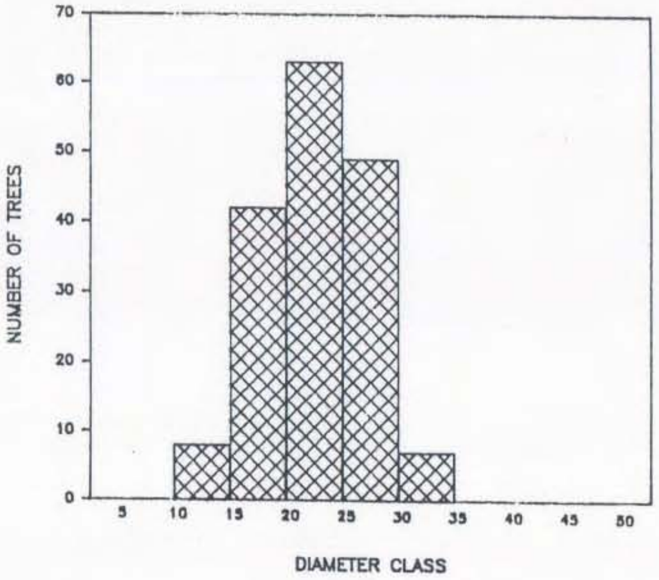

UNIT 11 PLOT 2

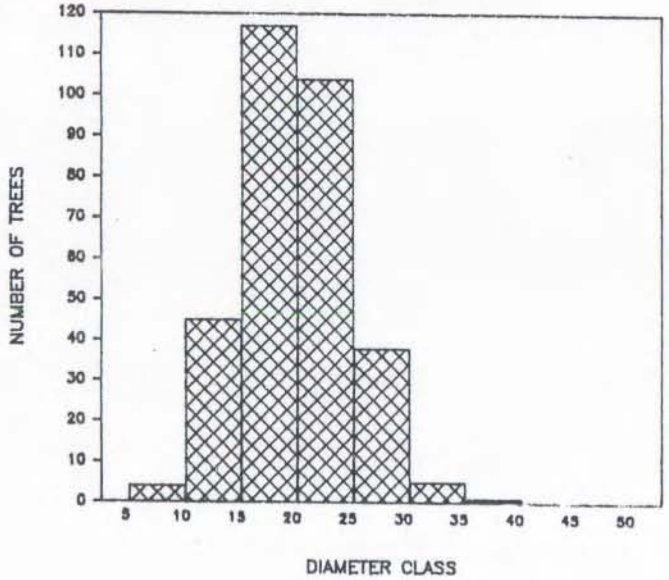

UNIT 12 PLOT 3

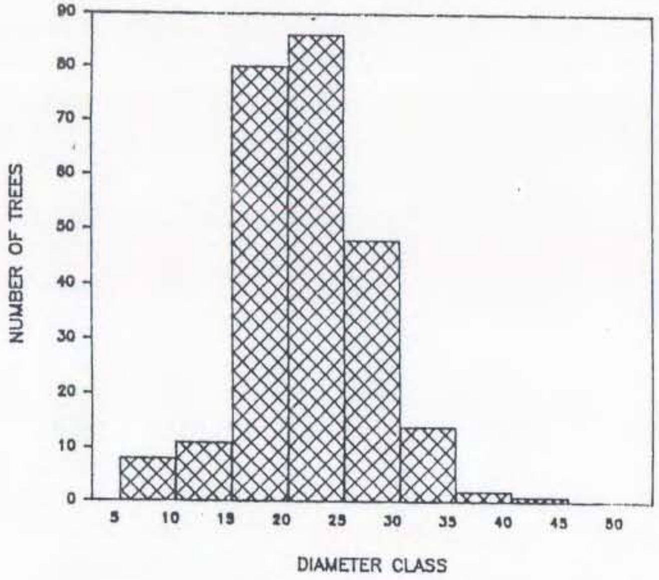

UNIT 12 PLOT 2

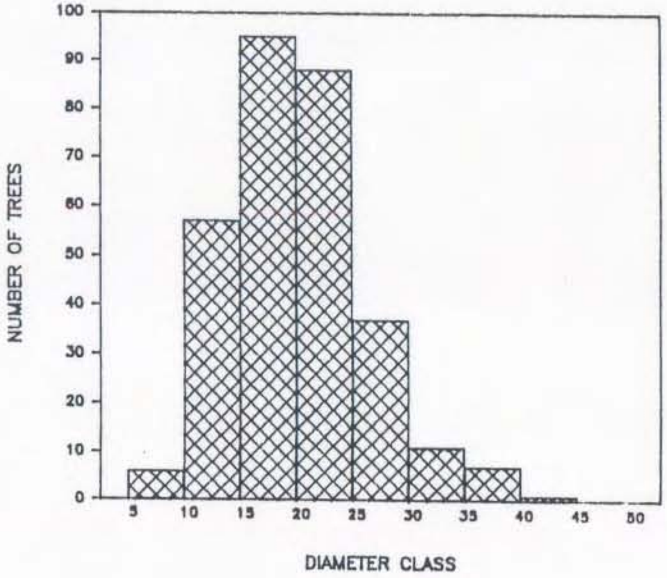

UNIT 12 PLOT 1

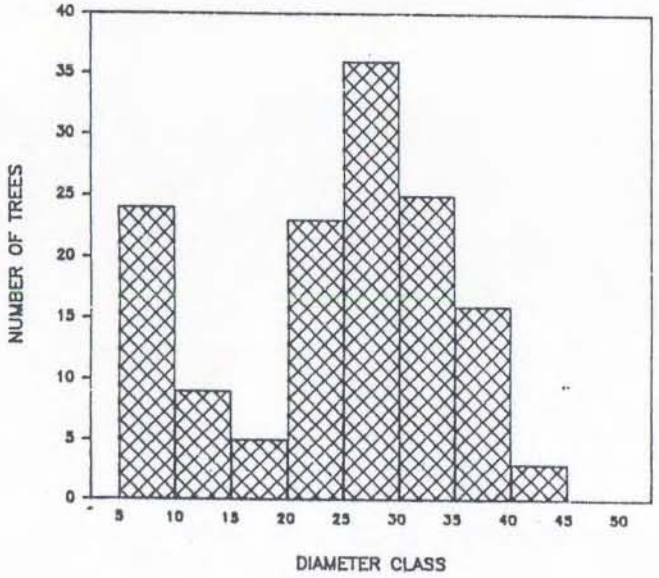

UNIT 13 PLOT 1

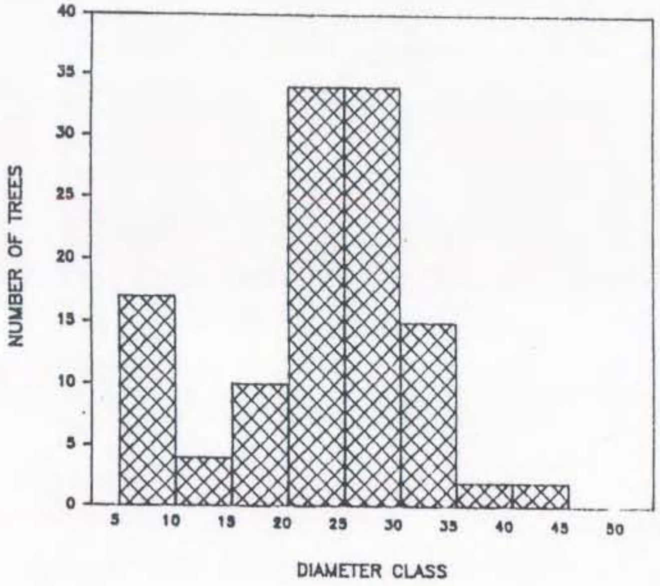

UNIT 13 PLOT 3

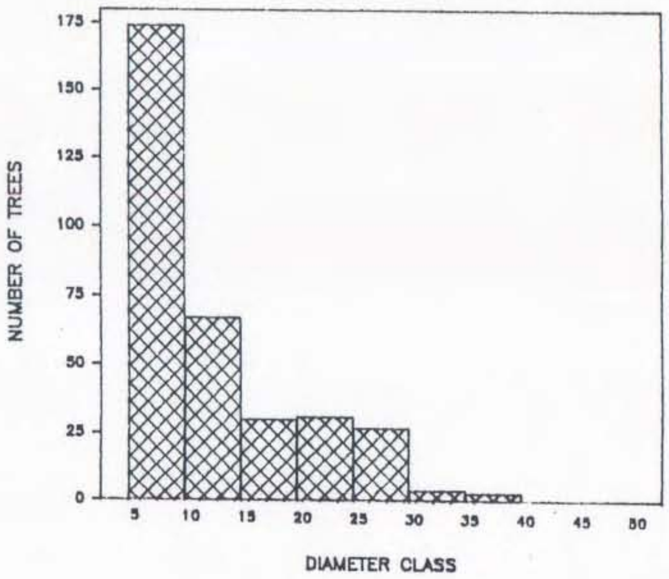

UNIT 13 PLOT 2

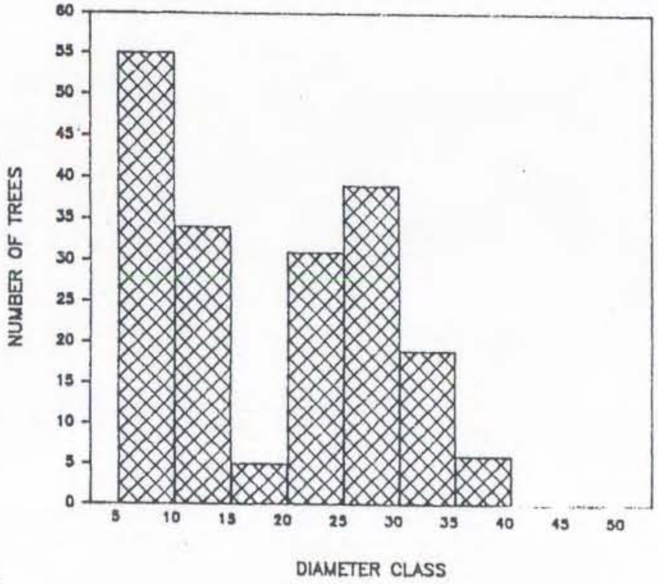

UNIT 14 PLOT 2

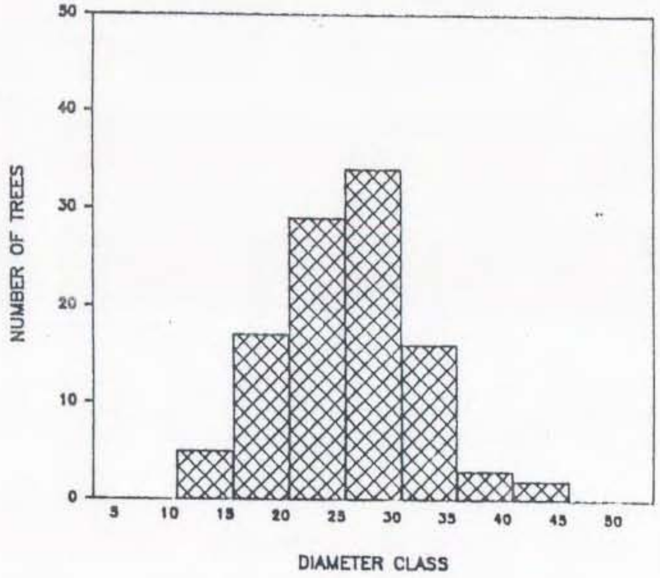

UNIT 14 PLOT 3

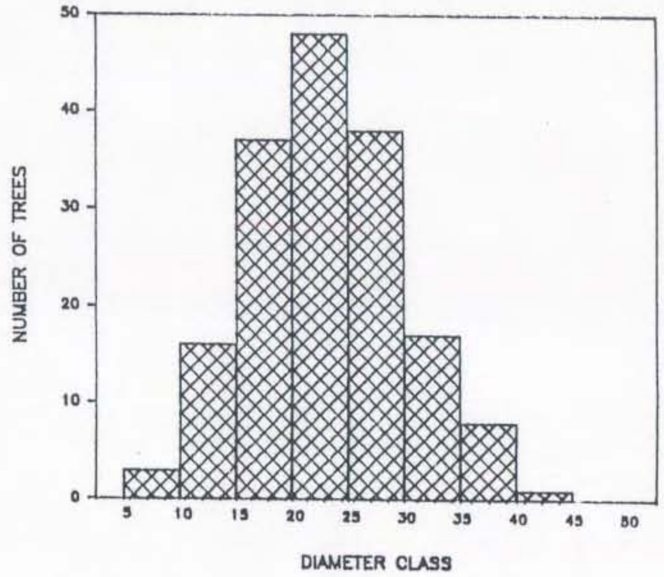

UNIT 14 PLOT 1

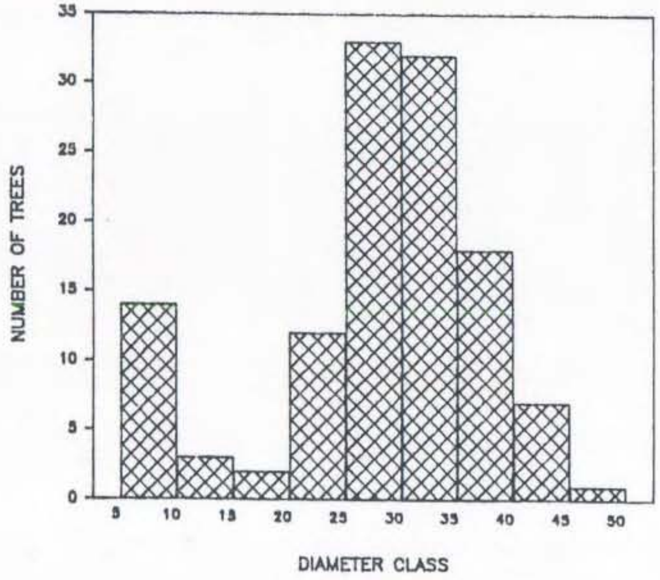

UNIT 15 PLOT 2

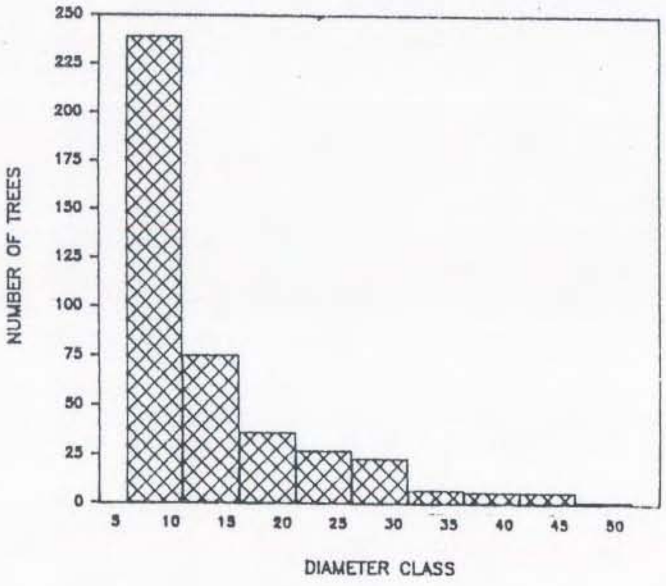

UNIT 15 PLOT 3

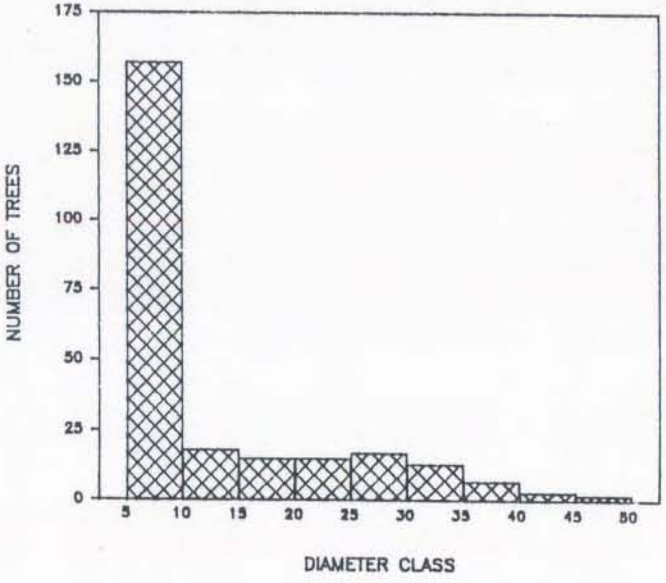

UNIT 15 PLOT 1

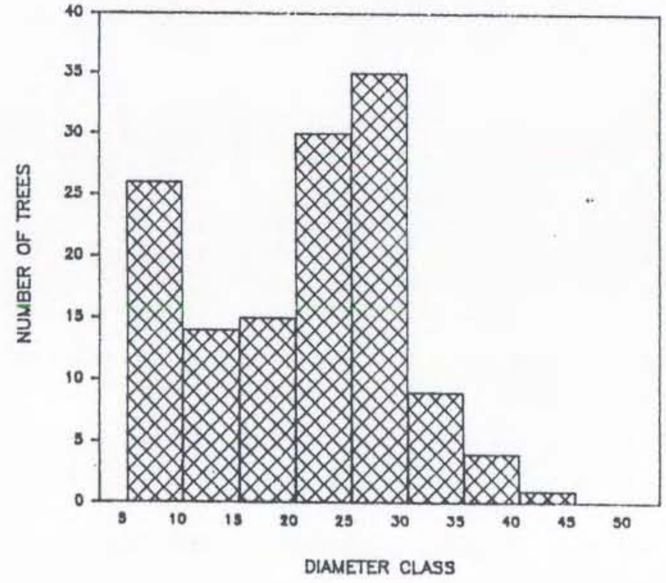

UNIT 16 PLOT 3

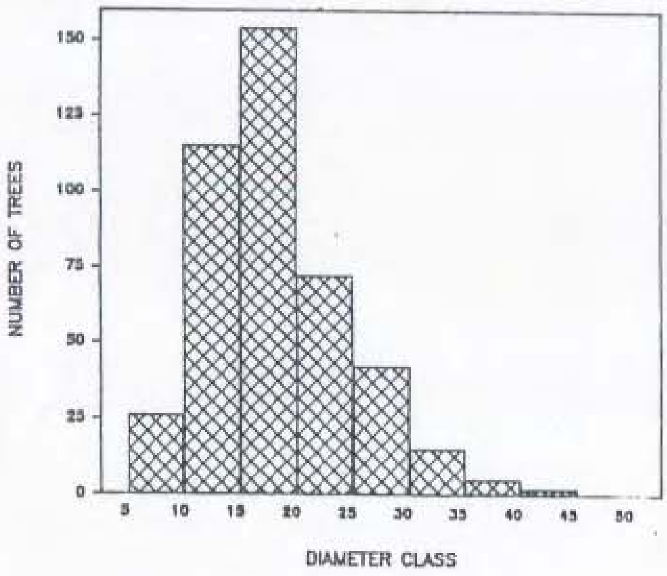

UNIT 16 PLOT 1

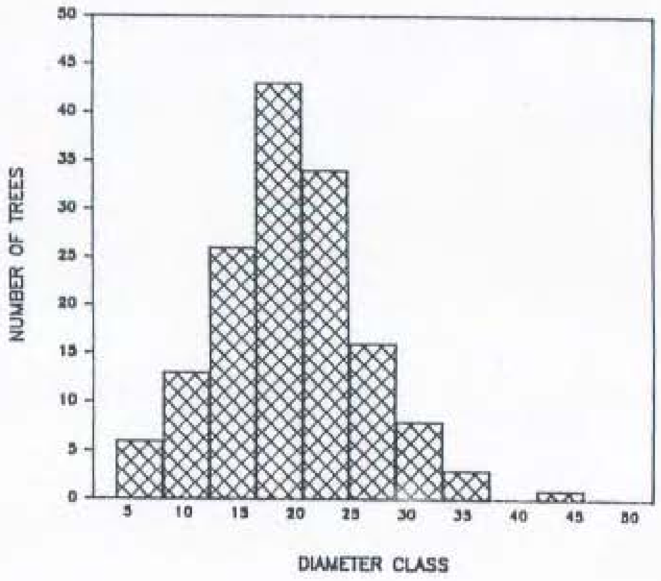

UNIT 16 PLOT 2

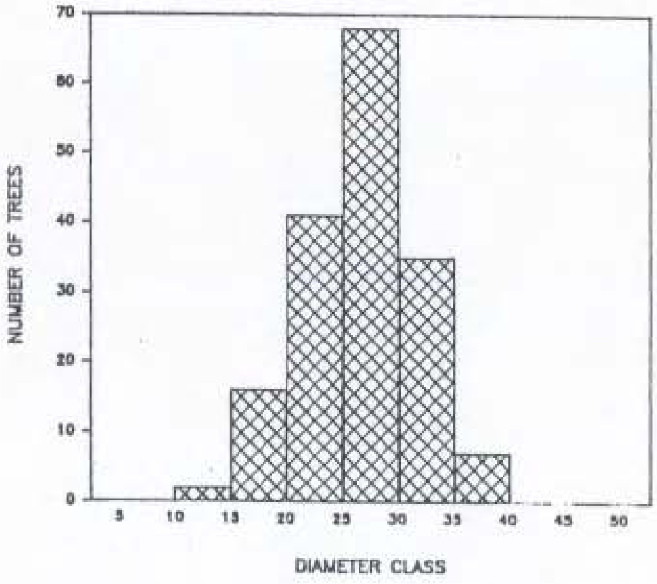

UNIT 17 PLOT 2

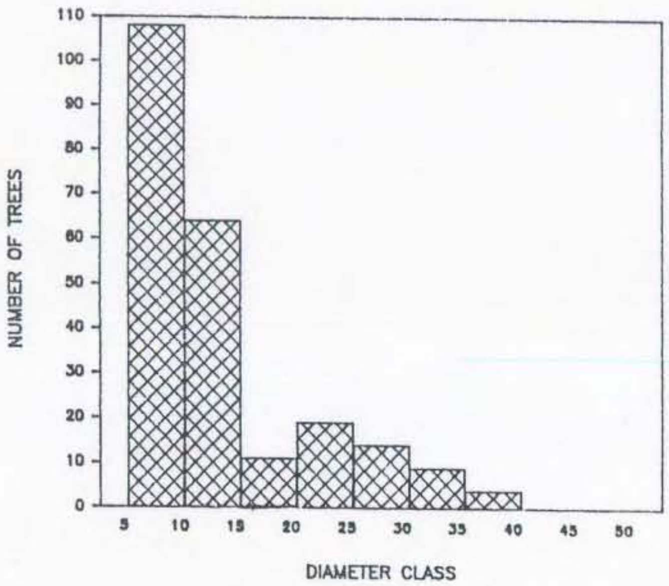

UNIT 17 PLOT 3

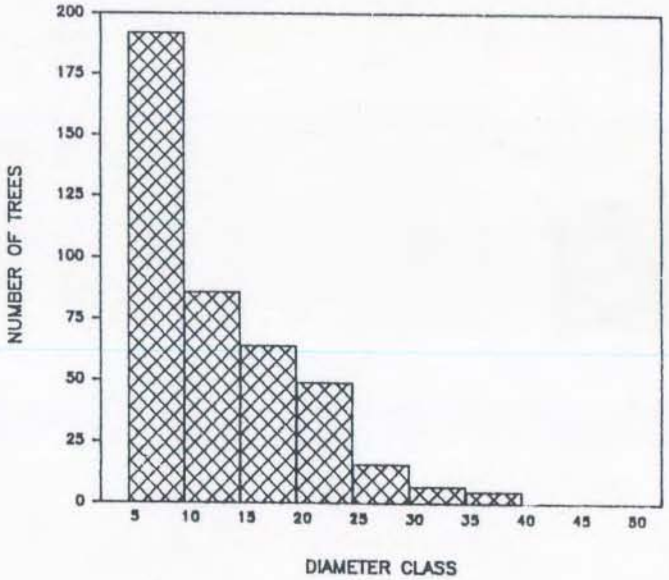

UNIT 17 PLOT 1

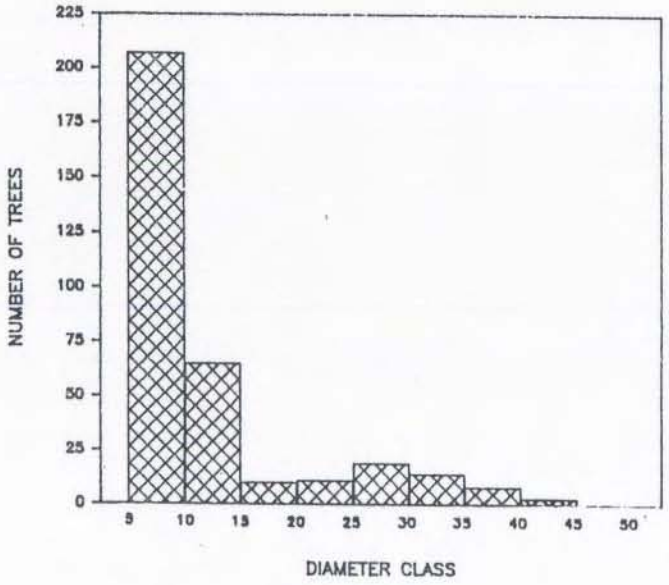

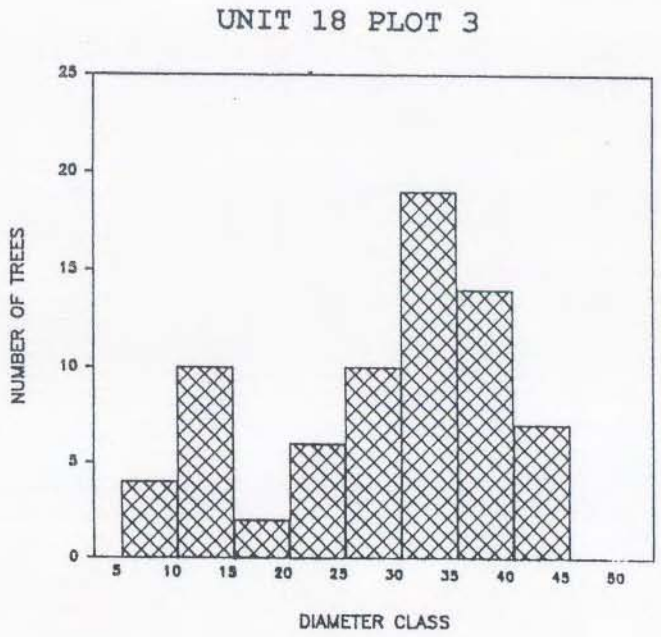
UNIT 18 PLOT 3
NUMBER OF TREES
0 5 10 15 20 25
5 10 15 20 25 30 35 40 45 50
DIAMETER CLASS

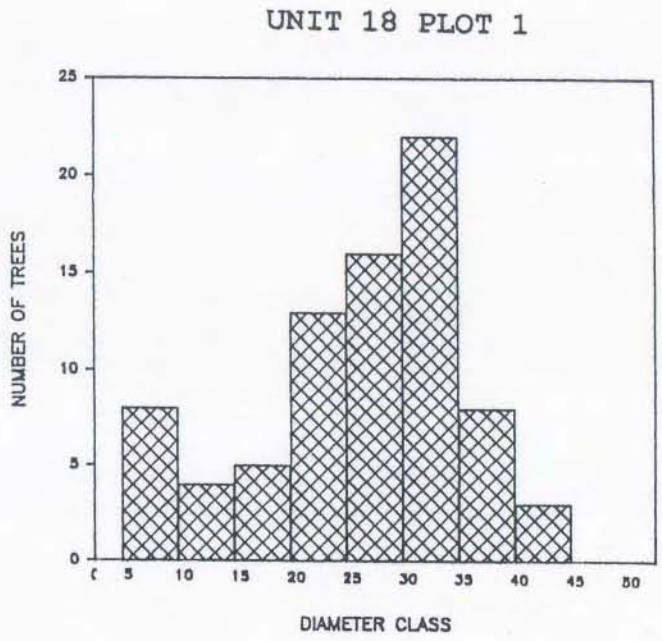
UNIT 18 PLOT 1
NUMBER OF TREES
0 5 10 15 20 25
5 10 15 20 25 30 35 40 45 50
DIAMETER CLASS

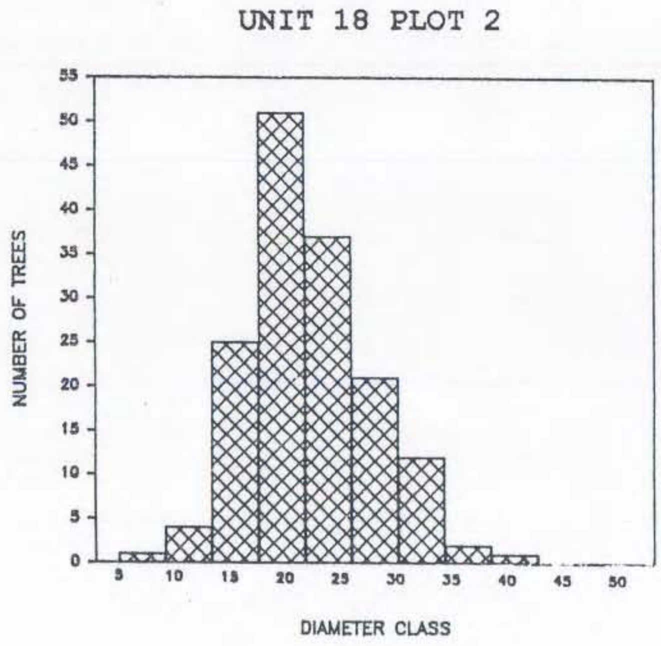
UNIT 18 PLOT 2
NUMBER OF TREES
0 5 10 15 20 25 30 35 40 45 50 55
5 10 15 20 25 30 35 40 45 50
DIAMETER CLASS
